# On the NF-Y regulome as in ENCODE (2019)

**DOI:** 10.1371/journal.pcbi.1008488

**Published:** 2020-12-28

**Authors:** Mirko Ronzio, Andrea Bernardini, Giulio Pavesi, Roberto Mantovani, Diletta Dolfini

**Affiliations:** Dipartimento di Bioscienze, Università degli Studi di Milano, Milano, Italy; University of Ottawa, CANADA

## Abstract

NF-Y is a trimeric Transcription Factor -TF- which binds with high selectivity to the conserved CCAAT element. Individual ChIP-seq analysis as well as ENCODE have progressively identified locations shared by other TFs. Here, we have analyzed data introduced by ENCODE over the last five years in K562, HeLa-S3 and GM12878, including several chromatin features, as well RNA-seq profiling of HeLa cells after NF-Y inactivation. We double the number of sequence-specific TFs and co-factors reported. We catalogue them in 4 classes based on co-association criteria, infer target genes categorizations, identify positional bias of binding sites and gene expression changes. Larger and novel co-associations emerge, specifically concerning subunits of repressive complexes as well as RNA-binding proteins. On the one hand, these data better define NF-Y association with single members of major classes of TFs, on the other, they suggest that it might have a wider role in the control of mRNA production.

## Author summary

The ongoing ENCODE consortium represents a useful compendium of locations of TFs, chromatin marks, gene expression data. In previous reports, we identified modules of CCAAT-binding NF-Y with individual TFs. Here, we analyzed all 363 factors currently present: 68 with enrichment of CCAAT in their locations, 38 with overlap of peaks. New sequence-specific TFs, co-activators and co-repressors are reported. Co-association patterns correspond to specific targeted genes categorizations and gene expression changes, as assessed by RNA-seq after NF-Y inactivation. These data widen and better define a coherent model of synergy of NF-Y with selected groups of TFs and co-factors.

## Introduction

Eukaryotic genomes contain thousands of protein coding and non-coding genes, and the understanding of their expression is a central issue in biology. In fact, this process regulates development, differentiation and, in some cases, transformation of cells. Gene expression is initiated by production of primary RNAs by RNA Polymerases, RNA Pol II in the case of mRNAs and most regulatory non-coding RNAs. The first event driving transcriptional activation by RNA Polymerase II is the recognition and binding of Transcription Factors (TFs) to specific DNA regulatory elements (promoters and enhancers). At least two additional classes of proteins are minimally required: co-activators and General Transcription Factors (GTFs). While TFs and co-activators are selective for classes of promoters and enhancers, GTFs are believed to partake in the activation of all genes [1]. At a higher level, the process is regulated by chromatin configuration, which either allows–euchromatin–or prevents–heterochromatin–the association of protein complexes to the regulatory elements. Chromatin states are variously associated to a plethora of histones Post-Translational Modifications (PTMs) and to DNA methylations, so that multiple configurations, rather than binary on/off states, have been described. In turn, these chromatin modifications are imparted by the enzymatic activities embedded in many–possibly most–co-activators.

The CCAAT box is a widespread DNA element in mammalian promoters [2–5], with a relatively precise location, from 60 to 100 base pairs upstream of transcription start sites (TSSs). It is found in inducible genes, including cell-cycle regulated, as well as in genes overexpressed in cancer cells [6–8]. The NF-Y trimeric Transcription Factor is the primary–likely the sole–CCAAT-binding activity in all eukaryotes [9]. The three subunits–NF-YA, NF-YB, and NF-YC–form a complex that binds DNA with high sequence-specificity and affinity. The NF-Y/CCAAT 3D structures of Aspergillus and mammals [10,11] show numerous protein contacts–>40 –over a considerable length of DNA (25/28 bp). NF-Y bends DNA severely–angle of 80°–serving also as an “architectural” TF, that is, bringing distal TFs closer to the GTFs around the TSS. Elimination of NF-Y from promoters by RNAi of one subunit, or overexpression of a Dominant Negative NF-YA mutant, is detrimental for functional recruitment of many TFs [12 and References therein]. The genes of the three NF-Y subunits are extremely conserved in all eukaryotes, and their importance in mammals was documented in conditional models of NF-YA knockout mice in various tissues [13].

An effort to order functional elements of the human genome is made by the ongoing ENCODE consortium [14,15]. This massive catalogue turns out to be informative as far as locations of TFs, chromatin configurations, RNA expression and other features, within the limits of a number of cancer cell lines [16–18]. TFs appear to be bound in groups, often very large ones; genomic locations are catalogued according to 16/18 chromatin states, ranging from highly active to completely repressed, depending on DNase I hypersensitive sites, levels of DNA methylation and neighboring histone PTMs.

NF-YA and NF-YB genomic locations were analyzed by ENCODE in the Tier 1 K562, HeLa-S3 and GM12878 cell lines: in our initial report, location analysis was matched with partial characterization of co-association with 78 available TFs in K562 cells [19]. The peaks of the two NF-Y subunits largely overlapped, and the motif retrieved was the expected logo, originally defined from *in vitro* studies [20]. Binding to enhancers and to LTR repetitive sequences was matched to different chromatin configurations [17–19]. These data were confirmed by further analysis [12, 21–23]. The novelty was the association to numerous regions devoid of positive histone PTMs, suggesting that NF-Y is a “pioneer” TF driving the opening of chromatin territories. This conclusion was later supported by numerous studies: (i) analysis of DNase I hypersensitive sites through machine-learning methods [24]; (ii) ChIP-seq and RNAi analysis in mES cells indicating that NF-Y promotes chromatin accessibility to Oct4, Sox2, and Nanog [25]; (iii) studies of chromatin opening in the very initial stages– 2/4 cells stage–of mouse development [26]; (iv) studies on LEC1/AtNF-YB9 as a crucial epigenetic determinant of somatic embryogenesis in Arabidopsis [27].

In the second round of ENCODE analysis, we identified classes of TFs which bind the same promoters and enhancers, in some cases with a precise arrangement of sites [12]. We catalogued the interplays in three distinct categories, based on the presence of CCAAT in the TF peaks, of peaks overlap with NF-Y without CCAAT, or of both conditions. We integrated data with protein-protein interactions and the characterization of the target genes after NF-Y-inactivation. We proposed a model whereby NF-Y is a pioneer only for selected classes of TFs and cofactors, rather than a widespread facilitator of binding of most TFs.

In this report, we pursued the studies on the NF-Y regulome on the ever-growing ENCODE datasets, incorporating expression and chromatin configuration data, as well as RNA-seq results generated by us after NF-Y inactivation.

## Methods

### ChIP-seq datasets

We considered all available ENCODE ChIP-seq datasets from K562, GM12878 and HeLa-S3 cell lines. Coordinates of “Optimal IDR thresholded peaks” were retrieved from the ENCODE repository (as of January 31^st^ 2019) as “bed narrowPeak” file type. Peaks available only on the hg38 assembly were converted to hg19, resulting in an initial number of 728 experiments. Since in some cases different experiments were available for the same TF, we filtered this initial dataset as follows. Duplicate experiments for the same TF in the same cell line were processed as previously described [28]: first of all, a total of 277 duplicate experiments performed with antibodies directed against a Tagged protein were removed. We further discarded all experiments (minima) with less than 10000 peaks or less than half of the peaks of the other replicates for the same factor. Finally, only experiments with replicates with overlap higher than 66% were kept, and the one with highest number of peaks was used for downstream analyses. TFs with replicate experiments not satisfying the latter condition were discarded altogether. Filtering resulted in 519 unique experiments with no replicates in the same cell line.

### Motif enrichment analysis

Motif enrichment analysis was performed with PscanChIP, a tool that given a set of peak summit coordinates evaluates Global and Local enrichment of TFs binding motifs in genomic regions surrounding the peaks [29].

Global enrichment estimates over-representation of TFBS motifs in the provided regions compared to a genomic background, computed on all regions of the genome available for TF binding. A reasonable estimate for the latter can be identified by DNaseI hypersensitivity. PscanChIP built-in genomic backgrounds thus include background expected matrices scores to which scores of matrices within input regions are compared, resulting for each matrix in a p-value expressing the probability of obtaining the same score difference with a set of randomly chosen genomic regions. A motif whose assigned p-value is significant for global enrichment could correspond to the actual binding site of the TF for which the ChIP-seq experiment was performed (usually the most significant one) or to binding sites of TFs co-associating with it across the genome.

Local enrichment evaluates instead over-representation of TFBS motifs with respect to genomic regions flanking those derived from the ChIP-seq. In particular, the higher the probability to find the motif close to provided peak summits, the lower the obtained p-value. A globally enriched motif usually is locally enriched, as well. A motif locally but not globally enriched indicates the binding of a factor colocalizing with the one analyzed by ChIP-seq, but only in a limited subset of regions.

For both measures, the enrichment was considered significant when the relative p-value was lower than 10^−10^, in order to keep only the most robust correlations. For experiments on the K562 cell line, the cell-specific background of PscanChIP was employed, while for GM12878 and HeLa-S3 cell, for which a cell specific background was not available, enrichment was assessed with respect to the “mixed background” option. Regions were scanned by PscanChIP with the JASPAR 2020 Redundant matrix collection, and the CCAAT-box matrix employed to evaluate its enrichment was MA0060.1, as in previous work [12].

### Positional bias analysis

PscanChIP predicts the presence of a positional bias between peak summits and the matrix of the factor (when available). We considered as positive scores those whose p-values lower than 10^−10^. Thereafter, each factor positive for the presence of CCAAT was first verified for the actual co-presence of NF-Y peaks, and then precise distances were computed using each of the corresponding matrices present in JASPAR 2020 Redundant version.

### Peak co-association analysis

The computation and statistical evaluation of peak overlap was performed as recently described [28]. Briefly, the overlap between two ChIP-seq peak sets was computed by counting the number of summits of the first TF falling within 300 bp regions centered on the peak summits of the second. This corresponds to setting a maximum distance between summits of 150 bp. To assess the significance of the overlap, that is, evaluate the probability of finding a given number of overlaps by chance, we employed an estimate of the number of accessible regions for TF binding with an approach similar to the one used for evaluating global enrichment of motifs. That is, this value was estimated as the number of 150 bp-width DNaseI hypersensitive regions in the cell lines employed. With small differences, this value had an average of 250000, which is the value we employed in our calculations [12,28]. Statistical significance was evaluated according to a Poisson distribution, p-values were Bonferroni corrected, and the -Log_10_ of the p-values was used for producing the clustered heatmaps. Overlaps with observed values lower than expected ones were further multiplied by -1, to distinguish between factors with overlap significantly higher, or lower, than expected in the presentation of data.

Overlap regions between two factors within NF-Y peaks were computed with the same criterium as above, employing the peaks summits overlapping NF-YB for both factors and using the number of NF-YB peaks for the computation of the p-values associated with the overlap.

Overlaps were finally considered significant when the -Log_10_ of the p-value was greater than 100. In particular, we considered NF-YB significantly overlapping with a factor if more than 10% of its peaks are shared with the factor.

Heatmaps were obtained by clustering the log-transformed p-values, as described above, using Pearson correlations and the centroid method.

### Pathways enrichment analysis

Peaks summit of TFs of Table 1 or Table 2, in common with the ones of NF-YB, were annotated with the HOMER software. Genes with a peak in their promoter region (-1000 and +100 from TSS) were submitted for Pathway analysis to KOBAS 3.0 [30]. A matrix with pathways (columns) and TFs binding (rows) was built by keeping terms with p-values lower than 10^−5^. Enriched pathways with a p-value higher than 10^−5^, or a background number of genes higher than 200 were discarded. To reduce the redundancy of pathways and improve the legibility and interpretation of the resulting plot, pathways characterized by the same genes were merged into the most general one. The final plot was produced by employing, and custom editing, the *UpsetR* [31] package.

**Table 1:** Results of the PscanChIP analysis.

	GM12878			HeLa-S3			K562				MCF-7	H1-hESC	A549	IMR-90	SK-N-SH	HEK293T	HCT116
TFs	CCAAT Enriched	Pos Bias	%	CCAAT Enriched	Pos Bias	%	CCAAT Enriched	Pos Bias	%	TFs	CCAAT Enriched	CCAAT Enriched	CCAAT Enriched	CCAAT Enriched	CCAAT Enriched	CCAAT Enriched	CCAAT Enriched
**ARID3A**		No	7	N.D.				No	5	**ARID3A**		N.D.	N.D.	N.D.	N.D.	N.D.	N.D.
**ASH1L**	N.D.			N.D.				No	29	**ASH1L**	N.D.	N.D.	N.D.	N.D.	N.D.	N.D.	N.D.
**ATF1**	N.D.			N.D.				No	11	**ATF1**	N.D.	N.D.	N.D.	N.D.	N.D.	N.D.	N.D.
**ATF2**	N.D.			N.D.				Yes	2	**ATF2**	N.D.	N.D.	N.D.	N.D.	N.D.	N.D.	N.D.
**ATF3**	N.D.			N.D.				Yes	2	**ATF3**	N.D.		N.D.	N.D.	N.D.	N.D.	N.D.
**ATF4**	N.D.			N.D.				Yes	1	**ATF4**	N.D.	N.D.	N.D.	N.D.	N.D.	N.D.	N.D.
**ATF7**		No	7	N.D.				No	6	**ATF7**		N.D.	N.D.	N.D.	N.D.	N.D.	N.D.
**BRCA1**		No	23		No	17		No	0	**BRCA1**	N.D.		N.D.	N.D.	N.D.	N.D.	N.D.
**C11orf30**	N.D.			N.D.				Yes	3	**C11orf30**	N.D.	N.D.	N.D.	N.D.	N.D.	N.D.	N.D.
**CBFA2T2**	N.D.			N.D.				No	4	**CBFA2T2**	N.D.	N.D.	N.D.	N.D.	N.D.	N.D.	N.D.
**CC2D1A**	N.D.			N.D.				No	5	**CC2D1A**	N.D.	N.D.	N.D.	N.D.	N.D.	N.D.	N.D.
**CEBPB**		No	15		Yes	3		Yes	3	**CEBPB**	N.D.	N.D.			N.D.	N.D.	N.D.
**CEBPZ**		No	59	N.D.				No	46	**CEBPZ**	N.D.	N.D.	N.D.	N.D.	N.D.	N.D.	N.D.
**CHD2**		No	21		Yes	17	N.D.			**CHD2**	N.D.			N.D.		N.D.	N.D.
**CREM**		No	12	N.D.				No	8	**CREM**	N.D.	N.D.	N.D.	N.D.	N.D.	N.D.	N.D.
**CTCF**		Yes	2		Yes	1		Yes	1	**CTCF**						N.D.	
**CUX1**		No	8	N.D.				No	3	**CUX1**		N.D.	N.D.	N.D.	N.D.	N.D.	N.D.
**DDX20**	N.D.			N.D.				No	21	**DDX20**		N.D.	N.D.	N.D.	N.D.	N.D.	N.D.
**DEAF1**	N.D.			N.D.				No	22	**DEAF1**	N.D.	N.D.	N.D.	N.D.	N.D.	N.D.	N.D.
**E2F1**	N.D.				No	12		No	10	**E2F1**		N.D.	N.D.	N.D.	N.D.	N.D.	N.D.
**E2F4**		No	30		No	25		No	18	**E2F4**		N.D.	N.D.	N.D.	N.D.	N.D.	N.D.
**E4F1**		No	24	N.D.				No	9	**E4F1**		N.D.	N.D.	N.D.	N.D.	N.D.	N.D.
**ELF1**		No	13	N.D.			N.D.			**ELF1**	N.D.	N.D.	N.D.	N.D.	N.D.	N.D.	N.D.
**FOS**		Yes	90		No	13		Yes	47	**FOS**		N.D.	N.D.		N.D.	N.D.	N.D.
**FOXM1**	N.D.			N.D.				No	7	**FOXM1**	N.D.	N.D.	N.D.	N.D.	N.D.		N.D.
**GABPA**		No	13		No	14		No	11	**GABPA**		N.D.	N.D.	N.D.	N.D.	N.D.	N.D.
**HCFC1**		No	24	N.D.				No	14	**HCFC1**		N.D.	N.D.	N.D.	N.D.	N.D.	N.D.
**HMBOX1**	N.D.			N.D.				No	2	**HMBOX1**	N.D.	N.D.	N.D.	N.D.	N.D.	N.D.	N.D.
**IRF1_0**	N.D.			N.D.				Yes	22	**IRF1_0**	N.D.	N.D.	N.D.	N.D.	N.D.	N.D.	N.D.
**IRF3**		No	46		No	89	N.D.			**IRF3**	N.D.	N.D.	N.D.	N.D.		N.D.	N.D.
**JUN**	N.D.				No	4		No	6	**JUN**				N.D.	N.D.	N.D.	N.D.
**JUND**	N.D.				Yes	4		No	5	**JUND**	N.D.		N.D.	N.D.		N.D.	
**JUN_3**	N.D.			N.D.				No	5	**JUN_3**	N.D.	N.D.	N.D.	N.D.	N.D.	N.D.	N.D.
**KDM1A**		No	3	N.D.				No	1	**KDM1A**	N.D.			N.D.	N.D.	N.D.	N.D.
**MAFF**	N.D.				Yes	3		Yes	1	**MAFF**	N.D.	N.D.	N.D.	N.D.	N.D.	N.D.	N.D.
MAFG*	N.D.			N.D.				Yes	1	**MAFG**	N.D.	N.D.	N.D.	N.D.	N.D.	N.D.	N.D.
**MAFK**		No	4		Yes	5		Yes	2	**MAFK**					N.D.	N.D.	N.D.
**MAX**		No	15		No	9		No	9	**MAX**	N.D.		N.D.	N.D.	N.D.	N.D.	N.D.
**MBD2**	N.D.			N.D.				No	19	**MBD2**		N.D.	N.D.	N.D.	N.D.	N.D.	N.D.
**MEIS2**	N.D.			N.D.				Yes	4	**MEIS2**	N.D.	N.D.	N.D.	N.D.	N.D.	N.D.	N.D.
**MITF**	N.D.			N.D.				Yes	4	**MITF**	N.D.	N.D.	N.D.	N.D.	N.D.	N.D.	N.D.
**MTA2**		No	4	N.D.			N.D.			**MTA2**		N.D.	N.D.	N.D.	N.D.	N.D.	N.D.
**MYBL2**	N.D.			N.D.				No	11	**MYBL2**	N.D.	N.D.	N.D.	N.D.	N.D.	N.D.	N.D.
**NEUROD1**	N.D.			N.D.				No	15	**NEUROD1**		N.D.	N.D.	N.D.	N.D.	N.D.	N.D.
**NFE2**		No	47	N.D.				No	2	**NFE2**	N.D.	N.D.	N.D.	N.D.	N.D.	N.D.	N.D.
**NFIC**		No	5	N.D.				Yes	2	**NFIC**	N.D.	N.D.	N.D.	N.D.	N.D.	N.D.	N.D.
**NF-YA**		No	96		Yes	70		Yes	82	**NF-YA**	N.D.	N.D.	N.D.	N.D.	N.D.	N.D.	N.D.
**NF-YB**		Yes	100		Yes	100		Yes	100	**NF-YB**	N.D.	N.D.	N.D.	N.D.	N.D.	N.D.	N.D.
**NR2F1**		No	5	N.D.				No	2	**NR2F1**	N.D.	N.D.	N.D.	N.D.	N.D.	N.D.	N.D.
PBX2*	N.D.			N.D.				Yes	5	**PBX2**	N.D.	N.D.	N.D.	N.D.	N.D.	N.D.	N.D.
**PBX3**		Yes	20	N.D.			N.D.			**PBX3**	N.D.	N.D.	N.D.	N.D.	N.D.	N.D.	N.D.
**PKNOX1**		Yes	12	N.D.				Yes	7	**PKNOX1**		N.D.	N.D.	N.D.	N.D.		N.D.
**RAD21**		Yes	2		Yes	2		Yes	1	**RAD21**					N.D.	N.D.	N.D.
**RAD51**		No	11	N.D.				Yes	12	**RAD51**		N.D.	N.D.	N.D.	N.D.	N.D.	N.D.
**RBM25**	N.D.			N.D.				No	7	**RBM25**	N.D.	N.D.	N.D.	N.D.	N.D.	N.D.	N.D.
**RCOR1**		No	12		No	9		No	5	**RCOR1**		N.D.		N.D.		N.D.	N.D.
**RFX1**	N.D.			N.D.				No	5	**RFX1**	N.D.	N.D.	N.D.	N.D.	N.D.	N.D.	N.D.
**RFX5**		No	40		Yes	14		No	21	**RFX5**				N.D.		N.D.	N.D.
**RUNX3**		Yes	6	N.D.			N.D.			**RUNX3**	N.D.	N.D.	N.D.	N.D.	N.D.	N.D.	N.D.
**SIX5**		No	26	N.D.				No	20	**SIX5**	N.D.		N.D.	N.D.	N.D.	N.D.	N.D.
**SMC3**		Yes	3		Yes	2		Yes	2	**SMC3**	N.D.	N.D.			N.D.	N.D.	N.D.
**SP1**		Yes	27	N.D.				No	17	**SP1**			N.D.	N.D.	N.D.		N.D.
**TBP**		No	17		No	14		No	12	**TBP**	N.D.		N.D.	N.D.	N.D.	N.D.	N.D.
**TOE1**	N.D.			N.D.				No	7	**TOE1**		N.D.	N.D.	N.D.	N.D.	N.D.	N.D.
**USF1**		No	23	N.D.				Yes	13	**USF1**	N.D.		N.D.	N.D.	N.D.	N.D.	N.D.
**USF2**		No	24		Yes	13		Yes	28	**USF2**	N.D.					N.D.	N.D.
**ZBTB40**		No	10	N.D.				No	4	**ZBTB40**		N.D.	N.D.	N.D.	N.D.	N.D.	N.D.
**ZNF143**	N.D.				No	12		Yes	5	**ZNF143**	N.D.		N.D.	N.D.	N.D.	N.D.	N.D.
**ZNF24**		No	4	N.D.			N.D.			**ZNF24**		N.D.	N.D.	N.D.	N.D.	N.D.	N.D.
ZNF507*	N.D.			N.D.				No	0	**ZNF507**		N.D.	N.D.	N.D.	N.D.	N.D.	N.D.

The factors significantly enriched in CCAAT motif presence in the proximity of peak summits are listed in alphabetical order, in the three Tier 1 cell lines. Asterisks indicate the use of tagged proteins by ENCODE. Pos. Bias indicates the presence of a Positional bias among the peak summits of the factor and CCAAT. The percentage of TF peaks overlapping those of NF-YB is indicated. For the other cell lines shown, only the presence of CCAAT by PscanChIP is reported. In dark green: factors with Global enrichment of CCAAT as primary binding motif. In green: factors with Global enrichment of CCAAT as secondary binding motif. In light green: factors with Local enrichment of CCAAT. In red: No CCAAT enrichment. In yellow: new factors. No Data are referred to as N.D.

**Table 2:** Analysis of peaks overlap.

	GM12878		HeLa-S3		K562	
TFs	Overlap Score YB	% YB	Overlap Score YB	% YB	Overlap Score YB	% YB
BHLHE40	291	**17**	N.D.		no	
CBFB	228	**18**	N.D.		N.D.	
CCNT2	N.D.		N.D.		245	**20**
CREB3L1	N.D.		N.D.		125	**17**
E2F6	N.D.		173	**8**	293	**17**
E2F8	170	**8**	N.D.		164	**13**
ELF4	N.D.		N.D.		267	**21**
ELK1	323	**11**	323	**13**	107	**5**
ELK4	N.D.		292	**12**	N.D.	
ETS1	288	**14**	N.D.		121	**11**
GABPB1	N.D.		N.D.		231	**26**
GTF2F1	N.D.		323	**19**	N.D.	
HDGF	118	**16**	N.D.		N.D.	
HMGN3	N.D.		N.D.		271	**17**
HNRNPLL	N.D.		N.D.		302	**18**
IRF1_1	N.D.		N.D.		149	**12**
IRF1_2	N.D.		N.D.		176	**14**
KDM5B	N.D.		N.D.		149	**19**
KLF5	287	**15**	N.D.		N.D.	
MAZ	323	**27**	323	**23**	N.D.	
MNT	N.D.		N.D.		250	**18**
MTA3	no		N.D.		166	**23**
MXI1	323	**25**	323	**23**	241	**12**
MYC	no		267	**17**	183	**25**
MYC_0	N.D.		N.D.		155	**18**
MYC_2	N.D.		N.D.		177	**28**
MYC_3	N.D.		N.D.		163	**13**
NFATC3	113	**17**	N.D.		N.D.	
NFE2L2	N.D.		132	**10**	N.D.	
NR2C1	238	**12**	N.D.		no	
NRF1	323	**10**	148	**6**	323	**21**
PML	N.D.		N.D.		121	**17**
POLR2A	323	**26**	N.D.		N.D.	
POLR2A_0	N.D.		N.D.		323	**24**
POLR2A_1	N.D.		N.D.		323	**26**
POLR2A_2	N.D.		N.D.		323	**24**
POLR2A_3	N.D.		N.D.		323	**26**
POLR2AphosphoS2	269	**15**	323	**22**	no	
POLR2AphosphoS5	208	**26**	N.D.		146	**23**
POLR2B	N.D.		N.D.		102	**16**
POLR2H*	N.D.		N.D.		110	**17**
RB1	100	**14**	N.D.		no	
SIN3A	323	**16**	N.D.		126	**12**
SMAD5	305	**13**	N.D.		323	**24**
TAF1	323	**23**	323	**28**	323	**24**
TBL1XR1	179	**14**	N.D.		no	
TCF7L2	N.D.		153	**18**	no	
YY1	126	**24**	N.D.		243	**14**

### Analysis of chromatin states

Cell line-specific chromatin states employed were retrieved from the RoadMap Epigenome repository [32]; in particular, the “Core 18-state model (6 marks)” mnemonic file was used. Peaks summit of either factors with CCAAT enrichment and significant overlap with NF-YB, or factors with only CCAAT enrichment, were assigned to the corresponding chromatin states.

### Other tools

Analyses were performed with both Python (2.7) and R (3.2.5) in-house scripts. Employed Python libraries were *pybedtools*, *pandas* and *numpy*, whereas R packages used were *gplots*, *UpsetR*, *tidyverse* collection and DESeq2. Conversion of coordinates between different genome assemblies was performed with the LiftOver tool available at the UCSC Genome Browser [33].

### Cell culture, siRNA transfections and Western blot analysis

HeLa cells were grown in DMEM high glucose with L-glutamine (EuroClone) supplemented with 10% FBS, 100 U/mL penicillin and 100 μg/mL streptomycin. The day before transfection, 0.15 × 10^6^ cells/well were seeded in antibiotic-free medium in a 6-well plate. Cells at 25–30% confluence were transfected with 50 nM siRNA (control pool of scramble oligos and NF-YB siRNA J-010002-08-0002, ON-TARGET*plus*, Dharmacon) using 3.75 μL Lipofectamine 3000 Reagent (ThermoFisher) in 1.5 mL final volume of Optimem (ThermoFischer) per well. 16 hours post-transfection, cells were detached by trypsin treatment, pooled and split in new wells. 72 hours post-transfection cells were harvested for protein extracts and RNA preparation. Three independent inactivation experiments were performed. The RNAs were isolated using TRI-reagent (Merck) and further purified with RNeasy Mini Kit (Qiagen) following the RNA clean-up protocol. RNAs were then quantified with Nanodrop and RNA integrity assessed with Agilent Tapestation. Total protein extracts were prepared in RIPA buffer and used for Western blotting. The membrane was probed with primary antibodies and secondary HRP-conjugated secondary antibodies (Sigma Aldrich). Primary antibodies: anti-NF-YA (G2, Santa Cruz Biotechnologies), anti-NF-YB (GeneSpin), and anti-Vinculin (Sigma Aldrich) as loading control.

### RNA-seq experiments and analysis

Total RNAs were poly-T purified, randomly fragmented and transformed in cDNA with NEB library preparation protocol. Library preparation and sequencing were performed by Novogene. Sequencing was performed with the following requirements: paired end with 150 nt read length and at least 30 million tags for each sequencing. FASTQ files were retrieved, and sequencing quality was evaluated by FastQC software. Tags were mapped with RSEM 1.2.11 against human transcriptome (GRCh37/hg19). Differentially expressed genes (DEGs) were evaluated with DESeq2 [34], with thresholds FDR<0.01 and |Log_2_FC|>1. Promoters of DEGs were further analysed for Transcription Factors Binding Sites (TFBS) enrichment using Pscan [35] with the JASPAR 2016 set of matrices. Expression data after inactivation of NF-YB and ChIP-seq data were merged. Factors significantly overlapping with NF-YB and factors with peaks summit significantly CCAAT enriched were clustered in two different heatmaps according to Log_2_FC values of genes. In particular, for each of the two states (UP and DOWN) a pairwise analysis on a Fisher test was run given the number of genes regulated by two factors TF(a) and TF(b) and the total number of genes regulated by the single factor. The obtained p-values were -Log_10_ transformed and employed to build a heatmap by hierarchical clustering with Pearson distance and centroid as clustering method. RNA-seq raw data and processed data are available at GEO under the accession number GSE151237.

## Results and discussion

### Outline of the bioinformatic analysis

**Fig 1:**
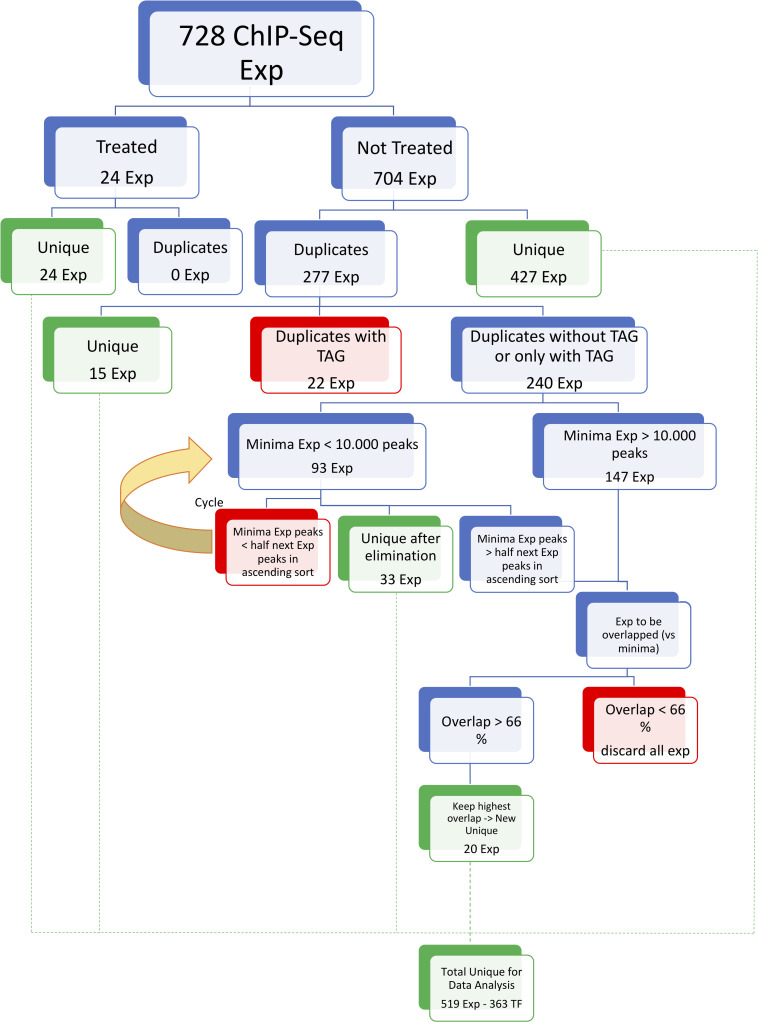
Workflow of data preparation for the bioinformatic analysis.

The general workflow of our analysis of ENCODE data is outlined in Fig 1. The 728 ChIP-seq experiments of K562, HeLa-S3 and GM12878 were considered because of the availability of ChIP-seq data of NF-Y; they were divided in Not-treated (704) and Treated (24): this latter category refers to factors–including RNA Pol II–whose binding was monitored after treatment of cells with various stimuli. The former group was further divided in Unique (427 ChIP-seqs in the three Tier 1 cells), directly inserted in the pipeline, and duplicates (277 experiments). 22 ChIP-seq experiments performed with Tagged overexpressed TFs were discarded because of the concomitant presence of ChIP-seq made with antibodies against the endogenous TF. 240 ChIP-seqs were in duplicate only in one of the two conditions, that is with Tagged proteins, or with antibodies against the endogenous TF: they were both considered. However, some of these duplicate experiments were very heterogeneous, both in the number of peaks, and in overlap of sites within duplicates, and sometimes triplicates. To avoid analysis of spurious data, or cherry picking some of these datasets, ChIP-seqs of TFs for which all replicates have a minimal number of peaks >10000 were automatically considered. For those in which there was one–or more–replicate with <10000 peaks, we discarded all experiments that shared <50% of overlap of peaks with the next ascending experiment of the same factor. The duplicate experiments selected were further processed so that only those with >66% overlap were considered. If more than two experiments had >66%, the highest overlap was considered. This brought the total number of ChIP-seq experiments to be included in our analysis to 519 (Fig 1).

Before we proceed with the description of the findings, a few additional issues need clarifications.

1. The ENCODE consortium has progressively fine-tuned processing of data: our previous analysis–as of October 2014 –was performed on the available datasets denoted as “SYDH” at the UCSC Genome Browser [12]. Subsequently, “uniform” datasets were produced, where the original raw data were re-processed with a uniform pipeline and more stringent quality controls, especially on the concordance of replicate experiments. All new data are now available at www.encode.org. This also entails differences in the nomenclature of some of the TFs and in peaks distribution (number and locations) for most. While this does not change–and in most cases reinforces–the global picture of the 154 TFs previously analyzed, it does lead to the elimination of a few factors from the previous lists (See below).
2. ENCODE contains ChIP-seq data of many cell types. NF-Y locations were analyzed in three cell lines, K562, GM12878 and HeLa-S3: thus, we initially considered only factors whose ChIP-seq data are available in these cells; thereafter, for all factors showing significant overlap with NF-Y in any of these lines, the CCAAT box was searched in the respective peaks for all other lines.
3. ENCODE has also analyzed NF-YC in the hepatic HepG2 cells. We did analyze this dataset and came to the conclusion that CCAAT is indeed enriched in the peaks (Not shown). However, we were puzzled by the very high number of peaks, as compared to the Tier 1 lines, as well as to other NF-Y ChIP-seq experiments reported independently from the ENCODE consortium. Most importantly, parallel analysis of the TFs analyzed in HepG2 shows an overlap of peaks with the vast majority of the factors (~80%). This was in striking contrast to the observation previously–and currently–made in Tier 1 cell lines, for which the overlap is around 20%. For these reasons, we felt that further refinement is required for the NF-YC HepG2 data, as they currently stand, before we could include them in our pipeline.

Having gathered data with these stringent criteria, we initially performed two types of experiments: (i) evaluation of the enrichment of the CCAAT matrix in the peaks of ChIP-seq experiments of all TFs and cofactors, using the PscanChIP software. This method classifies motif enrichment as “Global” or “Local” and signals a positional bias of motifs–CCAAT in this case–with respect to peaks summits [28]. (ii) Computation of overlaps between NF-Y peaks and those of all other factors, irrespective of CCAAT enrichment with PscanChIP: this validates the enrichment of CCAAT, as it formally proves that the identified CCAAT are indeed NF-Y-bound, and it also identifies co-association with additional factors. We previously detailed why we think this latter group is relevant [12].

### Analysis of CCAAT enrichment

PscanChIP assesses the enrichment of a given matrix in the summits of ChIP-seq peaks [29]: for NF-Y, matrix NFYA MA0060.1 in JASPAR 2020 Redundant, which is identical to the one previously used by us [12]. We applied it to the summits for the 519 ChIP-seq experiments selected, corresponding to 363 TFs of three cell lines. For all factors, the output is either negative–no enrichment of CCAAT in the peaks–or positive, defined in different ways. This was then integrated by another layer of analysis: computing of peaks overlaps between NF-Y and the individual factor, allowing verification as to whether NF-Y is actually bound to the enriched CCAAT. These types of information allowed us to define different levels of positivity (Table 1). “Global” enrichment of CCAAT in the peaks signals a very high frequency, that is, that a sizable fraction of peaks contains a CCAAT motif. This could take the form of “primary binding site” (Dark Green in Table 1), namely the CCAAT box, rather than the TF’s own binding site, can be singled out to be the main sequence responsible for the TF binding to DNA, as determined by co-bound ChIP-seq runs. This does not imply that the factor actually binds the CCAAT box, but rather that NF-Y might be responsible–directly or indirectly–for its recruitment to the location. A second possibility is that CCAAT is a “secondary binding site”, found together with the TF’s expected matrix, which is the most significantly enriched one according to the analysis. In this case, co-operative binding with NF-Y in many *loci* is proposed (Green in Table 1). Finally, the CCAAT box could be found to have significant “local” enrichment only (Light Green in Table 1), signaling close binding of NF-Y and the factor, but to a more limited set of genomic *loci*, likely in specific gene families.

The analysis of the 363 ENCODE factors in K562, HeLa-S3 and GM12878 identified a total of 68 proteins (Table 1), not including NF-YB and NF-YA, run as positive controls and returning the expected high significance of enrichment for the CCAAT motif. Most factors (48) are sequence-specific TFs, which represent the majority of proteins analyzed by ENCODE. The number is more than twice that (33) obtained in our previous analysis of 154 factors [12], yet this fraction of the total number of experiments considered is similar, 19% in this analysis, 21% in the previous one. All factors previously identified are present, with the expected “global/local” partitioning, except Sp2 and SRF. The elimination of the former is due to their removal from ENCODE after the reprocessing of data with the “uniform” quality criteria (as discussed below). Also, SRF, previously catalogued as “local”, is absent in the K562 reprocessed data, while the three ChIP-seq experiments of GM12878 were eliminated by our stringent criteria of inclusion because of insufficient overlap among the replicate experiments. Two factors changed their nomenclature: Co-REST is now RCOR1, and CDP is now CUX1. Most of the additions are either in the “local” group (Light Green) or in the “global” with “secondary binding site” (Green). The only addition catalogued as “global” with “primary binding site” is PKNOX1.

ChIP-seq datasets were then analyzed for overlap of peak summits with those of NF-Y. For this, we only considered the peaks of NF-YB, which showed a larger–and more robust–cohort than NF-YA [19]. For this task, we measured the percentage of NF-YB summits within a distance of 150 bp from the peak summits of each of the other TFs, assessing its statistical significance. In our previous analysis, 29 out 33 factors with CCAAT enrichment, as identified by PscanChIP, had in turn significant overlap, the exceptions being subunits of the Cohesin complex CTCF/SMC3/RAD21 and CUX1 [12]. Here, again, the majority of the 68 factors did show a significant overlap with NF-YB peaks. As expected, the larger overlaps (in percentage of peaks) are found for TFs with “Global” enrichment (Table 1). Interestingly, however, a sizeable cohort of 17 proteins overlapped only marginally with NF-YB (<5%). Among them, we confirm the CTCF/SMC3/RAD21 *trio* and add several members of the Basic-Leucine Zipper (B-Zip) family, such as ATF2/3/4 and MAFG/F; the related MAFK shows borderline overlap (5%, Table 1). The meaning of CCAAT enrichment in sites with little or no significant overlap of peaks will be expanded below.

Looking at Table 1 vertically, most factors are found in K562 (57, with 4 positives in other lines but negatives in this line); GM12878 (25 positives, 13 negatives) and HeLa-S3 (18 positives, 3 negatives). Some factors with “local” enrichments are positive only in one cell line, while most “global” ones are found in all. A possible reason for the cell-type selectivity is the relative abundance of the factor in a given cell type: for example, FOS is lowly expressed in HeLa-S3, as compared to K562; in turn, this can impact on overall peaks number: for E4F1, CEBPB, KDM1, BRCA, for example, we noticed that the “negative” cell lines yield a considerably lower number of peaks. In addition, factors could be bound separately to specific closed chromatin areas of tissue-specific promoters and enhancers in different cell types.

**Fig 2:**
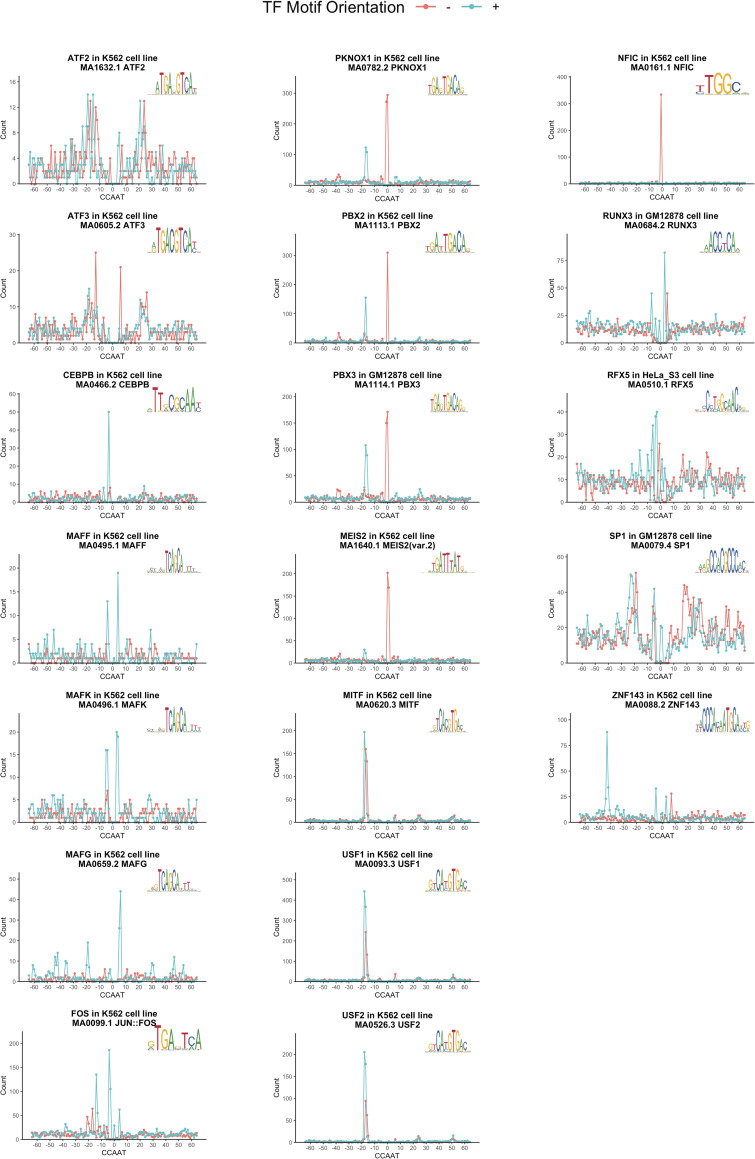
Positional Bias motif distances plot. Distribution of distances between the center of positive TF motifs and the middle A of CCAAT-box. Data obtained from PscanChIP output, given co-binding peak summits, centering on CCAAT and picking the relative TF matrix. Colors state motif direction with respect to the plotted positional weight matrix.

A second important information derived by the PscanChIP analysis is the identification of positional bias within sites. That is, whether the CCAAT box is found as having a spacing preference with respect to the summit of the individual TF (PscanChIP motif-centered analysis). In all, 30 factors showed positional bias, using a rather stringent criteria of positivity, p-value <10^−10^. Almost all are sequence-specific TFs, while most cofactors are negative. We decided to investigate more precisely this issue: for each TF positive according to PscanChIP positional bias score in at least one cell line, we first identified all peaks with CCAAT enrichment and binding of NF-YB, and then computed the distance of the individual matrix of the TF with respect to the central A of the CCAAT pentanucleotide. In addition, we made the following considerations. (i) Positional bias of some notorious interactors, such as FOS and JUNs, were apparently negative in a cell-type dependent way: for example, FOS was positive in K562, but not in HeLa-S3, and *viceversa*, JUND in HeLa-S3, but not in K562. This led us to evaluate the p-values of all factors higher than the original PscanChIP threshold, but still significant. As shown in S1 Table, most TFs do show statistical significance in positioning with CCAAT, even in cell lines scoring negative according to the stringent PscanChIP criteria. This result suggests that whenever a positional bias is scored it is generally carried in different cellular contexts, although at different levels of significance. (ii) Some factors do not have a matrix (c11orf30/EMSY, CHD2, RAD51) and therefore could not be further analyzed. (iii) We chose the JASPAR 2020 Redundant database for analysis, which is the most recent and comprehensive. By so doing, we identified 19 TFs with a positional bias between motifs, as shown in Fig 2 with representative matrices, and, with all data, in S1 Fig. Some of these–Sp1, USF1, USF2, RUNX3, C/EBPB, MAFK, ZNF143– have been discussed before [12,17–19,21,28]. FOS, previously found on double CCAAT boxes, is now detected, as in a previous study [21], also with its consensus “TRE” matrix, 10/12 bp upstream of CCAAT. Note that additional B-Zips scoring positive are ATF2 and ATF3, with little overlap with NF-Y in peaks, but a reasonable positional bias. MAFF has a spacing similar to MAFK, MAFG a different geometry, mostly at the 3’ end of the CCAAT. In addition to USF1/2, another B-HLH TF is MITF, also with an identical distance and positioning (10–12 bp at 5’ of CCAAT). The PBX2 previously reported bias is confirmed [28]: we now extend and better define it to homeodomain TALE members, PBX3, PKNOX–also known as PREP1 –and, to a less extent, MEIS2: they recognize a similar matrix and show a very precise positional bias of 11 bp upstream of CCAAT, as well as overlapping peaks. The functional implications of this finding for cooperative–or inhibitory–interactions are discussed below. NFIC is a new and important addition because of the peculiar geometry, with the matrix overlapping with CCAAT: originally described as CAAT-binding entity, NF1/CTF (CAAT Transcription Factor) recognizes a palindromic sequence based on the CCAA tetranucleotide, indeed part of the NF-Y matrix [36,37]. Because of the high number of co-bound sites, one could exclude that binding of NF1C is mutually exclusive with NF-Y. As nothing is known on the 3D structure of this family of TFs, the structural–and functional–outcome of co-binding will have to be analyzed carefully.

Finally, we extended the PscanChIP analysis of the 68 positive factors to all other ENCODE cell lines for which ChIP-seqs are available. Because of the absence of NF-Y data in these lines, verification based on peaks overlap was impossible. By and large, however, the data on the presence of CCAAT in peaks are consistent with Tier 1 cell lines, both for “global” (CHD2, DDX20, E2F4, IRF3, MBD2, PKNOX1, RAD51, SIX5, USF1/2) as well as “local” (ATF7, JUND, MAFK, ZNF143) connections. In particular, we note cell-type differences in the positivity for CCAAT of these latter TFs.

### Analysis of peaks overlap of all ENCODE factors

In accordance with our pipeline, we extended the general peaks overlap analysis to all proteins present in ENCODE, independently from the enrichment of the CCAAT box motif in their peaks. We calculated overlaps, described by the presence of the peak summits of the factor within 150 bp on either side from the summit of NF-YB peaks. Essentially, there are three reasons for computing these data. (i) Although the window of PscanChIP and peaks overlap analysis is the same– 150 bp–the former assay must contain completely the relatively long (16 bp) NF-Y matrix, whereas in peaks overlap analysis, the summits–made of single nucleotides–are more likely to score positive in the same interval. (ii) The observation of “broad” peaks width of some proteins (usually cofactors) over a sizeable length of DNA: the calculated punctiform summit might in these cases imperfectly reflect the actual binding area. The CCAAT motif could indeed be enriched, but simply missed because of the stringent spacing parameters of PscanChIP. (iii) NF-Y might be indirectly recruited to DNA in the absence of CCAAT, but in the presence of another TF: in this case, this TF might go completely unnoticed in the PscanChIP analysis, in which we considered only enrichment for the CCAAT box.

We applied the same threshold as in our previous study [12]: significance is considered for overlap >10% of NF-YB peaks and Co-association Score >100 (See Methods). A total of 38 proteins have significant overlap with NF-Y (Table 2). With respect to the previous analysis, 15 proteins are confirmed, 5 discarded: POU2F2 because the data were removed by ENCODE; THAP1 and GTF2B because the reprocessed data fall below the overlap threshold of 10% (8% and 7%, respectively). EGR1 and GTF2F1 –the latter only in K562 –because of the modest overlaps between different replicates, leading to elimination in the filtering step of our pipeline. In addition, some factors are present in different datasets of K562 treatments: 2 of IRF1, 4 of MYC and 7 of RNA Pol II A (in addition to subunits B and H). In essence, we have now doubled the number of factors with a significant overlap. Altogether, we felt appropriate to add them to our downstream analysis. To confirm and characterize the results obtained for each of the factors listed in Table 2, we ran PscanChIP exclusively on the subset of *loci* where peaks bound by NF-YB and the said factor overlap. The results, shown in S2 Table, detail that the majority of these factors do show enrichment of CCAAT, and with a “global” signature, as expected, when the analysis is restricted to co-bound regions.

Factors with significant overlap with NF-YB peaks, but not with significant CCAAT enrichment, according to PscanChIP are listed in alphabetical order. The asterisk in POLR2H indicates that a tagged protein was used for analysis. Measurement of the “overlap score” with NF-YB peaks is detailed in [12] and in Methods. The percentage of NF-YB peaks overlapping those of the factor is indicated.

### Different groups of NF-Y co-association

**Fig 3:**
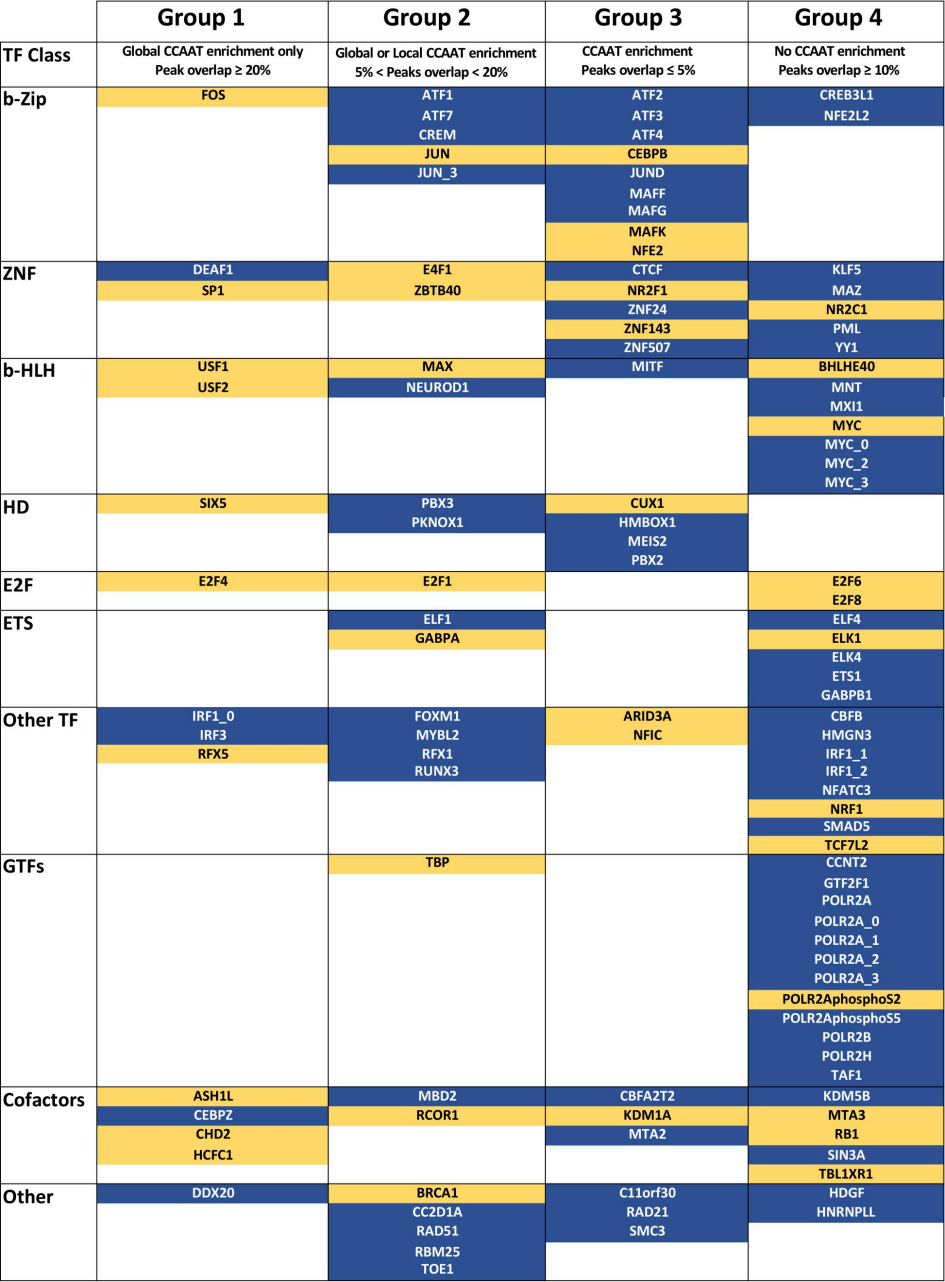
Partitioning of NF-Y associated Factors in four Groups. The Factors are grouped according to different degree of co-association with NF-Y, based on the results in Table 1 and Table 2. In Blue, factors whose data are unanimously present in all cell lines for which experiments are available; in yellow: factors present in one, or more, but not in all cell lines.

The different levels of co-association stemming from the two types of analysis result in classification of four Groups (previously three) (Fig 3). With respect to the previous one, Group 1 is the same; because of the emergence of many proteins with CCAAT but marginal peaks overlap, we split previous Group 2, creating a new Group 3. Previous Group 3, with peaks overlap but no CCAAT, is now Group 4.

#### Group 1

TFs with “global” enrichment for CCAAT as primary or secondary motif and overlap of the NF-Y/TF peaks >20% of the factor peaks in at least one dataset (Dark Green and Green in Table 1). Two factors are moved to Group 2, PBX3 and TBP, because of slight overlap drops in reprocessed data (20% and 17%, respectively). Most TFs–IRF1/3, RFX5, Sp1, E2F4, USF1/2 –have their own motif as primary, unlike FOS. Overall, the current data reinstate their extremely high overlap in the updated ENCODE dataset, with the exception of Sp2, whose absence is merely an issue of different processing of the data and failure to pass more stringent quality controls. In fact, (i) the related Sp1 is present with a “global” enrichment status, in line with the synergistic effect that NF-Y/Sp1 play in dozens of dissected promoters [5,38]. (ii) Independent ChIP-seq performed in MEFs, including in Sp2 KO cells, detailed robust and specific association of Sp2 to double CCAAT locations, by tethering via the Q-rich activation domain of Sp2 [39].

DEAF1, IRF1, ASH1L, CEBPZ, DDX20 are new entries. DEAF1 is a SAND domain TF [40], unnoticed so far as a NF-Y partner; IRF1 joins another member of the family, IRF3. IRF3, involved in transcriptional regulation of immune response genes [41], is devoid of the expected IRF binding motif. A double CCAAT motif can be observed in stimulated K562 cells data of IRF1, present in Group 4. Note that it is positive only after induction by α-IFN (IRF1_0 in Group 1) or γ-IFN (IRF1_1 and IRF1_2 in Group 4), as the TF levels are basally very low. Originally a member of the *Drosophila* “Trithorax” complex, ASH1L is a KMT–Lysine Methyl Transferase–writing H3K36me1/2, a histone mark associated with transcribed regions [42,43]: its presence is not overly surprising, given the overlap of NF-Y with H3K36me3 [19], and the importance of NF-Y binding for deposition of active methyl marks [11]. In addition, HCFC1 is part of the H3K4me MLL complex, and other proteins such as RCOR1 (Group 2), KDM1A (Group 3) and KDM5A (Group 4) also impact on this mark, reinstating a role of NF-Y in organizing recruitment of machines that impinge positively–or negatively–on crucial methylation marks.

DDX20 –Gemin3/DP103 –is intriguing, since it is an RNA Helicase, shown to play dual roles in transcriptional activation–with RNA Pol II and p300 –as well as repression, with HDACs [44,45]. As for CEBPZ, despite the new–and misleading–nomenclature, it is not a member of the CEBP B-Zip family of TFs. It was previously called CBF (CCAAT Binding Factor), originally isolated in expression libraries with a HSP70 CCAAT probe [46]. Interestingly, we previously named the same entity HSP-CBF and showed that (i) it is not a sequence-specific CCAAT-binding protein, and (ii) it is able to coactivate CCAAT promoters in an NF-Y-dependent manner [47]. Thus, the inclusion of CEBPZ in Group 1 extends to the whole genome our previous results: remarkably, 46% and 59% of sites overlap with NF-Y in K562 and GM12878, respectively. Incidentally, CEBPZ was recently shown to be an RNA-binding protein [48], adding to the factors of such category, which surprisingly emerge from our analysis.

#### Group 2

TFs with “global”, or more often “local” CCAAT enrichment (Light Green in Table 1) and peak overlap of the factor >5 and <20%. A sizeable number of factors are in this group, notably with a “local” label. Most of the new entries belong to well represented TF families: ATF1/7 (B-Zip), E4F1 and ZBTB40 (ZNF), NeuroD1 (B-HLH); RFX1 joins the related RFX5 (Group 1) activating MHC Class II genes, well known to be coregulated by NF-Y and RFX [49]. We find a robust link between NF-Y and the TALE homeodomain subfamily, which controls patterning and differentiation. PBX3 and PKNOX1 are in Group 2, PBX2 and MEIS2 in Group 3. CCAAT boxes were reported in the locations of PBX1, another member of this family, when associated with PREP1/PKNOX1 [50]. There are four very relevant twists in our findings. First, PKNOX1 and MEIS2 are competitors for PBX interactions [51]: finding of the former in Group 2 (CCAAT present with NF-Y) and of the latter in Group 3 (CCAAT present but no NF-Y) is a suggestion of mutually exclusive binding of MEIS2/PBX with NF-Y at CCAAT sites. Dynamic knock-down/overexpression experiments are required to verify this hypothesis. Second, along the same lines, positional bias are found, particularly with PBX/PKNOX1, taking the form of co-binding on CCAAT, or of highly selective positioning 11 bp upstream of CCAAT. Note that the upstream site is hardly evident with MEIS2. Third, NF-Y/PREP1/PBX complexes have been dissected biochemically *in vitro*: an important role is played by Sp2, which binds to composite NF-Y/PBX sites, favoring stabilization of the binary complexes [39]. Fourth, the interplay is evolutionarily conserved in *Zebrafish*, where it promotes Zygotic Transcriptional Activation, the earliest event of gene expression in development [52,53]. The NF-Y/TALE interplay is a typical example of genomic studies inviting further structural characterization of complexes at the biochemical level, to gain a better comprehension of the synergistic *vs* opposing functional effects.

In Group 2, we find many subunits of repressive complexes. FOXM1 and MYBL2 are part of the DREAM complex [54], together with E2F4 (Group1): this is not surprising, since CCAAT and CDE-CHR elements, bound by DREAM, are known to cooperate in regulation of cell-cycle G2/M promoters. We previously showed that NF-Y RNAi leads to removal of E2F4, believed to be one of the DNA-binding components of the complex, from such promoters [12]. Another repressive protein is MBD2, which “reads” methylated DNA as a subunit of the NURD complex [55]; note that we find other proteins of this complex: MTA2 in Group3, MTA3 [56] and SIN3A [57] in Group 4. RCOR1 (CoREST) is part of another–predominantly but not exclusively–repressive complex with KDM1A (Group 3). We also find other proteins impacting on mRNA biology: RBM25, a factor involved in exon inclusion [58] and HNRNPLL, involved in alternative splicing process (Group 4) [59]; TOE1 is a Deadenylase and a 3' exonuclease of telomerase RNA [60], whose inclusion in the list is not immediately obvious to rationalize.

#### Group 3

TFs showing enrichment of CCAAT, but peaks overlap of factor <5%. This group is newly created because of the number of factors in this condition: the crucial issue, in this case, is related to mutually exclusive binding with NF-Y. In addition to the above mentioned PBX2/MEIS2, the NF-Y/CTCF (with RAD21 and SMC3) connections–or rather lack of–are intriguing: in mouse ES cells, in fact, there is overlap between CTCF and NF-Y upon neuronal differentiation, but not in growing cells [25], suggesting that it might be cell-type specific or related to the growth/developmental status of the cell. A third important class potentially undergoing selectivity are B-Zip TFs. Most members of this class analyzed by ENCODE–except BACH1, BATF, CREB3, FOSL1, JUNB, NFE2L1 –are among NF-Y partners. Yet, they are essentially split in two groups: those with NF-Y-bound CCAAT nearby in Group 2 –FOS, ATF1/7, JUN, CREM–and those apparently avoiding NF-Y, such as small MAFs, JUND, NFE2 and ATF2/3/4 (Group 3). All these TFs tend to bind to TRE (TPA Responsive Element) or related motifs, which are indeed found next to CCAAT with a strong positional bias in a statistically significant number of promoters (Fig 2): this is a further indication of potential synergism with selected members of the B-Zip family, and mutually exclusive binding with others. The interplays will have to be further dissected with representative members of these TFs in biochemical experiments and dynamic experiments *in vivo*. An additional TF whose binding might be mutually exclusive with NF-Y is CUX1 [61]. Finally, C11orf30 –better known as EMSY [62,63]–is a large BRCA2 –and HP1-interacting protein, involved in transcriptional repression, DNA repair and control of genomic stability [64–67]: yet another indication that NF-Y binding is not inevitably connected to gene activation.

#### Group 4

Factors of Table 2 with >10% of NF-YB peak overlap, but no significant enrichment for CCAAT in PscanChIP. The key point is that many factors are members of larger families present in Table 1 –E2F6/8, B-HLH, ETS, ZNF–or of complexes with other subunits present in Groups 2–3, such as MTA3, SIN3A. This might signal a different behavior of individual members of a family. The cases of ETS and B-HLH TFs are illustrative. ETS domain proteins all recognize a similar motif [68]. All members of this class analyzed by ENCODE–GABP, ELK1, ELF1, ELK4, ETS1– are present in our list; only ELF1 and GABP show enrichment for CCAAT boxes in their peaks. Nevertheless, dissection of the genomic ERK2 pathway in hESCs identified pivotal ELK1 sites, further validated by ChIP-seq experiments, as well as CCAAT boxes and Sp1, E2F, NRF1 sites [69]. Most B-HLH are in this Group, particularly MYC/MNT/MXI1, whose obligate partner for sequence-specific DNA-binding MAX is in Group 2; only USF1/2 are in Group 1. As for E2Fs, those analyzed by ENCODE all correlate with NF-Y: E2F4 and E2F1 are in Groups 1 and 2, while E2F6/8 in Group 4. We previously reported that the NF-Y/E2F4 partnership is associated with repression on *nucleosome* and *protein DNA complex* genes, activation on *DNA replication* and *mitosis* targets. CCAAT and E2Fs sites are the most enriched in promoters of genes overexpressed in cancer: originally observed in profiling experiments [8], we–and others–are confirming the presence of this *duo* in systematic analysis of RNA-seq TCGA data of epithelial cancers [70–75]. Furthermore, the NF-YA and E2F1/3 genes are overexpressed in cancers. It is somewhat surprising not to find CCAAT in E2F6/8 sites; this might be due to technicalities–antibodies, for example–or it could reflect the reported lack of distance bias among CCAAT and E2F sites [19]. Note that E2F6, structurally devoid of an activation domain, is a repressor, thus adding to the growing list of factors of this category.

Except for TBP, in Group 2, and GTF2B (TFIIB), all GTFs present in ENCODE are in this group: the TFIID subunit TAF1, involved in Initiator recognition [76], the P-TEFb subunit CCNT2, involved in RNA Pol II elongation [77], and the RNA Pol II associated GTF2F1 (TFIIF). Incidentally, finding GTF2F1, whose function is linked to that of GTF2B [78] suggests that the absence of the latter is merely due to technical problems in some of the ChIP-seq replicates. The negativity of GTFs in PscanChIP might be due to the strict window constraints of such analysis: GTFs sitting on the edge of TSS are at the limit of detection in the -80/-100 CCAAT locations. In our previous analysis, we did not analyze RNA Pol II peaks: we find that subunit A, analyzed under multiple inducing conditions, B and H are present, as well as CTD phosphorylation of Serine 2 and 5. This result is in line with the diffusion of CCAAT in core promoters (some 25% overall), the notorious location of CCAAT at a relatively fixed position from the TSS [9], and the recent, important discovery that NF-Y dictates the positioning of TSS preference in CCAAT promoters, thus potentially serving a TBP-like role [79]. NF-Y removal induced a lack of RNA Pol II recruitment to ER-stress promoters under basal conditions [80], suggesting a role of NF-Y binding prior to RNA synthesis.

### Co-association modules

**Fig 4:**
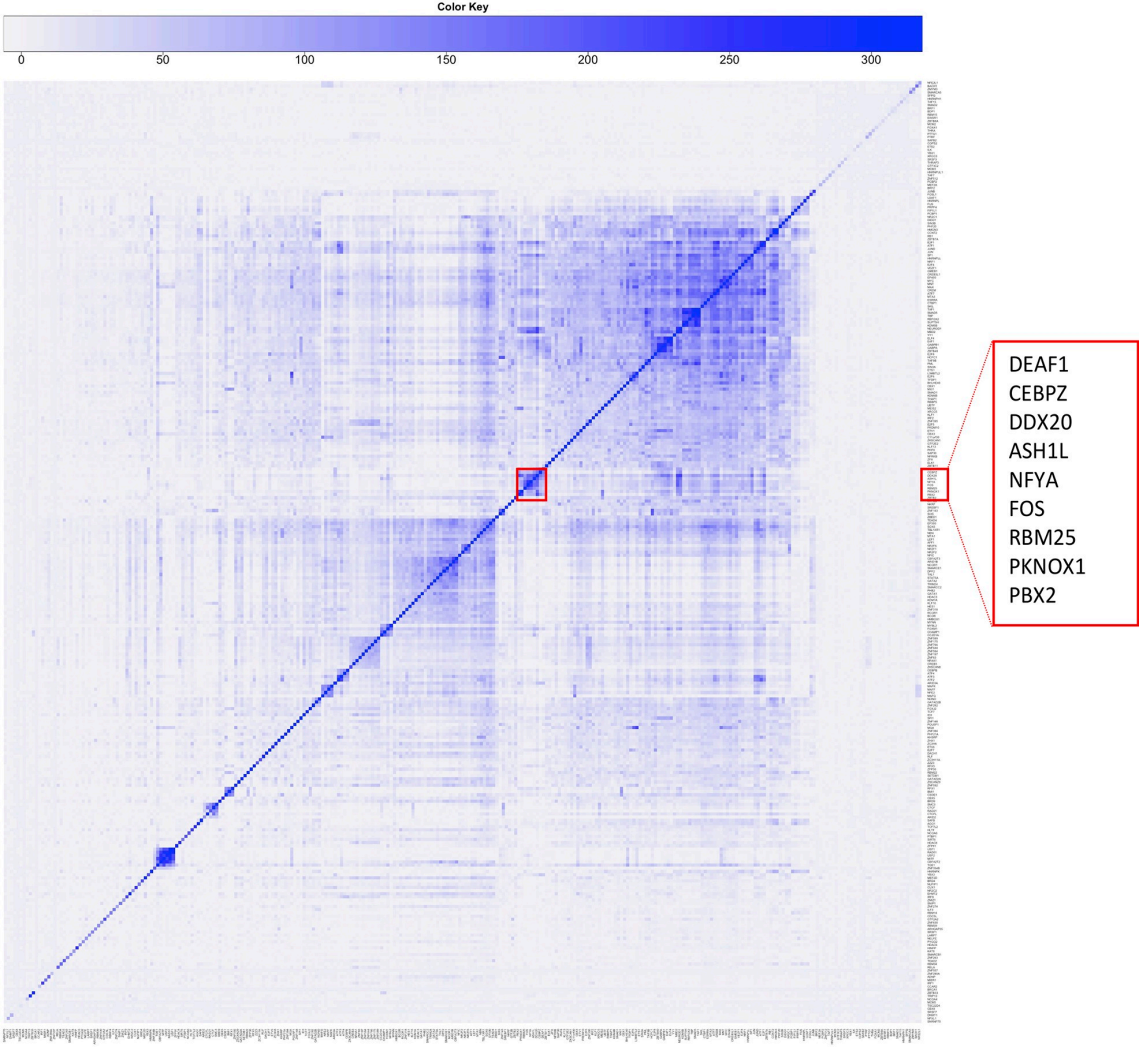
Co-association analysis among all NF-Y-coassociated factors in K562. Pairwise co-association scores restricted to regions co-bound by NF-YB and all other factors, in K562. Scores are defined as -Log 10 of the p-value if the overlap is higher than expected, Log 10 of the p-value otherwise. NF-Y cluster is highlighted in the red box.

TFs tend to co-associate in regulatory regions. We assessed overlap and its significance according to the number of peak summits located within 150 bp from one another in K562 cells, including those not associated with NF-Y. This allowed to derive a global co-association map for all TFs, as discussed before [28]. We represented the results with a matrix built according to co-association scores computed from the p-value associated with the significance of the overlap (S3 Table). The data show that NF-YA/NF-YB cluster with CEBPZ and USF2 only, with most other factors found in three sizeable mega-clusters. Zooming into the NF-YB peaks, thus measuring co-associations in NF-Y-bound regions, the picture is more informative, as determined graphically by the use of heatmaps: the intensity of color in a cell (*x*,*y*) is proportional to the significance of the overlap between factors *x* and *y*. NF-YA peaks are clustered with DEAF1/CEBPZ/DDX20/ASH1L/FOS/RBM25/PKNOX1/PBX2 (Fig 4, K562). Note the presence of RNA-binding proteins RBM25, DDX20, and of CEBPZ. In reality, this is part of a much larger cluster containing >100 factors that include all K562 Group 1 factors, except USF1/2 and SIX5.

To extend these results, co-associations were derived in a pairwise manner among the NF-Y partners present in Table 1 or Table 2, by clustering the co-association matrix, thus highlighting TFs that tend to co-localize when binding together with NF-Y. At a global level, NF-YA and NF-YB peaks are on the edge of a very large cluster of TFs, GTFs and other factors, that includes RFX5 and FOS (S2A Fig). Several separate sub-clusters are visible: MAFs and ATF2/3/4, HMBOX1/CUX1/NF2F1/KDM1A/CBFA2T2, the expected CTCF/SMC3/RAD21 (with ZNF143) and PBX2/PKNOX1/MEIS2. Restricting analysis to NF-YB peaks, PBX2/PKNOX1 –but again, not MEIS2– joins the smaller NF-YA cluster, containing FOS/ASH1L/RBM25/DDX20, bordering with CEBPZ and DEAF1 (S2B Fig). As expected, some GTFs are clustered together, as are MYC/MAX/MNT, whereas ETS, E2Fs and other B-HLH proteins are partially overlapping, but often subclustered in separate groups.

The same type of approach was used for GM12878 (S3A and S3B Fig) and HeLa-S3 data (S4A and S4B Fig). In GM12878, NF-Y is close to FOS, CEBPZ in a well-defined sub-cluster, away from the two mega-clusters. In the NF-YB-restricted peaks, instead, PBX3/PKNOX and IRF3 joins the NF-Y sub-cluster, as part of a mega-cluster which includes many of the TFs of Tables 1 and 2, with additional factors such as RB1, STAT1 and SMAD5. Note that, again, CTCF/RAD21/SMC3 are in a different sub-cluster, away from NF-Y. In HeLa-S3 cells, a large cluster is visible already in the global analysis, centered on NF-Y, with E2Fs, ETS factors (GABPA, ELK1/4), MAZ, TAF1, NRF1 (S4A Fig). This is the case in the NF-YB-restricted peaks (S4B Fig), in which additional factors are added: TBP with the GTFs, MYC, MAX and MXI1 together with RCOR1, BRCA1, RFX5, MAZ and CHD2. NF-Y is also close to IRF3, and FOS on the opposite edge.

In summary, Groups 1/4 TFs tend not only to connect to NF-Y singularly, but also to be clustered together, binding the same regions and forming discrete regulatory modules, further supporting the classification made above.

**Fig 5:**
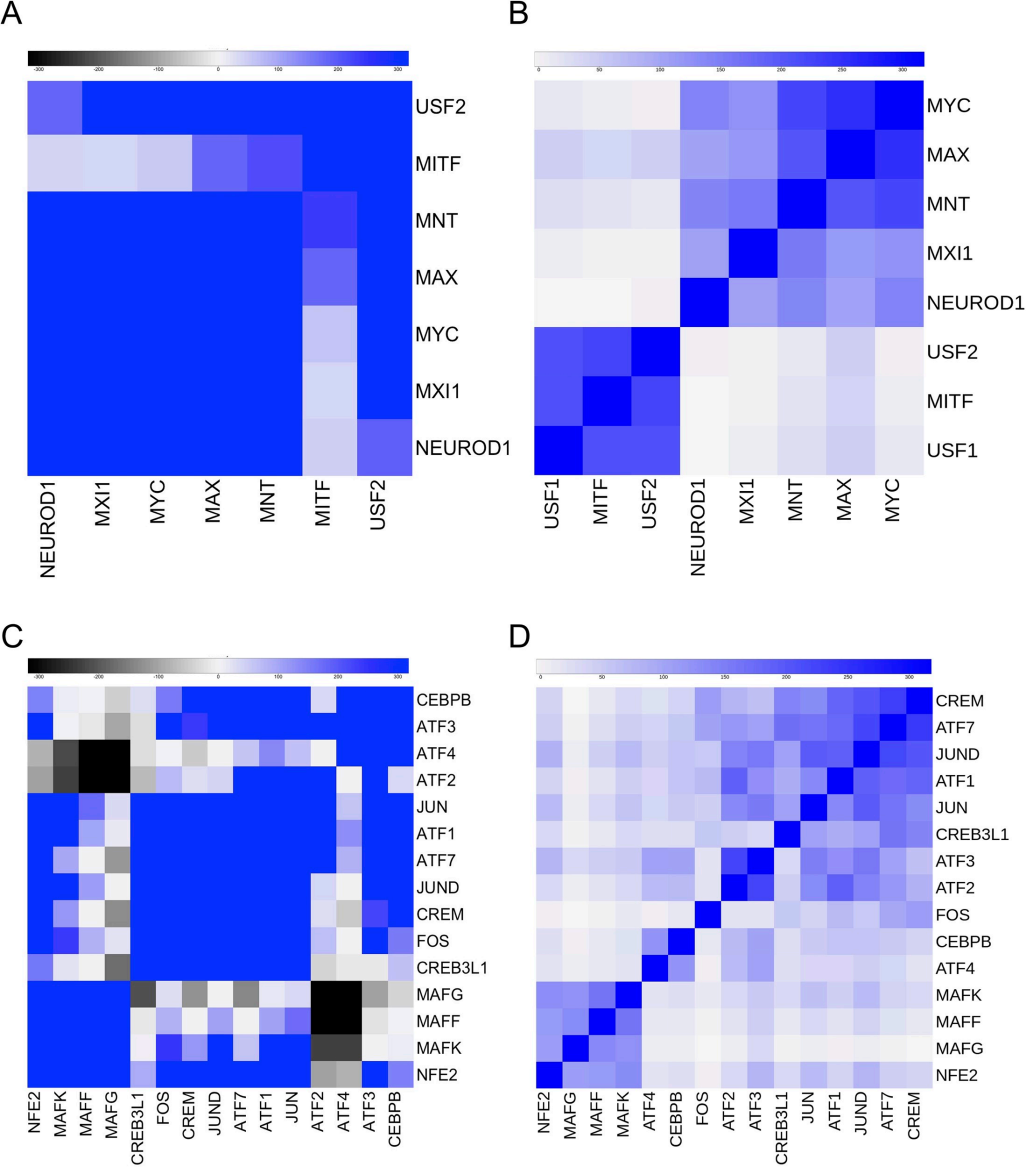
Co-association analysis of B-HLH, B-Zip. Clustering of a subset of TFs of K562. **A**. Heatmap of pairwise co-association scores of B-HLH TFs. **B**. Same as A of B-HLH TFs, restricted to NF-YB-bound regions. **C**. Heatmap of pairwise co-association scores of B-Zip TFs. **D**. Same as C of B-Zip TFs, restricted to NF-YB-bound regions.

We then looked more deeply at two TFs family with large NF-Y overlaps, B-HLH and B-Zip. Concerning the formers, we show heatmaps of K562 genome-wide and NF-YB-specific peaks (Fig 5A and 5B). There appear to be two opposite patterns: the first is represented by MYC and the related MXI1, recognizing similar, if not identical, E box matrices, and showing robust overlaps with NF-YB (12/28% of NF-YB peaks); note that MYC is positive in four different datasets. Yet, CCAAT is visibly missing in their peaks, signaling that they are unevenly positioned around E boxes, with no fixed distance. At the opposite, USF1/2 are confirmed among close partners of NF-Y: “global”, with secondary binding (E box being primary), high peak overlaps (13/28% of USF1/2 peaks) and very strong positional bias between sites, precisely measured at 10/12 bp (Fig 2) [12,19]. One group of sites bound by both NF-Y and USFs is repetitive sequences of the HERV subtype, which are mostly associated to inactive chromatin (See below) [16,19]. Between these two patterns, we find the rest of the B-HLH TFs: MAX and the pathways-specific NEUROD1 (Group 2) have CCAAT, but relatively low overlaps; MITF (Group 3), with a strong positional bias, essentially identical to that of USF1/2, but also possibly avoiding CCAAT and NF-Y-binding near the targeted E boxes.

As for B-Zip TFs, the heatmaps in K562 of genome-wide and NF-YB-restricted peaks show separate sub-groups (Fig 5C and 5D): a cluster of MAFs, with NF-E2, present globally and in NF-YB peaks; a global cluster with C/EBPB, ATF4 and ATF3, with the latter separated to a new cluster in NF-YB peaks. All other B-Zips are in a large cluster globally and in NF-YB peaks, with the exception of ATF2 with ATF3, and FOS. In summary, MAFs and ATF2/3/4 form subgroups, largely independent from NF-Y locations. An expected feature is the presence of *bona fide* TRE sites, or variation of the sort, as signaled by PscanChIP and shown in Fig 2: the only exception is CREB3, which has YY1 sites as the most significant. Taken together, these data indicate that irrespective of the presence of nearby sites, often with defined distance, single members of a family of TFs have distinct propensity to associate with NF-Y: in turn, this might suggest that domains other than the DBDs either promote co-association or stabilize it.

### Pathway enrichment of co-localizations

**Fig 6:**
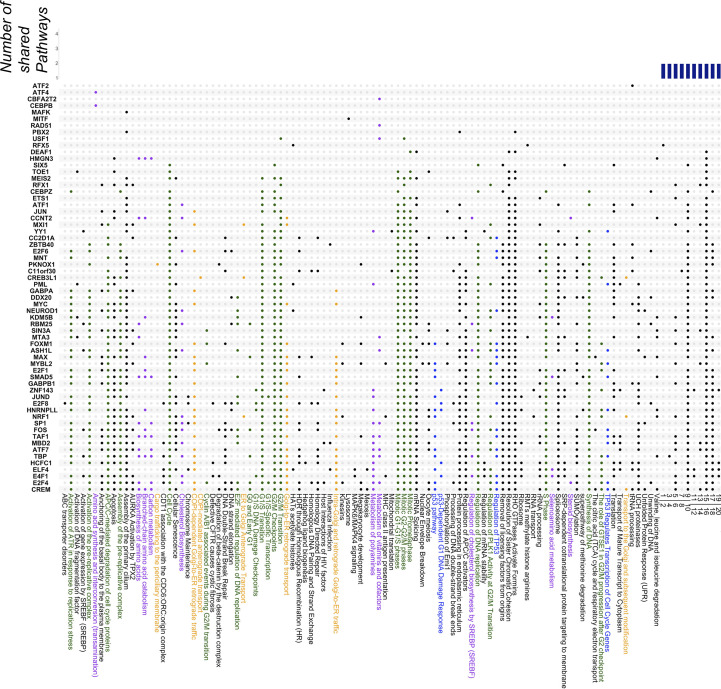
Analysis of co-association and Pathways Enrichment. List of factors with significant overlap and/or CCAAT enrichment in K562, in ascending order based on the number of associated pathways. Blue bars represent the number of shared pathways among different factors, starting from one. Highlighted dots stand for positive intersection between the factor and the individual pathway. In green, cell-cycle related pathways; in purple, metabolism pathways; in blue, p53-related pathways; in yellow, endomembrane-system related pathways.

To get information about which genes are potentially coregulated by groups of TFs with NF-Y, we considered all factors of Tables 1 and 2. Peaks summits in common with NF-YB were annotated with the HOMER tool. We selected genes whose promoter–from -1000 to +100 bp from the TSS–harbors a peak summit; KOBAS 3.0 was run for genes regulated by each NF-Y/TF module to highlight enriched pathways (p-value < 10^−5^ and relative number of background genes <200). Results of K562 cell line are summarized in an UpSet-like plot (Fig 6 and S4 Table): we ranked TFs in rows, according to the increasing number of associated pathways from top to bottom vertically. Also, we show pathways ranked according to the number of factors co-associated with, from top to bottom horizontally. In general, more than half of the factors are associated to >25 pathways, and many of these pathways include more specific terms which can be ascribed to a broad category. E2F1/4/6/8, for example, are collectively associated to most *cell cycle* terms. However, categories are uniquely associated to specific members: E2F8 to *ABC transporter disorder*, *defective CFTR cause Cystic Fibrosis*, *G1/S DNA damage checkpoints*, *p53 DNA-damage response*; E2F1 *translation*, *rRNA processing*; E2F4 to *carbon metabolism* and *CyclinA/B1 associated events during G2/M transition*. In this latter category, E2F4 is with partner factors FOXM1, MYB2L and SIN3A, all subunits of the DREAM complex [54]; in the somewhat related *nuclear envelope breakdown*, we find MYBL2 and FOXM1. This confirms the co-residency of NF-Y with DREAM on this specific class of promoters.

For some TFs, a role in specific pathways was already known: ATF4/CEBPB in amino acids synthesis [81,82]; MITF in lysosome biology [83]. Other associations are novel: RFX5 is found in *HAT acetylation* and *RMT arginine methylation* with PML, ELF4 and NEUROD1; surprisingly, not in *MHC Class II antigen presentation* [84], a category associated instead to MYBL2, NRF1 and E4F1. The related RFX1 is involved in several other, non-overlapping pathways.

Looking at the data vertically, many enriched pathways are–predictably–retrieved full of regulatory proteins co-bound (>20): (i) various terms related to cell-cycle, such as *activation/assembly of the pre-replicative complex*, *G1/S* and *G2/M transition and checkpoints*, *mitosis*/*resolution of sister chromatid cohesion*, *APC-mediated degradation of cell cycle proteins*, *regulation of DNA replication*/*S phase*/*DNA synthesis*, *removal of licensing factors from origin*. (ii) terms related to protein trafficking and processing, *Golgi-to-ER retrograde transport* and *endoplasmic reticulum*. (iii) Terms related to DNA damage response, *activation of ATR in response to replication stress*, *double-strand break repair*, *HDR through homologous recombination*, *p53 pathway*, *regulation of p53*, *p53 regulation of cell cycle genes*, *p53-independent DNA damage response* (pathway 15). (iv) Antigen processing and interferon signaling (pathways 17 and 18). A robust set of genetic and biochemical data support the role of NF-Y in these pathways [5,9,12,13,19,49,85]. Novel pathways with many factors involved are generic terms *translation*, *spliceosome/mRNA splicing*, *UCH proteinases*, *cellular senescence*, *sumoylation* and *apoptosis*. Additional pathways, with fewer factors (10 to 15) are expected from previous work: lipid metabolism (*cholesterol biosynthesis*, *steroid biosynthesis*, *activation of gene expression by SREBP*), *metabolism of amino acids* and *polyamines* [86,87]. A few terms are associated with single or very few factors: *RMTs methylate histone arginines* (RFX5 and MTA3), *ABC transporter disorder* (E2F8), *cargo trafficking to the periciliary membrane* (PKNOX1), *ER to Golgi anterograde transport* (NRF1, CREB3L1, MXI1), *Lysosome* (MITF), *tRNA processing* (ATF2) and *metabolisms of vitamins* (CBF2A2T2/RAD51/USF1).

We then performed an identical analysis on the GM12878 and HeLa-S3 datasets. Although fewer factors are present, the ones globally associated with many pathways are common in the three cell lines (S5A and S5B Fig respectively and extra-pathways in S5 and S6 Tables): General Transcription Factors (TBP and TAF1), cofactors (CHD2 and HCFC1), TFs (SP1 and E2F4). On the other hand, factors associated with more selective pathways, mostly TFs, tend to be more cell type specific.

### Functional analysis of co-localizations

**Fig 7:**
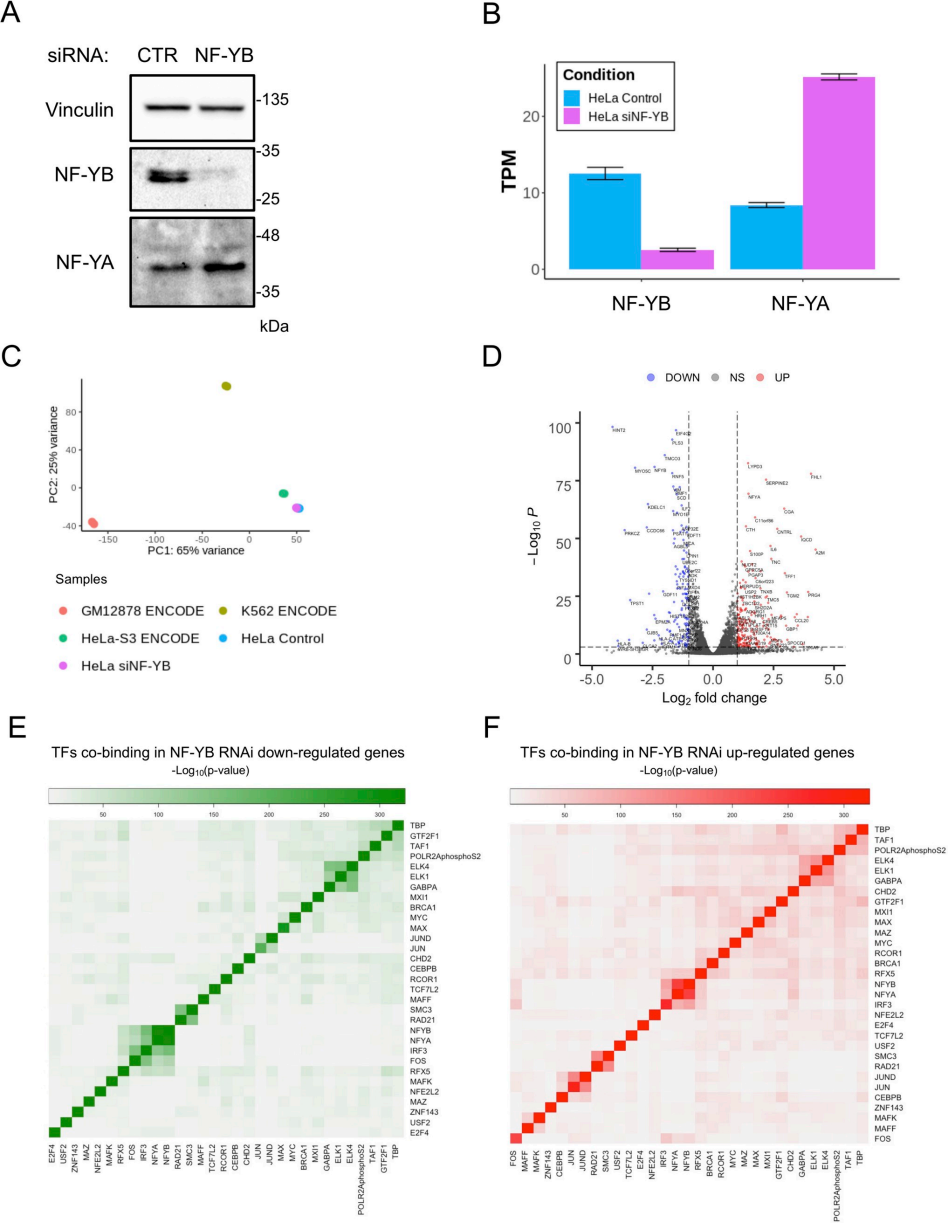
Analysis of mRNA expression data of HeLa cells after NF-YB RNAi A. Western Blot of NF-YA and NF-YB in control (CTR) and NF-YB knockdown HeLa cells. **B**. Barplot of NF-YB and NF-YA mRNA expression from RNA-seq dataset. **C**. Principal Component Analysis (PCA) of ENCODE RNA-seq data together with our HeLa cell line expression data after silencing of NF-YB. **D**. Volcano plot depicting differentially expressed genes of RNA-seq data after siNF-YB in HeLa cell line. **E**. Heatmap of clusters of TFs that bind promoters of down-regulated genes (green scale). **F**. Same as E for up-regulated genes (red scale).

In previous studies, we measured microarrays expression profilings after NF-YA inactivation by shRNA [19,87]. To gain deeper precision, we proceeded with functional characterization of NF-Y targeted genes performing RNA-seq after NF-YB inactivation by siRNA in HeLa cells. The choice of HeLa and NF-YB was due to efficient interference in this cell line. NF-YB inactivation was monitored by Western Blot (Fig 7A). RNA-seq data confirmed the inactivation of NF-YB also at mRNA level (Fig 7B). RNA-seq data for HeLa-S3, GM12878 and K562 are also present in ENCODE: we first verified the adherence of our RNA-seq to the ENCODE datasets by Principal Component Analysis (PCA). Fig 7C shows partitioning of our data with that of ENCODE, while GM12878 and K562 RNA-seq data are clearly distinct. We then retrieved up- and down-regulated genes (FDR < 0.01), which yielded 1622 and 1602 genes, respectively (S7 Table). A Volcano plot representation identifies *bona fide* NF-Y targets among down-regulated genes: HIST1, HLA-A/B/C, RRM2 (Fig 7D). We then analyzed proximal promoters of these genes (-450/+50 from TSS) for enrichment of TFBS–Transcription Factors Binding Sites–using Pscan, a software computing the frequencies of matrices present in the JASPAR database. S6 Fig shows CCAAT among the TFBS enriched in promoters of down-regulated, but not up-regulated genes, confirming that the formers are indeed CCAAT-dependent, whereas activation upon NF-YB removal is mediated by other matrices (and promoters-bound TFs). We monitored the mRNA levels of all TFs and cofactors present in Tables 1 and 2 from HeLa-S3 cells: the most down-regulated is NF-YB, and the most up-regulated NF-YA, both expected from previous data [88]. Most factors have relatively modest changes–< 0.5-fold–which suggests that effects are minimally due to secondary changes in the levels of such TFs (S7 Fig). In summary, we derived a robust set of genes with CCAAT boxes in promoters, whose expression depends upon NF-Y, and another CCAAT-less set which is increased upon NF-Y elimination, presumably in an indirect way.

Having shown no dramatic changes in the levels of most co-bound factors, we calculated the presence of each of them in up- and down-regulated genes: we expect that those selectively implicated in coregulation of NF-Y-dependent genes to be over-represented in cohorts of down-regulated genes. Vice-versa, the factors with which NF-Y mediates a negative effect will be skewed toward repression (that is, up-regulation upon NF-YB interference). We performed pairwise analysis and then gathered all data in heatmaps to give a graphical representation of groups of factors involved in a collective effort of repression or activation. Fig 7E and 7F show that for the most, TFs and cofactors are equally distributed, suggesting no specific cooperativity in one way or the other. On the other hand, a few factors segregate differently in NF-Y activated (Fig 7E) from repressed (Fig 7F): GTF2F1 moves away from GTFs–RNA Pol II, TAF1 and TBP–C/EBPB away from JUN/JUND, MAFF and MAFK move FOS away from the NF-Y sub-cluster with IRF3 and RFX5, into a repressive cluster. This suggests a positive functional role in promoting NF-Y-mediated expression of genes.

### NF-Y and chromatin states

ENCODE analyzed several chromatin features of Tier 1 cell lines, such as DNase I hypersensitive sites, DNA methylation, several histone PTMs, and also characterized the respective transcriptomes by RNA-seq. Histone PTMs were further processed to defined chromatin “states”, both by ENCODE and the RoadMap consortium. The latter defined 18 distinct chromatin states [32]. This annotation covers the whole genome segmented into non overlapping regions of 200 bp. Each region is assigned to a specific chromatin state, according to a model that takes into account the combination (presence/absence) of six marks. Major functional features are Active TSS (Promoters, in different tones of Red), Enhancers (Orange), and inactive/heterochromatin (Grey). We used this classification to analyze the sites of each protein present in Table 1 and Table 2.

**Fig 8:**
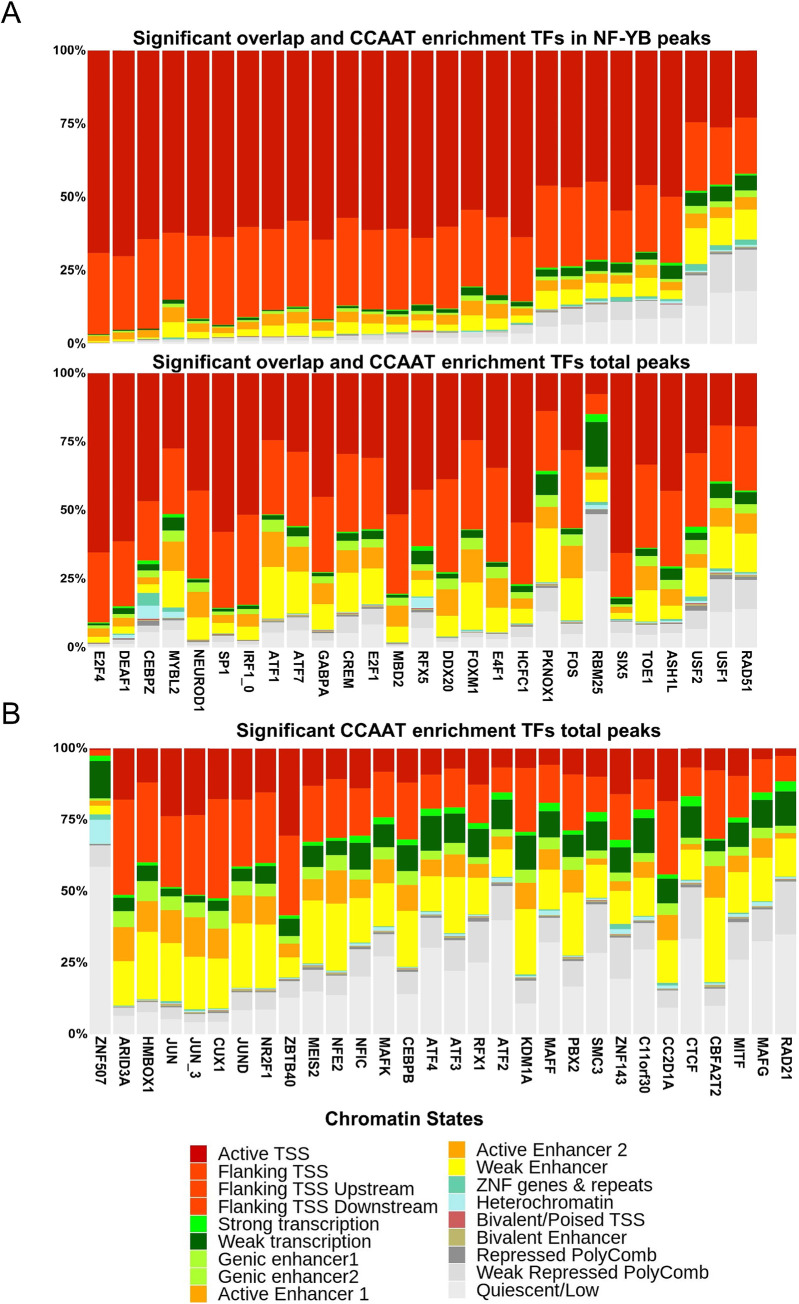
Chromatin states in locations of NF-Y co-associated factors. Relative distribution of chromatin states across ChIP-seq peak regions of the individual factor in K562 cell line. **A**. Upper panel: plot includes factors having significant overlap with NF-YB and significant CCAAT enrichment; the regions of the factor co-bound with NF-YB are included. Lower panel: same as upper panel, except that all peaks of the factors are computed. **B**. Same as A, except that factors with significant CCAAT enrichment, but not peaks overlap, are included.

Results were split according to two major categories of co-association with NF-Y: only CCAAT enrichment, or both CCAAT enrichment and significant overlap. For the last category, we evaluated additional chromatin states of peaks overlapping with NF-YB. The data shown in Fig 8A are those for factors co-localizing with NF-Y in K562 cells. On average, TFs peaks in common with NF-YB exhibited a majority of Active marks on TSS or flanking areas (Upper Panel); the comparison with the total peaks locations of the same factor (Lower Panel) indicates that several factors–MYBL2, ATF1/7, CREM, E2F1, PKNOX1– are shifted toward active promoter areas in NF-YB peaks. The RNA-binding protein RMB25 is mostly–>50%–associated to Polycomb and Quiescent/Low locations in total peaks, and predominantly– 75%–to active promoters in NF-YB peaks. This is somewhat similar for ASH1L, USF1 and USF2 in total peaks, but these factors maintain the configuration of Quiescent/Low and Weak Repressed Polycomb states in regions shared with NF-YB. For USF1, the results are consistent with previous identification of a significant portion of NF-YB-shared binding sites in repressed HERV/LTR regions [12,16,19,89]. For the factors having CCAAT enrichment but no significant overlap of peaks (Fig 8B), there is a clear skewing–around 50% of sites–toward regions with inactive chromatin marks, particularly for the Cohesin subunits RAD21/SMC/CTCF, B-Zips (ATFs, MAFs), RFX1, NFIC and ZNF507. These factors are apparently avoiding active promoters and mostly located on distal locations, often with inactive chromatin configurations. Of note, the apparently repressive KDM1, a H3K4 demethylase, is mostly–>75%–associated to active locations, either in promoters or enhancers. The results of GM12878 and HeLa-S3 are shown in S8 and S9 Figs, respectively. The similar behaviour of Cohesin and USF1/2 in both lines and of B-Zips, in HeLa-S3, reinforces the results of our analysis.

## Conclusions

The ever-growing emporium of data generated by the ENCODE Project invited an update on NF-Y “friends” on the genomes of three cancer cell lines. We confirmed the 2014 data, doubled the number of TFs and cofactors associated to NF-Y in a significant number of sites, and modified our previous classification, adding a novel Group. Many classes of TFs are represented, often with single members prevailing. Importantly, new classes of proteins enter the NF-Y circle, such as RNA-binding proteins–notably involved in alternative splicing–and subunits of repressive complexes (NuRD, DREAM). Functional experiments and analysis of chromatin features help partition specific factors and target genes categorization. The conclusions reached here represent the basis for prioritization of biochemical dissections of NF-Y/TFs interactions, through modelling of available 3D structures, as well as reconstruction *in vitro*–and possibly visualization by Cryo-EM–of higher order complexes. As ENCODE further expands with more physiological approaches, such as CRISPR-Cas9-mediated inactivations, we look forward to eventually reconstruct NF-Y interactions with all factors on all loci, *via* the pipeline devised here.

## Supporting information

S1 Table P-values associated to positional bias output by PscanChIP run on peaks summit of TF ChIP-seq experiment. TFs scored as positive, i.e. whose p-value<10^−10^, in at least one cell line are reported.
(XLSX)

S2 Table PscanChIP second run results. Factors belonging to Table 2 undergone to a second run of PscanChIP restricted to co-binding region with NF-YB. White/yellow background entries refers to first run, whereas white/black refers to second run results.
(XLSX)

S3 Table Pairwise overlap results restricted to NF-YB peak regions. (XLSX)

S4 Table Co-occurred enriched pathways of Fig 6 legend (K562 cell line). (XLSX)

S5 Table Co-occurred enriched pathways of S5A Fig legend (GM12878 cell line). (XLSX)

S6 Table Co-occurred enriched pathways of S5B Fig legend (HeLa-S3 cell line). (XLSX)

S7 Table Differentially expressed genes list. (XLSX)

S8 Table Enriched TFBS motif in promoter of up-regulated genes. Pscan output.
(XLSX)

S9 Table Enriched TFBS motif in promoter of down-regulated genes. Pscan output.
(XLSX)

S1 Fig Analysis of positional bias between the CCAAT matrix and all available TFs matrix in JASPAR 2020 Redundant database version. (PDF)

S2 Fig Heatmaps of pairwise co-association score of K562 cell line factors present in Table I or II. A. Genome-wide representation. B. Analysis restricted to NF-YB-bound regions.
(PDF)

S3 Fig Heatmaps of pairwise co-association score of all GM12878. A. Genome-wide representation. B. Analysis restricted to NF-YB-bound regions.
(PDF)

S4 Fig Heatmaps of pairwise co-association score of all HeLa-S3. A. Genome-wide representation. B. Analysis restricted to NF-YB-bound regions.
(PDF)

S5 Fig Analysis of co-association and Pathways Enrichment in GM12878 and HeLa-S3 cell lines. List of factors with significant overlap and/or CCAAT enrichment in A. GM12878 and B. HeLa-S3 cell lines, in ascending order based on the number of associated pathways. Blue bars represent the number of shared pathways among different factors, starting from one. Highlighted dots stand for positive intersection between the factor and the individual pathway. In green, cell-cycle related pathways; in purple, metabolism pathways; in blue, p53-related pathways; in yellow, endomembrane-system related pathways.
(PDF)

S6 Fig Pscan analysis. Output of Pscan on promoters of up- (left) and down- (right) regulated genes after NF-YB inactivation, showing most enriched TFBS.
(PDF)

S7 Fig Differential expression of genes for which corresponding TFs have ENCODE ChIP-seq experiment and belong to NF-Y partners. (PDF)

S8 Fig Chromatin states in GM12878 cell line. Relative distribution of chromatin states across factors ChIP-seq peak regions. A-B plots include factors with significant overlap with NF-YB and significant CCAAT enrichment. A. distribution of regions co-bound with NF-YB; B. distribution of all regions of factor. C. Distribution of regions of factors with significant CCAAT enrichment but not overlap.
(PDF)

S9 Fig Chromatin states in HeLa-S3 cell line. Relative distribution of chromatin states across factors ChIP-seq peak regions. A-B plots include factors with significant overlap with NF-YB and significant CCAAT enrichment. A. distribution of regions co-bound with NF-YB; B. distribution of all regions of factor. C. Distribution of regions of factors with significant CCAAT enrichment but not significant overlap.
(PDF)
