# Supplementary material for: On the NF-Y regulome as in ENCODE (2019)
Source: PLoS Comput Biol. 2020 Dec 28;16(12):e1008488. doi: 10.1371/journal.pcbi.1008488 (PMC7793273; doi:10.1371/journal.pcbi.1008488)

ATF4 in K562 cell line
MA0833.1 ATF4

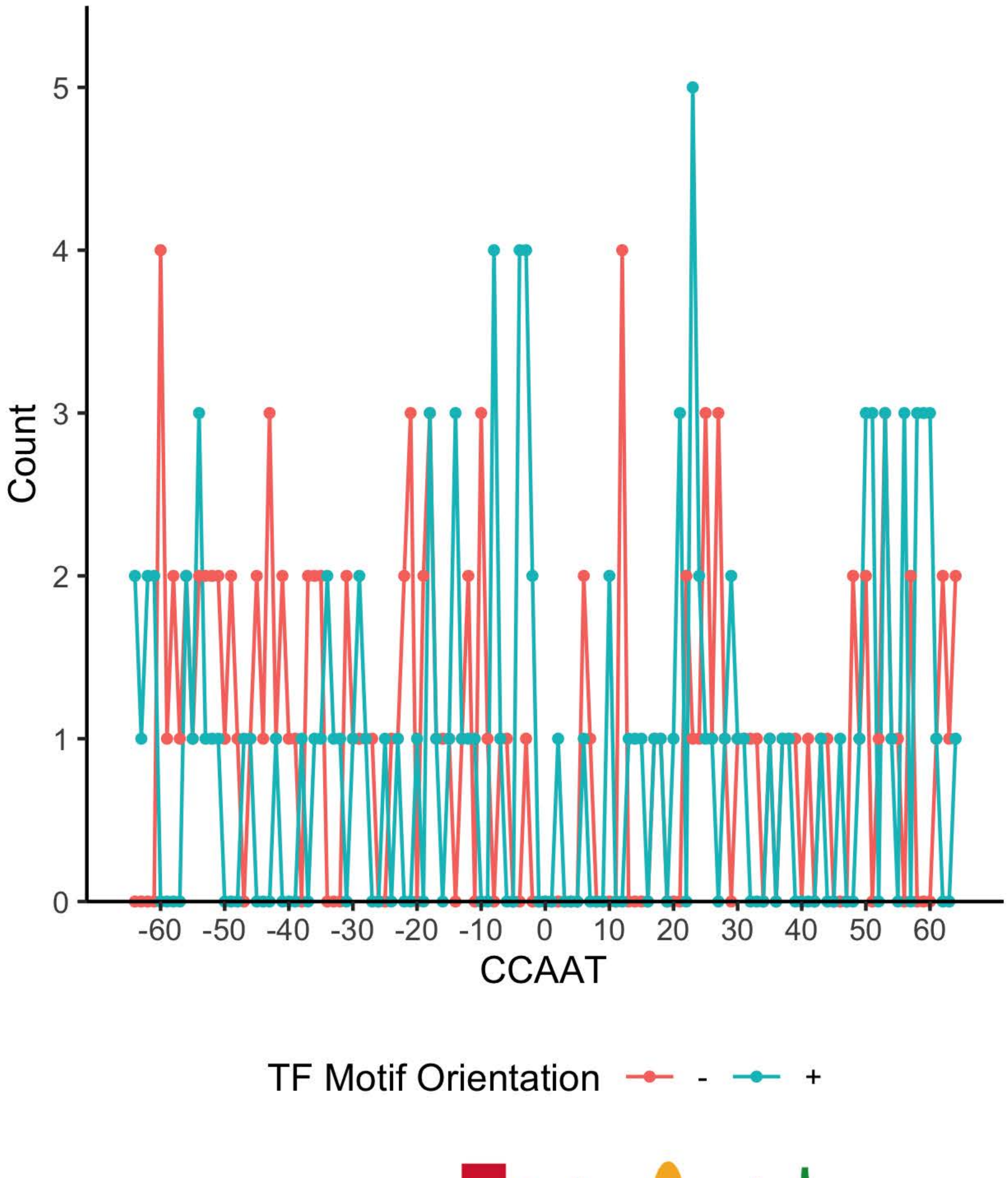

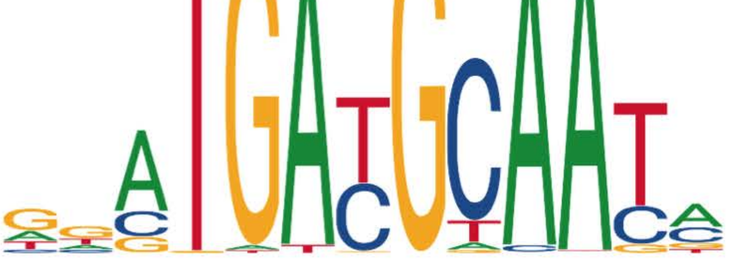

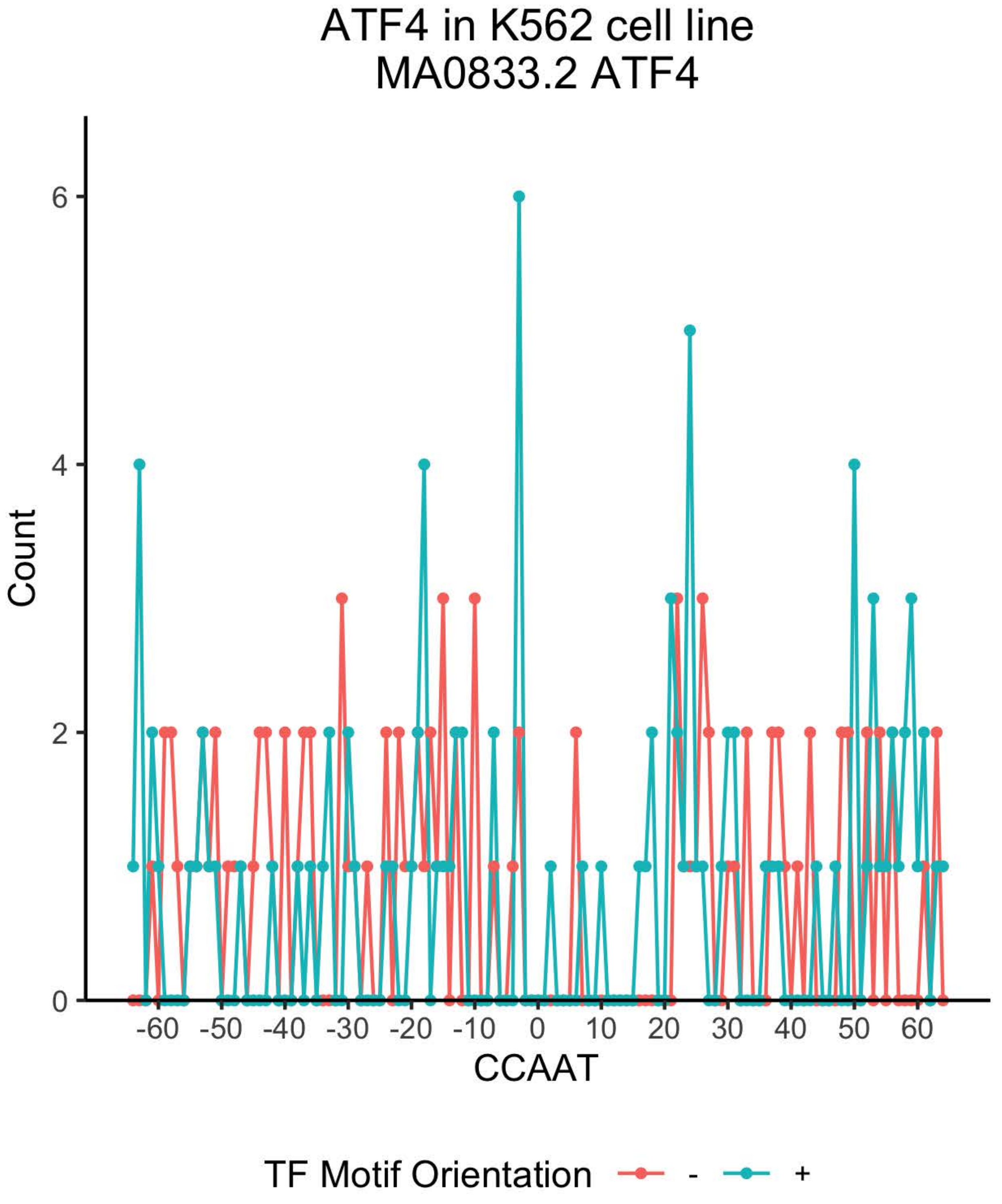
ATF4 in K562 cell line
MA0833.2 ATF4
Count
CCAAT
TF Motif Orientation
-
+

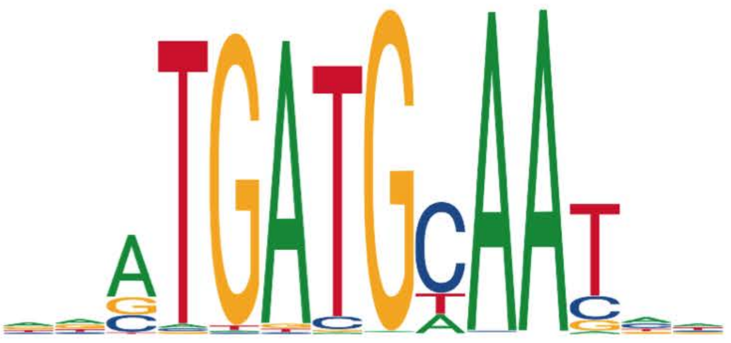

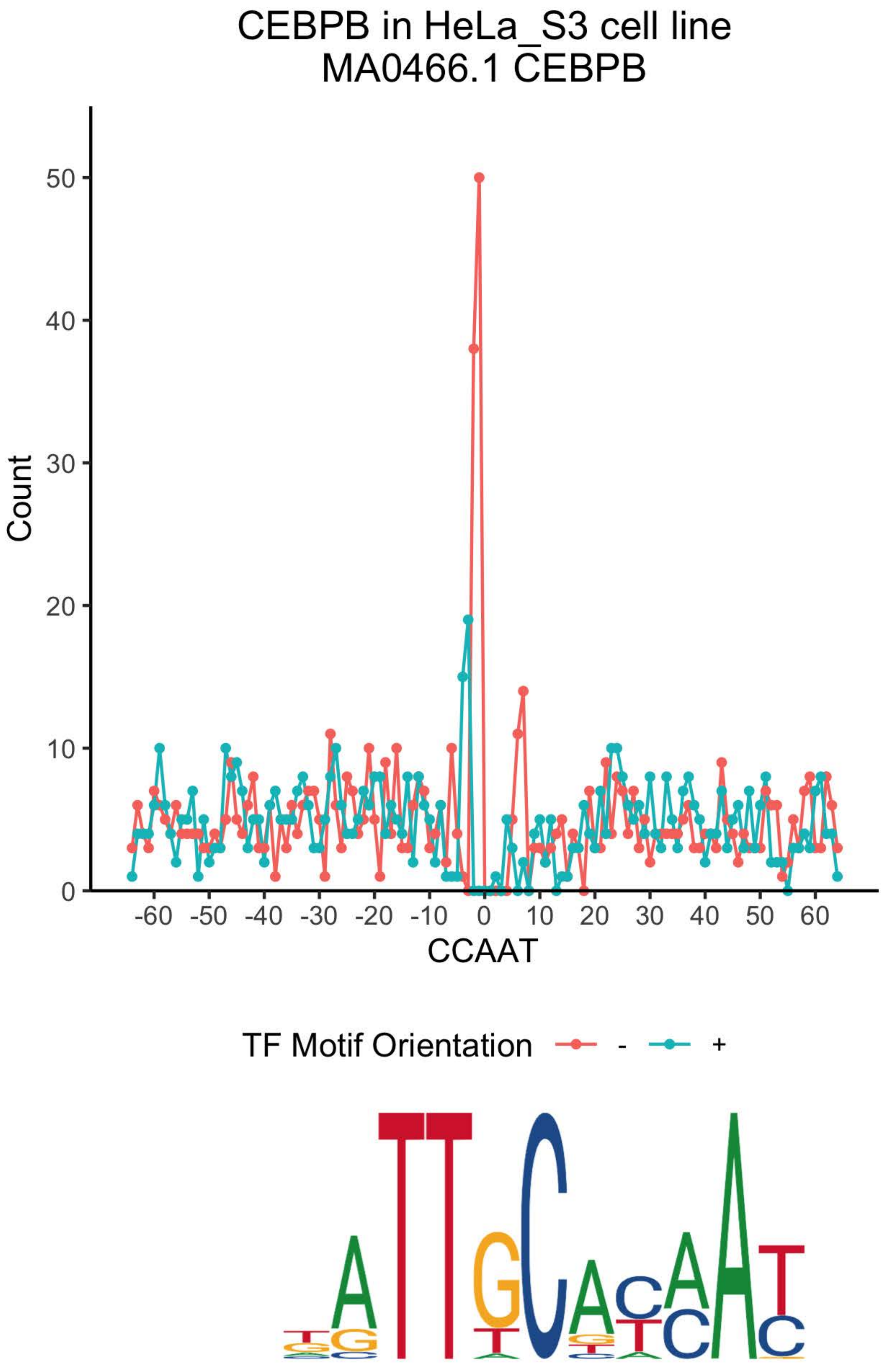

CEBPB in HeLa_S3 cell line
MA0466.1 CEBPB
Count
50
40
30
20
10
0
-60 -50 -40 -30 -20 -10 0 10 20 30 40 50 60
CCAAT
TF Motif Orientation - +

CEBPB in HeLa_S3 cell line
MA0466.2 CEBPB

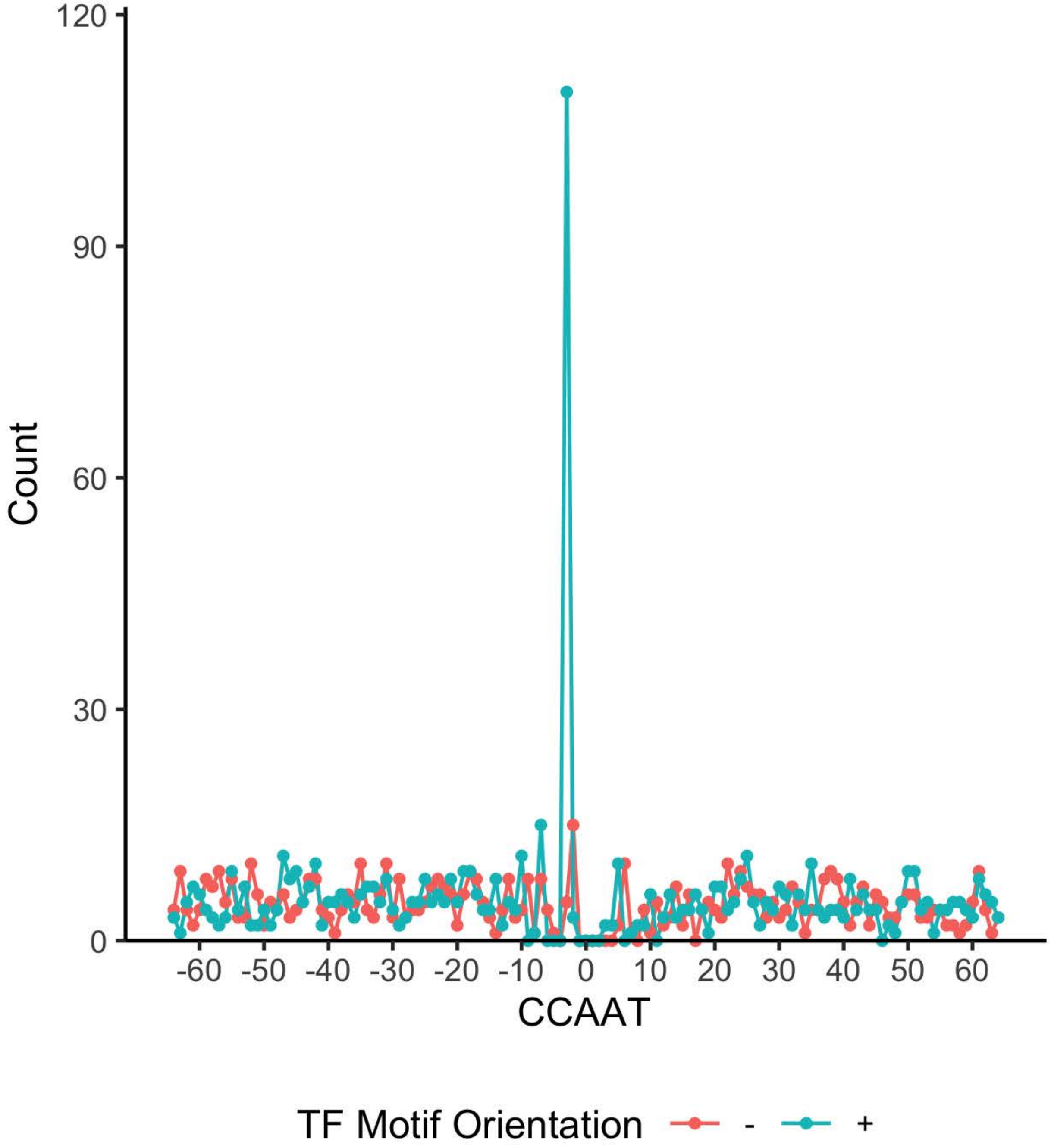

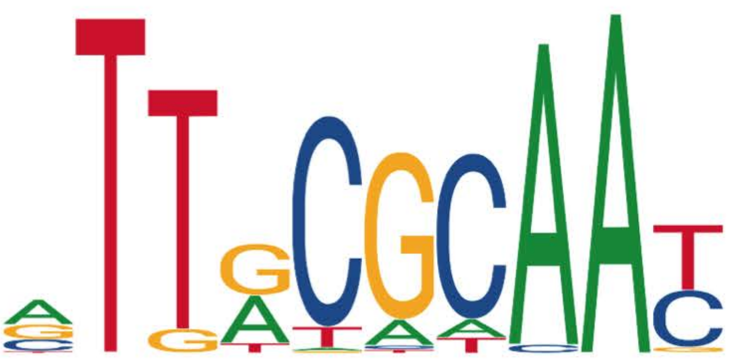

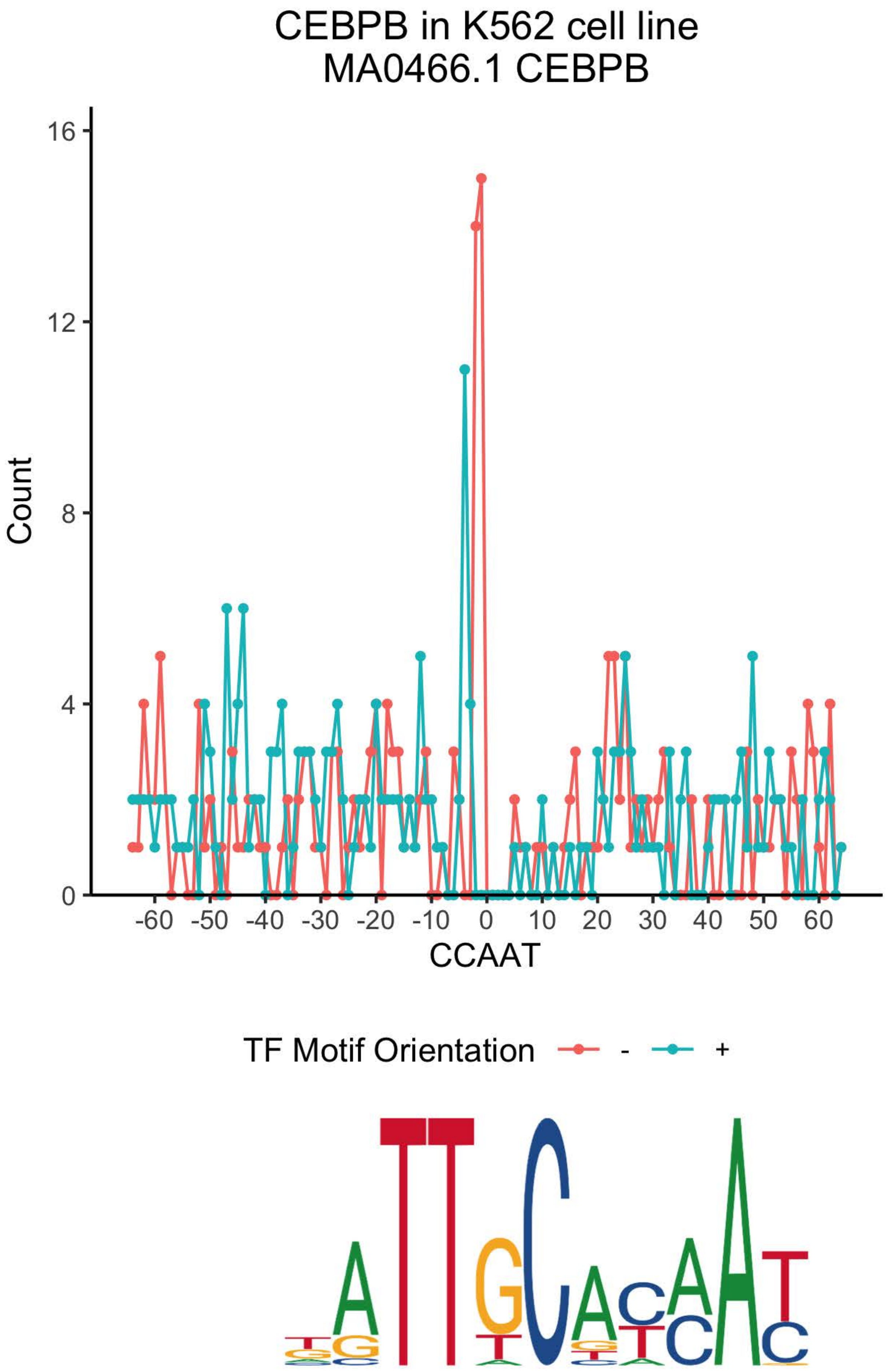
CEBPB in K562 cell line
MA0466.1 CEBPB
Count
16
12
8
4
0
-60 -50 -40 -30 -20 -10 0 10 20 30 40 50 60
CCAAT
TF Motif Orientation
-
+

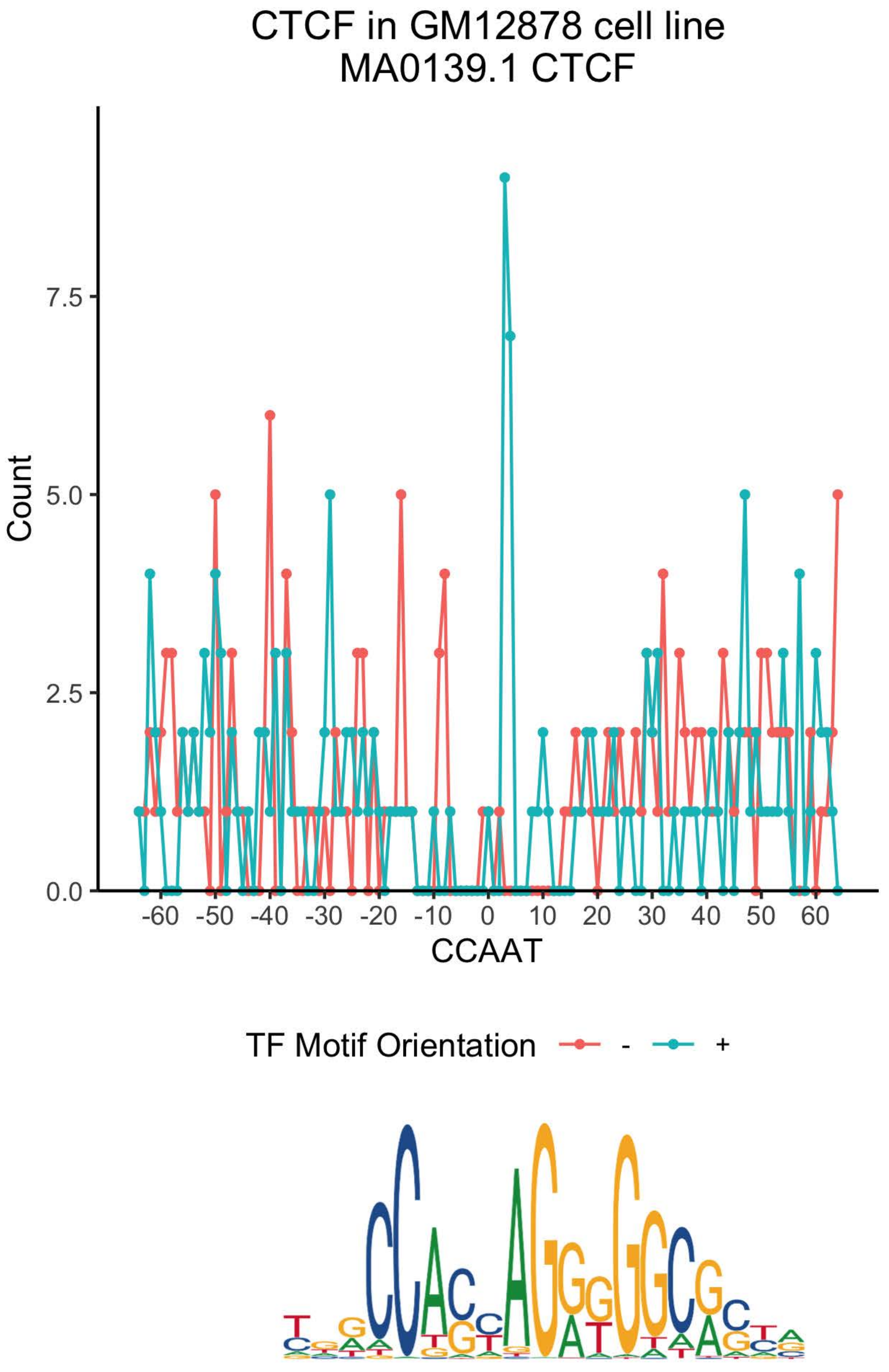
CTCF in GM12878 cell line
MA0139.1 CTCF
Count
7.5
5.0
2.5
0.0
-60
-50
-40
-30
-20
-10
0
10
20
30
40
50
60
CCAAT
TF Motif Orientation
-
+

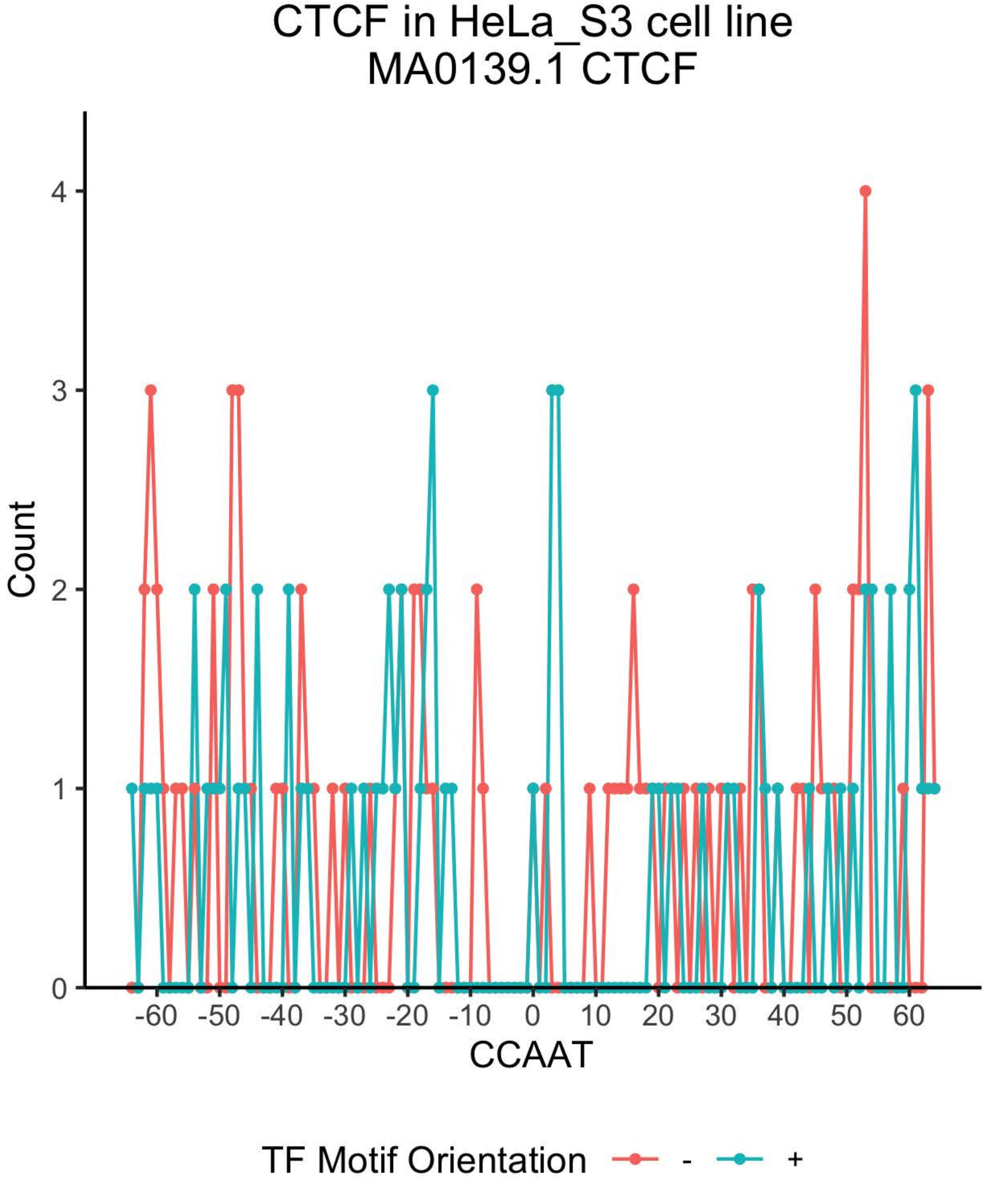

CTCF in HeLa_S3 cell line
MA0139.1 CTCF
Count
CCAAT
0
1
2
3
4
-60
-50
-40
-30
-20
-10
0
10
20
30
40
50
60
TF Motif Orientation
-
+

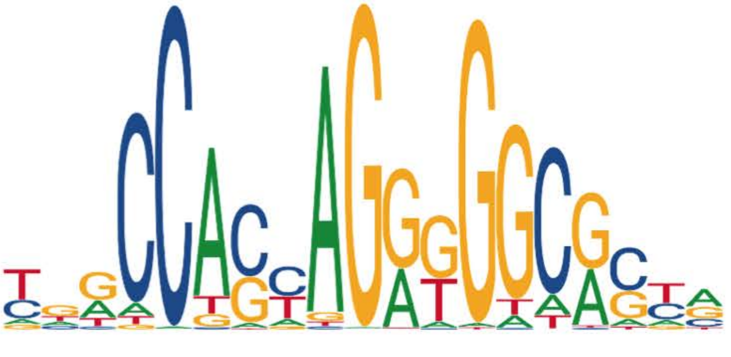

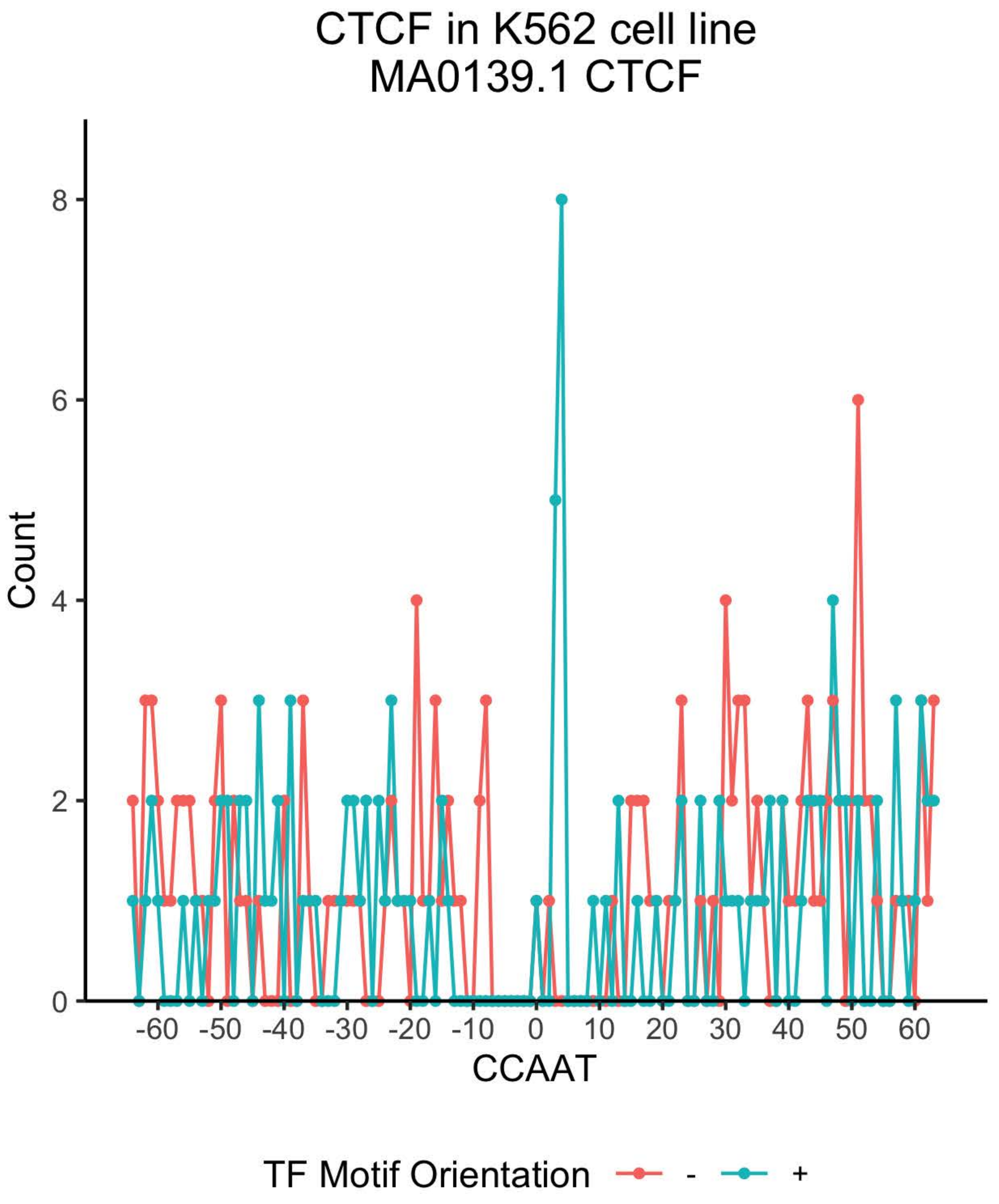
CTCF in K562 cell line
MA0139.1 CTCF
Count
0
2
4
6
8
-60 -50 -40 -30 -20 -10 0 10 20 30 40 50 60
CCAAT
TF Motif Orientation
-
+

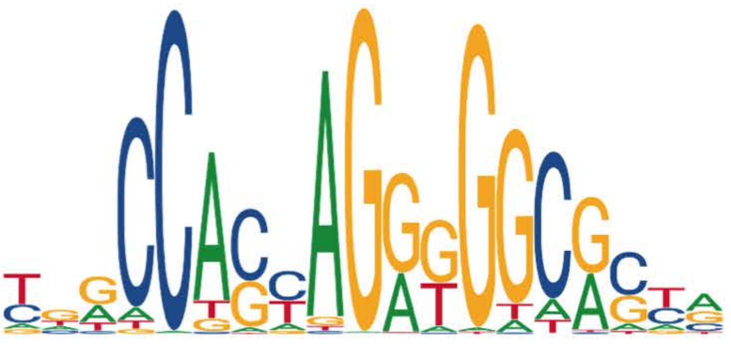

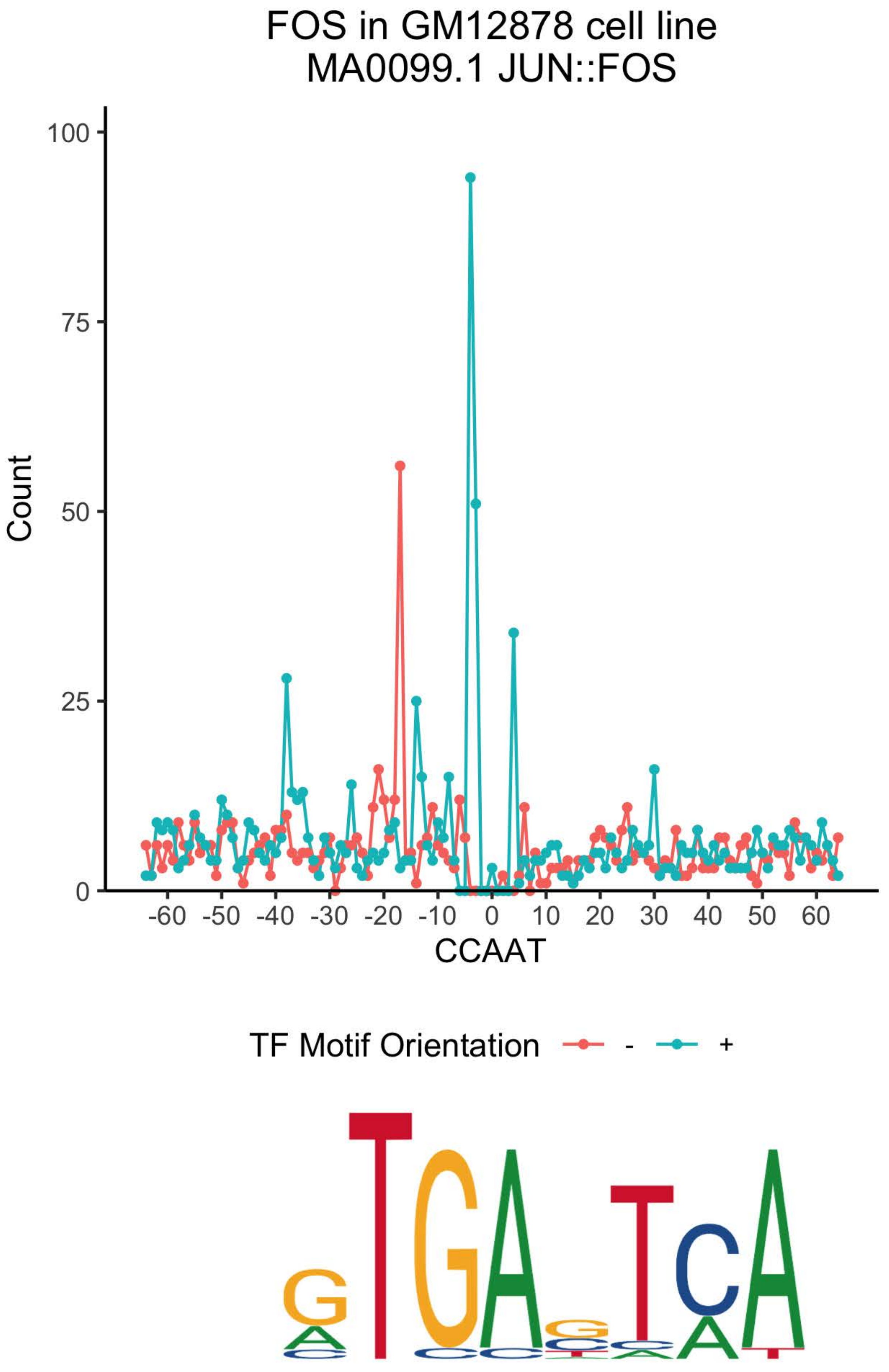

FOS in GM12878 cell line
MA0099.1 JUN::FOS
Count
100
75
50
25
0
-60 -50 -40 -30 -20 -10 0 10 20 30 40 50 60
CCAAT
TF Motif Orientation
-
+

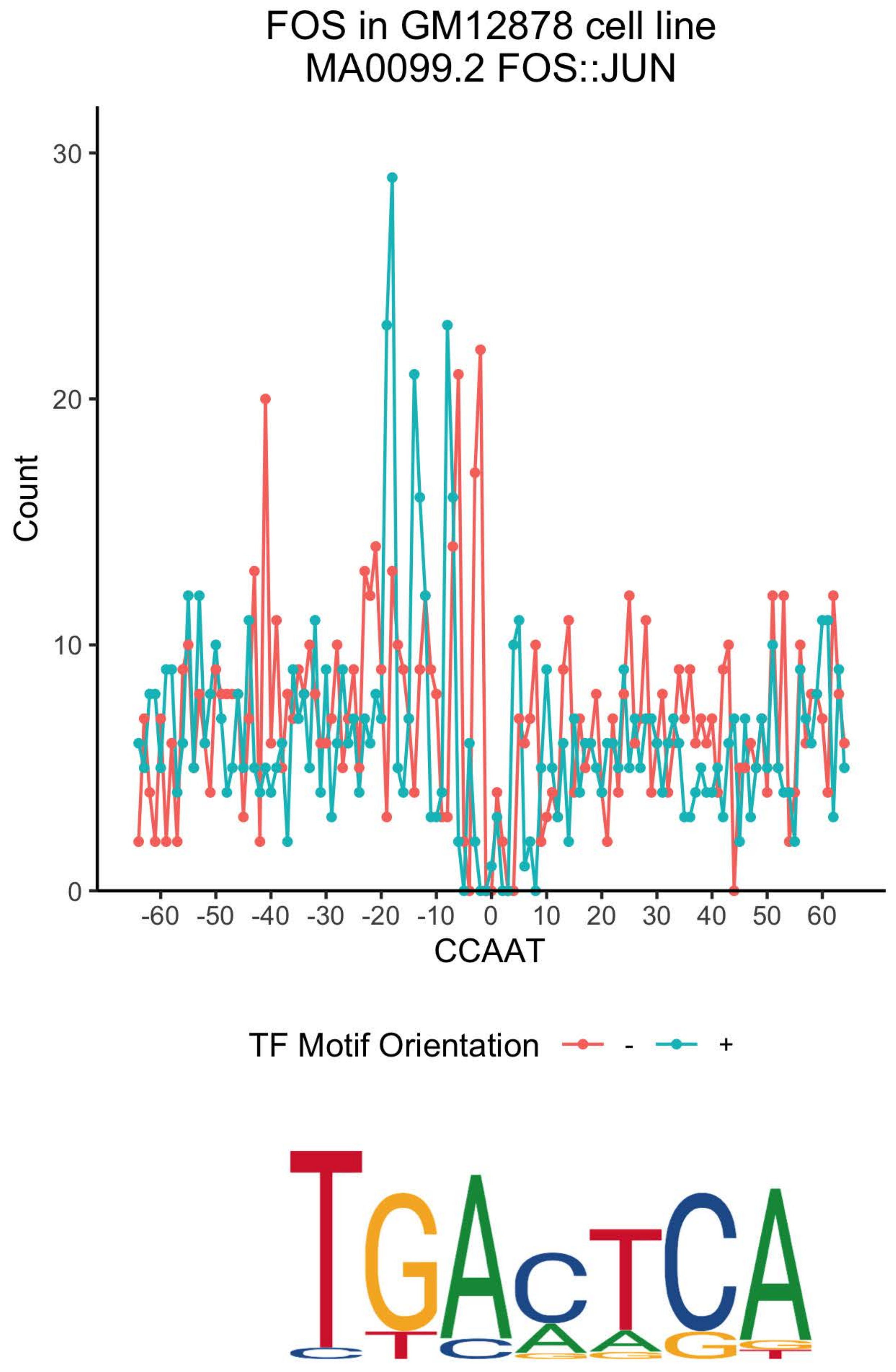
FOS in GM12878 cell line
MA0099.2 FOS::JUN
Count
0
10
20
30
-60 -50 -40 -30 -20 -10 0 10 20 30 40 50 60
CCAAT
TF Motif Orientation
-
+

FOS in GM12878 cell line
MA0099.3 FOS::JUN

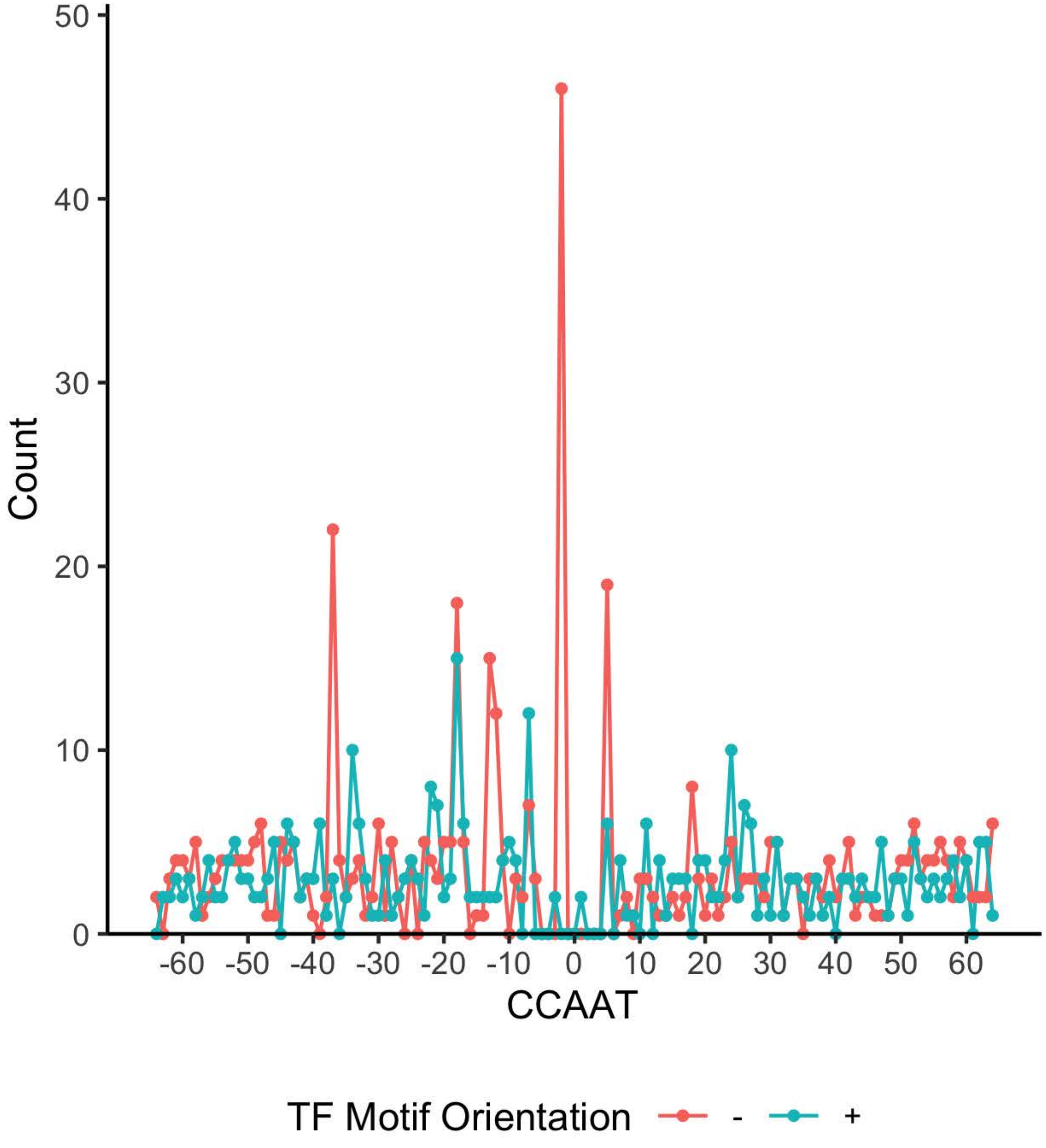

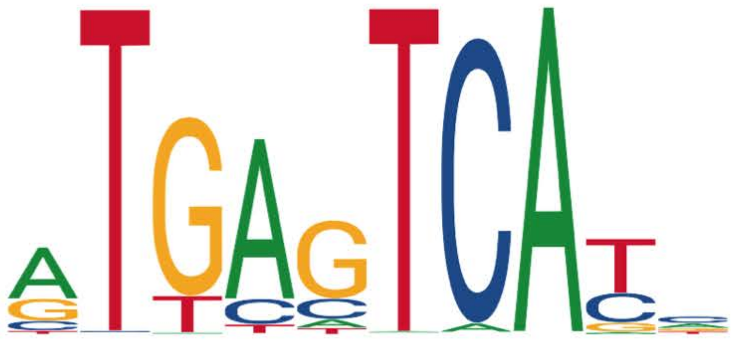

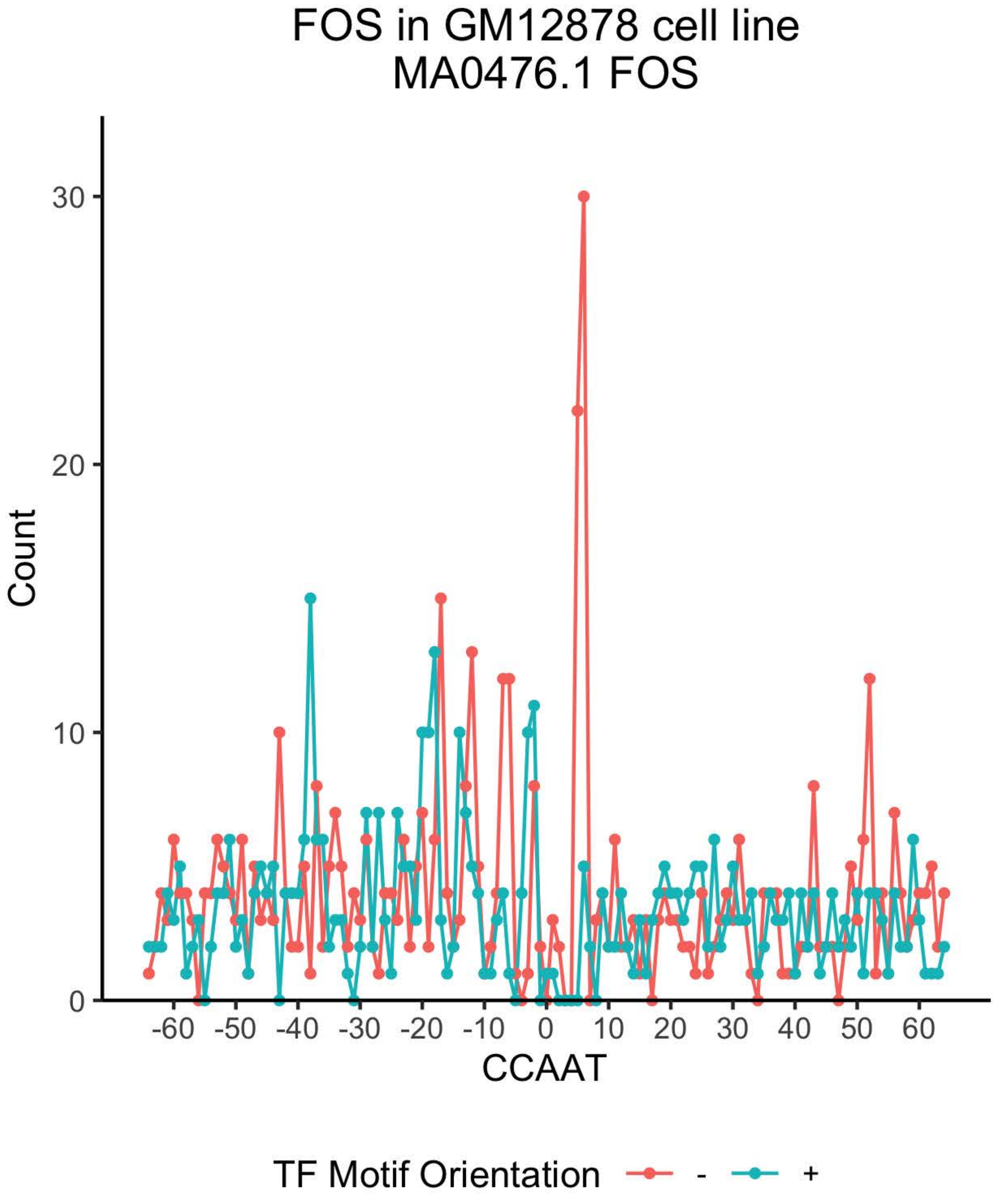
FOS in GM12878 cell line
MA0476.1 FOS
Count
30
20
10
0
-60
-50
-40
-30
-20
-10
0
10
20
30
40
50
60
CCAAT
TF Motif Orientation
-
+

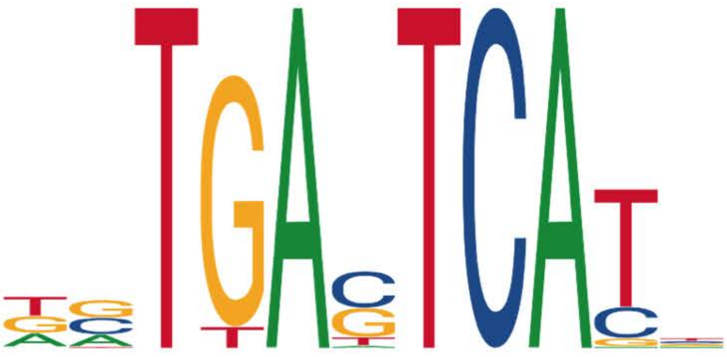

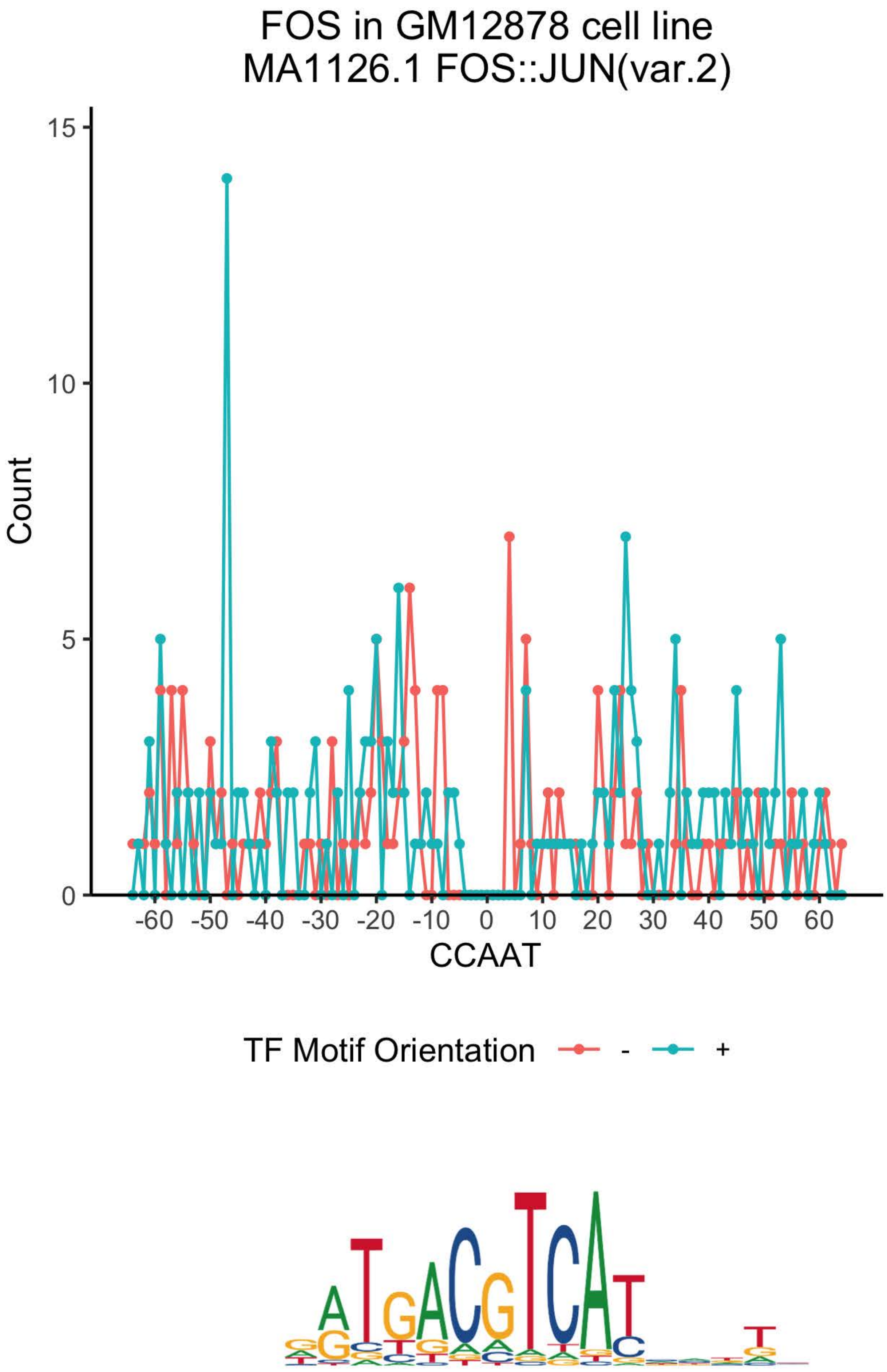
FOS in GM12878 cell line
MA1126.1 FOS::JUN(var.2)
Count
15
10
5
0
-60 -50 -40 -30 -20 -10 0 10 20 30 40 50 60
CCAAT
TF Motif Orientation
-
+

FOS in GM12878 cell line
MA1134.1 FOS::JUNB

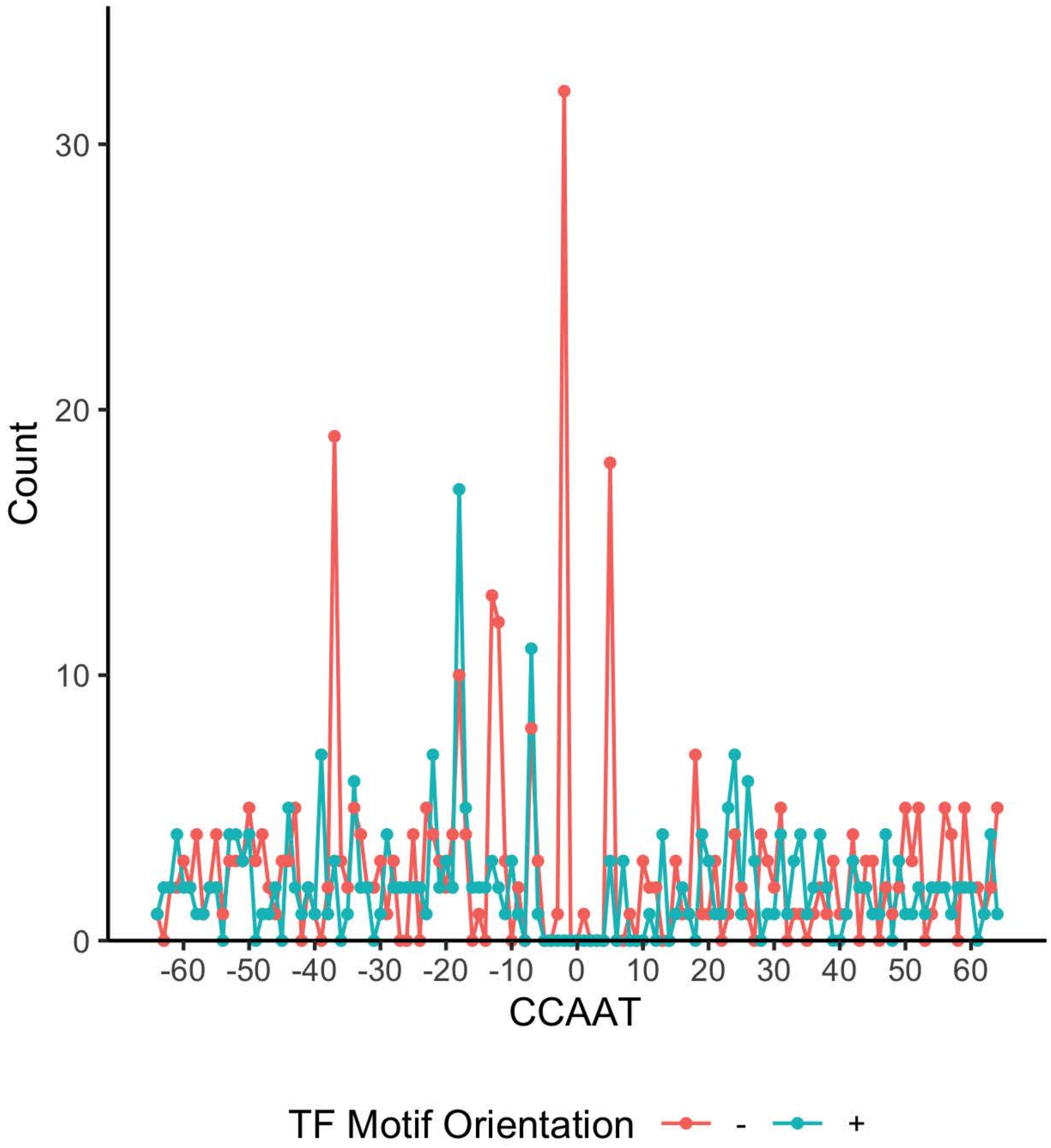

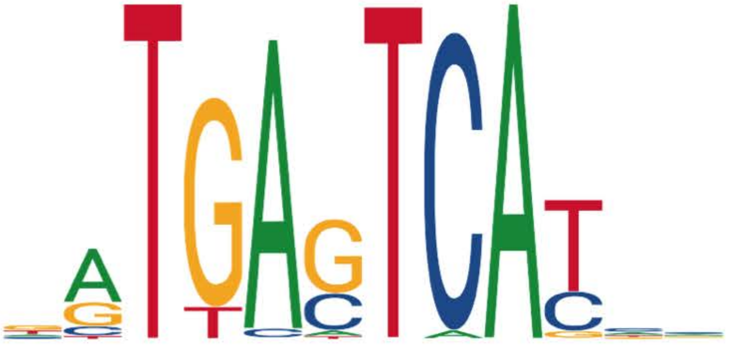

FOS in GM12878 cell line
MA1141.1 FOS::JUND

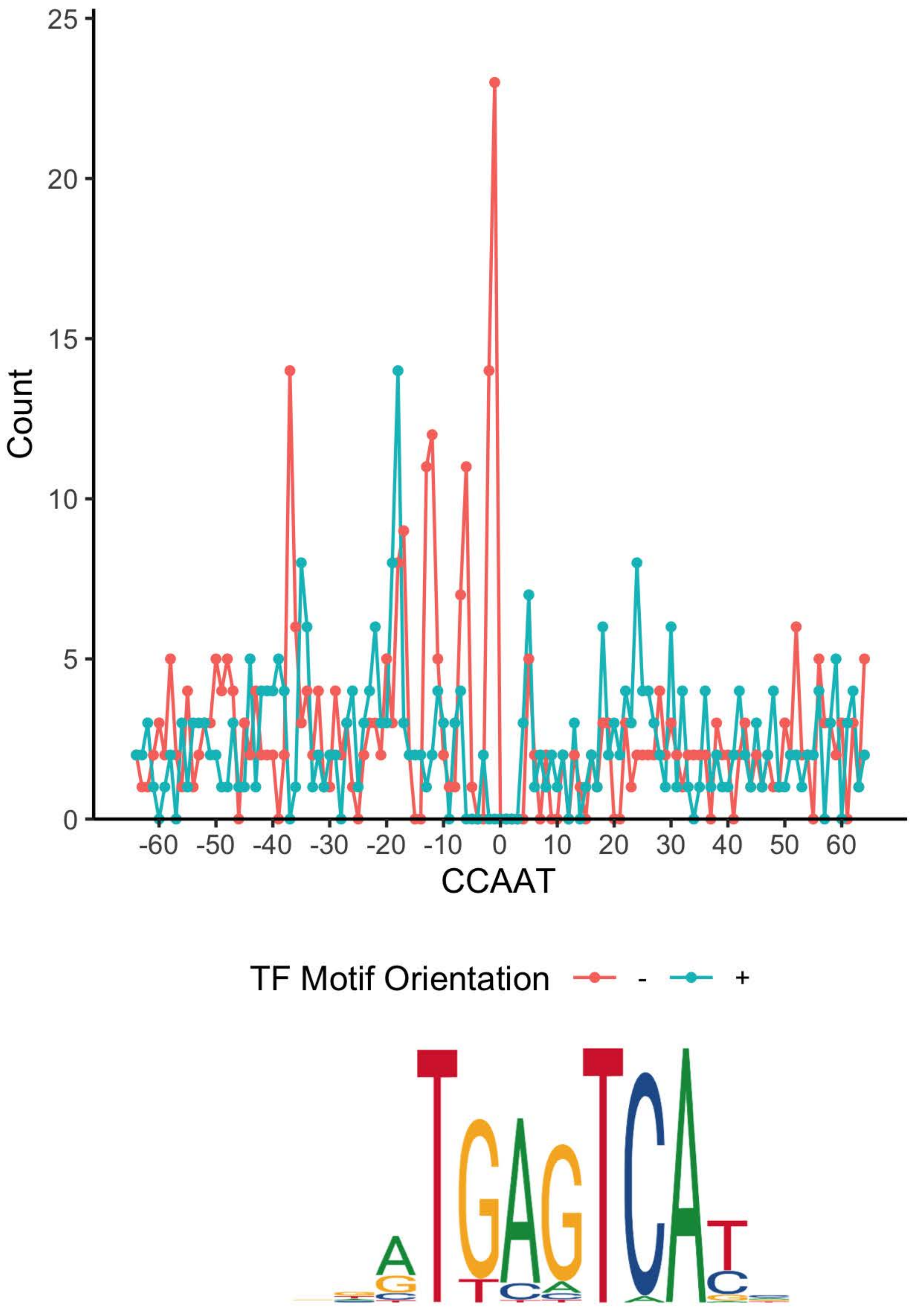

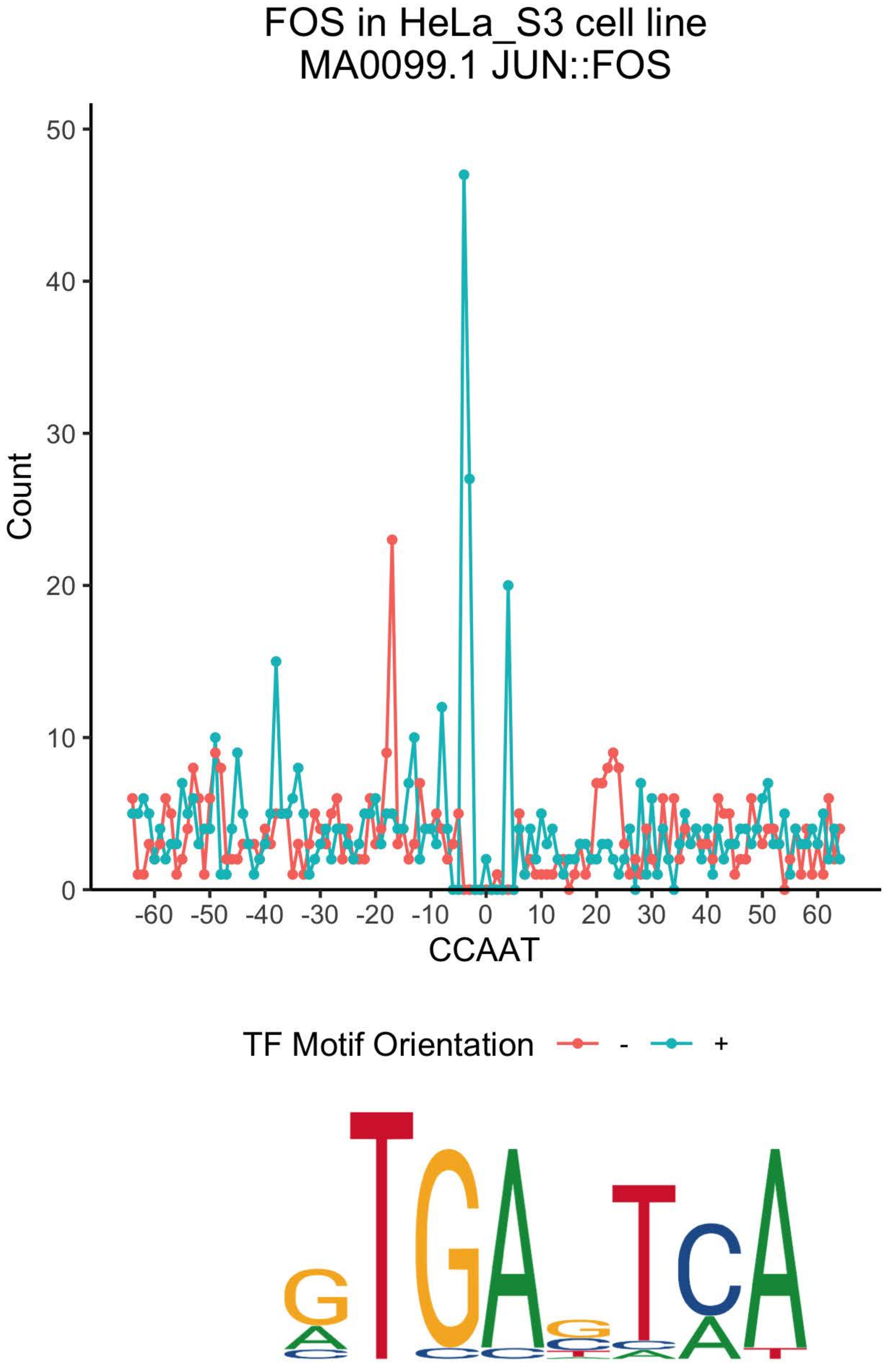
FOS in HeLa_S3 cell line
MA0099.1 JUN::FOS
Count
0
10
20
30
40
50
-60
-50
-40
-30
-20
-10
0
10
20
30
40
50
60
CCAAT
TF Motif Orientation
-
+

FOS in HeLa_S3 cell line
MA0099.2 FOS::JUN

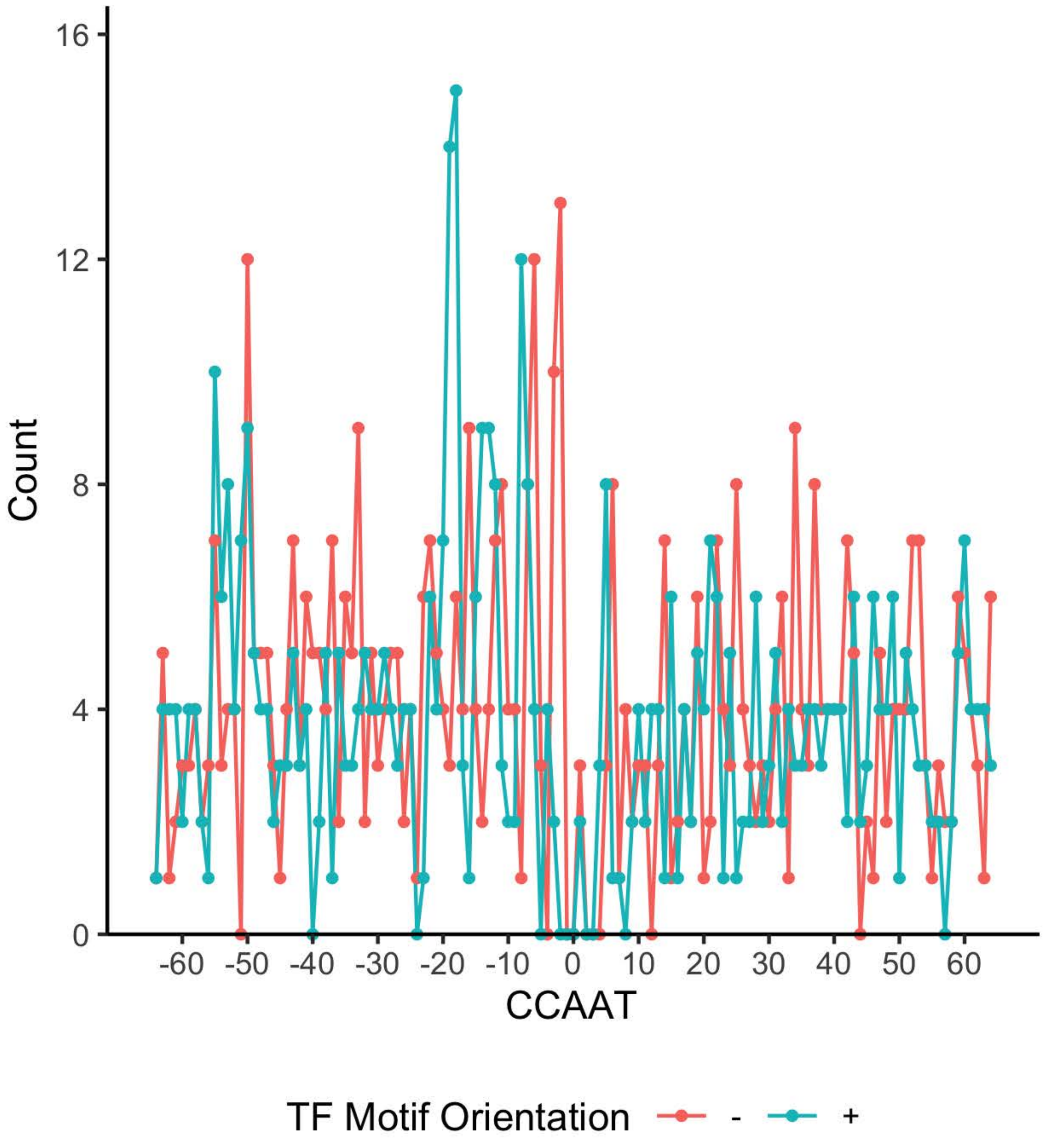

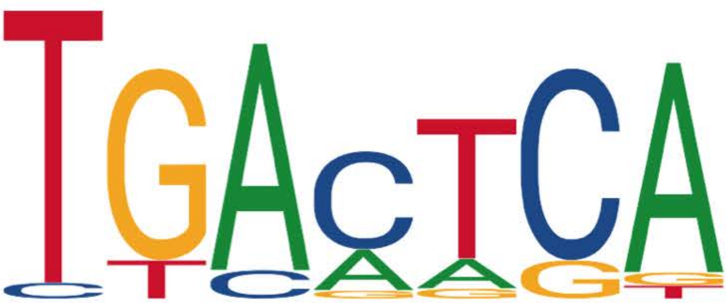

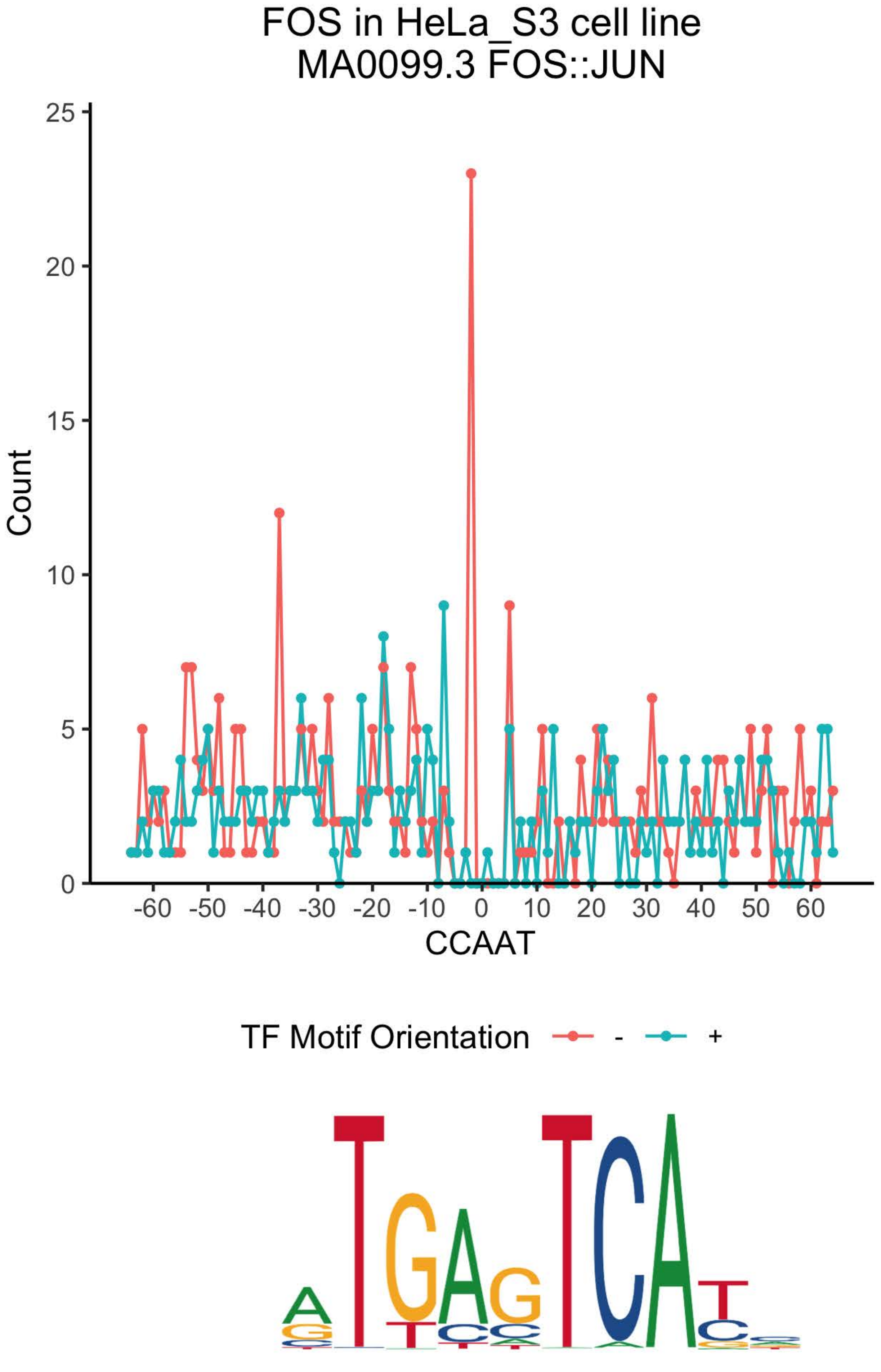
FOS in HeLa_S3 cell line
MA0099.3 FOS::JUN
Count
CCAAT
0
5
10
15
20
25
-60
-50
-40
-30
-20
-10
0
10
20
30
40
50
60
TF Motif Orientation
-
+

FOS in HeLa_S3 cell line
MA0476.1 FOS

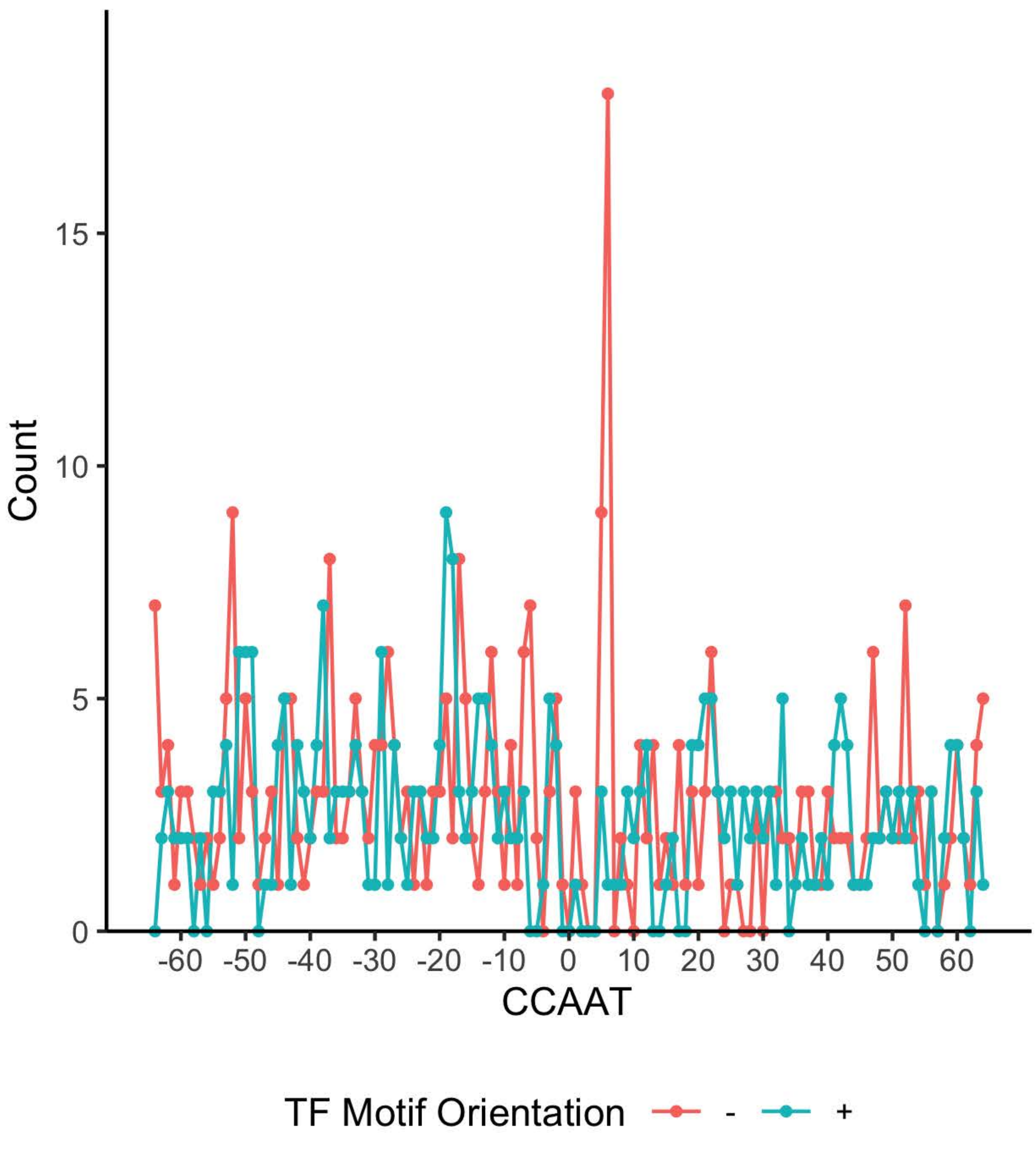

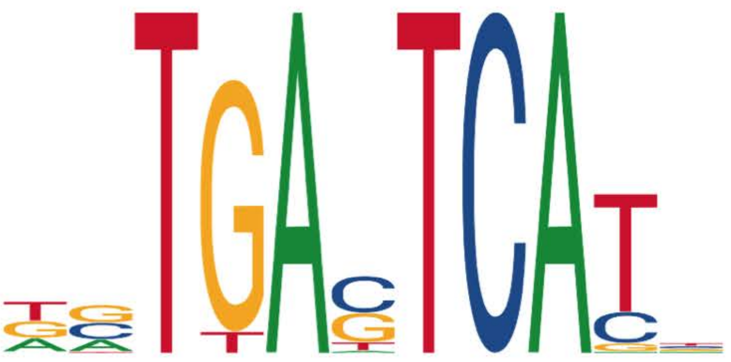

FOS in HeLa_S3 cell line
MA1126.1 FOS::JUN(var.2)

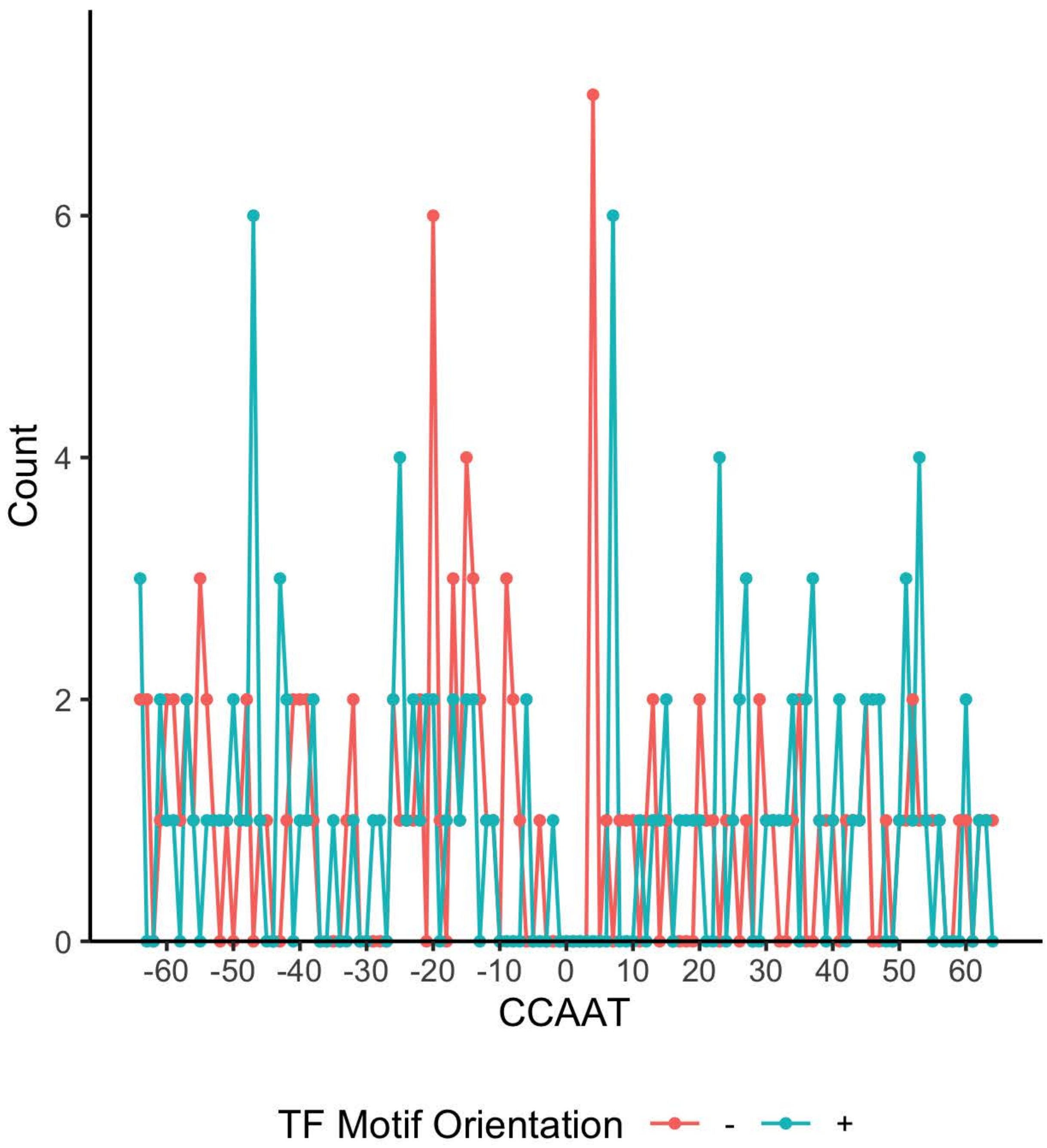

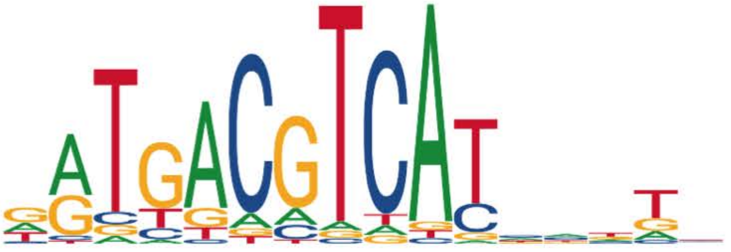

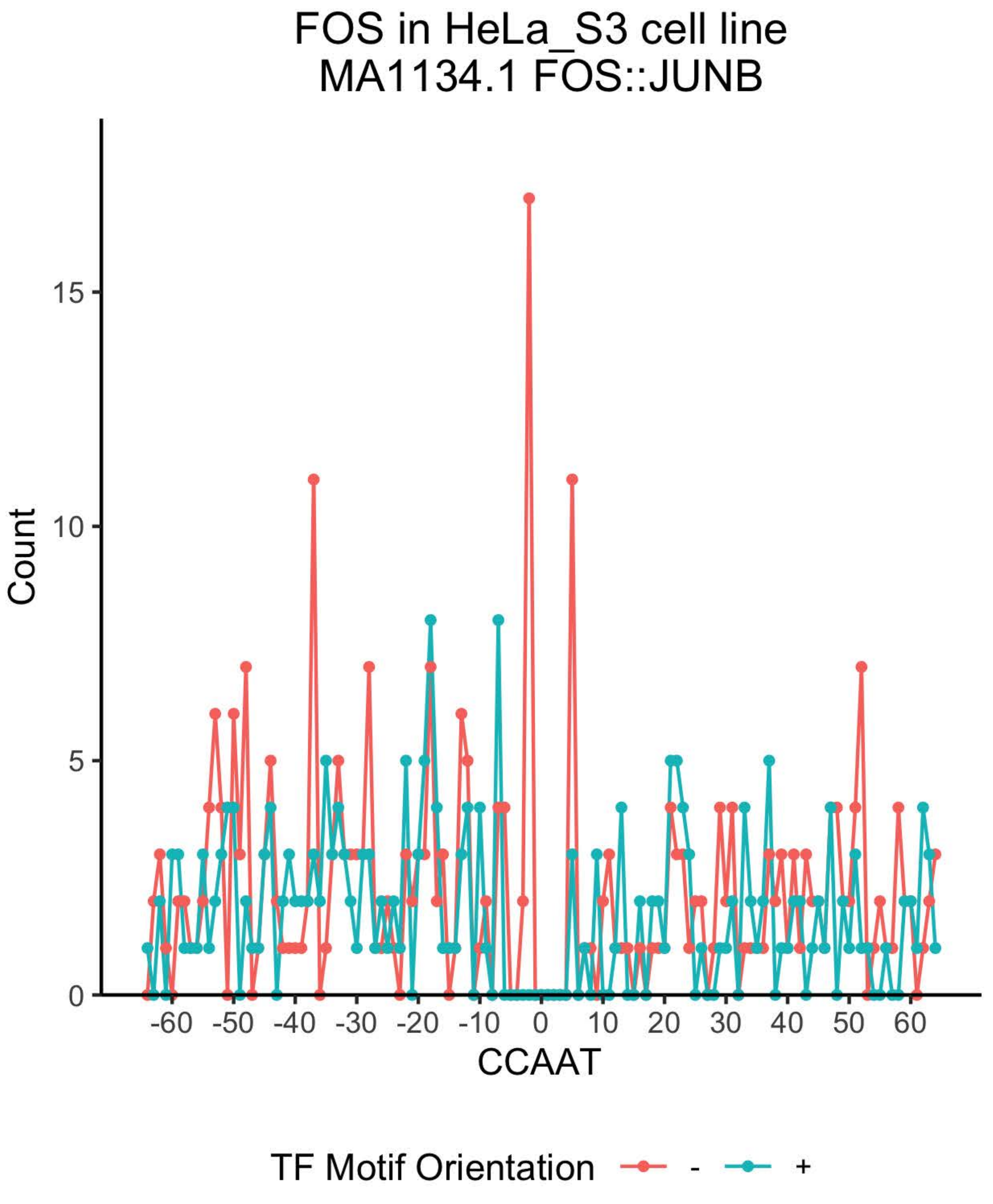
FOS in HeLa_S3 cell line
MA1134.1 FOS::JUNB
Count
15
10
5
0
-60 -50 -40 -30 -20 -10 0 10 20 30 40 50 60
CCAAT
TF Motif Orientation
-
+

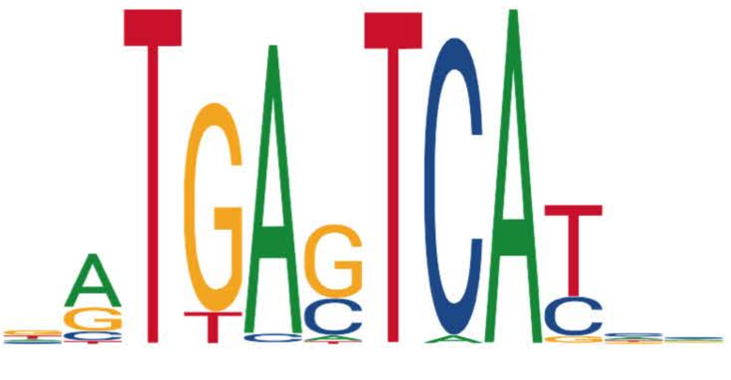

FOS in HeLa_S3 cell line
MA1141.1 FOS::JUND

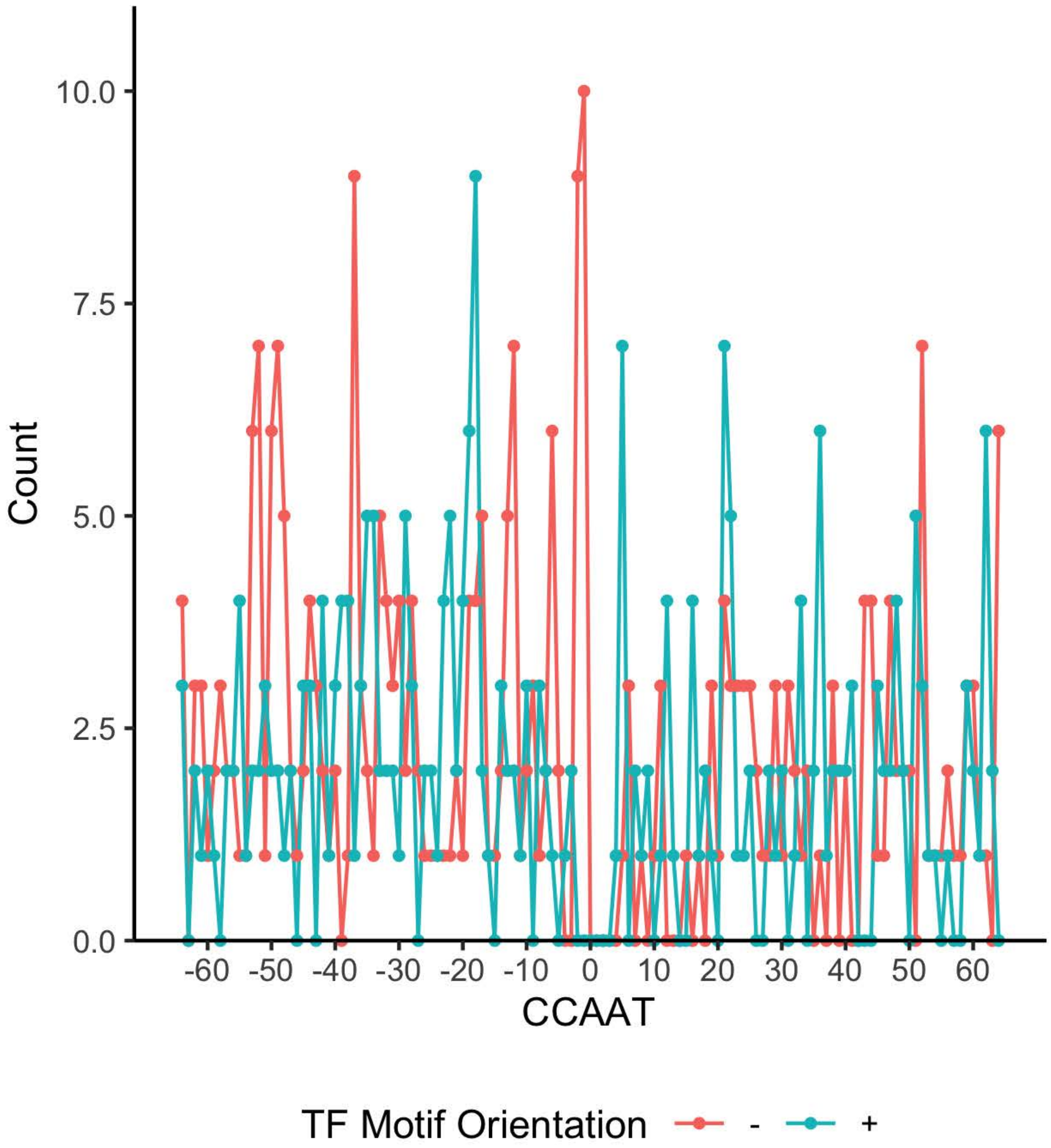

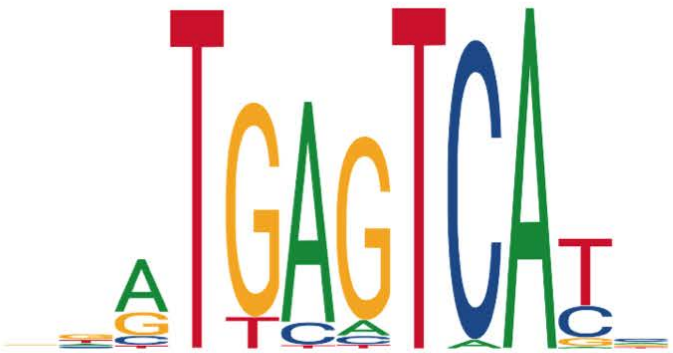

FOS in K562 cell line
MA0099.2 FOS::JUN

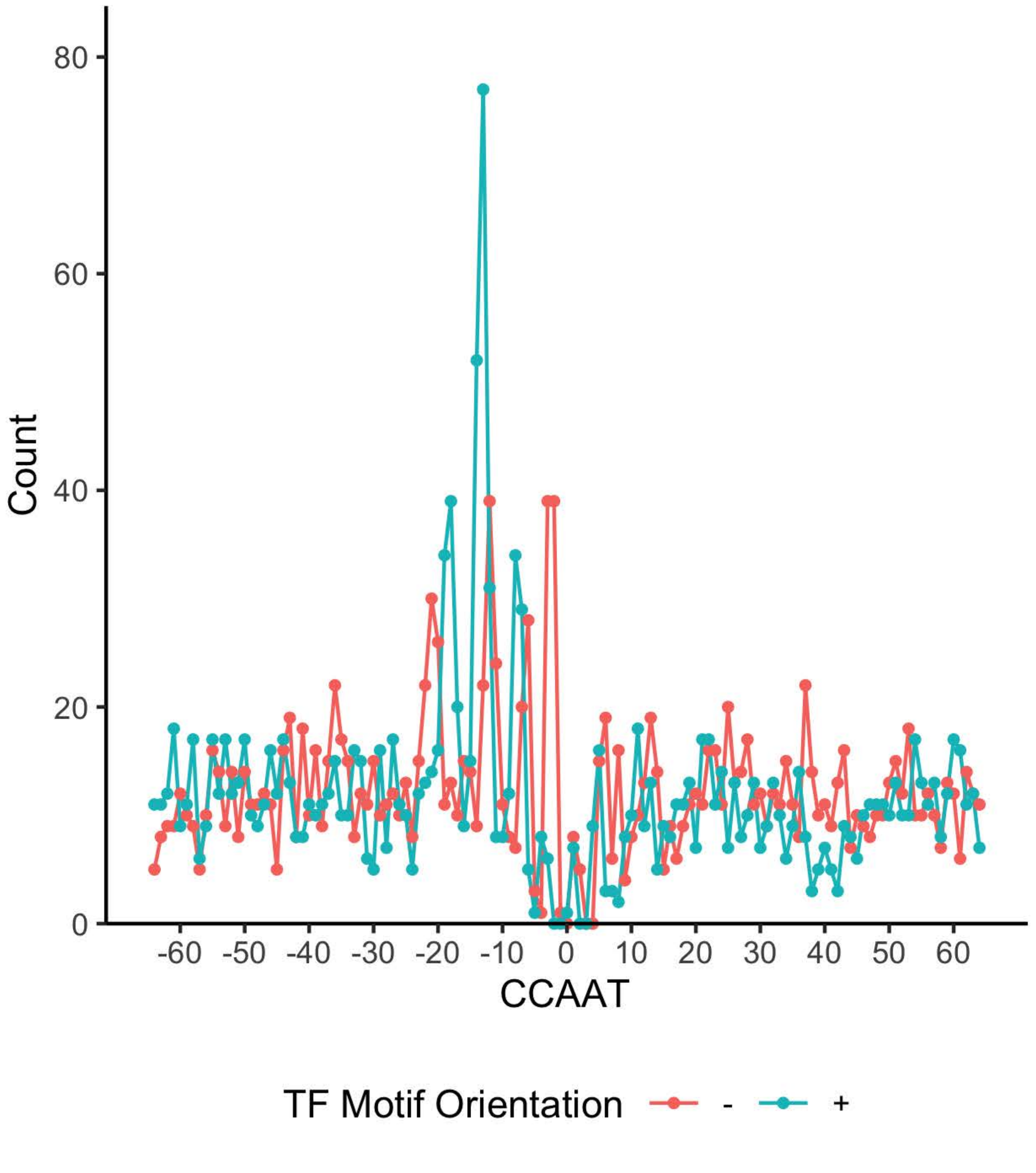

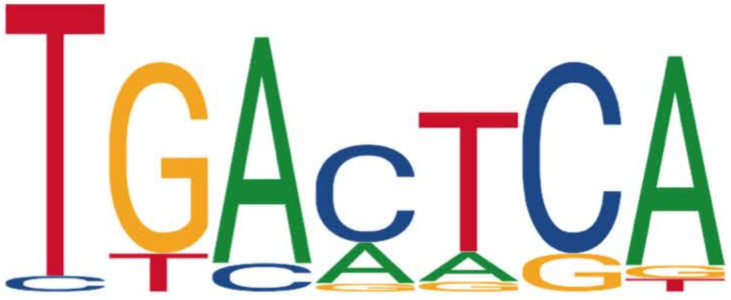

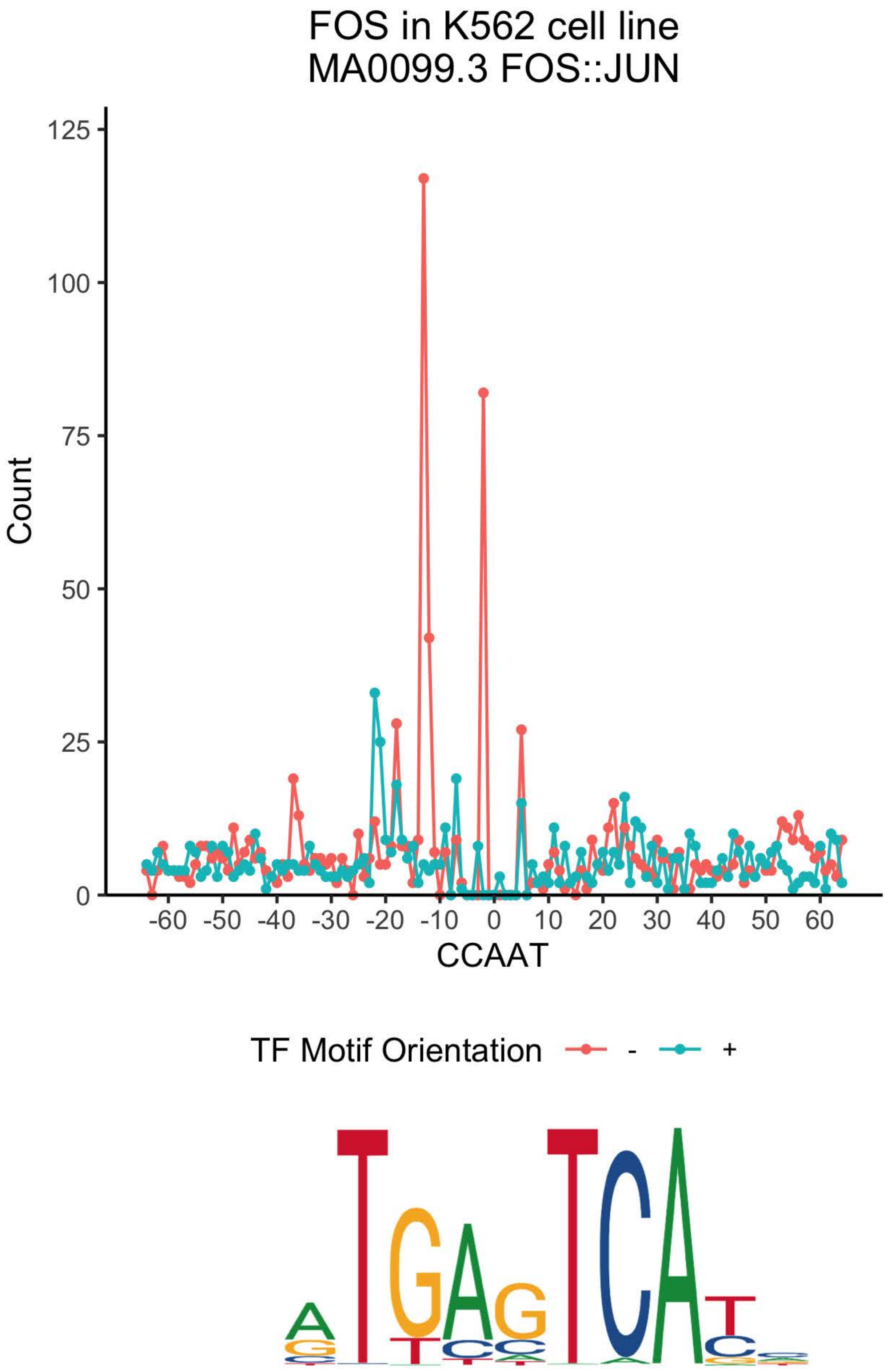
FOS in K562 cell line
MA0099.3 FOS::JUN
Count
125
100
75
50
25
0
-60 -50 -40 -30 -20 -10 0 10 20 30 40 50 60
CCAAT
TF Motif Orientation
-
+

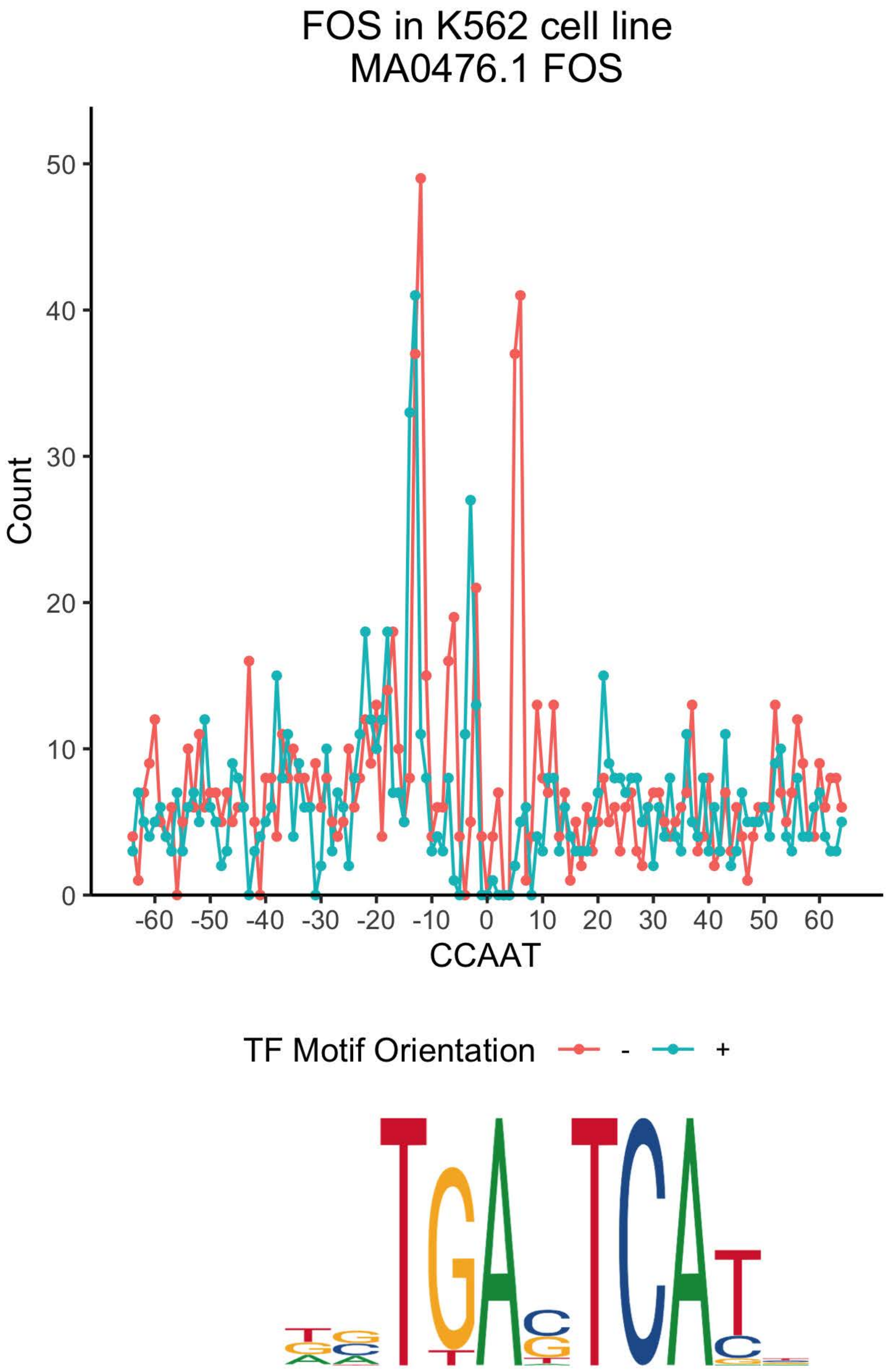
FOS in K562 cell line
MA0476.1 FOS
Count
50
40
30
20
10
0
-60 -50 -40 -30 -20 -10 0 10 20 30 40 50 60
CCAAT
TF Motif Orientation
-
+

FOS in K562 cell line
MA1126.1 FOS::JUN(var.2)

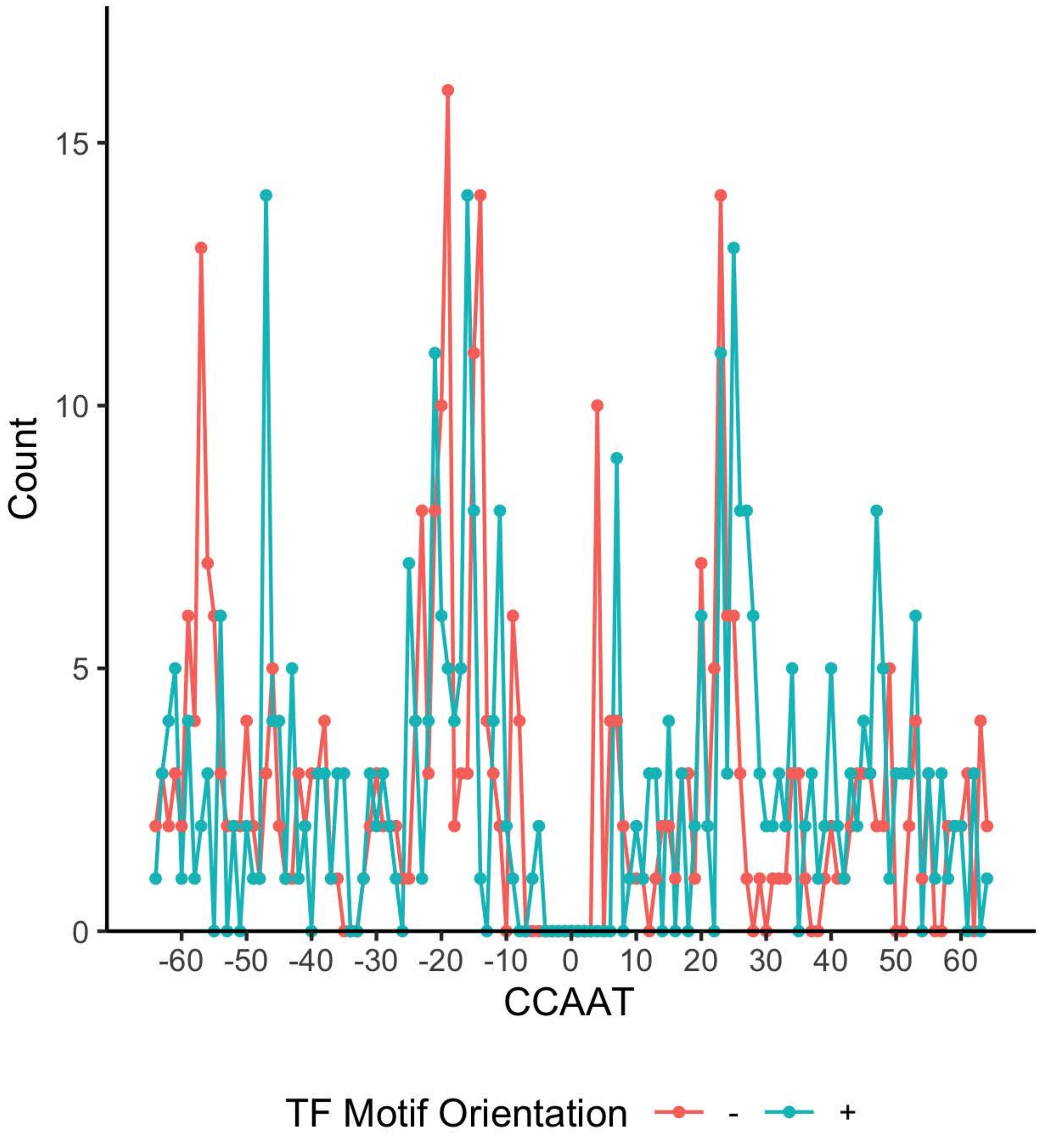

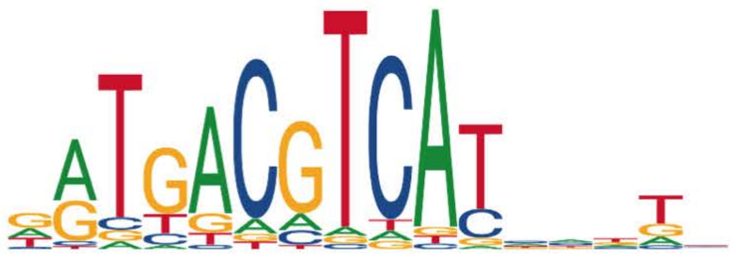

FOS in K562 cell line
MA1134.1 FOS::JUNB

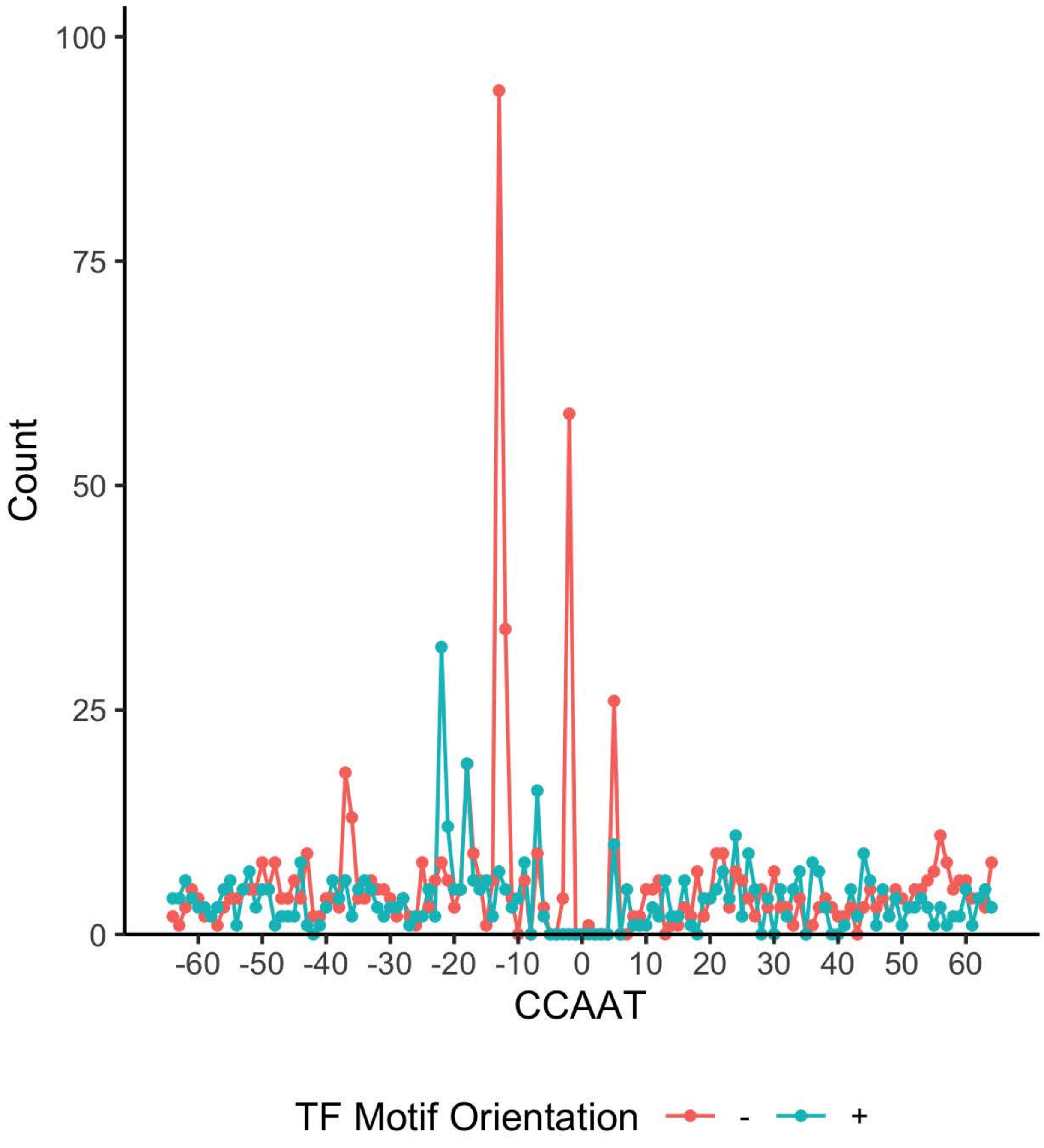

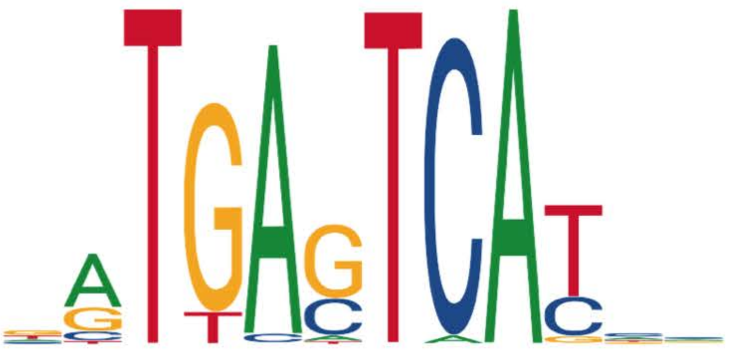

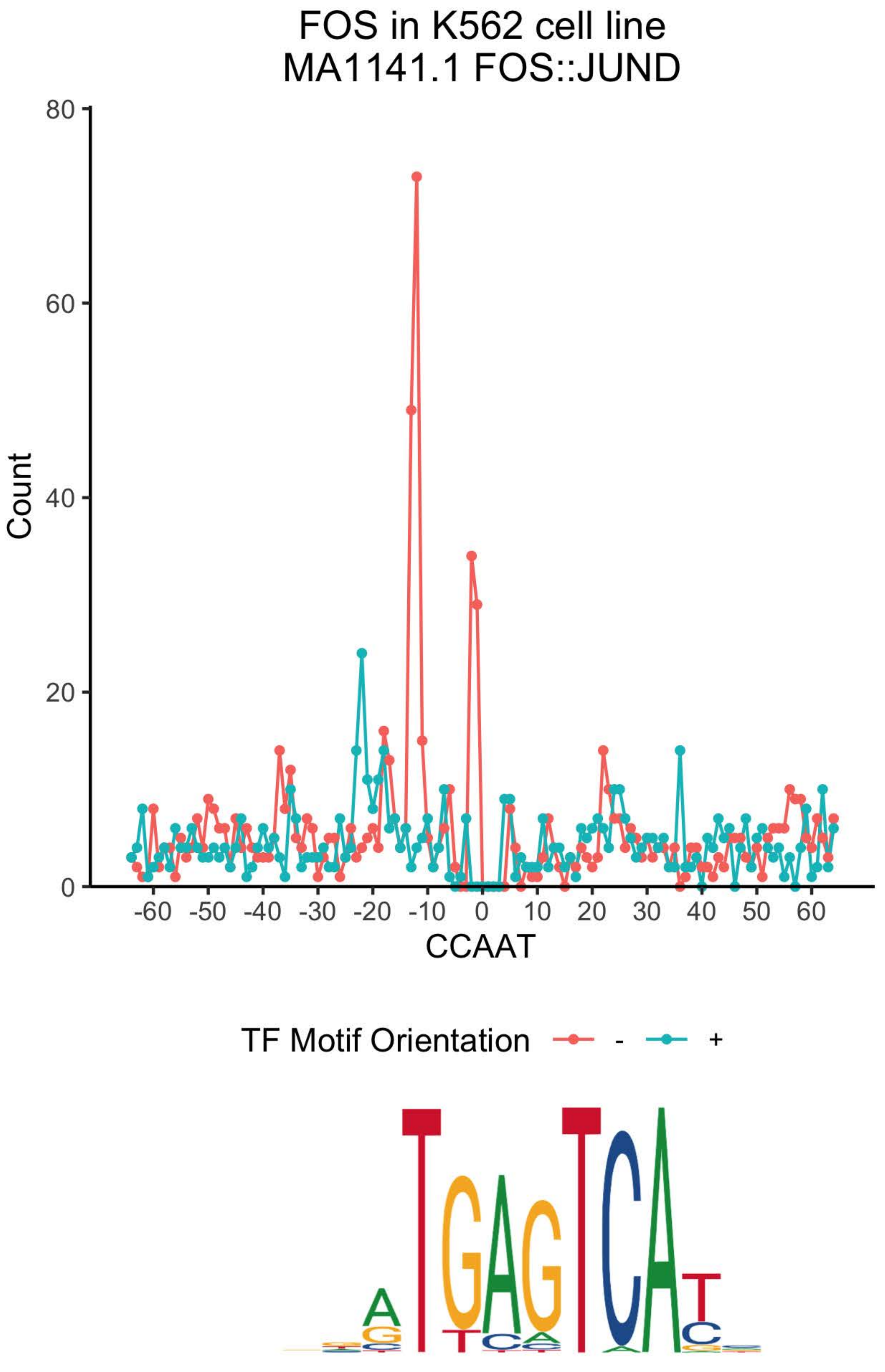

FOS in K562 cell line
MA1141.1 FOS::JUND
Count
CCAAT
0
20
40
60
80
-60
-50
-40
-30
-20
-10
0
10
20
30
40
50
60
TF Motif Orientation
-
+

IRF3 in HeLa_S3 cell line
MA1418.1 IRF3

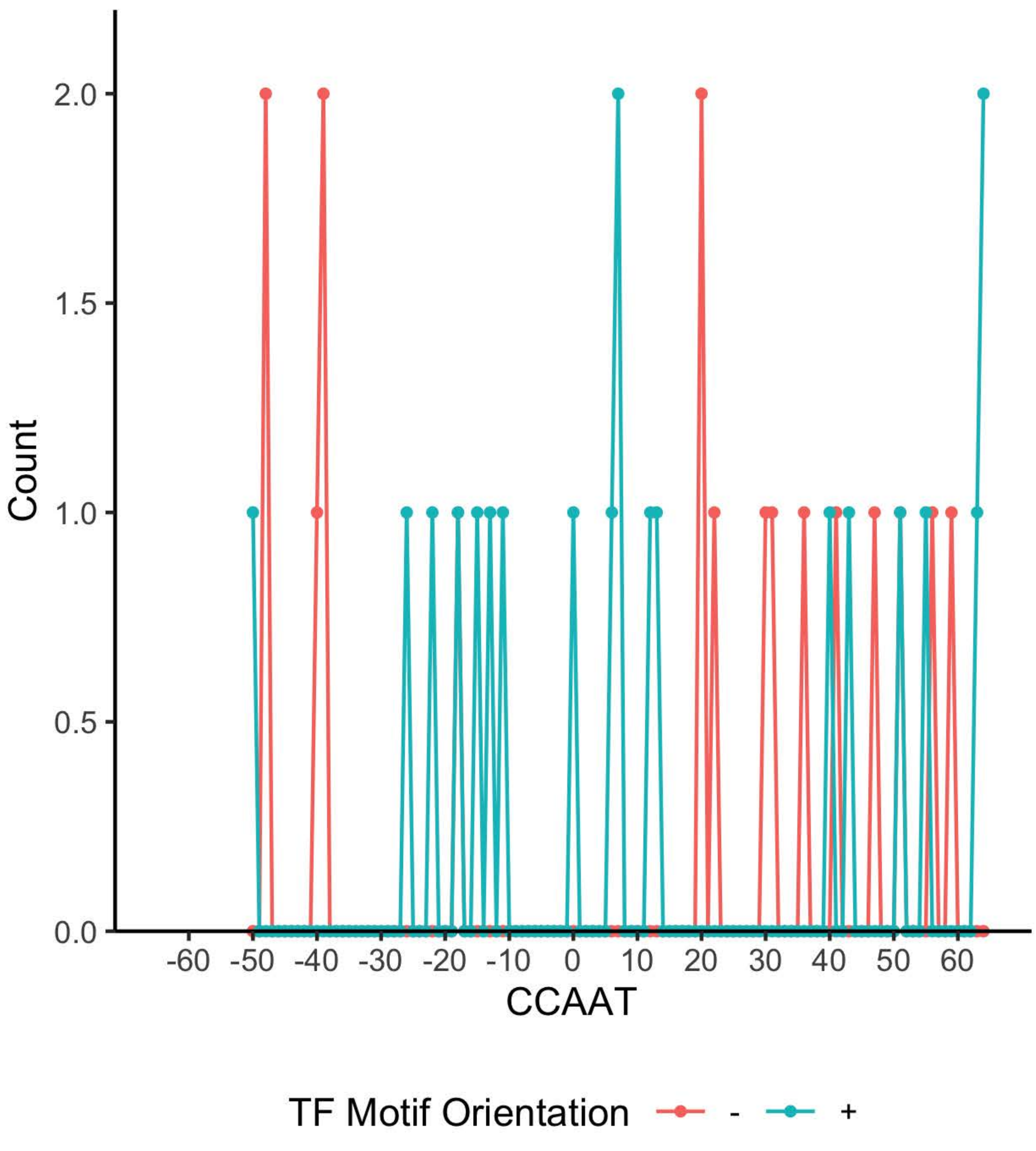

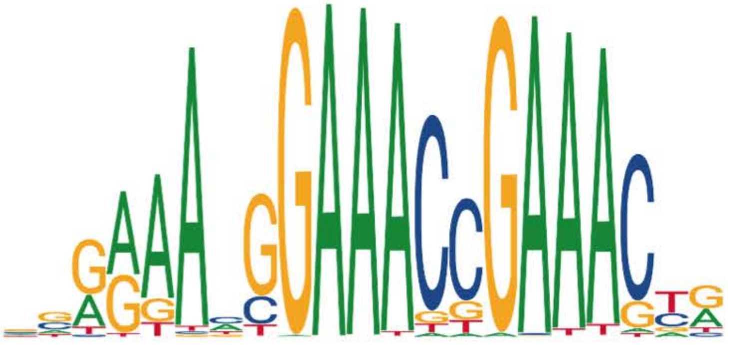

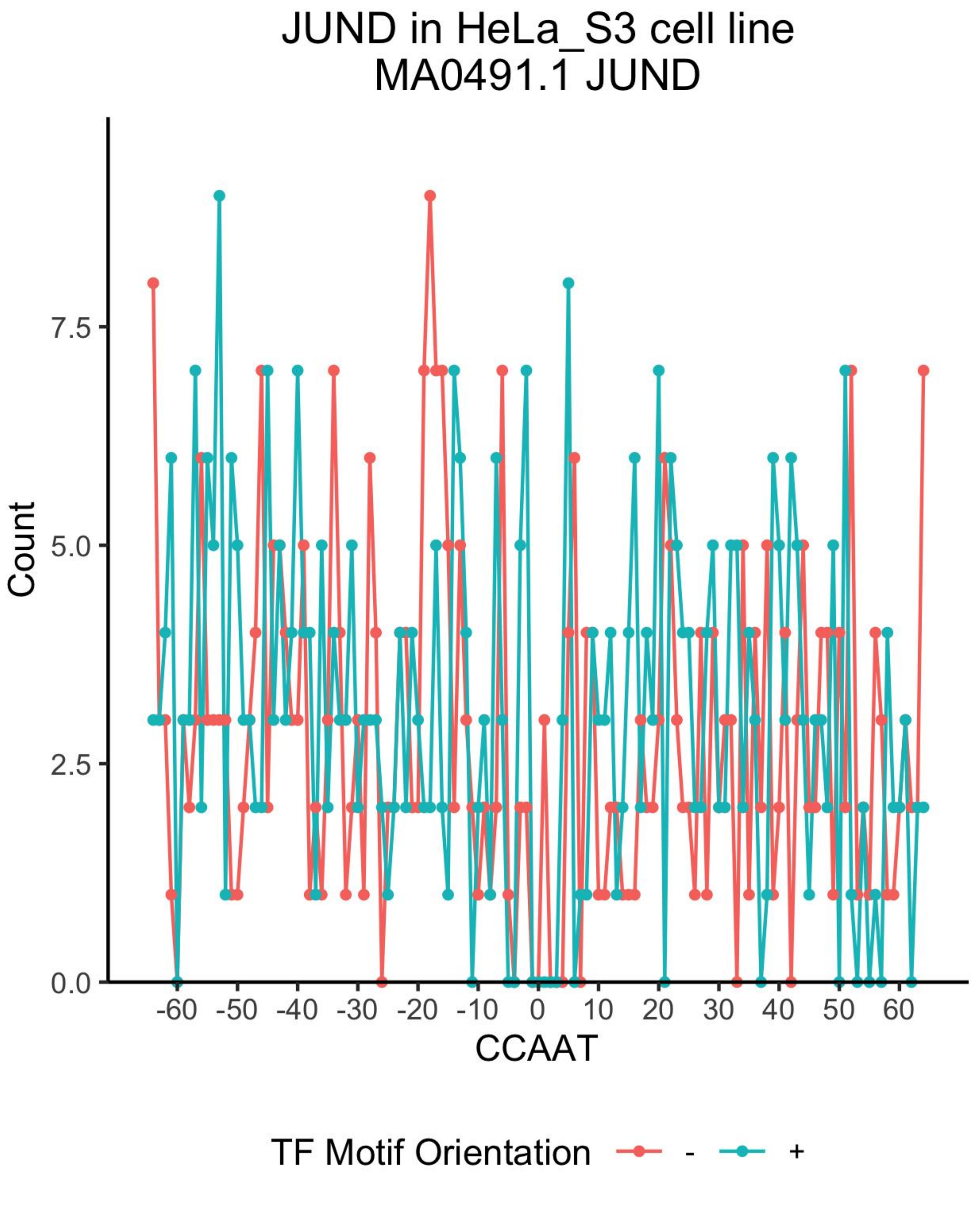

JUND in HeLa_S3 cell line
MA0491.1 JUND
Count
7.5
5.0
2.5
0.0
-60 -50 -40 -30 -20 -10 0 10 20 30 40 50 60
CCAAT
TF Motif Orientation
-
+

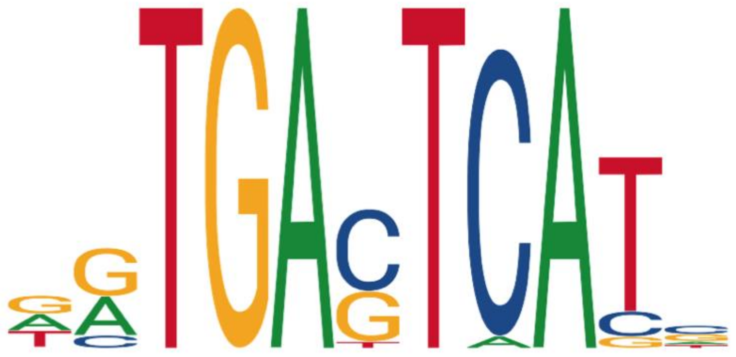

JUND in HeLa_S3 cell line
MA0491.2 JUND

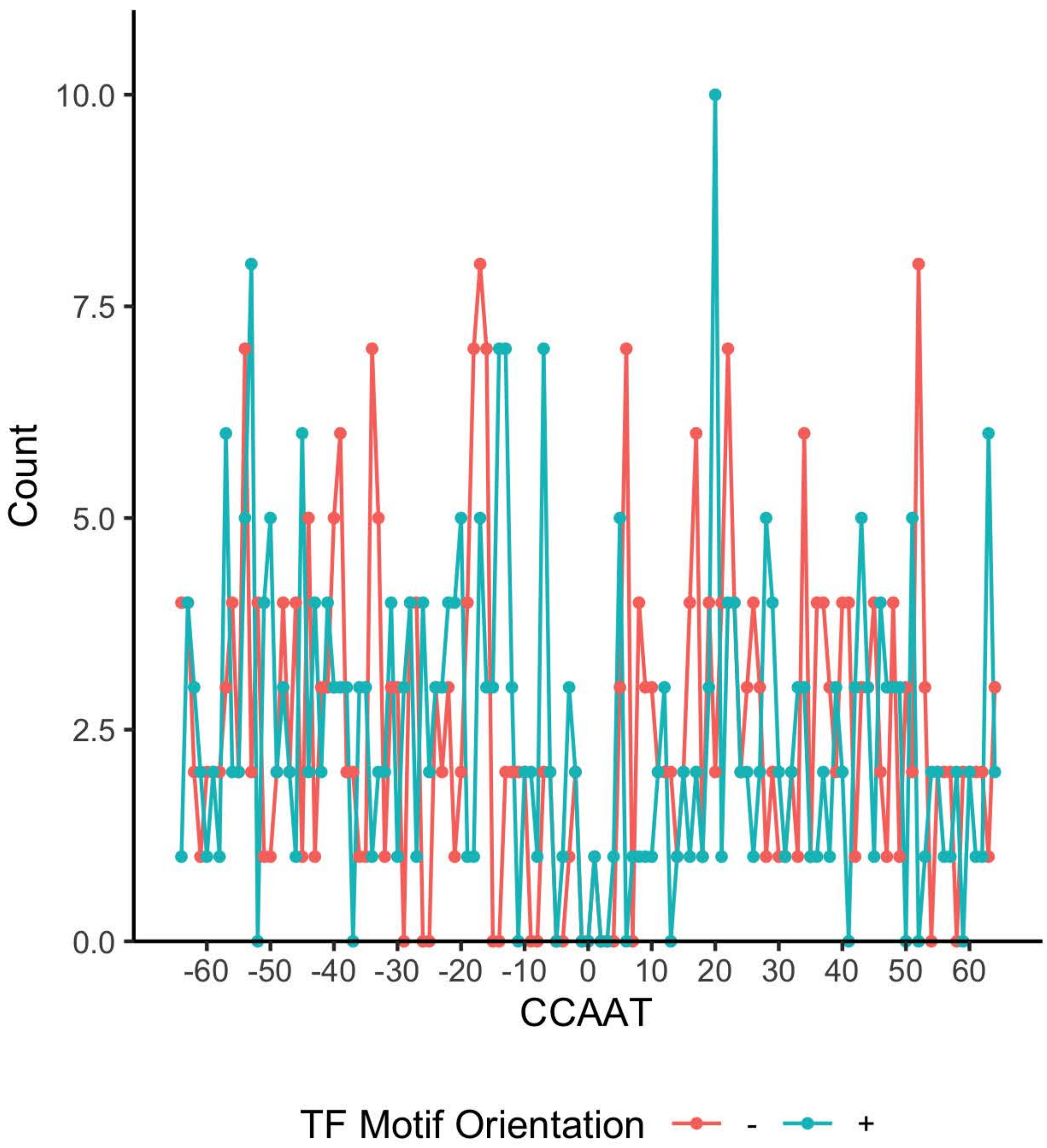

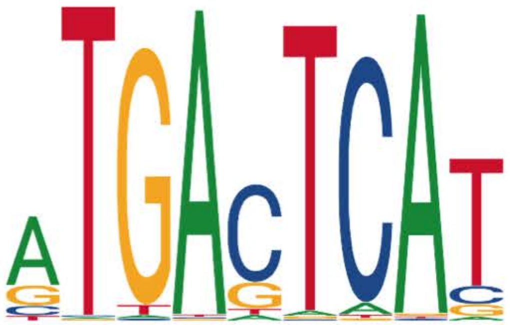

JUND in HeLa_S3 cell line
MA0492.1 JUND(var.2)

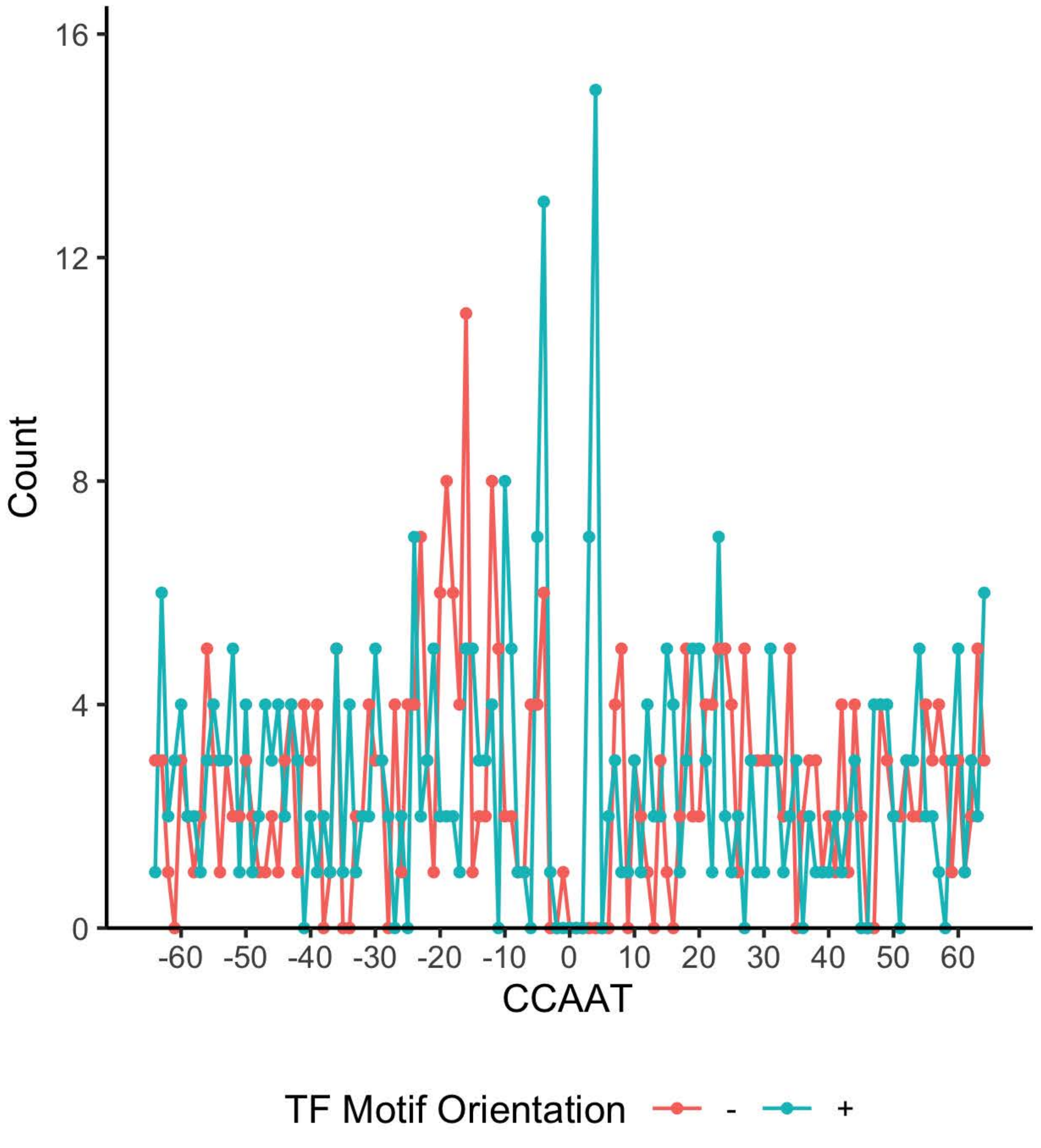

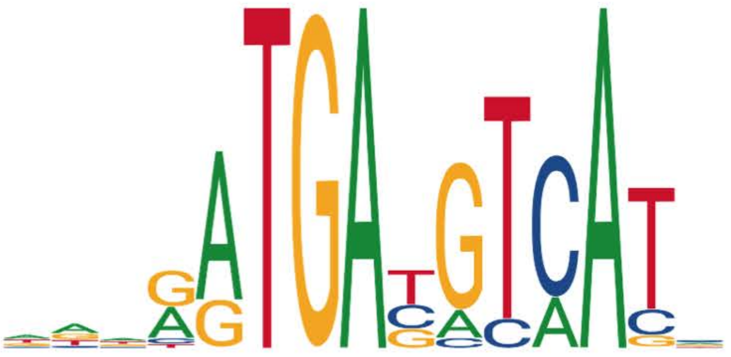

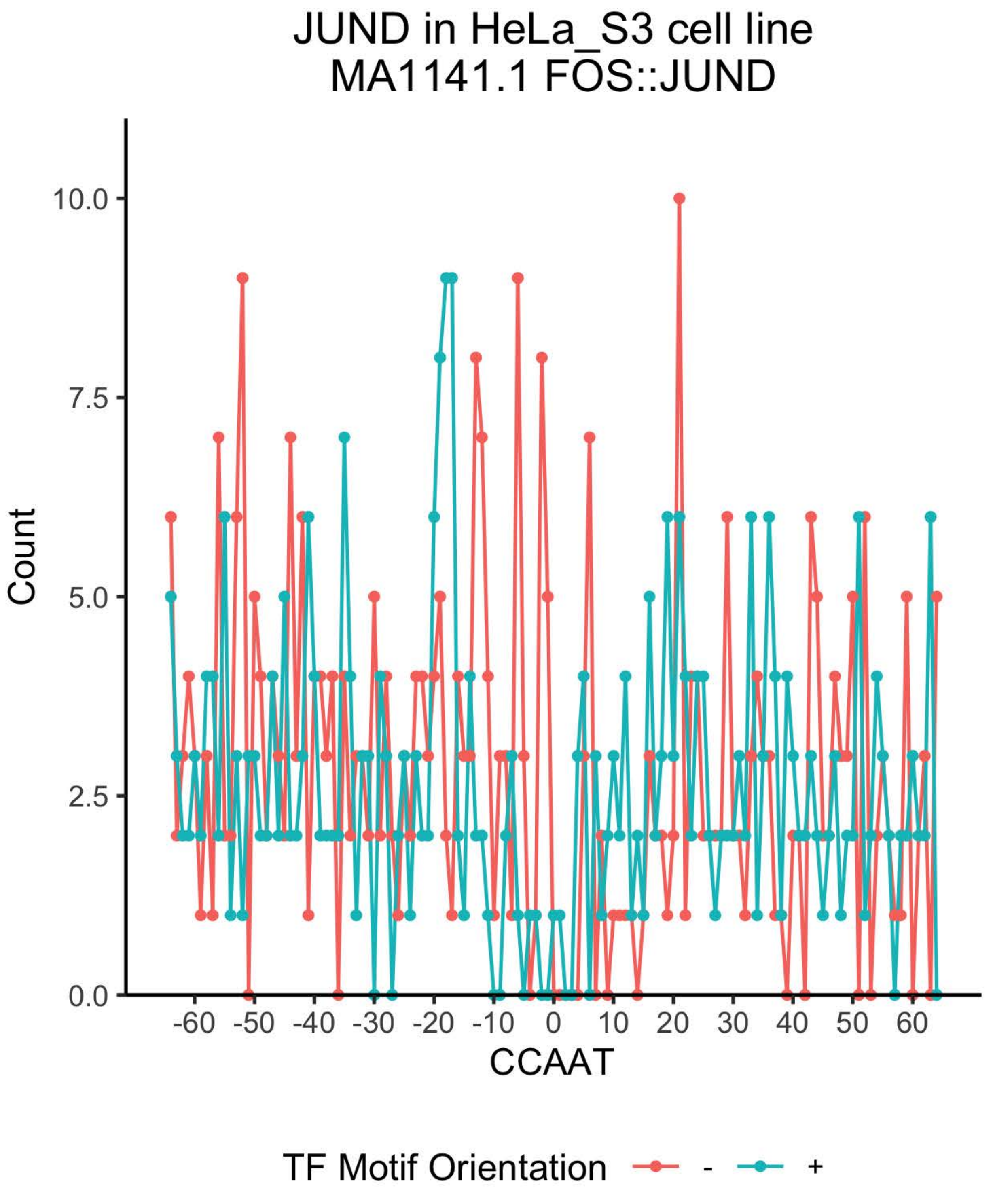
JUND in HeLa_S3 cell line
MA1141.1 FOS::JUND
Count
10.0
7.5
5.0
2.5
0.0
-60
-50
-40
-30
-20
-10
0
10
20
30
40
50
60
CCAAT
TF Motif Orientation
-
+

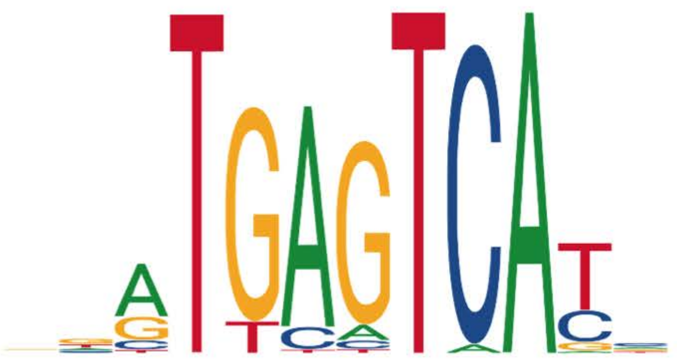

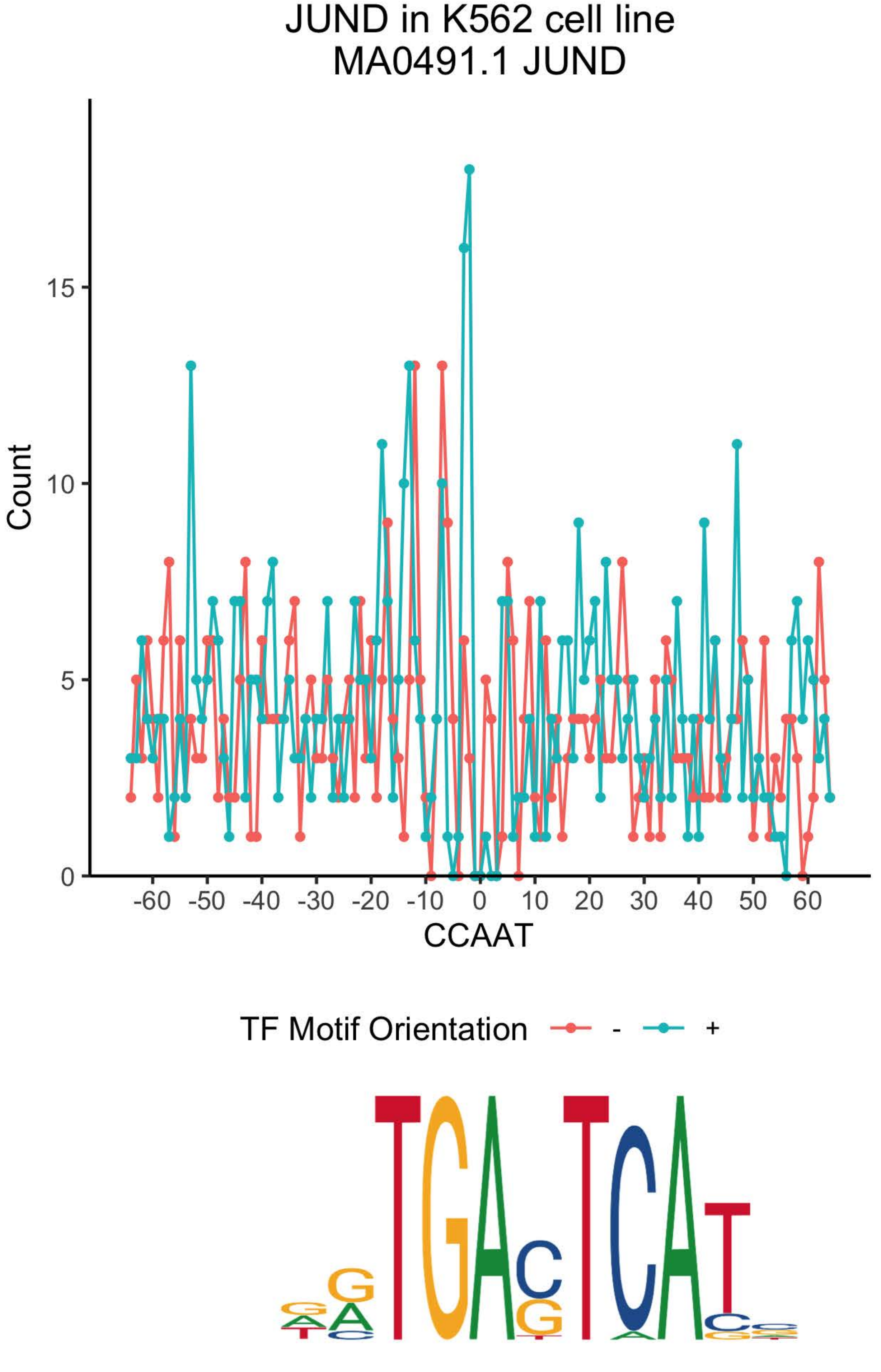
JUND in K562 cell line
MA0491.1 JUND
Count
15
10
5
0
-60 -50 -40 -30 -20 -10 0 10 20 30 40 50 60
CCAAT
TF Motif Orientation - +

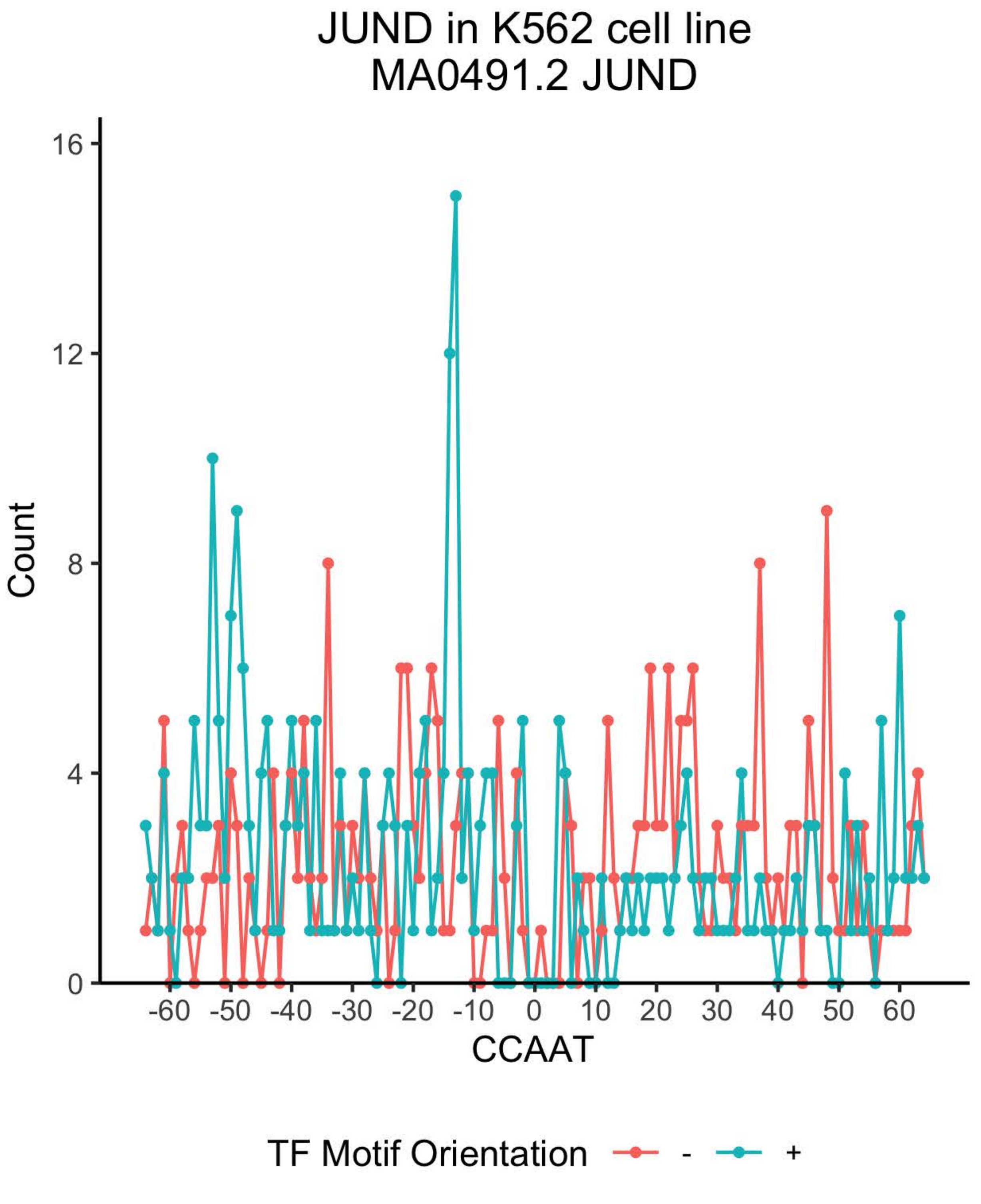
JUND in K562 cell line
MA0491.2 JUND
Count
16
12
8
4
0
-60 -50 -40 -30 -20 -10 0 10 20 30 40 50 60
CCAAT
TF Motif Orientation
-
+

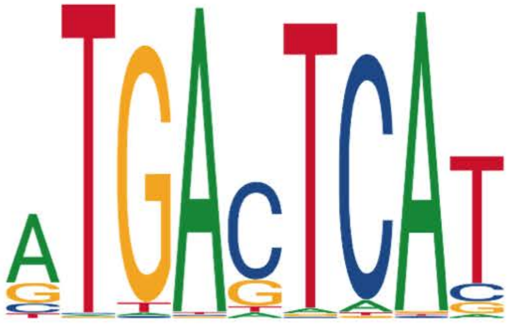

JUND in K562 cell line
MA0492.1 JUND(var.2)

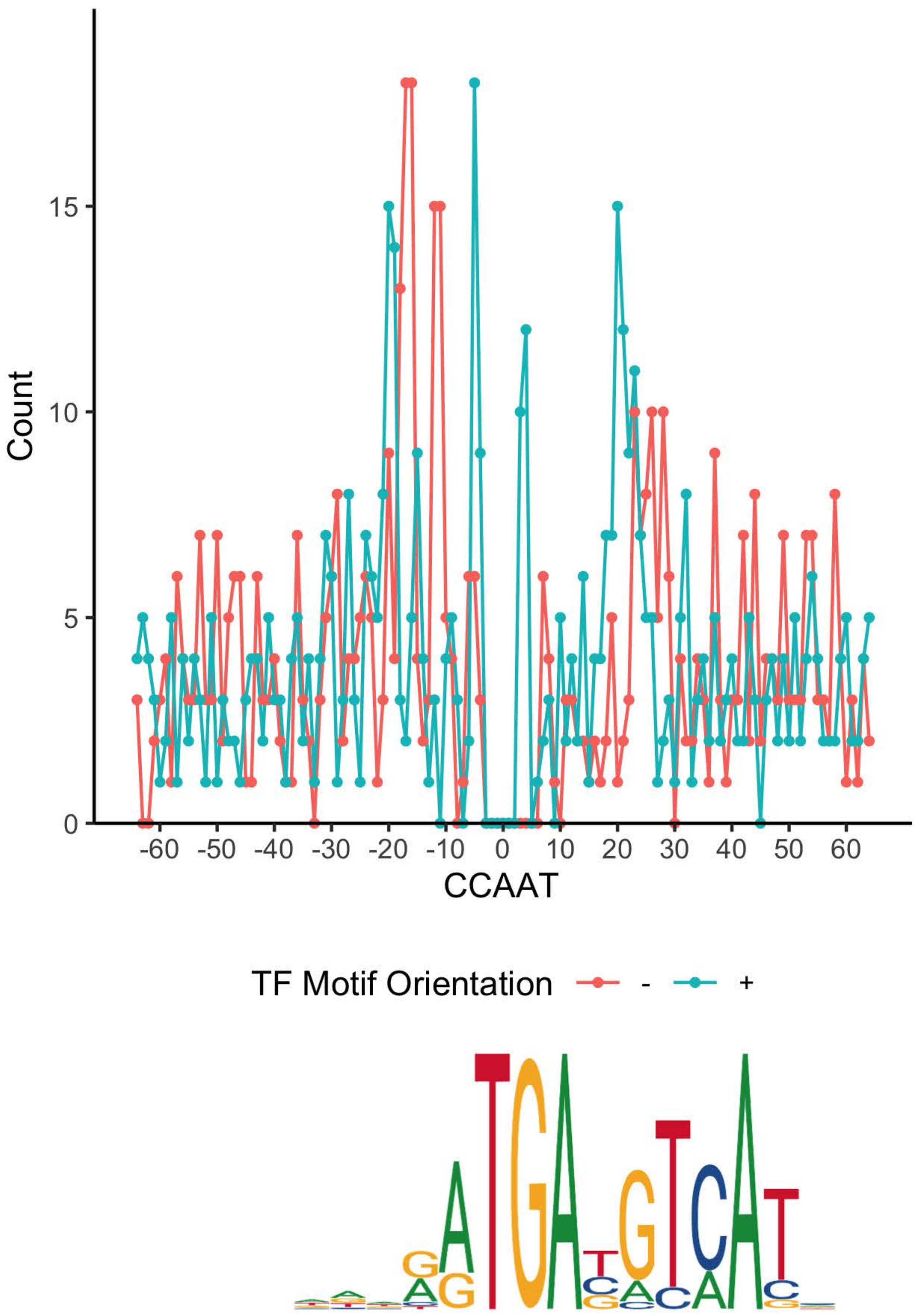

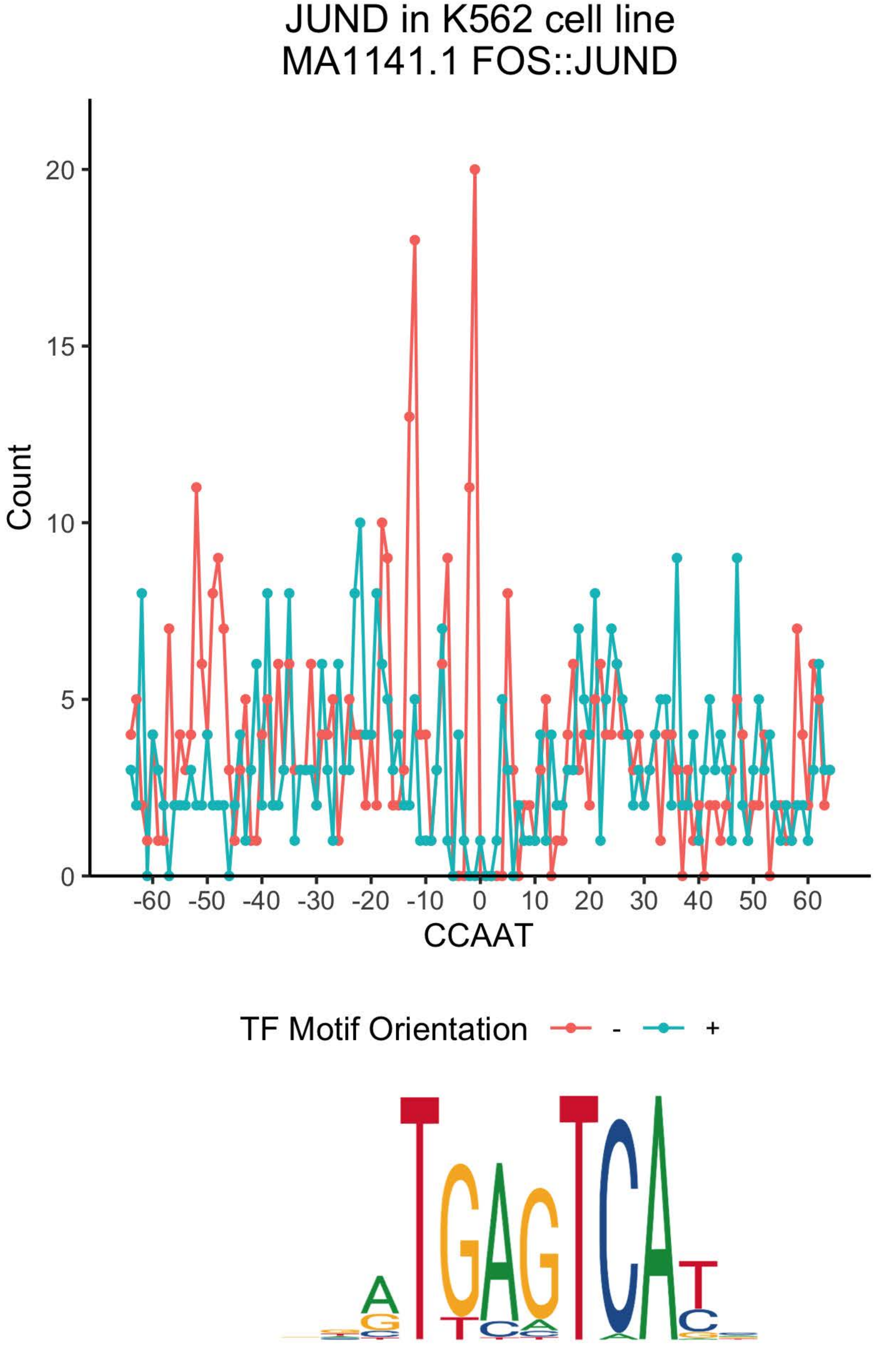

JUND in K562 cell line
MA1141.1 FOS::JUND
Count
20
15
10
5
0
-60 -50 -40 -30 -20 -10 0 10 20 30 40 50 60
CCAAT
TF Motif Orientation
-
+

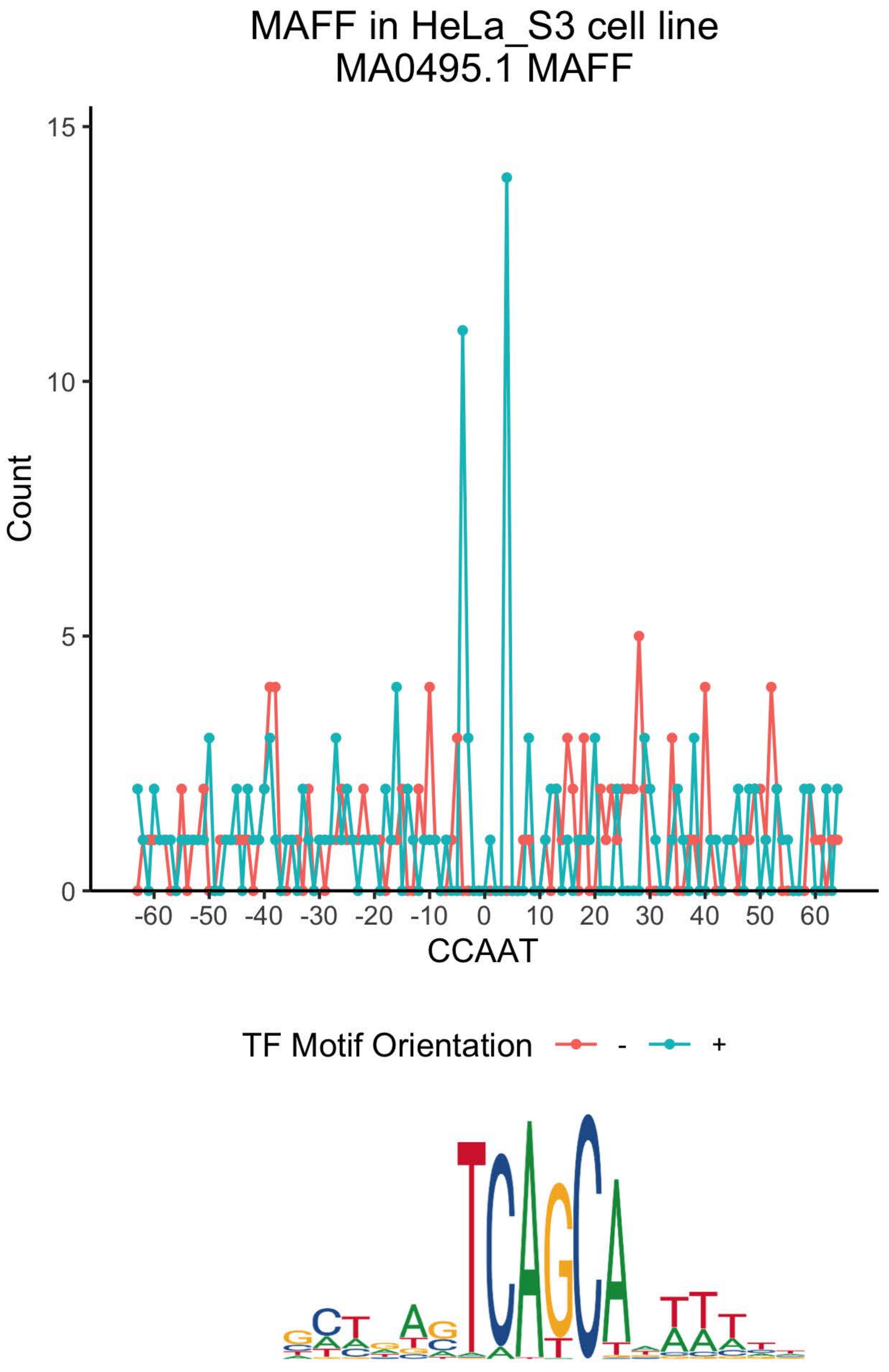
MAFF in HeLa_S3 cell line
MA0495.1 MAFF
Count
15
10
5
0
-60 -50 -40 -30 -20 -10 0 10 20 30 40 50 60
CCAAT
TF Motif Orientation
-
+

MAFF in HeLa_S3 cell line
MA0495.2 MAFF

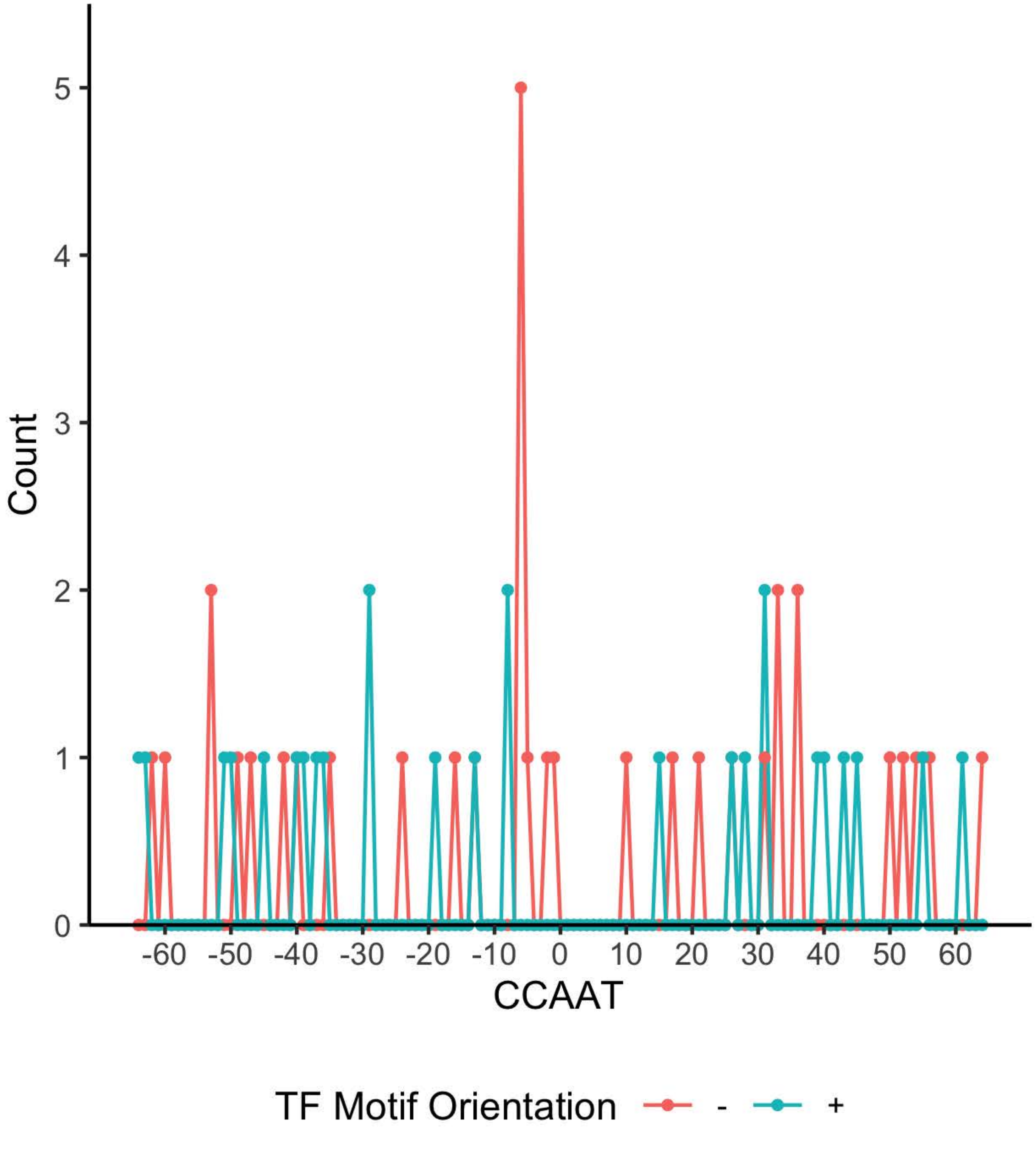

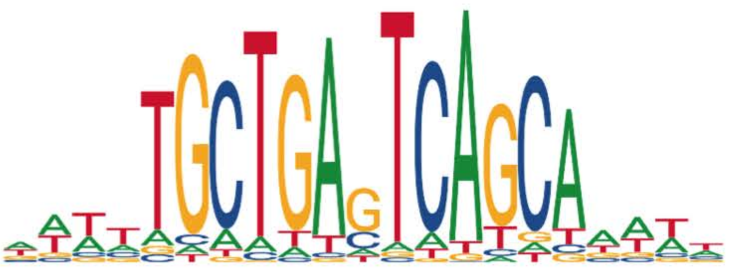

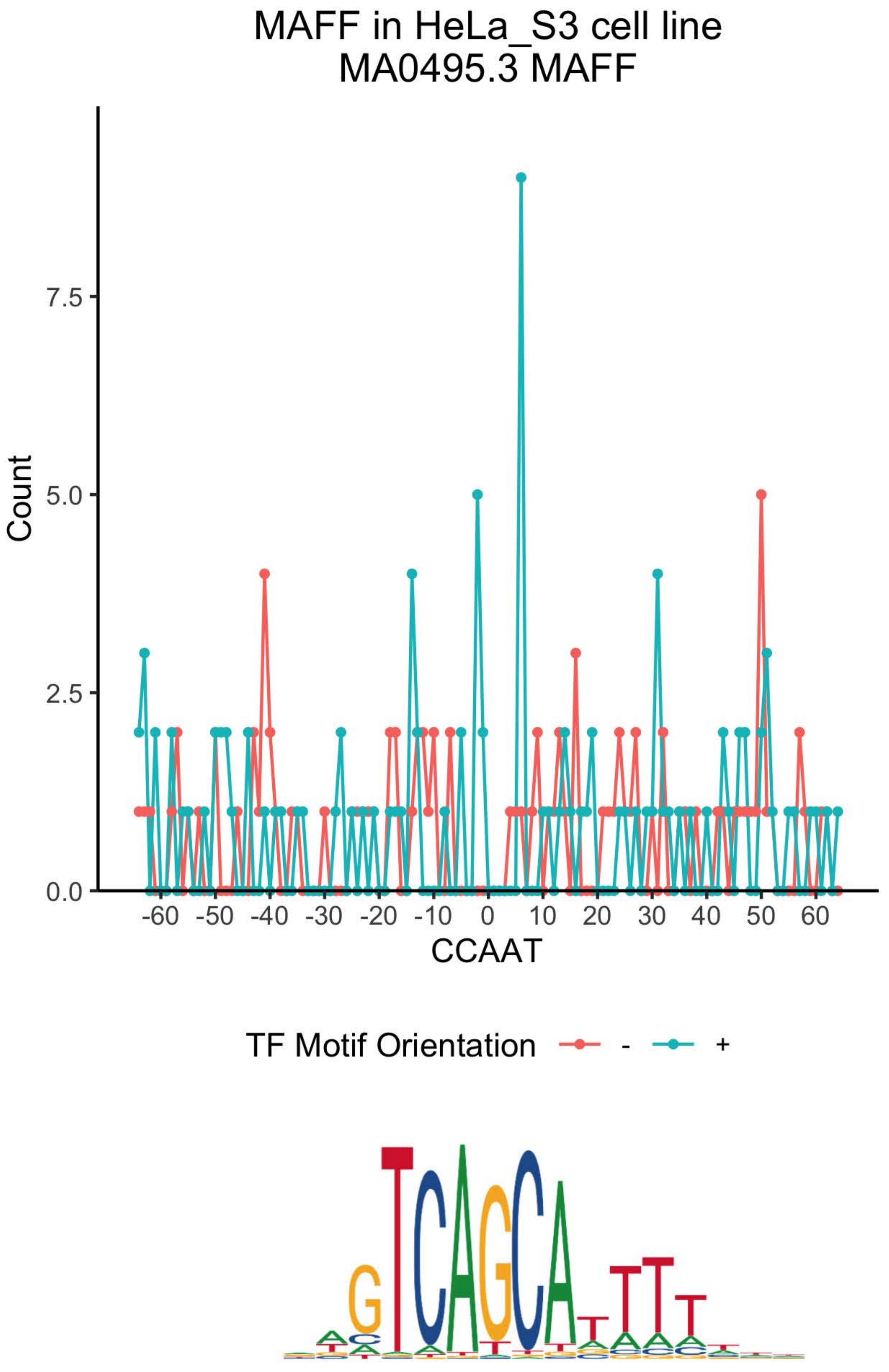

MAFF in HeLa_S3 cell line
MA0495.3 MAFF
Count
7.5
5.0
2.5
0.0
-60
-50
-40
-30
-20
-10
0
10
20
30
40
50
60
CCAAT
TF Motif Orientation
-
+

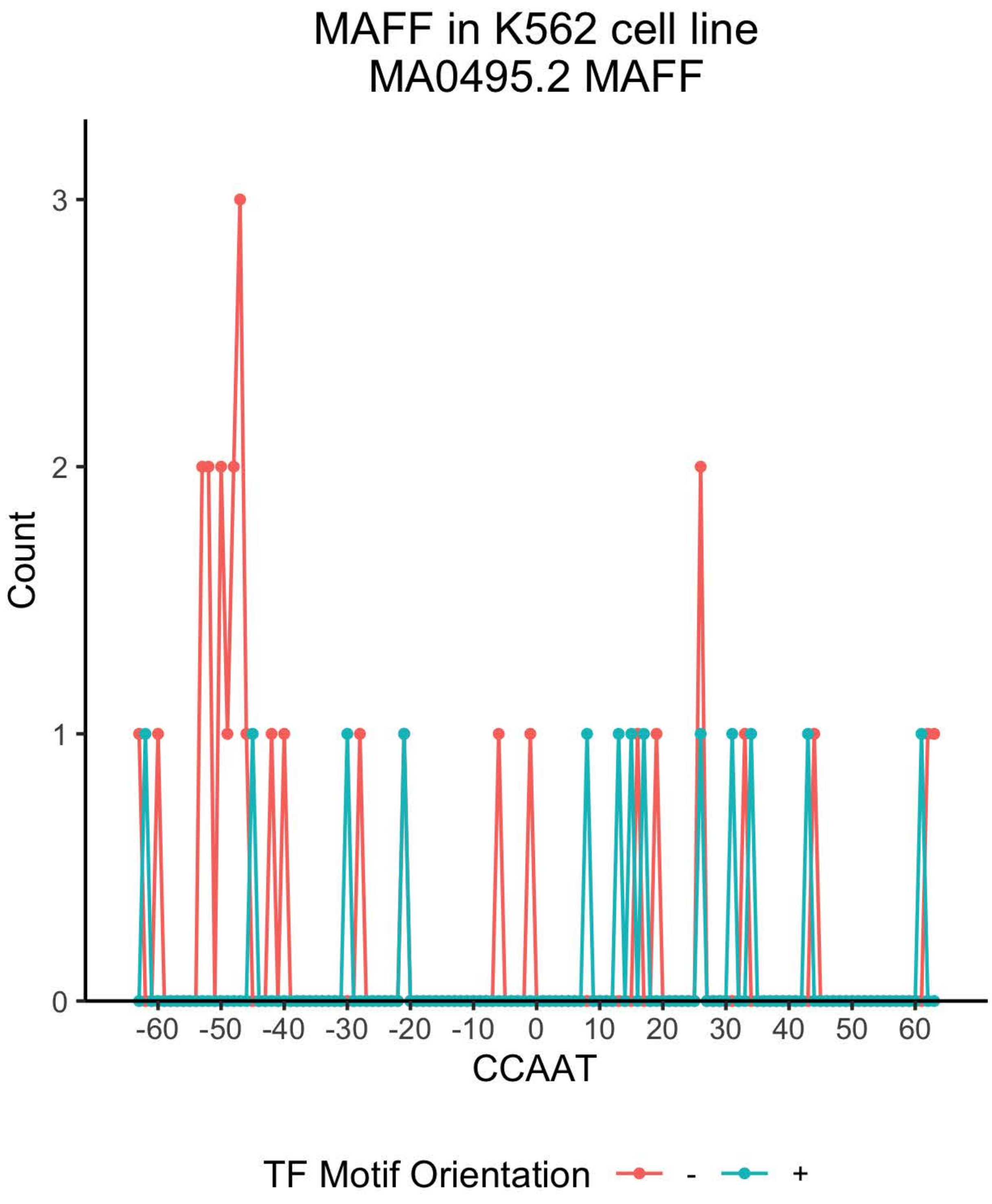

MAFF in K562 cell line
MA0495.2 MAFF
Count
CCAAT
3
2
1
0
-60
-50
-40
-30
-20
-10
0
10
20
30
40
50
60
TF Motif Orientation
-
+

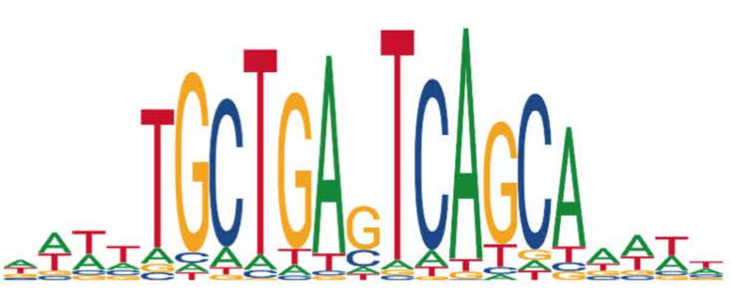

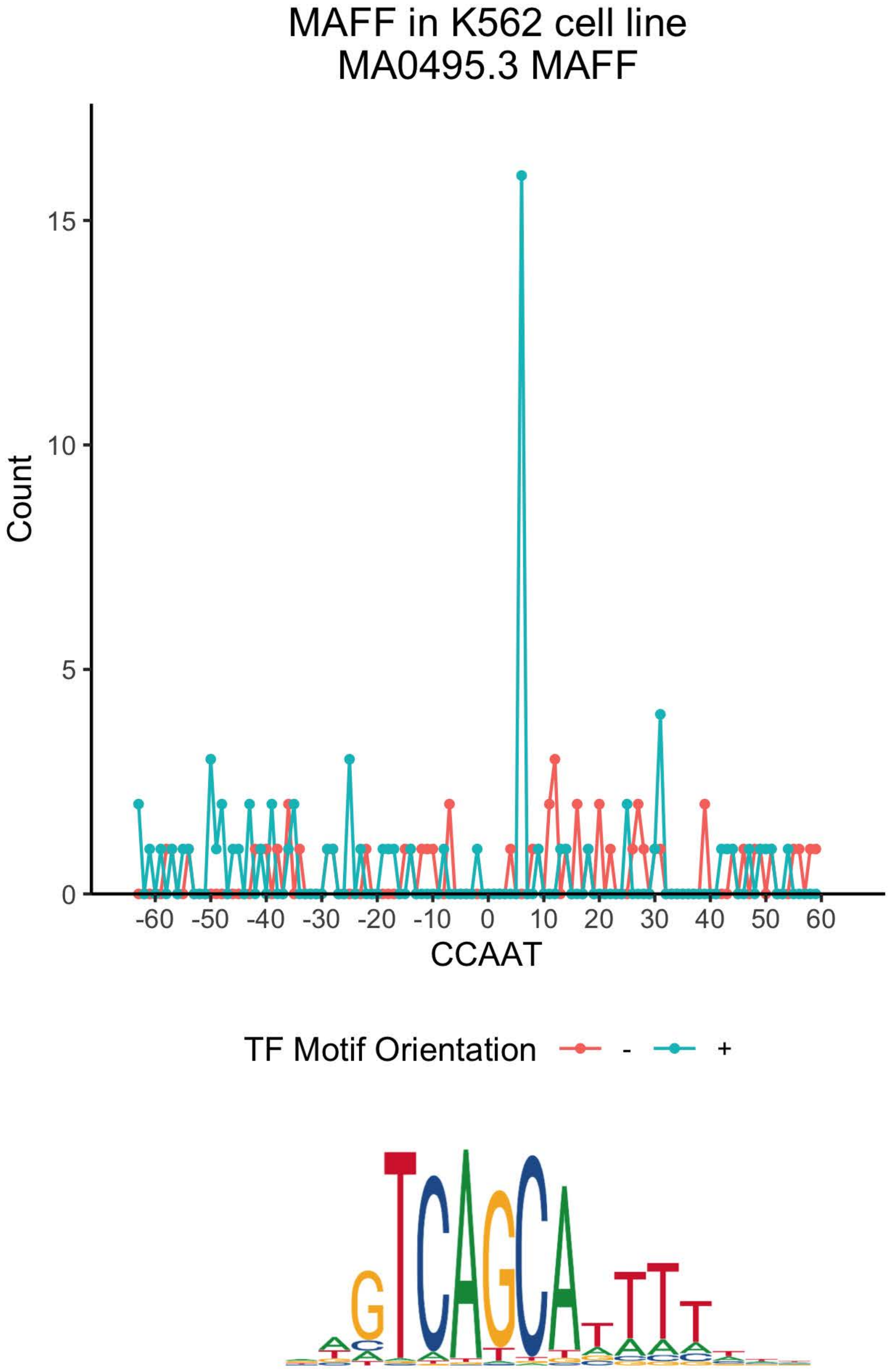
MAFF in K562 cell line
MA0495.3 MAFF
Count
15
10
5
0
-60
-50
-40
-30
-20
-10
0
10
20
30
40
50
60
CCAAT
TF Motif Orientation
-
+

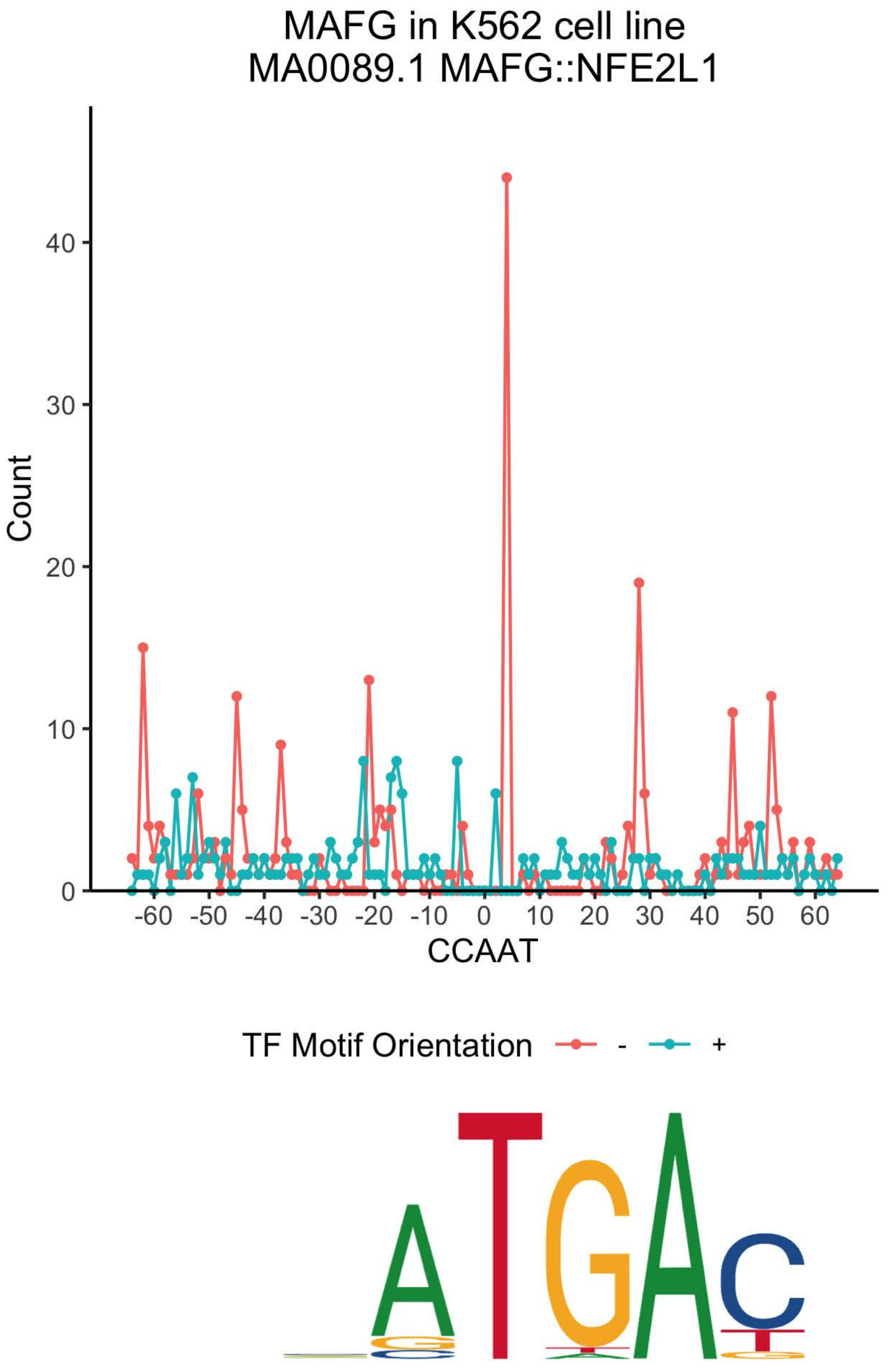
MAFG in K562 cell line
MA0089.1 MAFG::NFE2L1
Count
0
10
20
30
40
-60
-50
-40
-30
-20
-10
0
10
20
30
40
50
60
CCAAT
TF Motif Orientation
-
+

MAFG in K562 cell line
MA0659.1 MAFG

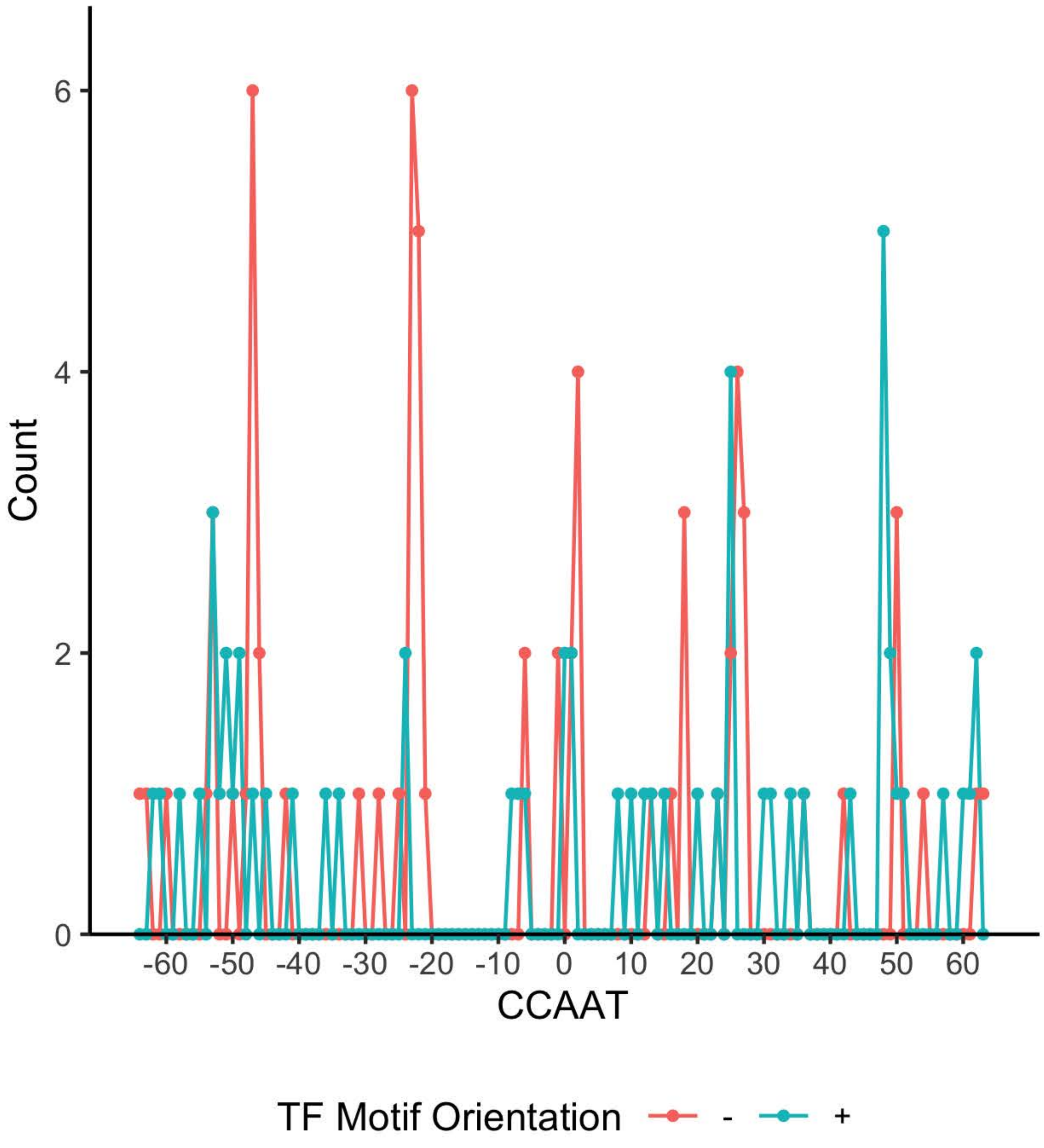

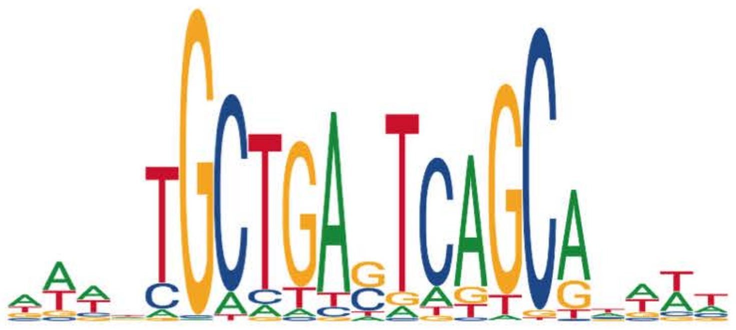

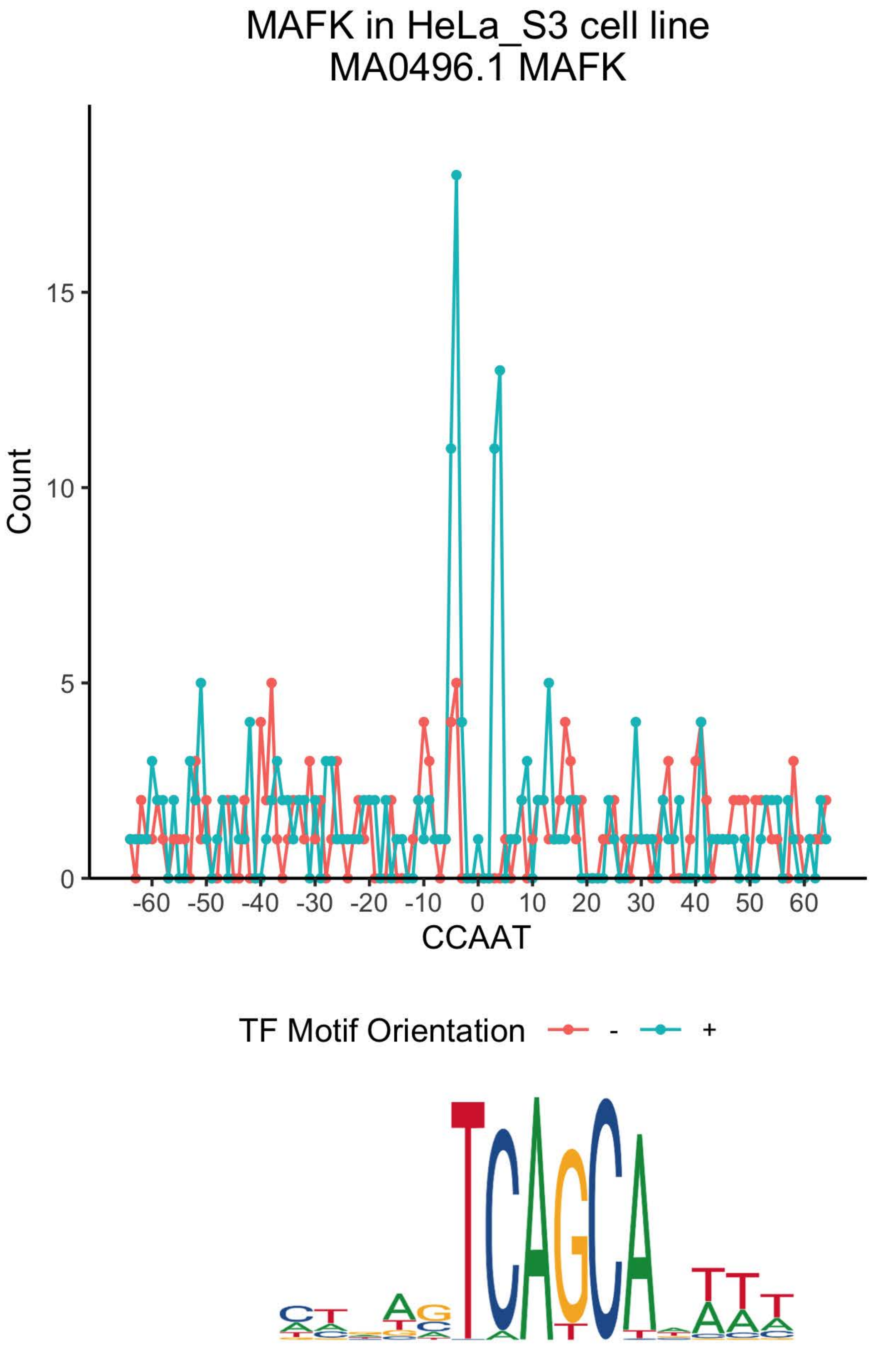
MAFK in HeLa_S3 cell line
MA0496.1 MAFK
Count
15
10
5
0
-60 -50 -40 -30 -20 -10 0 10 20 30 40 50 60
CCAAT
TF Motif Orientation
-
+

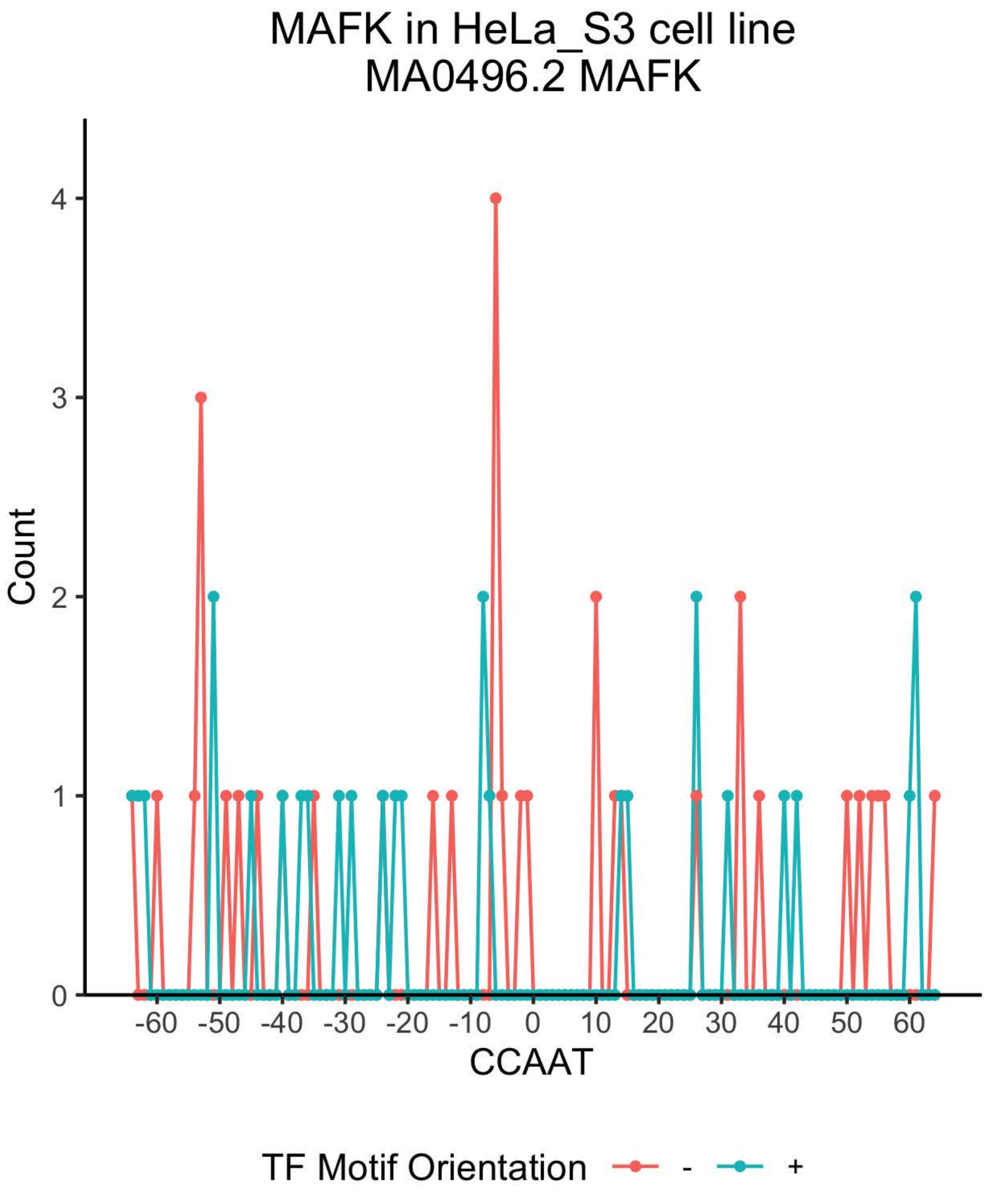
MAFK in HeLa_S3 cell line
MA0496.2 MAFK
Count
0
1
2
3
4
-60
-50
-40
-30
-20
-10
0
10
20
30
40
50
60
CCAAT
TF Motif Orientation
-
+

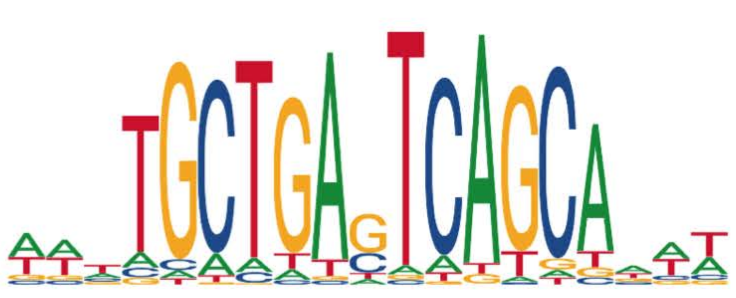

MAFK in HeLa_S3 cell line
MA0496.3 MAFK

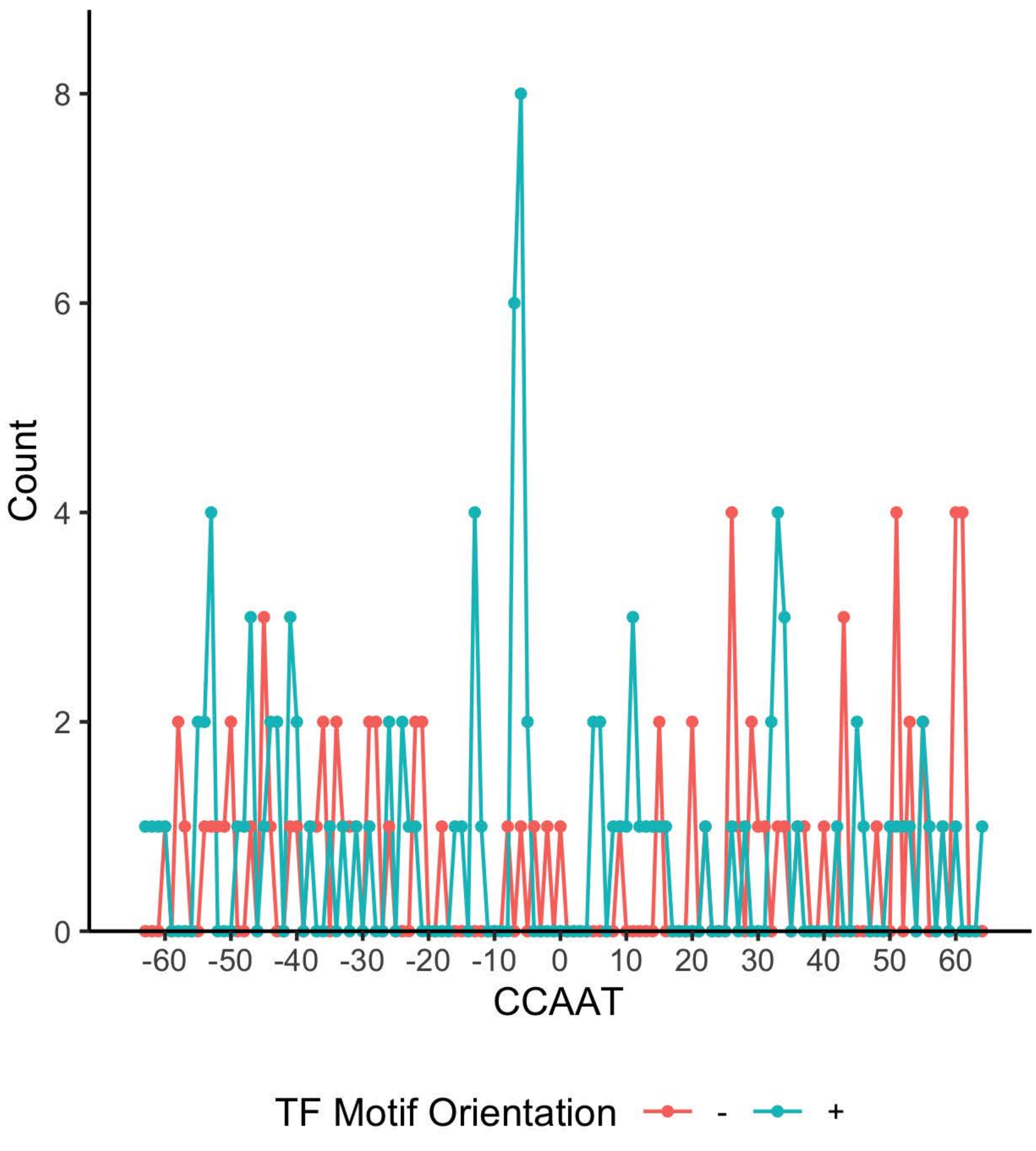

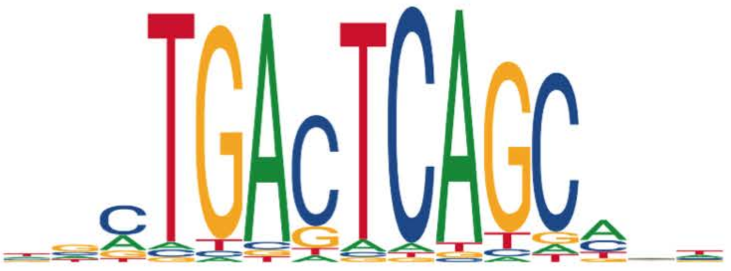

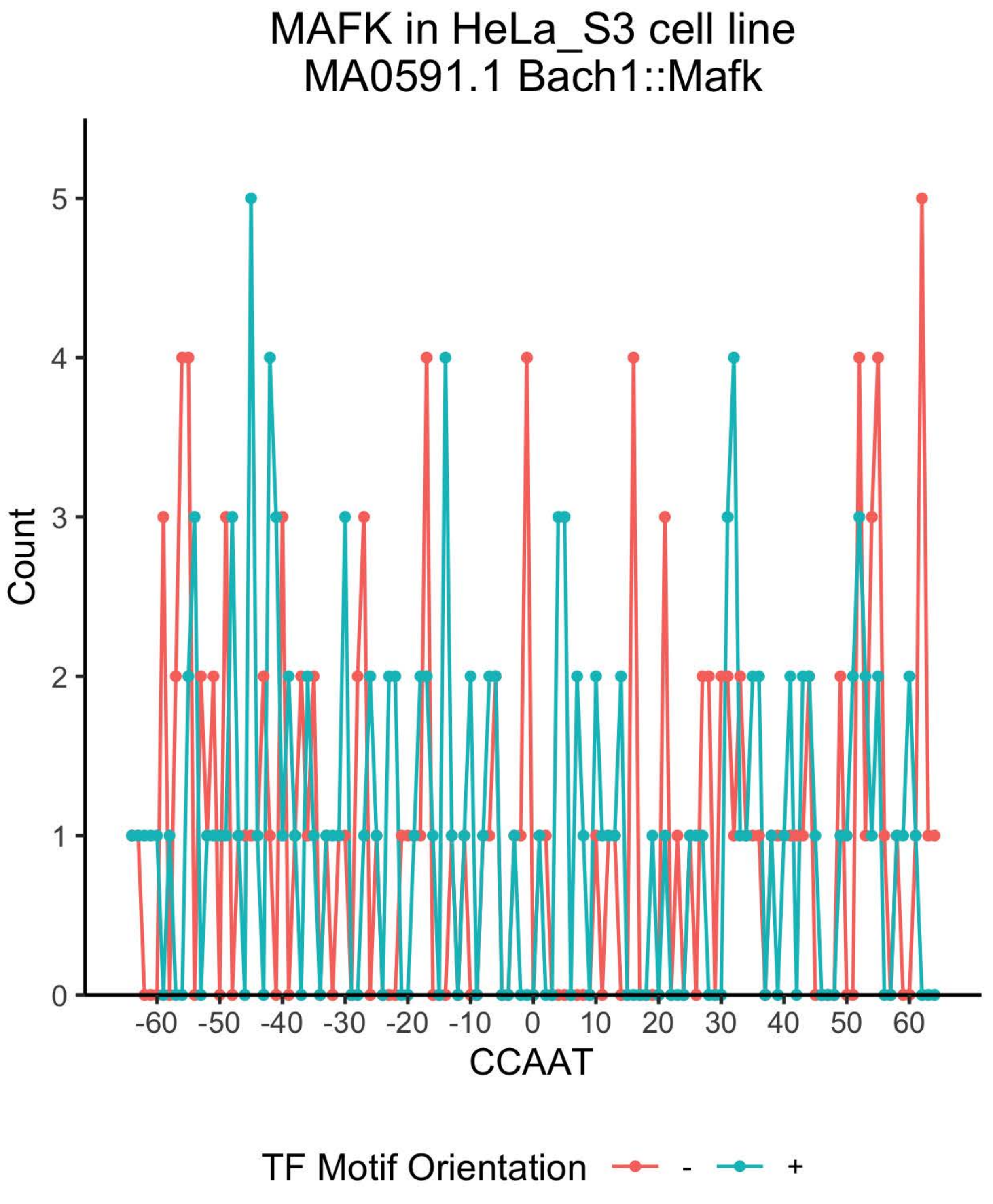

MAFK in HeLa_S3 cell line
MA0591.1 Bach1::Mafk
Count
0
1
2
3
4
5
-60
-50
-40
-30
-20
-10
0
10
20
30
40
50
60
CCAAT
TF Motif Orientation
-
+

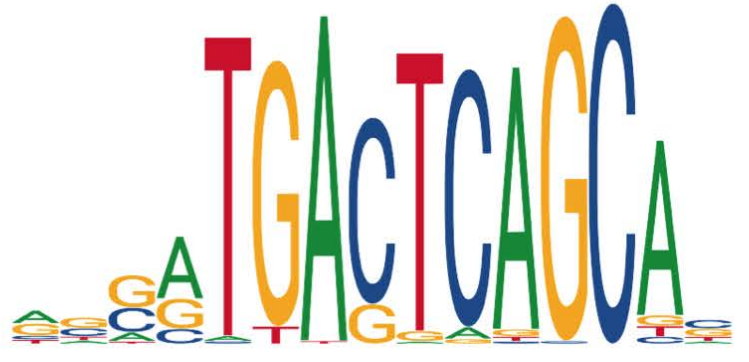

MAFK in K562 cell line
MA0496.2 MAFK

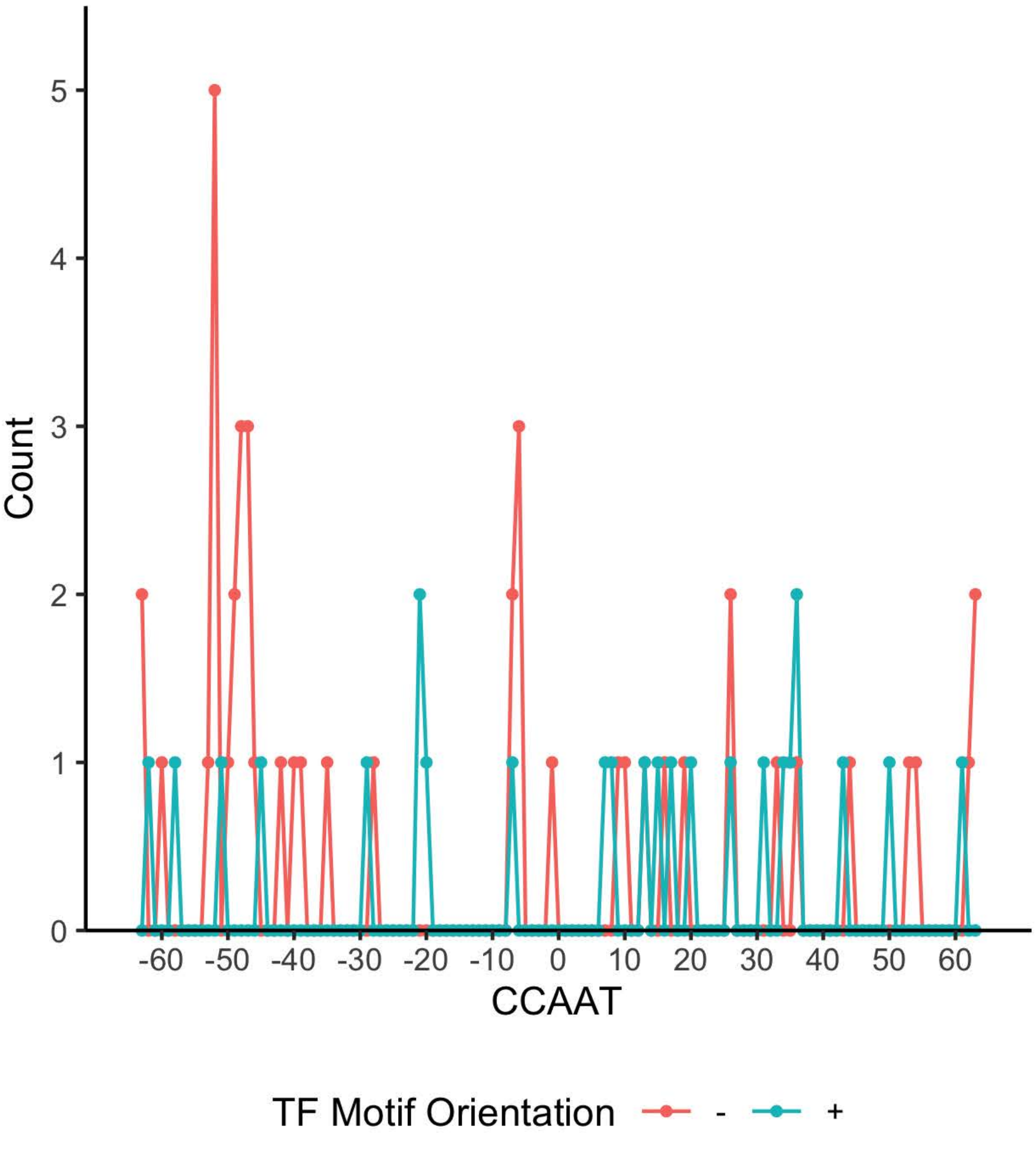

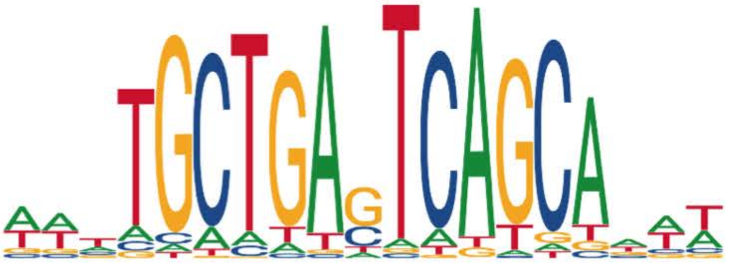

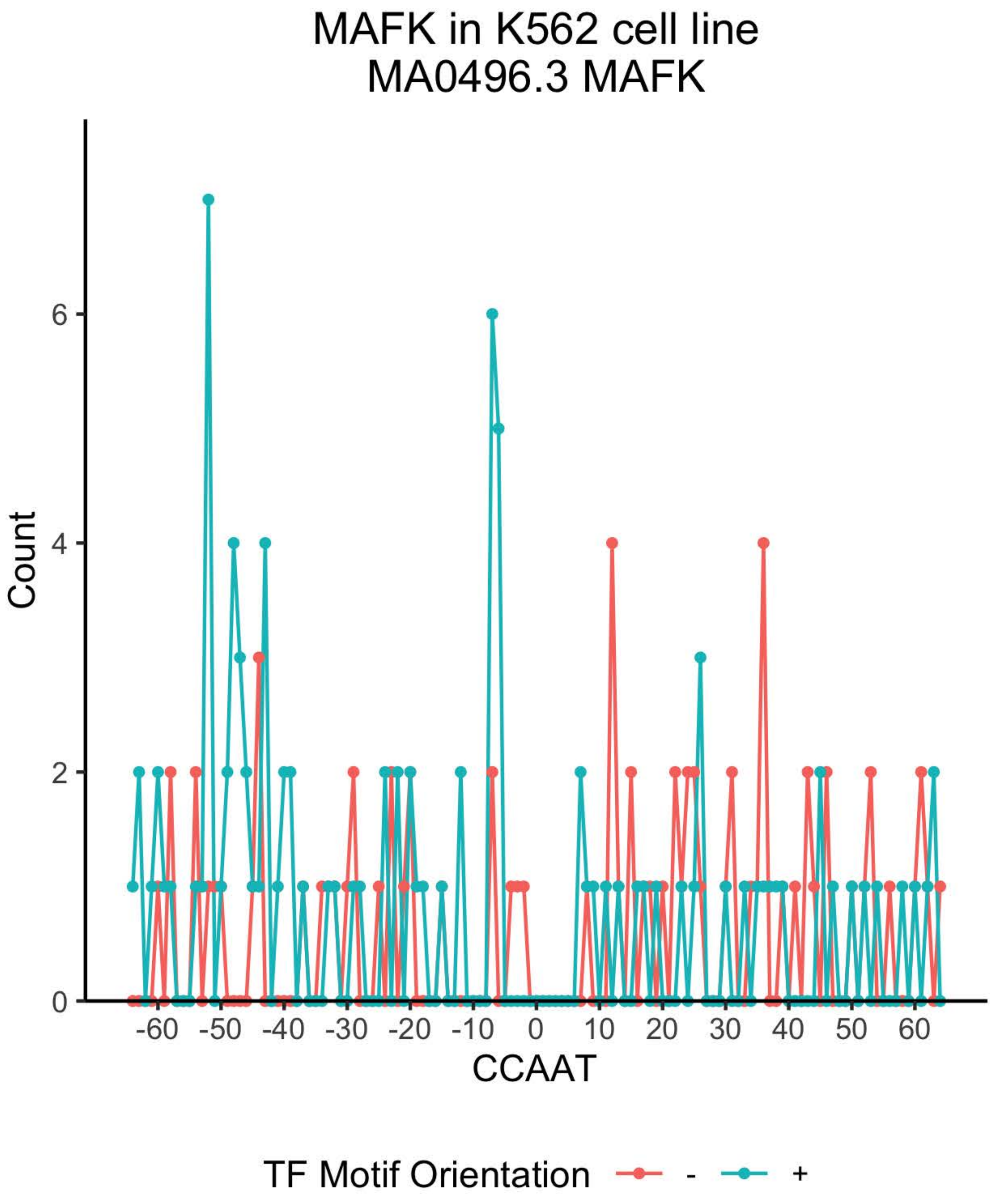
MAFK in K562 cell line
MA0496.3 MAFK
Count
0
2
4
6
-60
-50
-40
-30
-20
-10
0
10
20
30
40
50
60
CCAAT
TF Motif Orientation
-
+

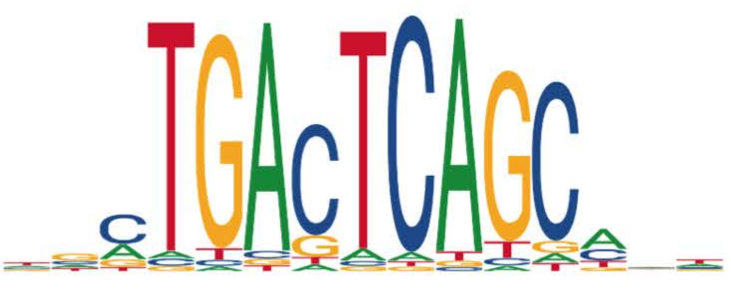

MAFK in K562 cell line
MA0591.1 Bach1::Mafk

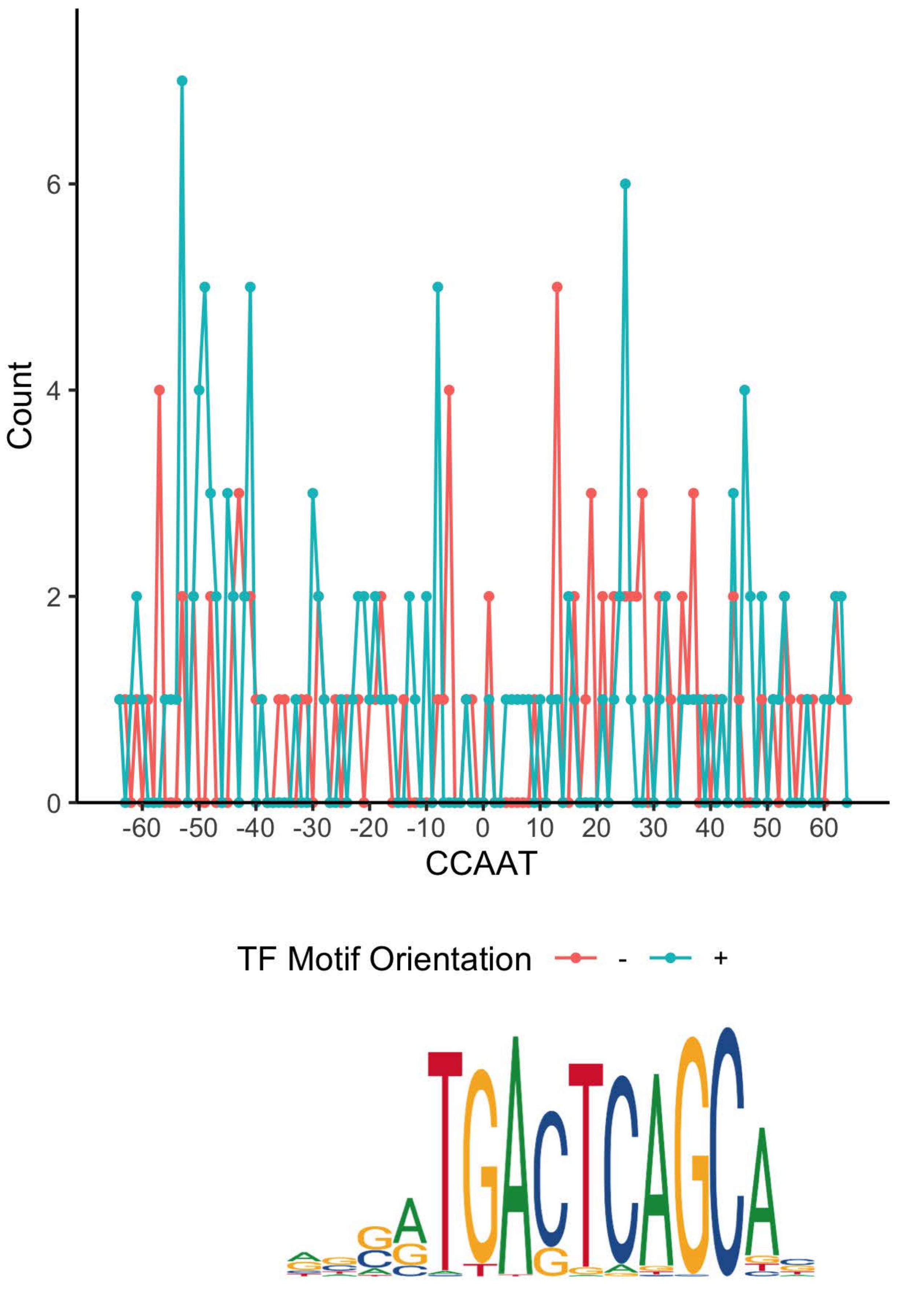

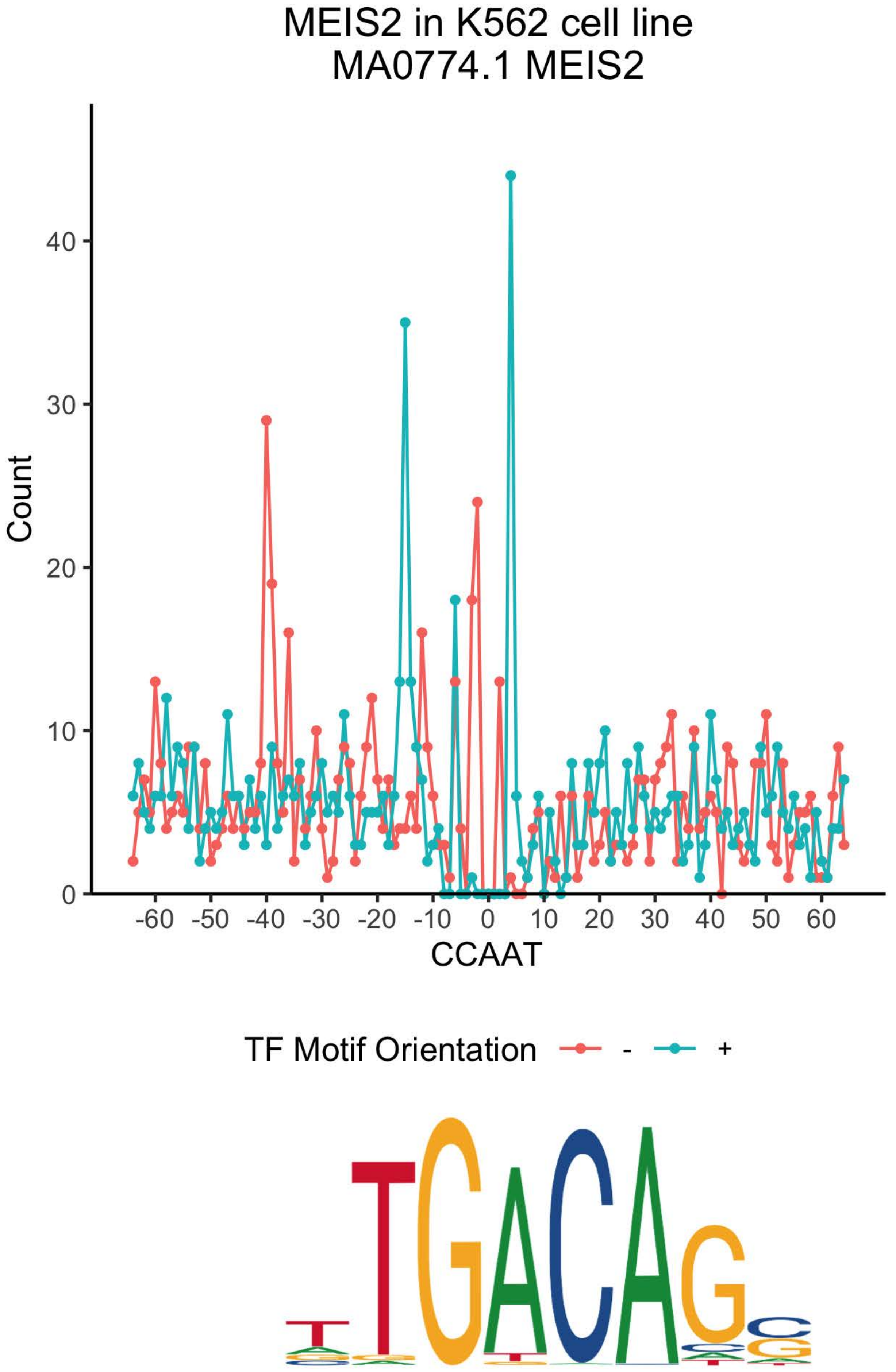

MEIS2 in K562 cell line
MA0774.1 MEIS2
Count
0
10
20
30
40
-60 -50 -40 -30 -20 -10 0 10 20 30 40 50 60
CCAAT
TF Motif Orientation
-
+

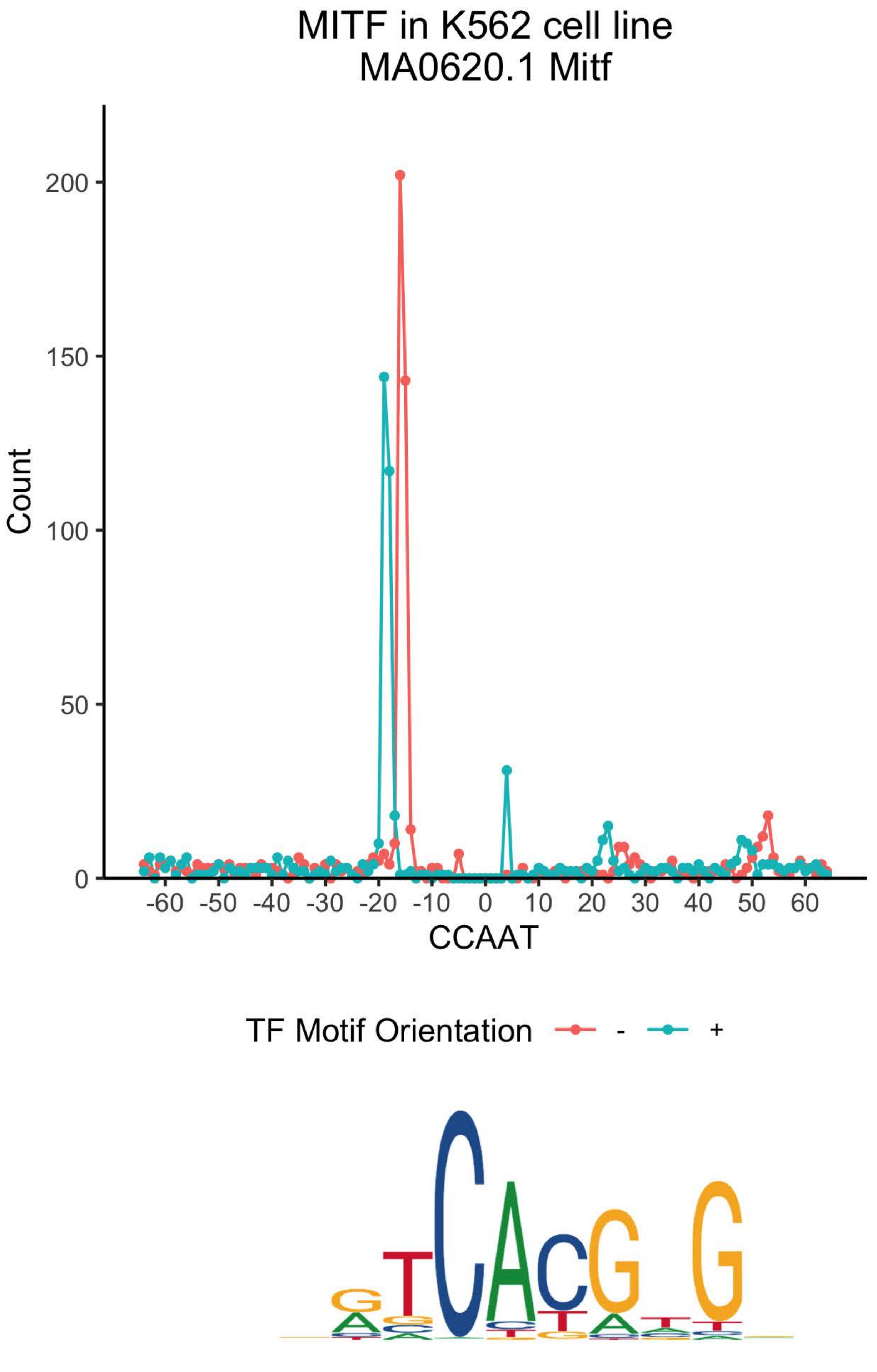
MITF in K562 cell line
MA0620.1 Mitf
Count
200
150
100
50
0
-60
-50
-40
-30
-20
-10
0
10
20
30
40
50
60
CCAAT
TF Motif Orientation
-
+

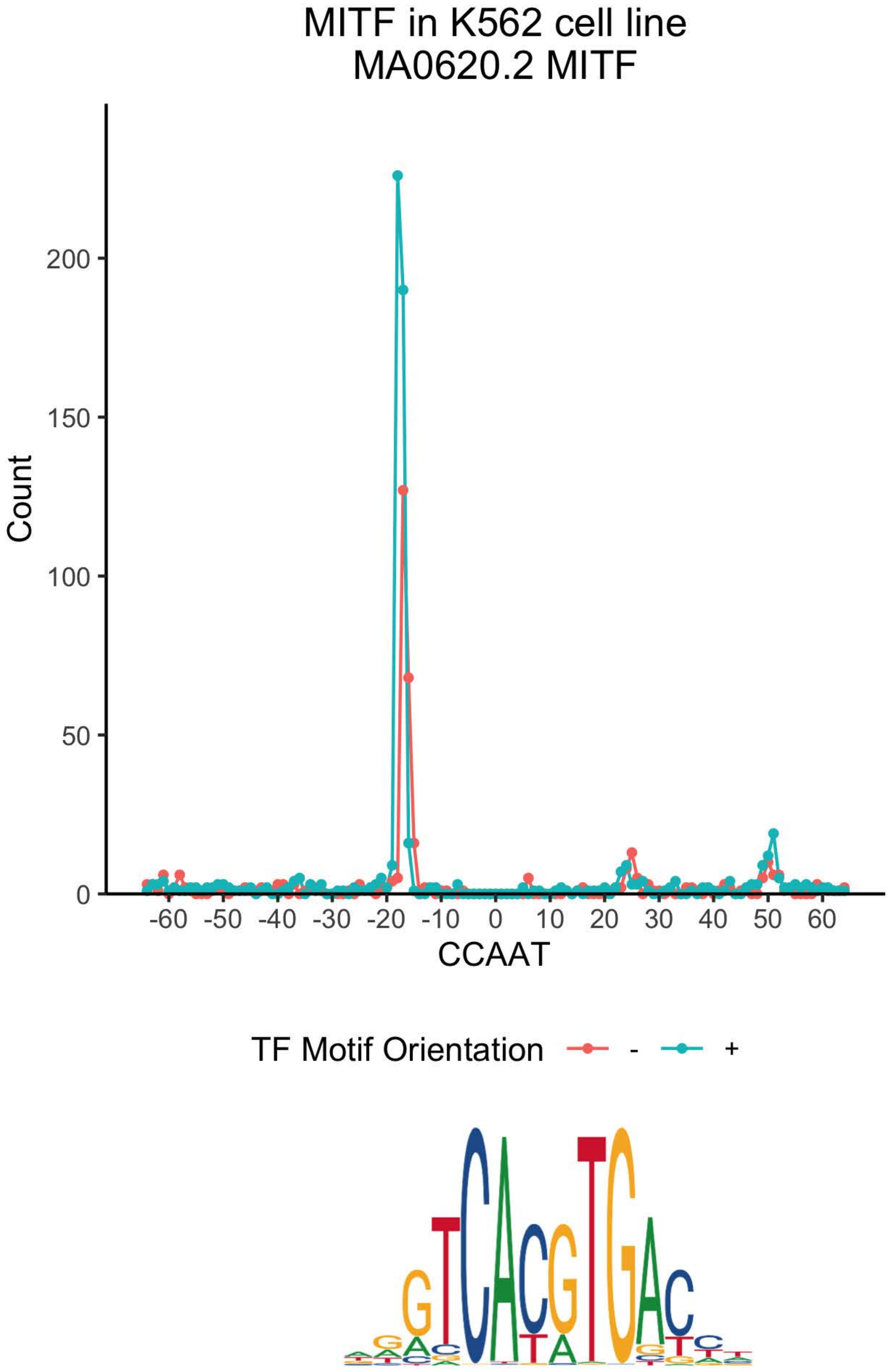
MITF in K562 cell line
MA0620.2 MITF
Count
200
150
100
50
0
-60 -50 -40 -30 -20 -10 0 10 20 30 40 50 60
CCAAT
TF Motif Orientation
-
+

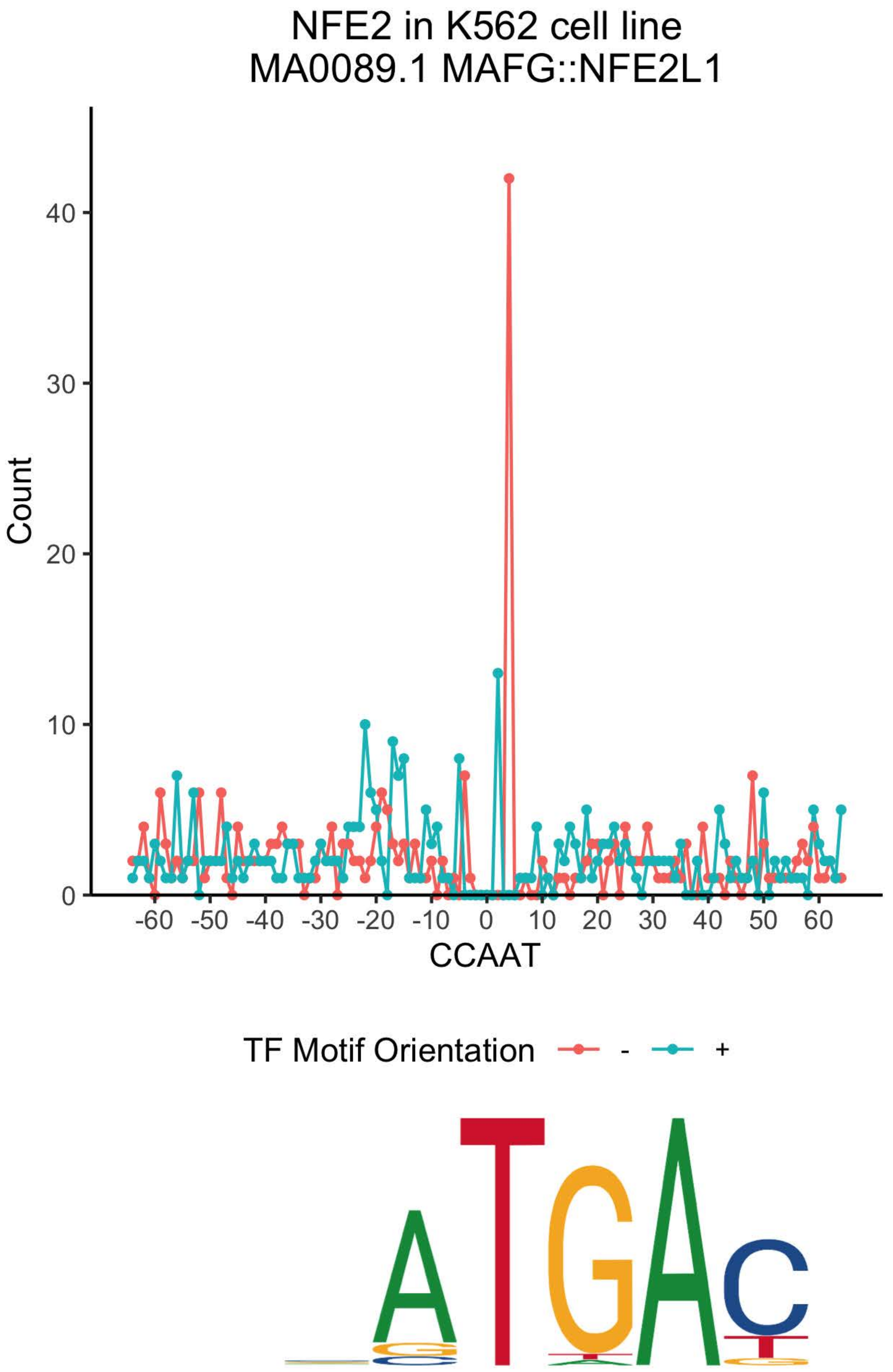
NFE2 in K562 cell line
MA0089.1 MAFG::NFE2L1
Count
40
30
20
10
0
-60 -50 -40 -30 -20 -10 0 10 20 30 40 50 60
CCAAT
TF Motif Orientation - +

NFE2 in K562 cell line
MA0089.2 NFE2L1

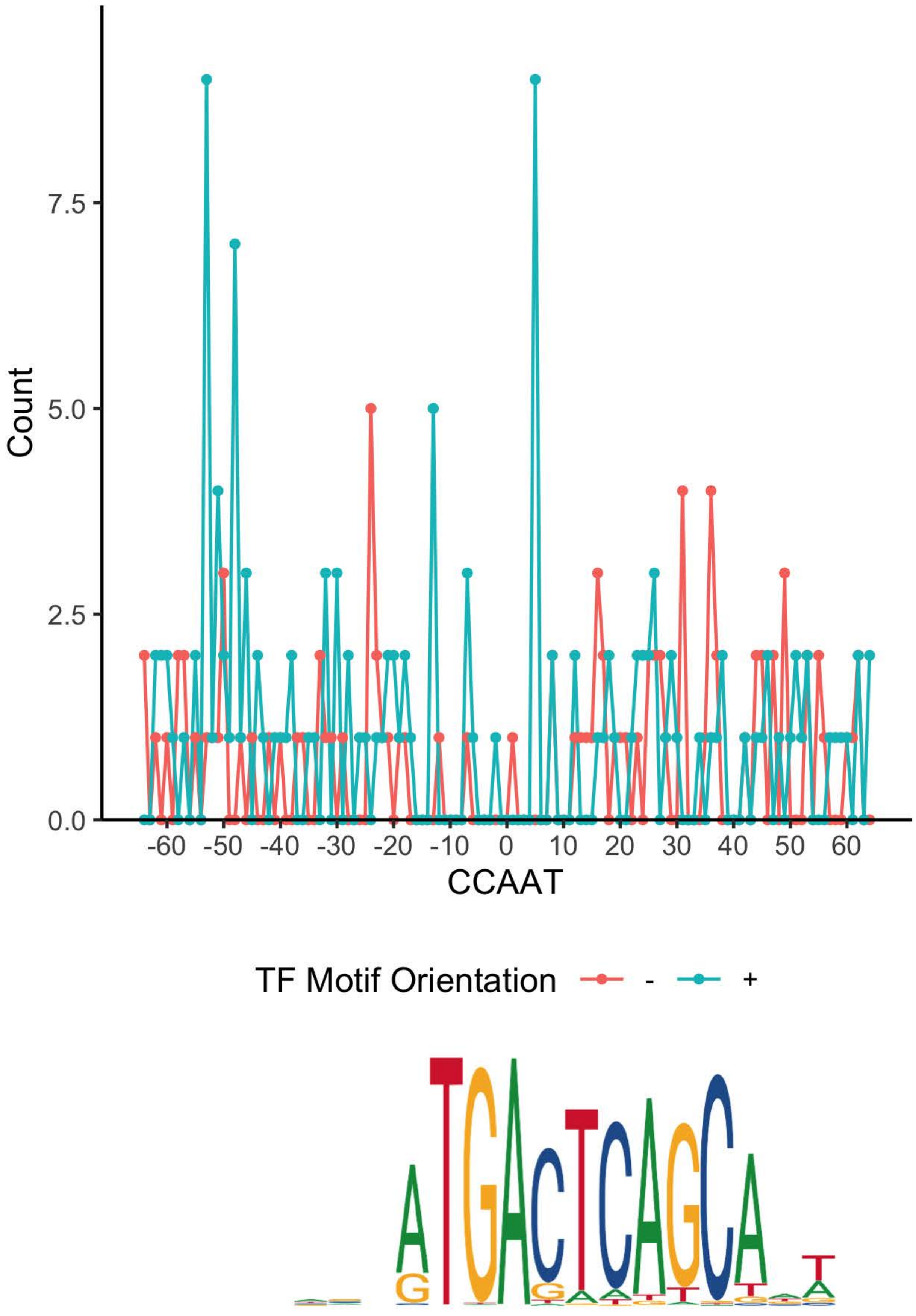

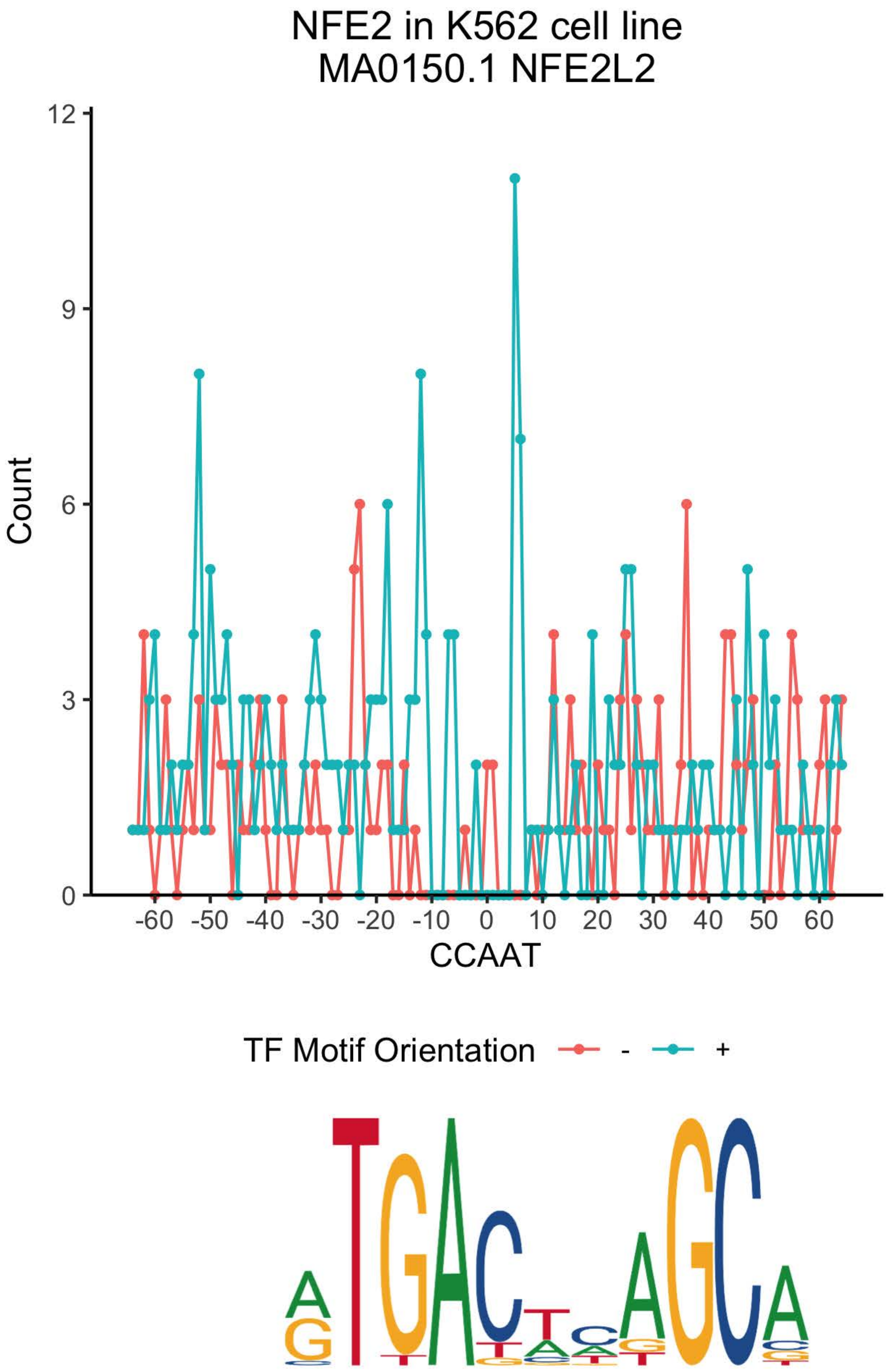

NFE2 in K562 cell line
MA0150.1 NFE2L2
Count
12
9
6
3
0
-60
-50
-40
-30
-20
-10
0
10
20
30
40
50
60
CCAAT
TF Motif Orientation
-
+

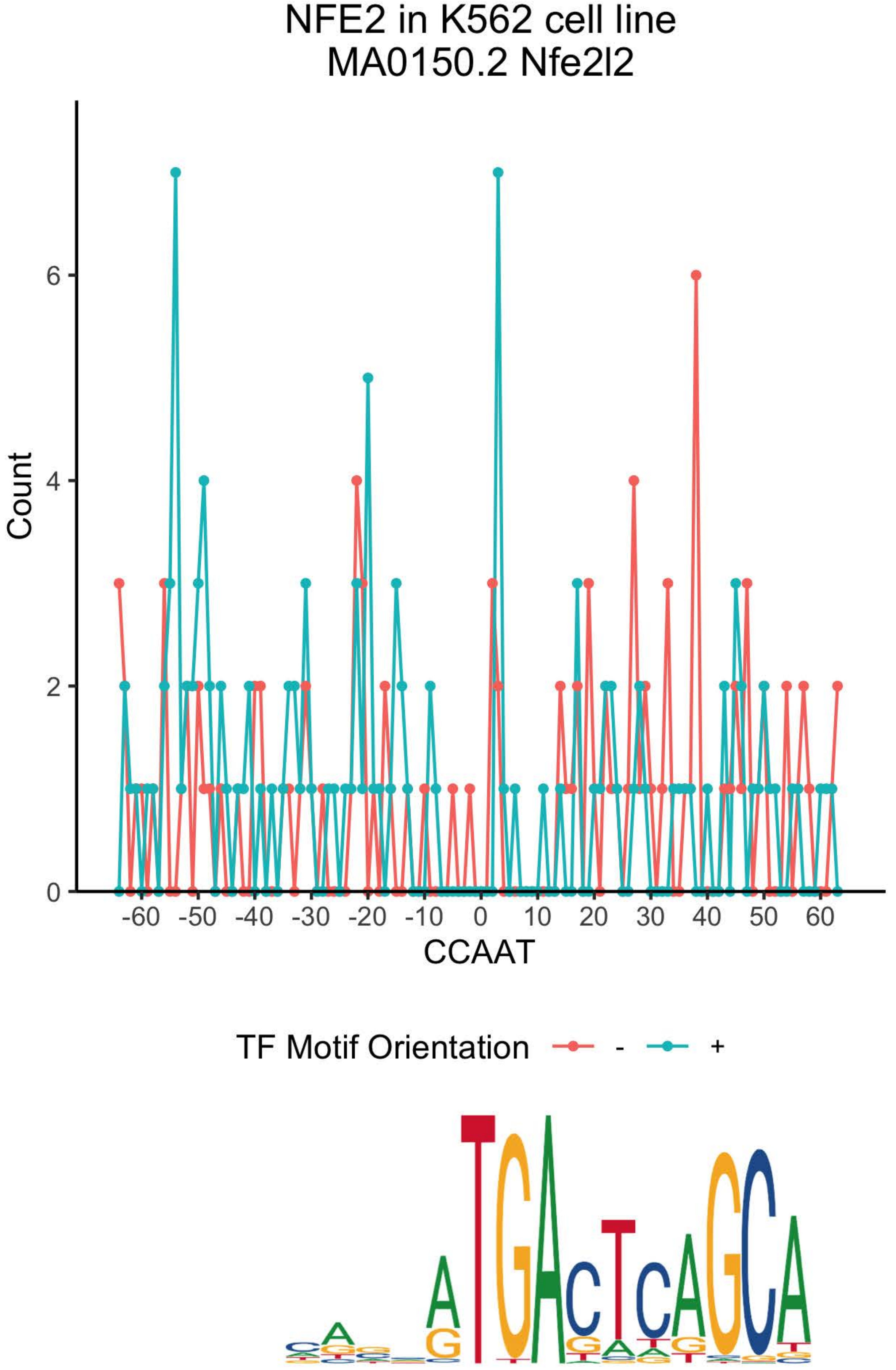
NFE2 in K562 cell line
MA0150.2 Nfe2l2
Count
6
4
2
0
-60 -50 -40 -30 -20 -10 0 10 20 30 40 50 60
CCAAT
TF Motif Orientation - +

NFE2 in K562 cell line
MA0501.1 MAF::NFE2

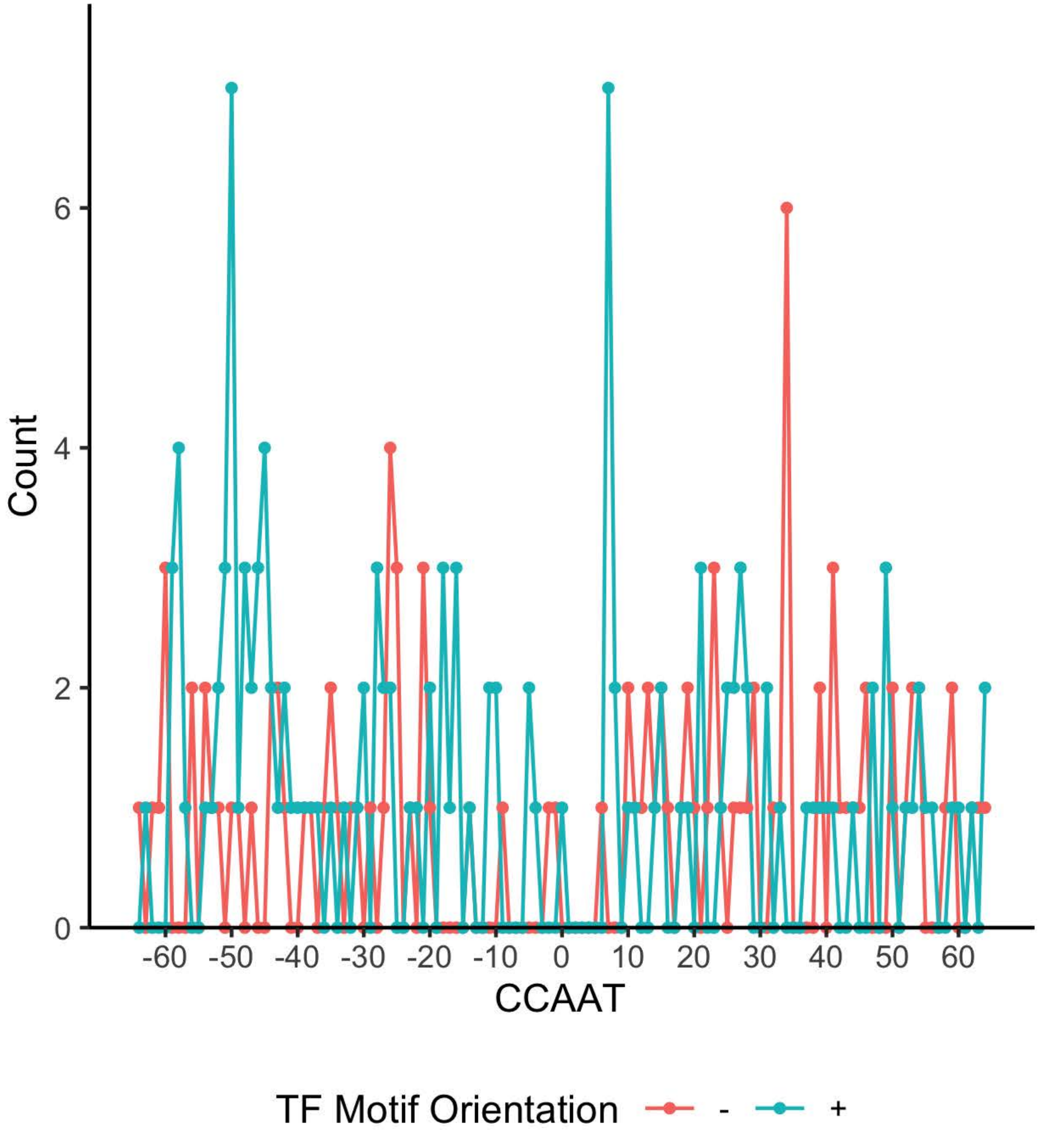

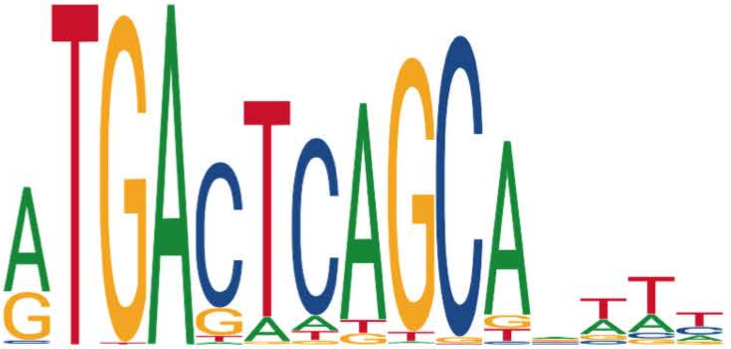

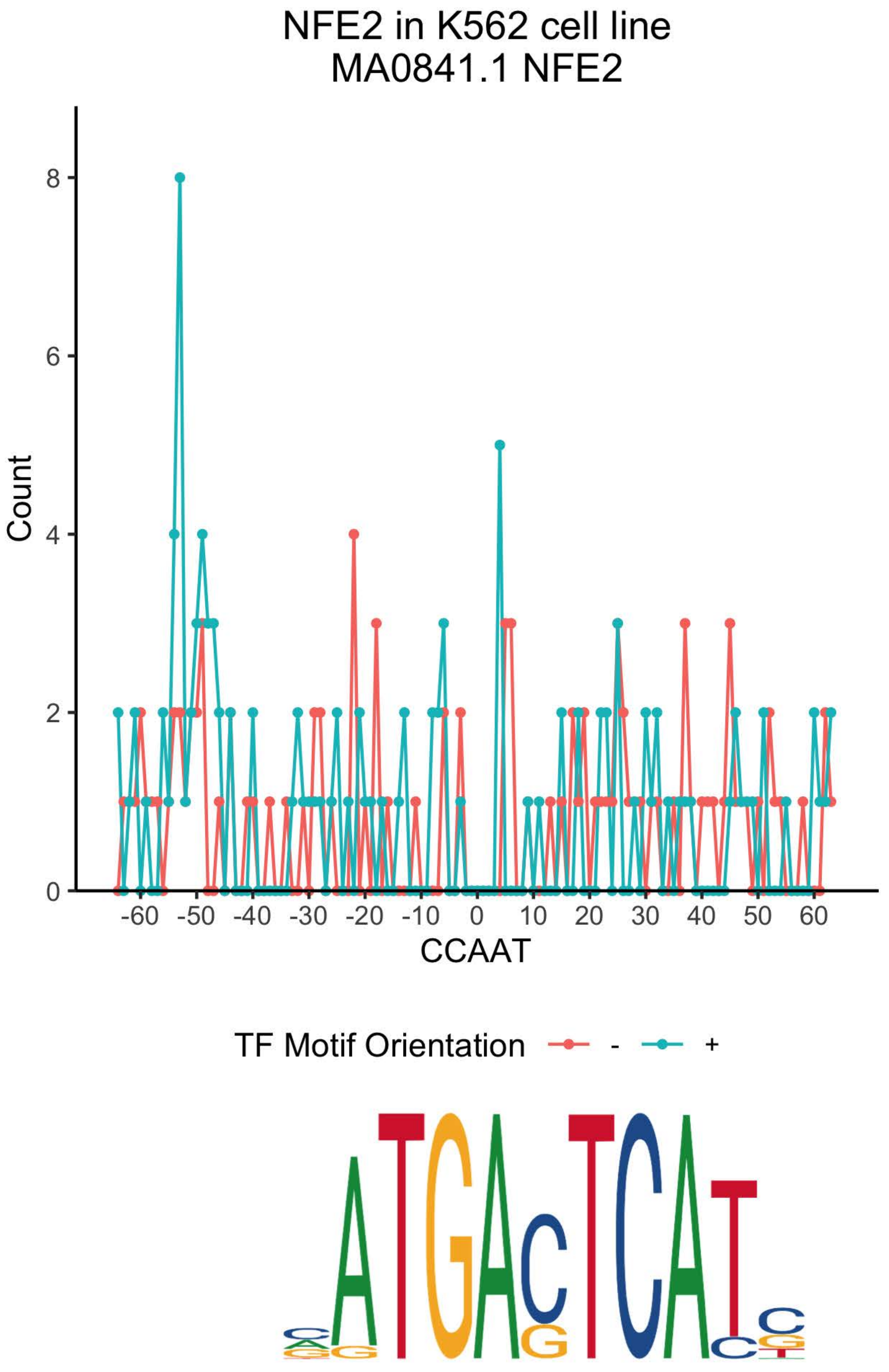
NFE2 in K562 cell line
MA0841.1 NFE2
Count
8
6
4
2
0
-60 -50 -40 -30 -20 -10 0 10 20 30 40 50 60
CCAAT
TF Motif Orientation
-
+

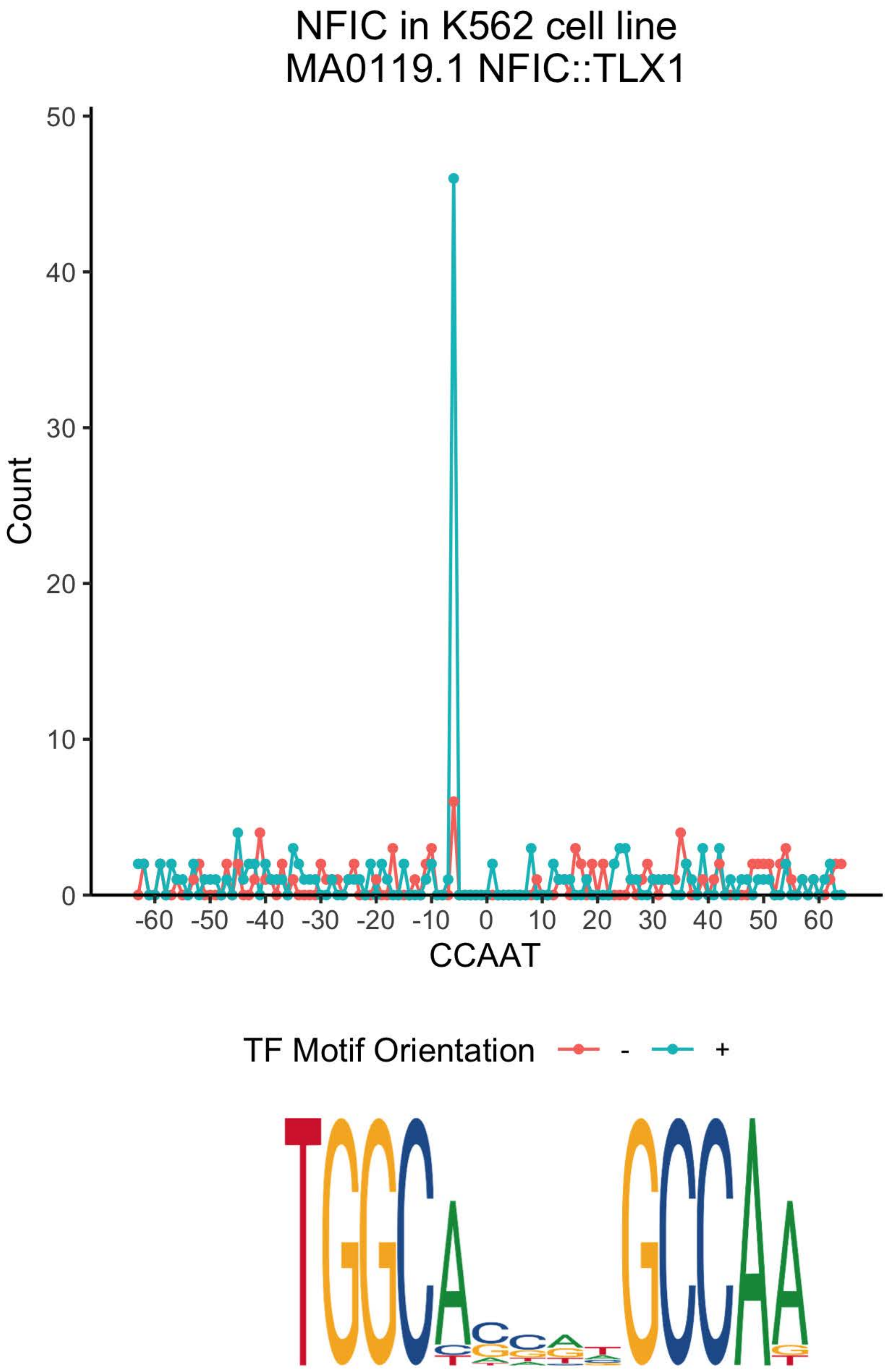

NFIC in K562 cell line
MA0119.1 NFIC::TLX1
Count
50
40
30
20
10
0
-60 -50 -40 -30 -20 -10 0 10 20 30 40 50 60
CCAAT
TF Motif Orientation
-
+

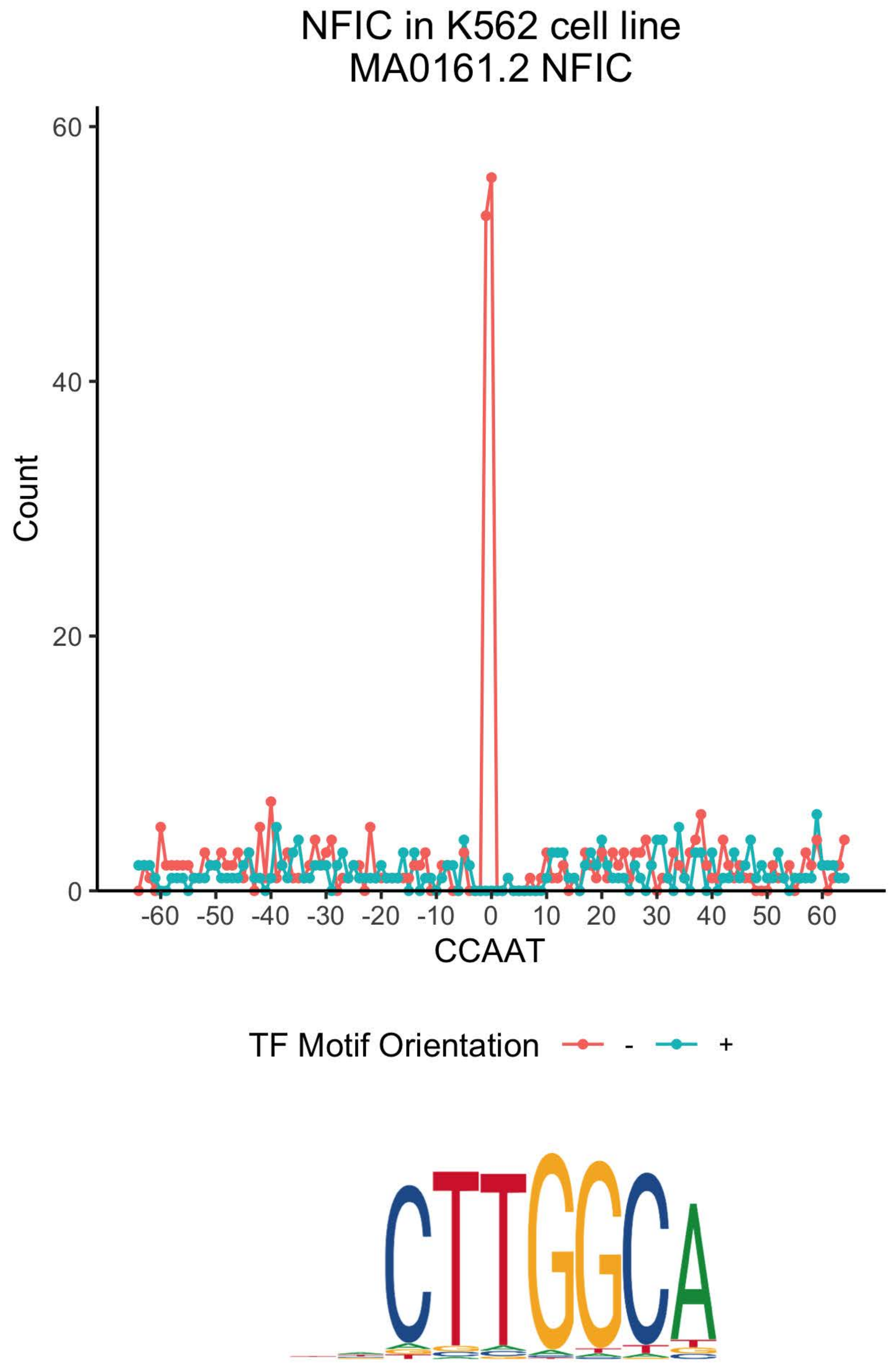
NFIC in K562 cell line
MA0161.2 NFIC
Count
0
20
40
60
-60 -50 -40 -30 -20 -10 0 10 20 30 40 50 60
CCAAT
TF Motif Orientation
-
+

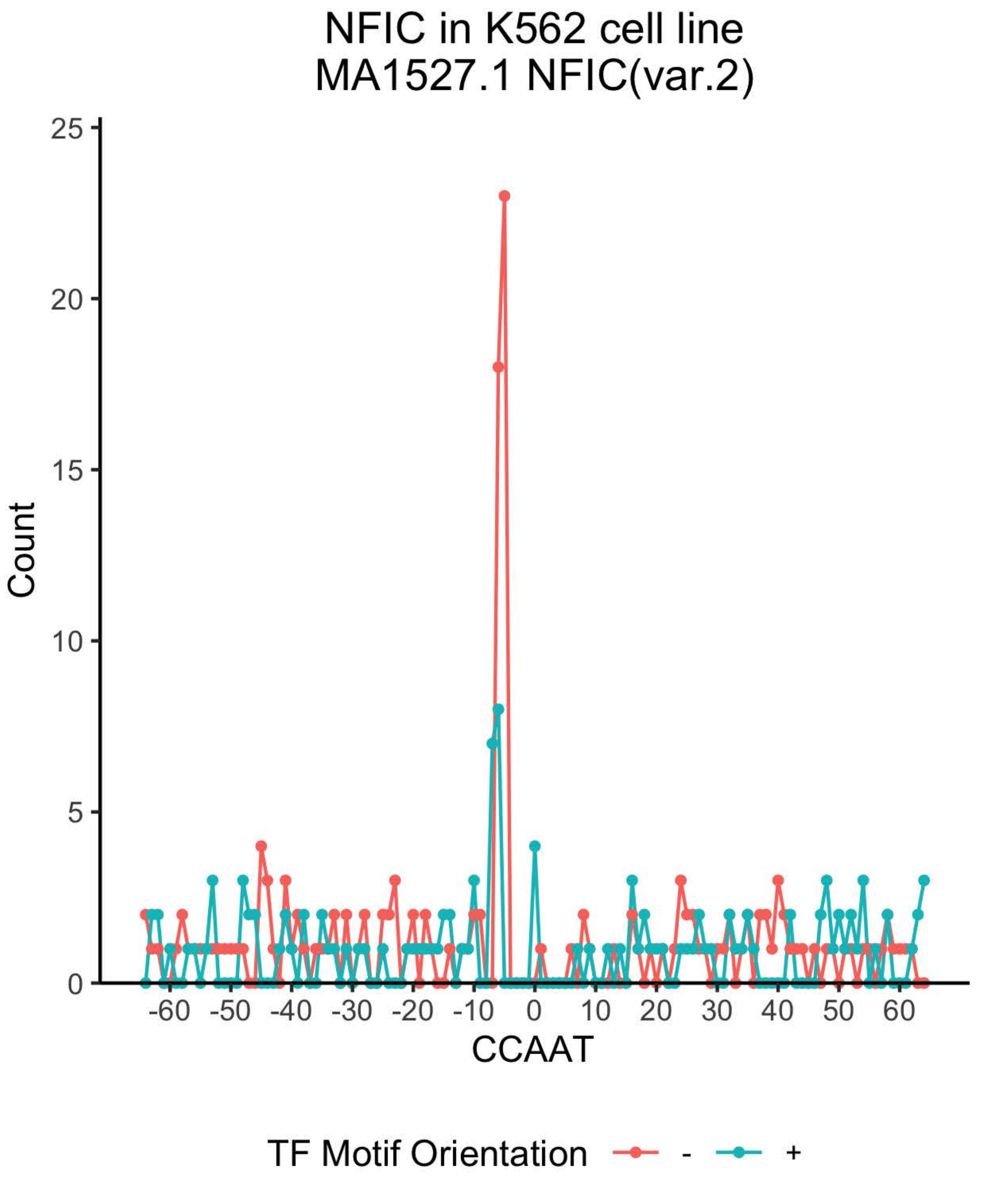

NFIC in K562 cell line
MA1527.1 NFIC(var.2)
Count
25
20
15
10
5
0
-60 -50 -40 -30 -20 -10 0 10 20 30 40 50 60
CCAAT
TF Motif Orientation
-
+

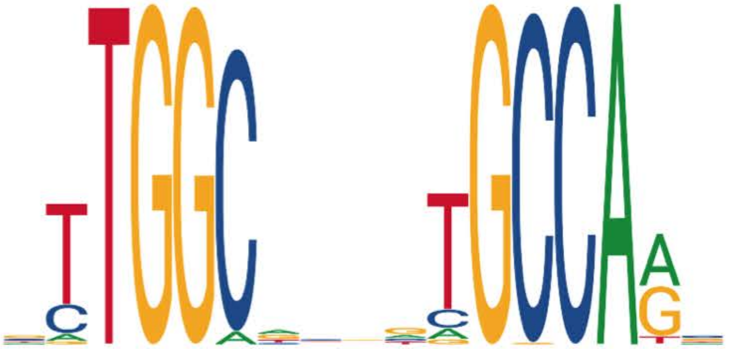

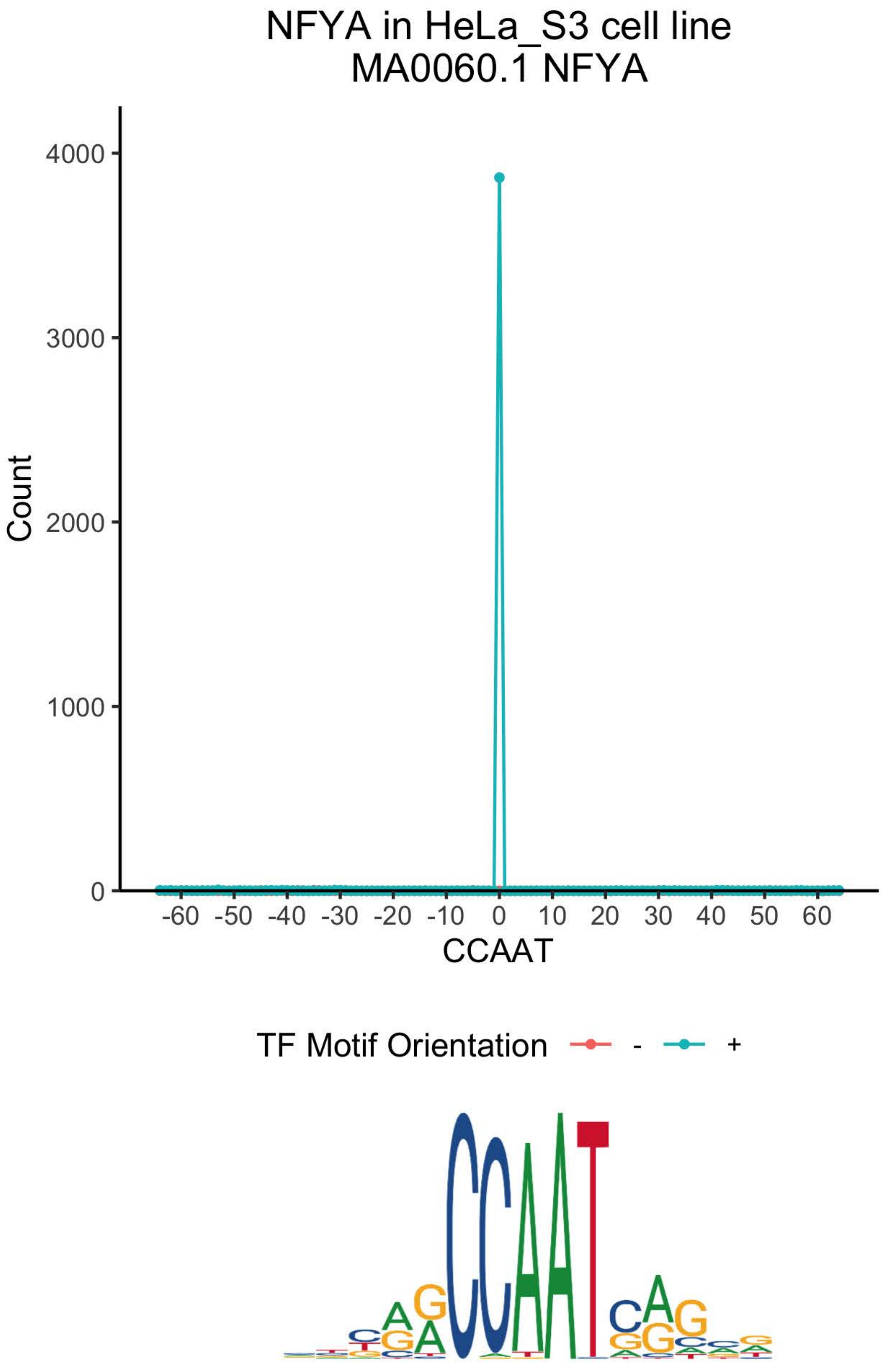

NFYA in HeLa_S3 cell line
MA0060.1 NFYA
Count
4000
3000
2000
1000
0
-60 -50 -40 -30 -20 -10 0 10 20 30 40 50 60
CCAAT
TF Motif Orientation
-
+

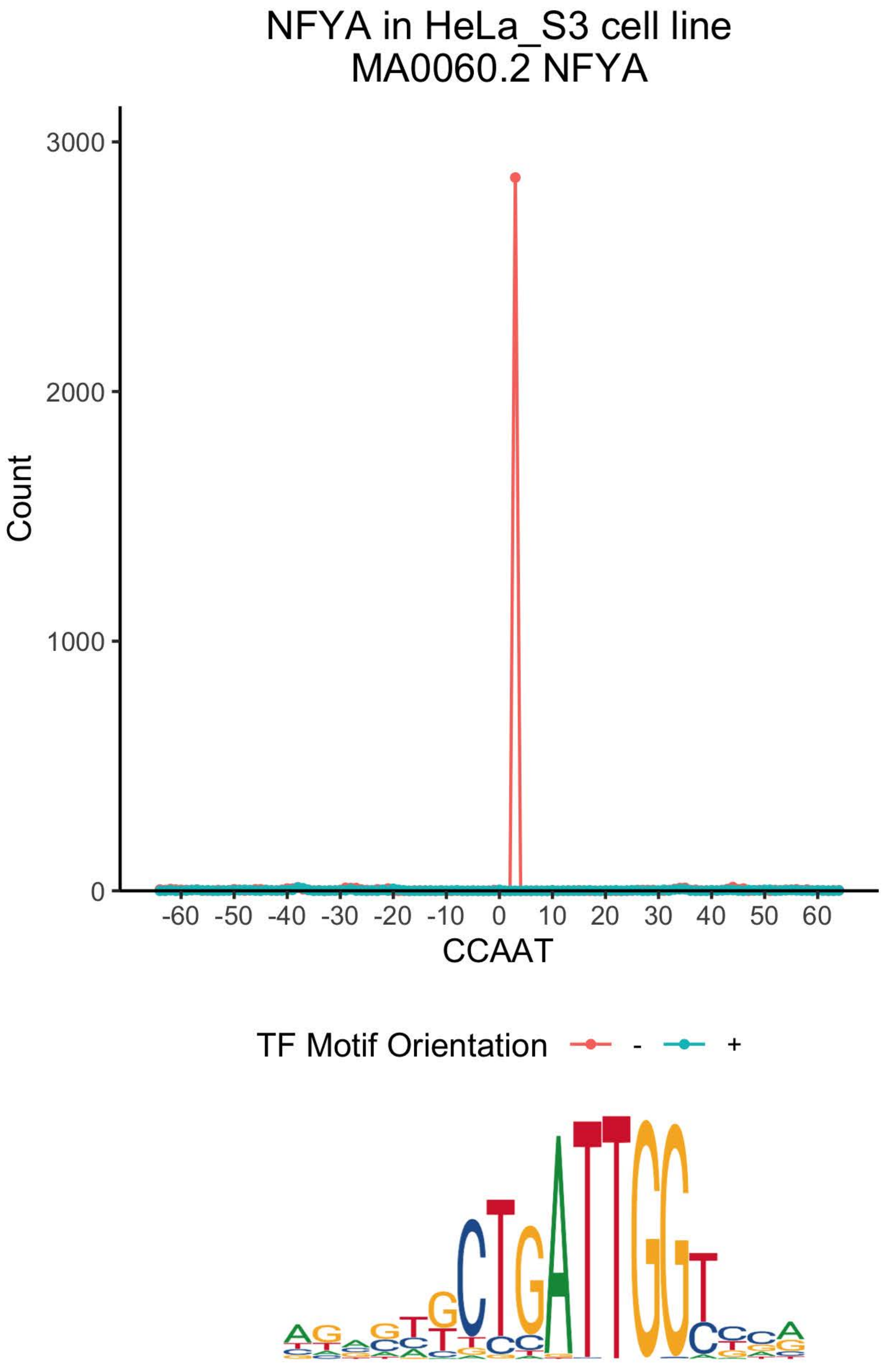
NFYA in HeLa_S3 cell line
MA0060.2 NFYA
Count
3000
2000
1000
0
-60 -50 -40 -30 -20 -10 0 10 20 30 40 50 60
CCAAT
TF Motif Orientation - +

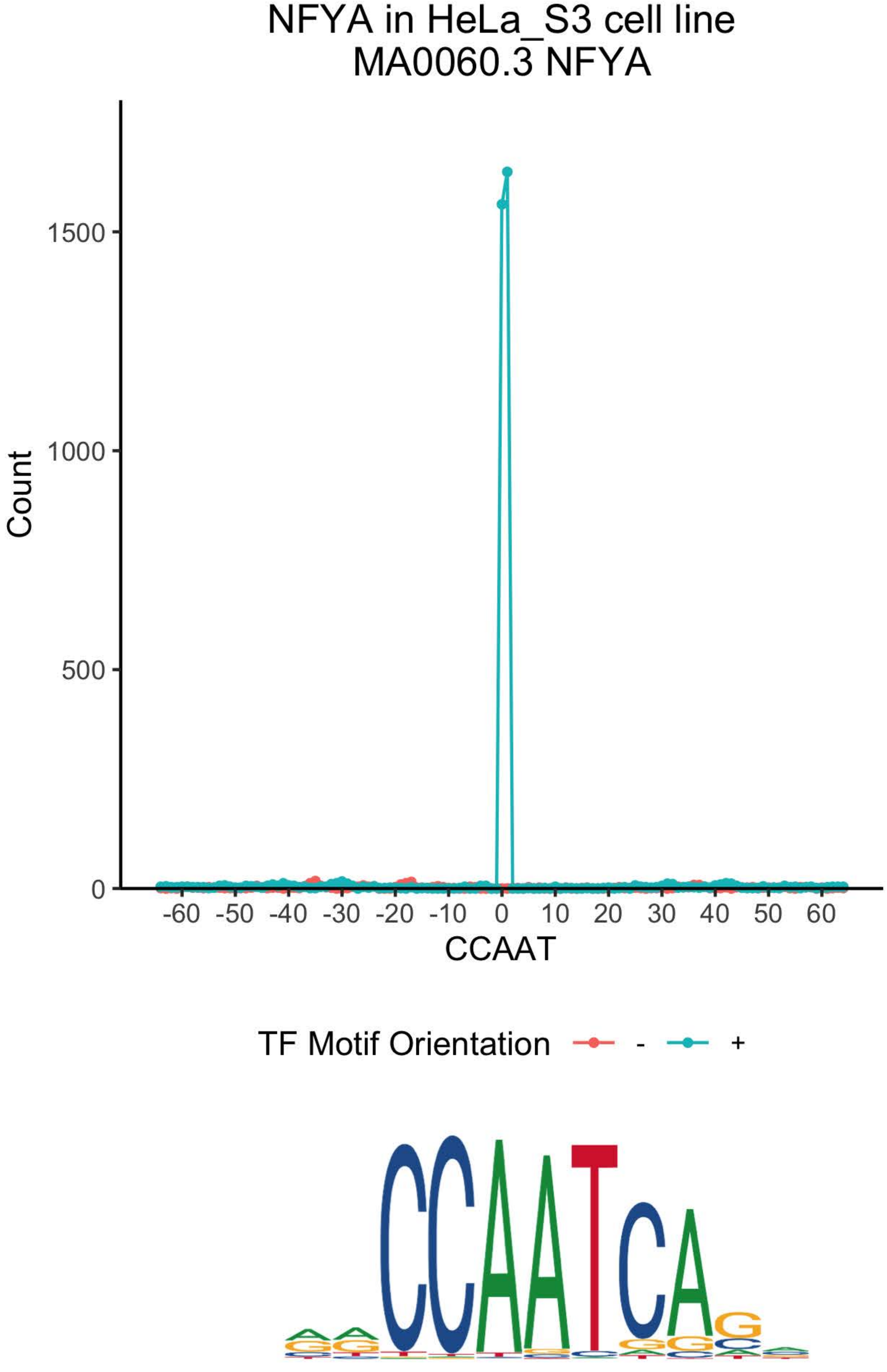

NFYA in HeLa_S3 cell line
MA0060.3 NFYA
Count
1500
1000
500
0
-60 -50 -40 -30 -20 -10 0 10 20 30 40 50 60
CCAAT
TF Motif Orientation
-
+

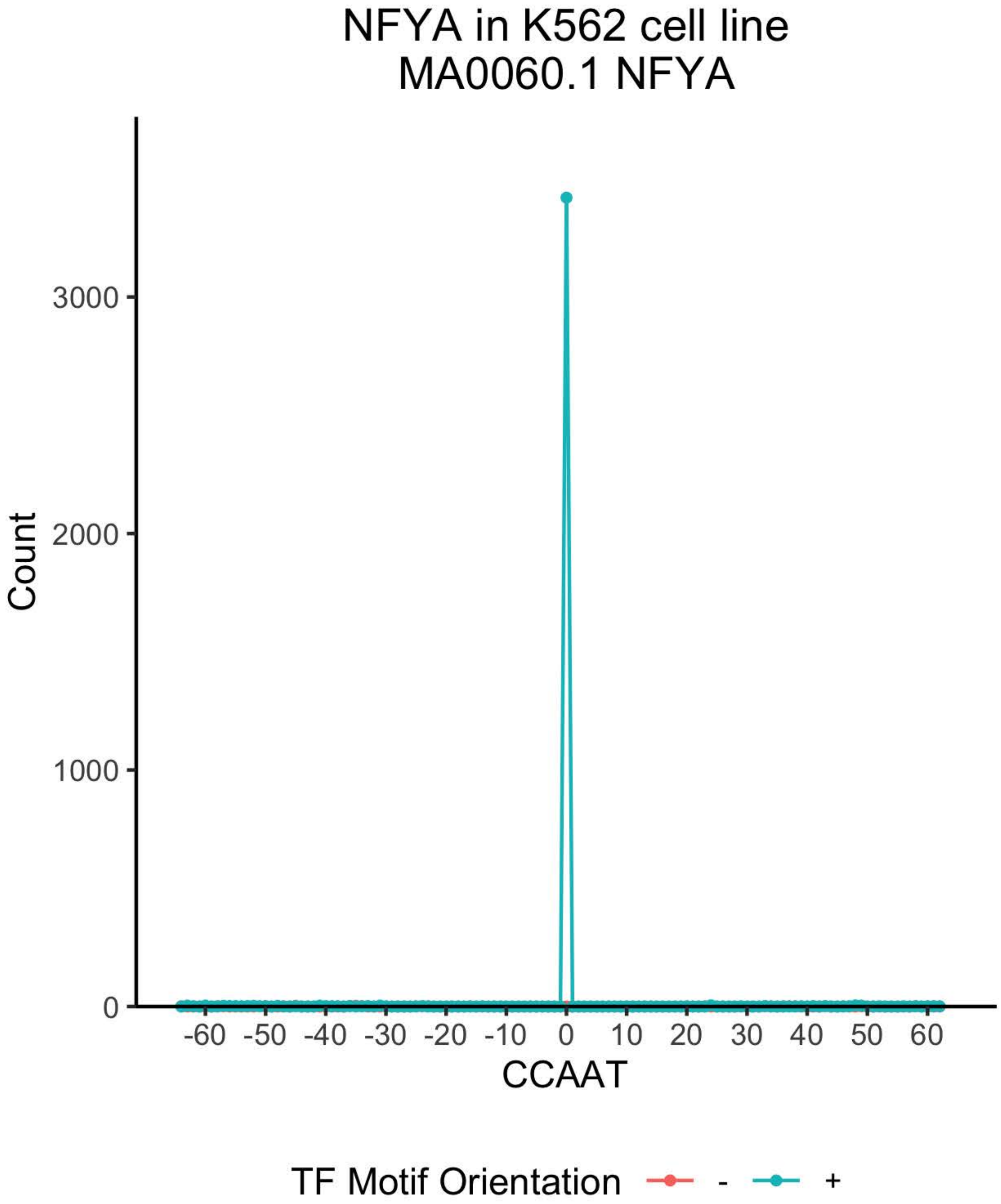

NFYA in K562 cell line
MA0060.1 NFYA
Count
3000
2000
1000
0
-60 -50 -40 -30 -20 -10 0 10 20 30 40 50 60
CCAAT
TF Motif Orientation - +

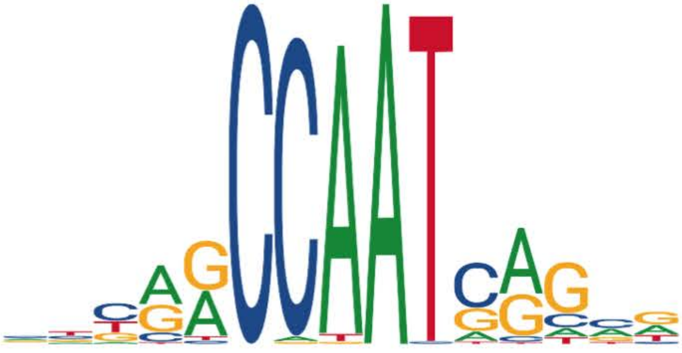

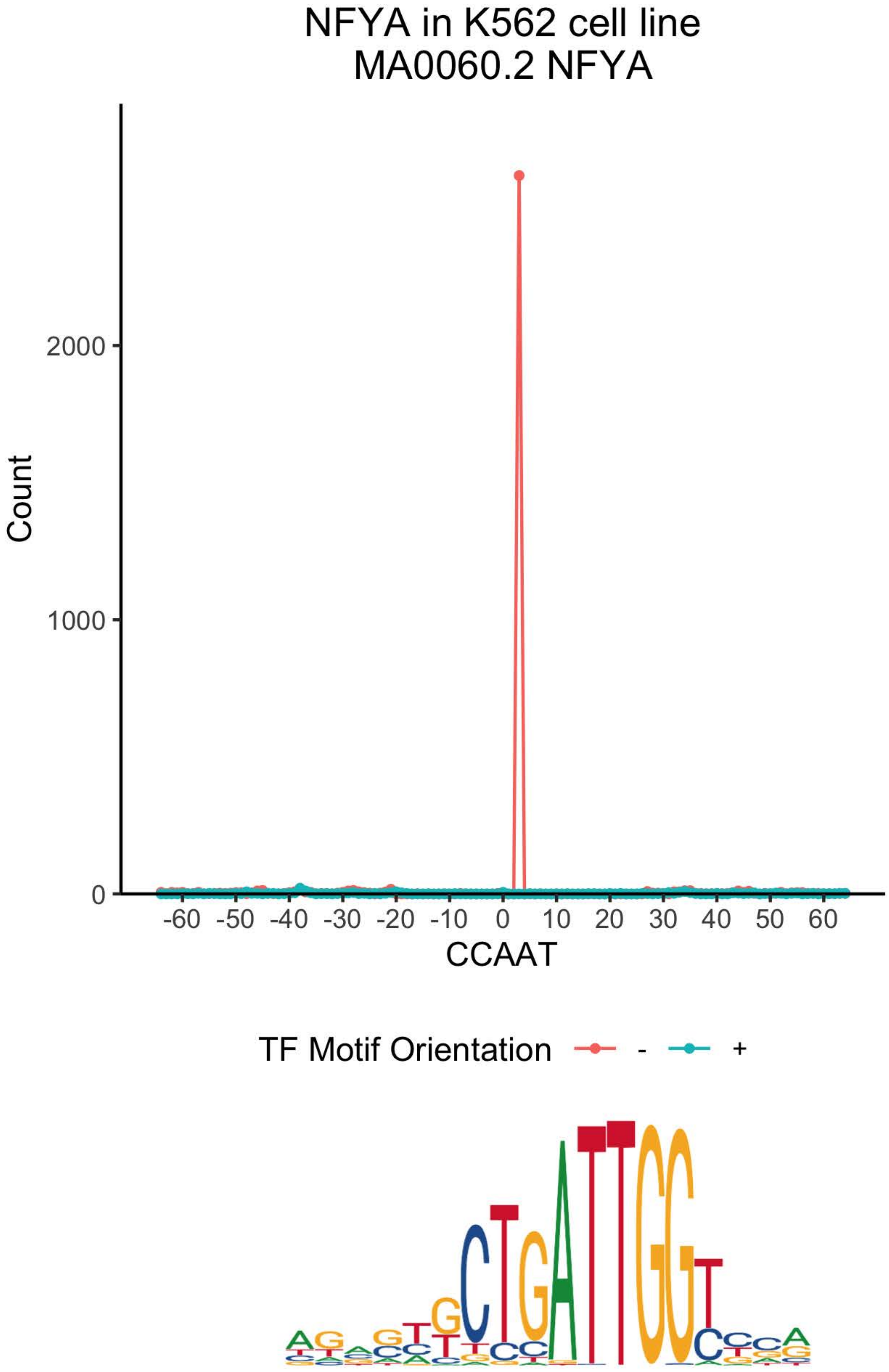
NFYA in K562 cell line
MA0060.2 NFYA
Count
2000
1000
0
-60 -50 -40 -30 -20 -10 0 10 20 30 40 50 60
CCAAT
TF Motif Orientation
-
+

NFYA in K562 cell line
MA0060.3 NFYA

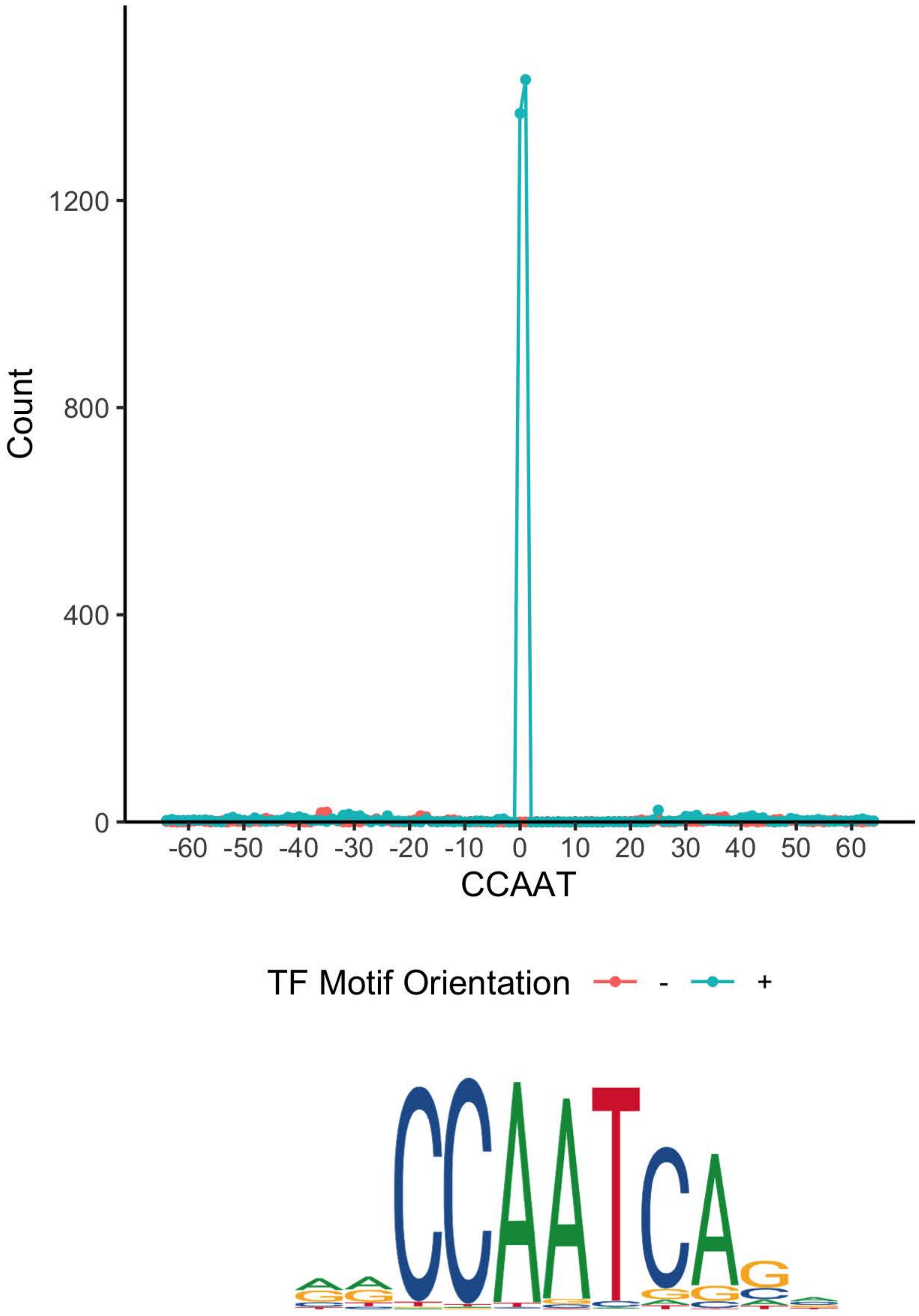

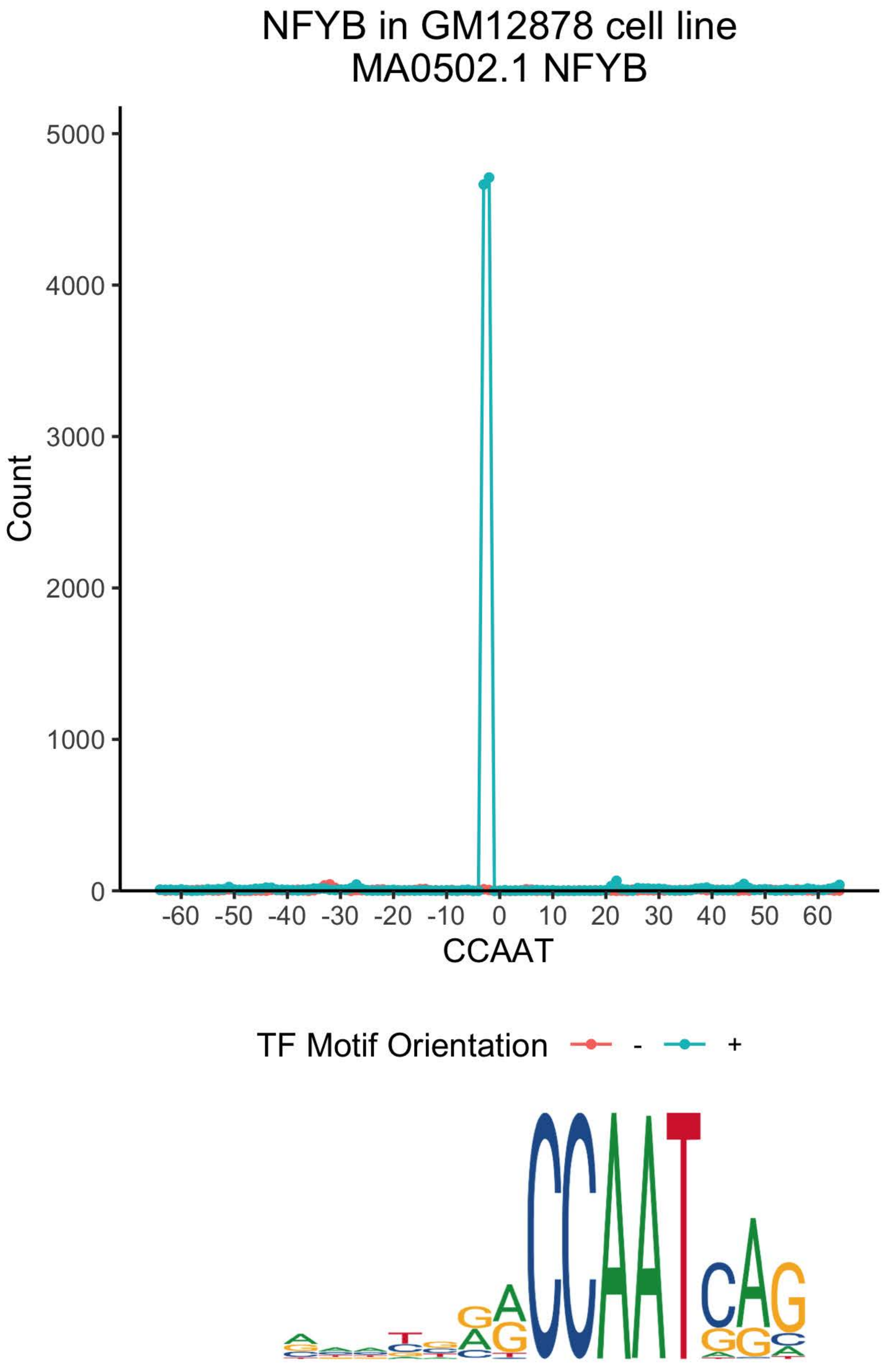
NFYB in GM12878 cell line
MA0502.1 NFYB
Count
5000
4000
3000
2000
1000
0
-60 -50 -40 -30 -20 -10 0 10 20 30 40 50 60
CCAAT
TF Motif Orientation
-
+

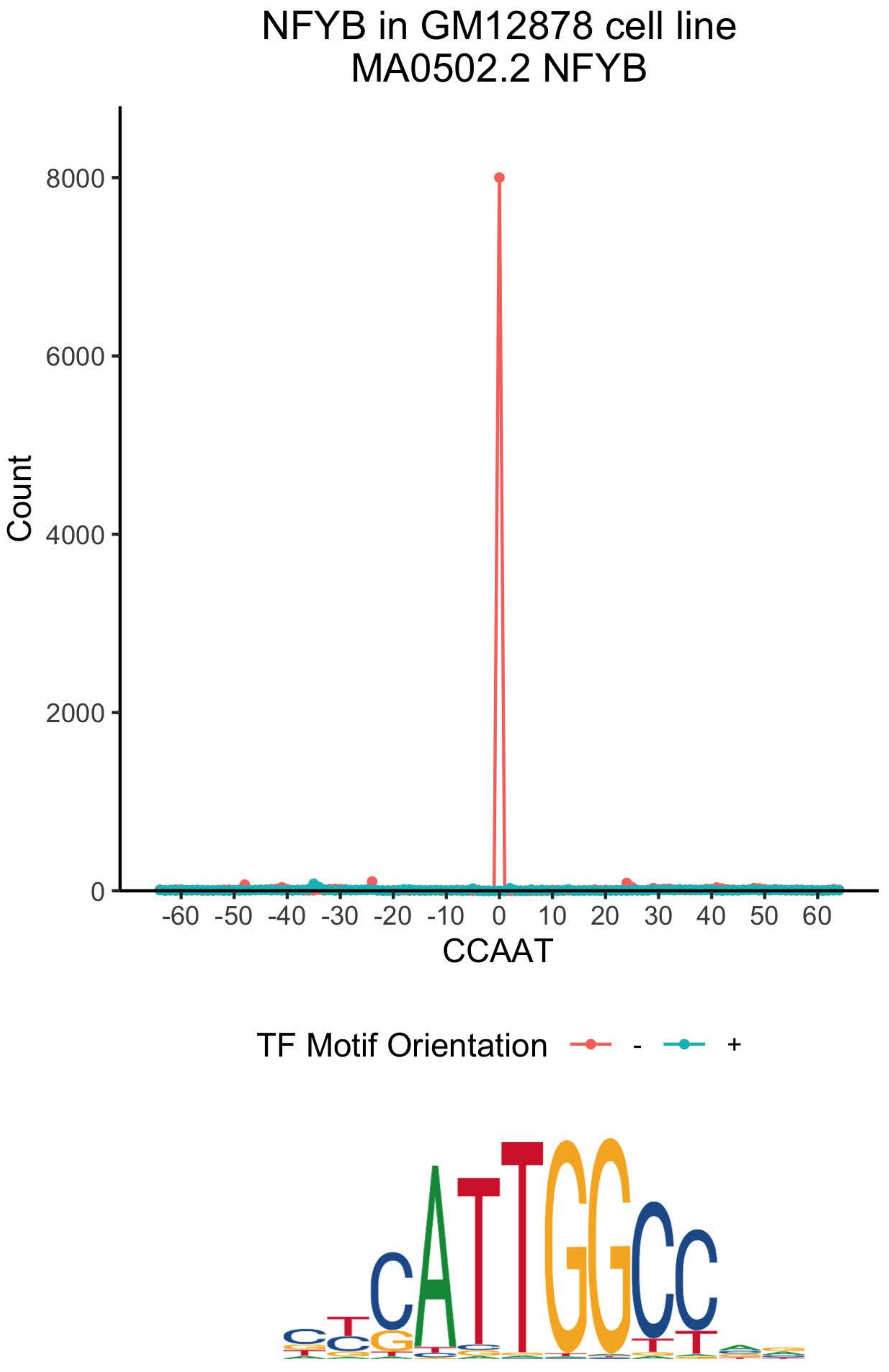
NFYB in GM12878 cell line
MA0502.2 NFYB
Count
8000
6000
4000
2000
0
-60 -50 -40 -30 -20 -10 0 10 20 30 40 50 60
CCAAT
TF Motif Orientation
-
+

NFYB in HeLa_S3 cell line
MA0502.1 NFYB

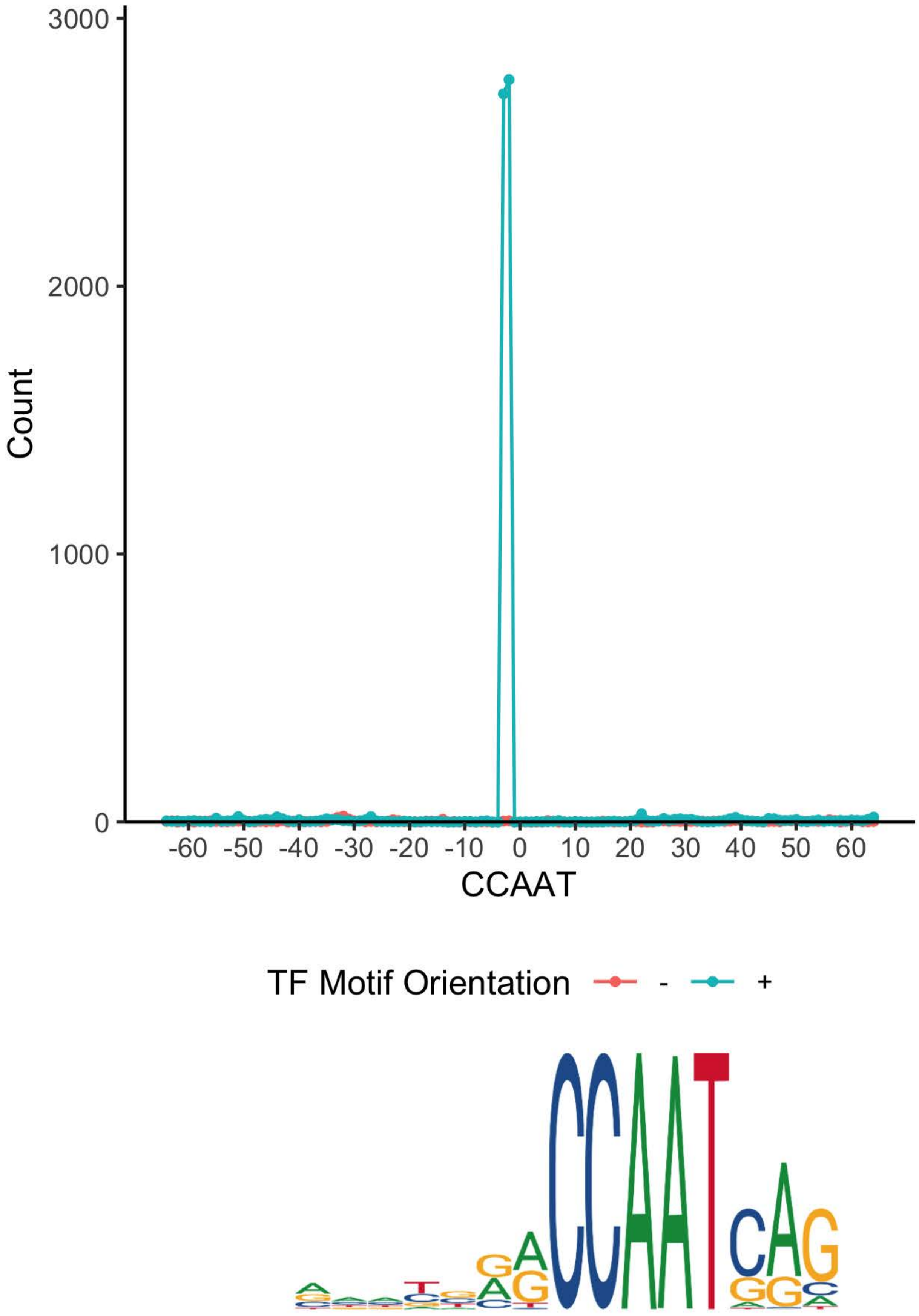

NFYB in HeLa_S3 cell line
MA0502.2 NFYB

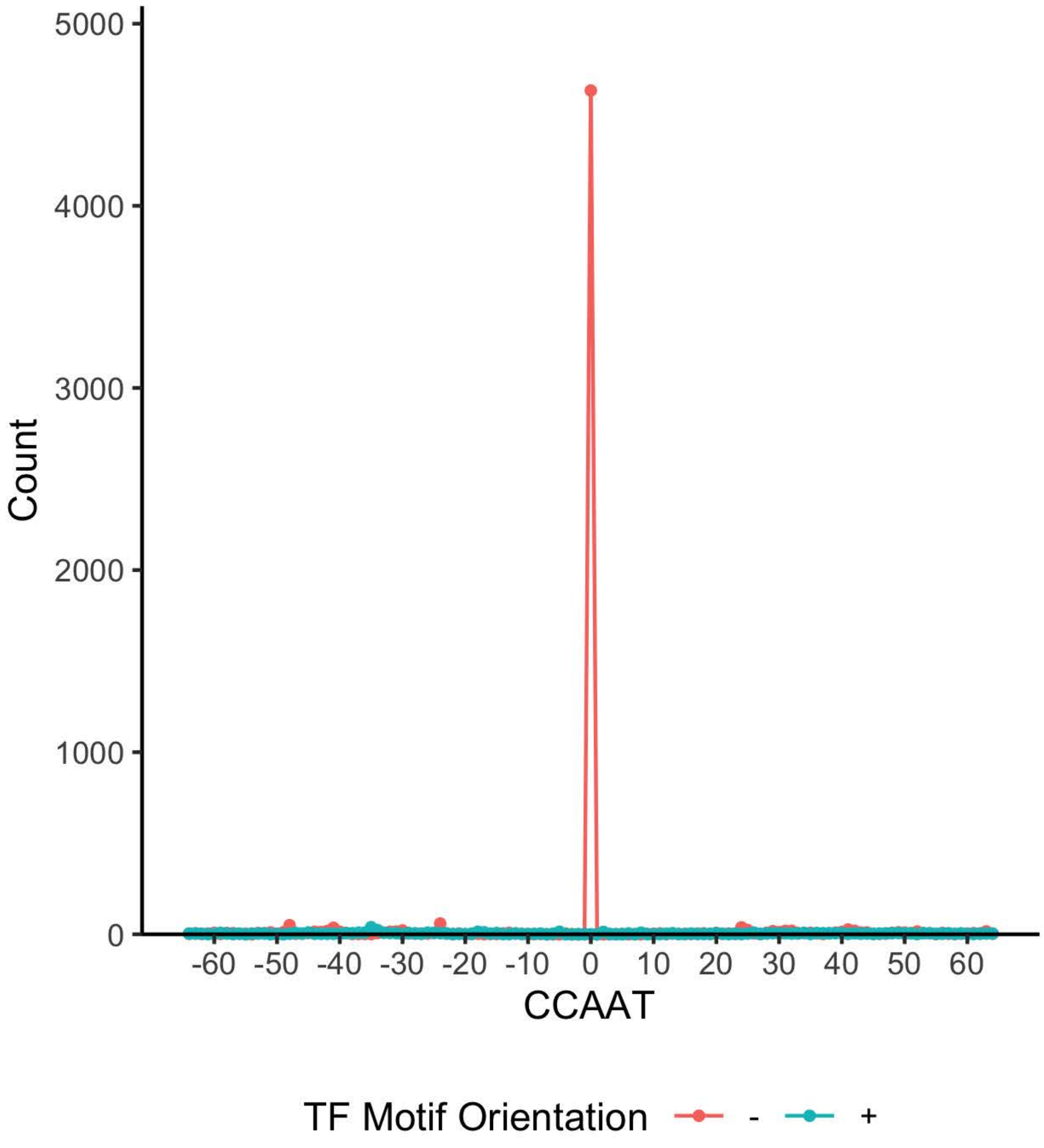

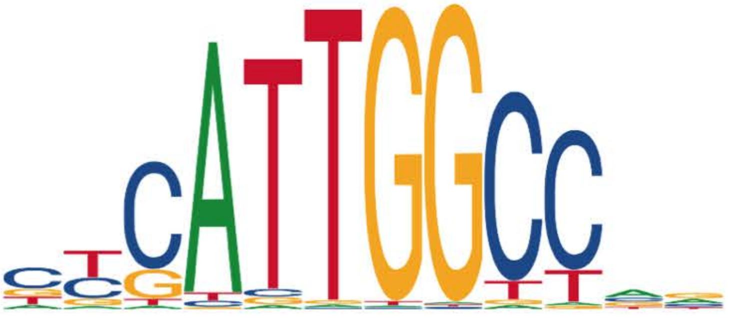

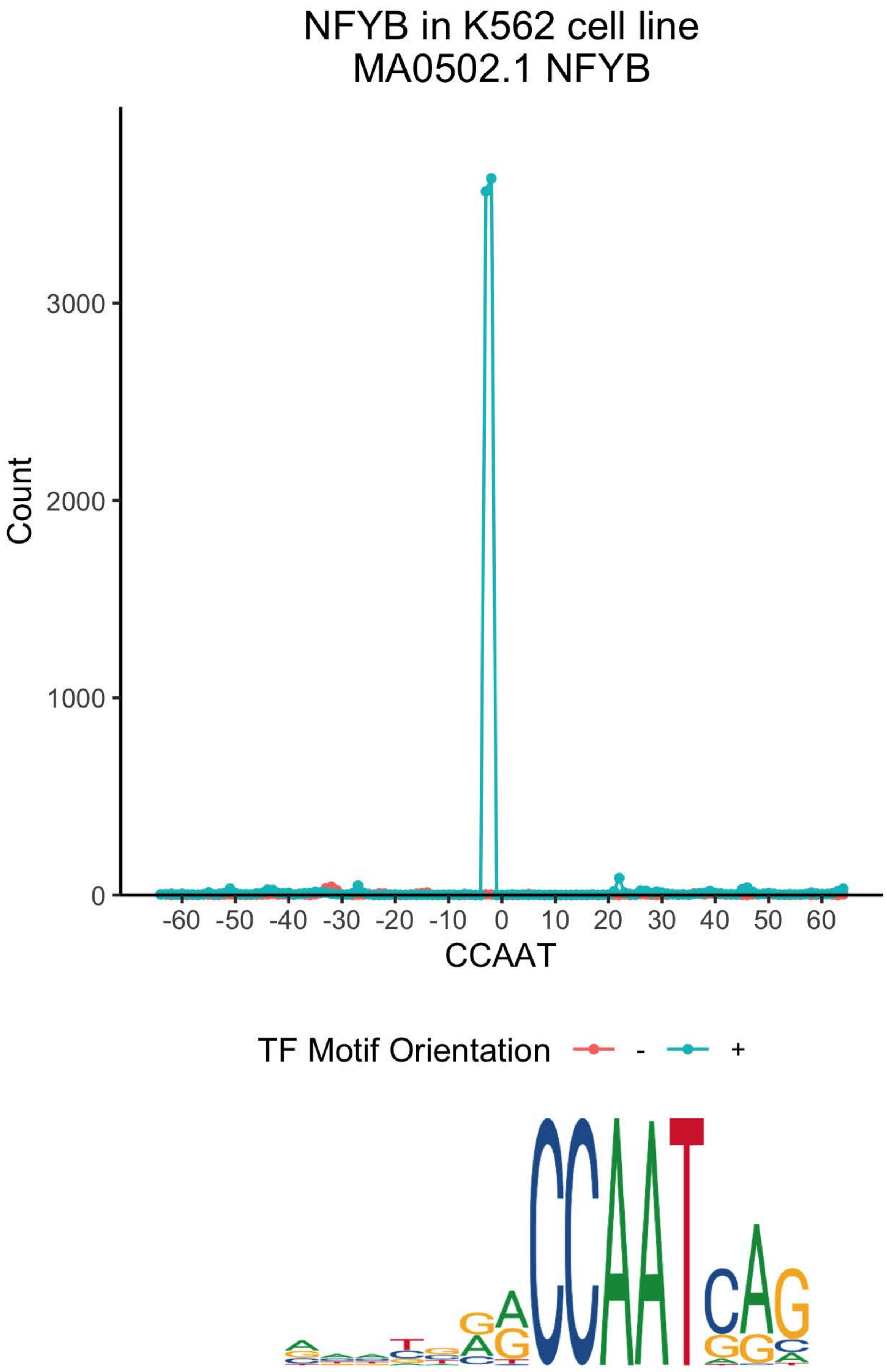
NFYB in K562 cell line
MA0502.1 NFYB
Count
3000
2000
1000
0
-60 -50 -40 -30 -20 -10 0 10 20 30 40 50 60
CCAAT
TF Motif Orientation
-
+

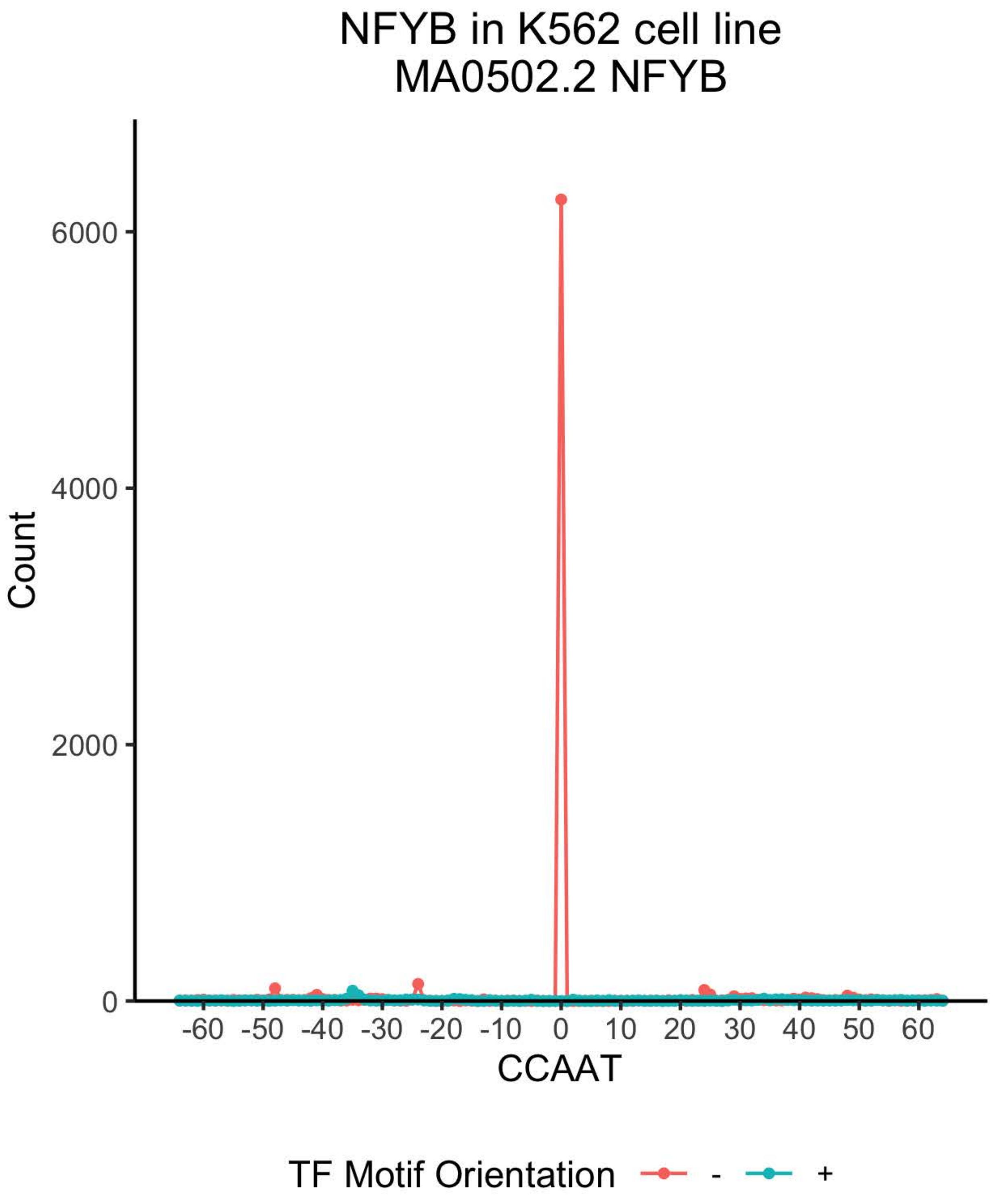
NFYB in K562 cell line
MA0502.2 NFYB
Count
6000
4000
2000
0
-60 -50 -40 -30 -20 -10 0 10 20 30 40 50 60
CCAAT
TF Motif Orientation
-
+

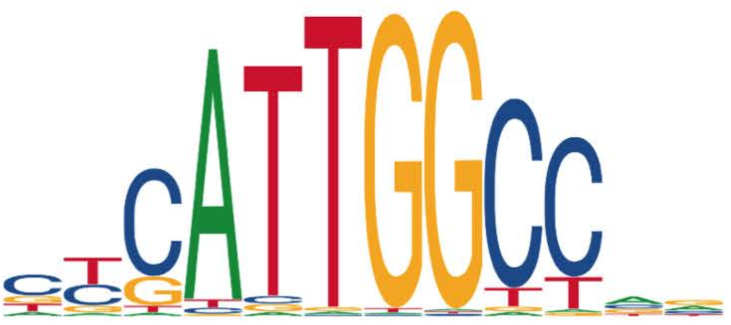

PBX2 in K562 cell line
MA1113.2 PBX2

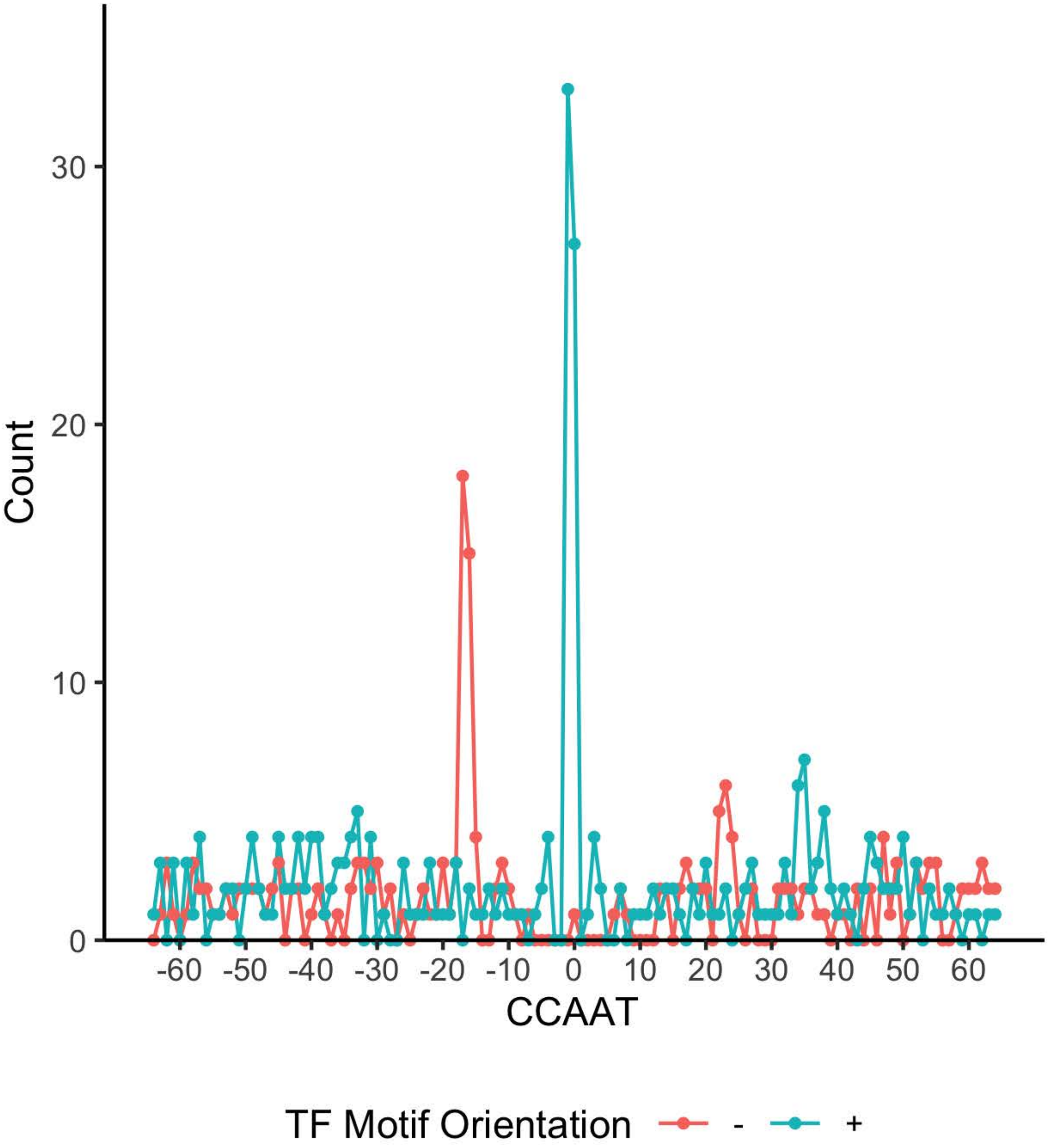

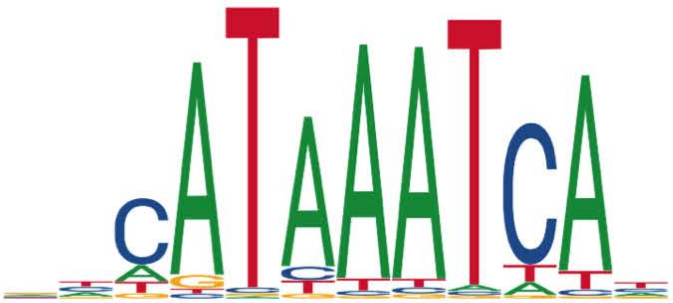

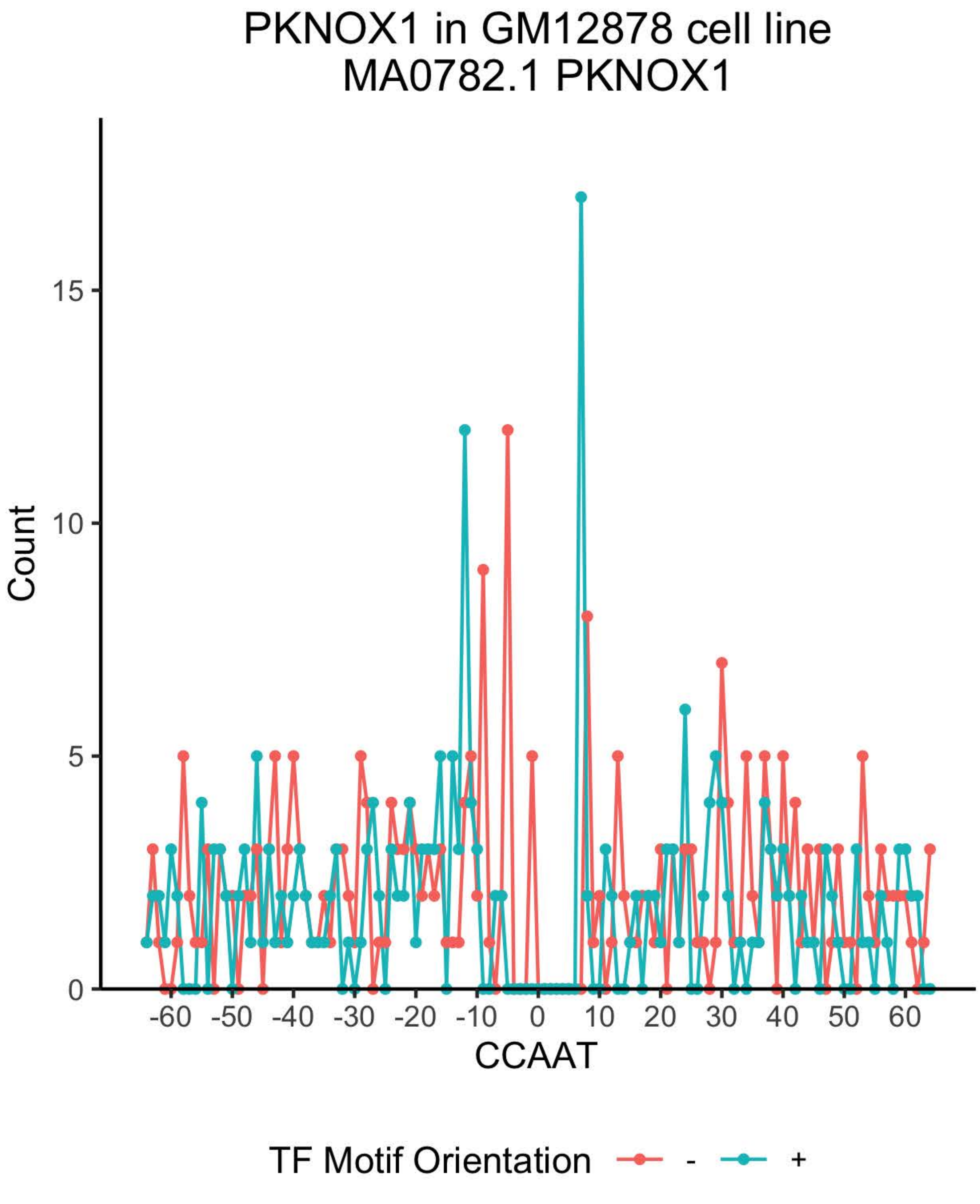

PKNOX1 in GM12878 cell line
MA0782.1 PKNOX1
Count
15
10
5
0
-60 -50 -40 -30 -20 -10 0 10 20 30 40 50 60
CCAAT
TF Motif Orientation
-
+

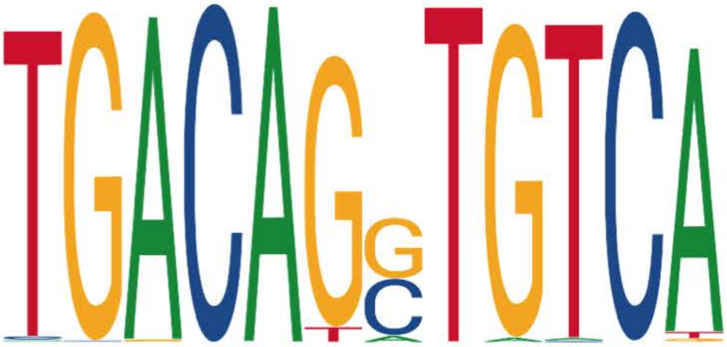

PKNOX1 in GM12878 cell line
MA0782.2 PKNOX1

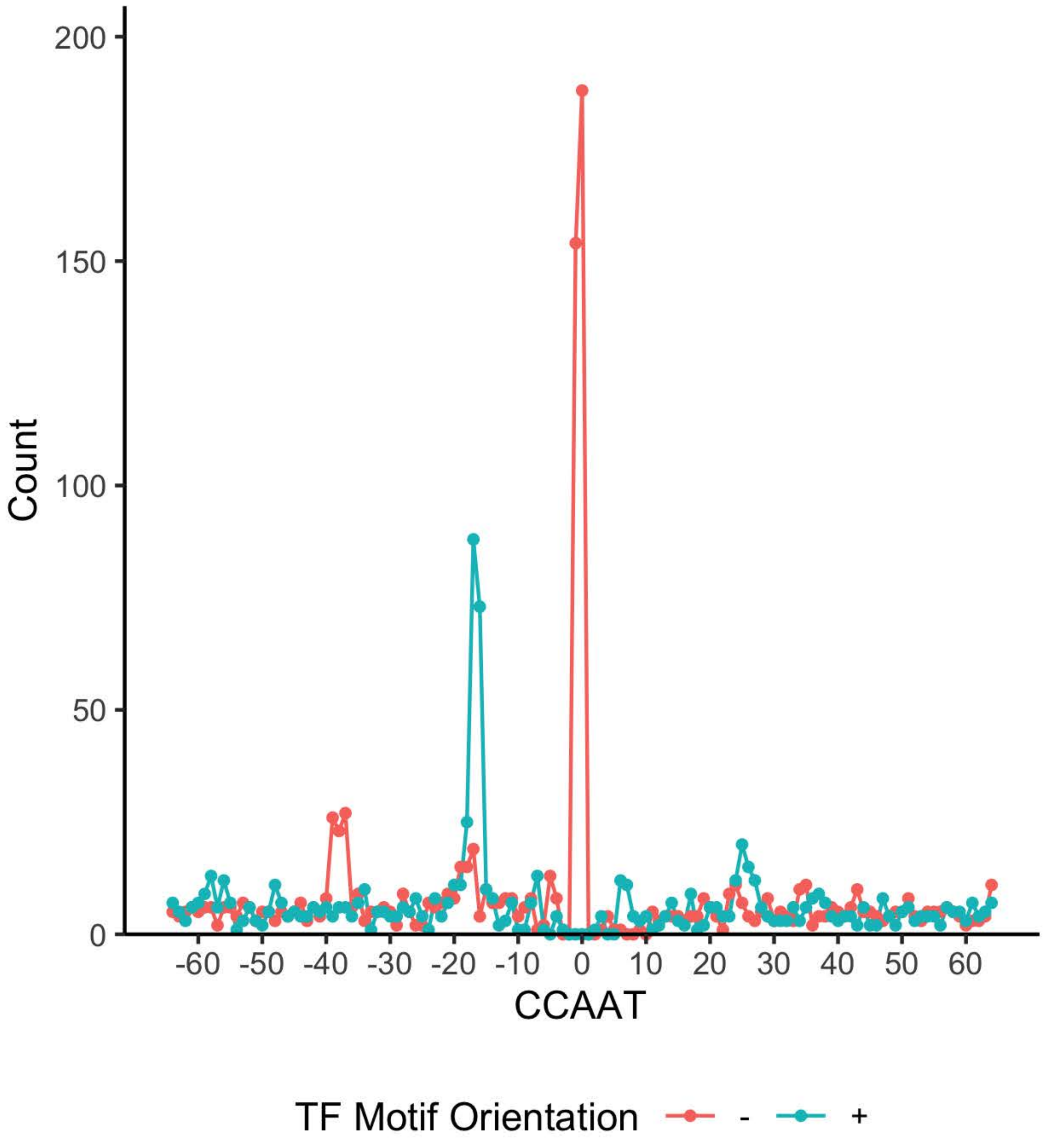

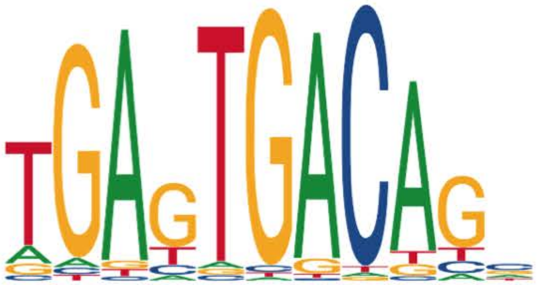

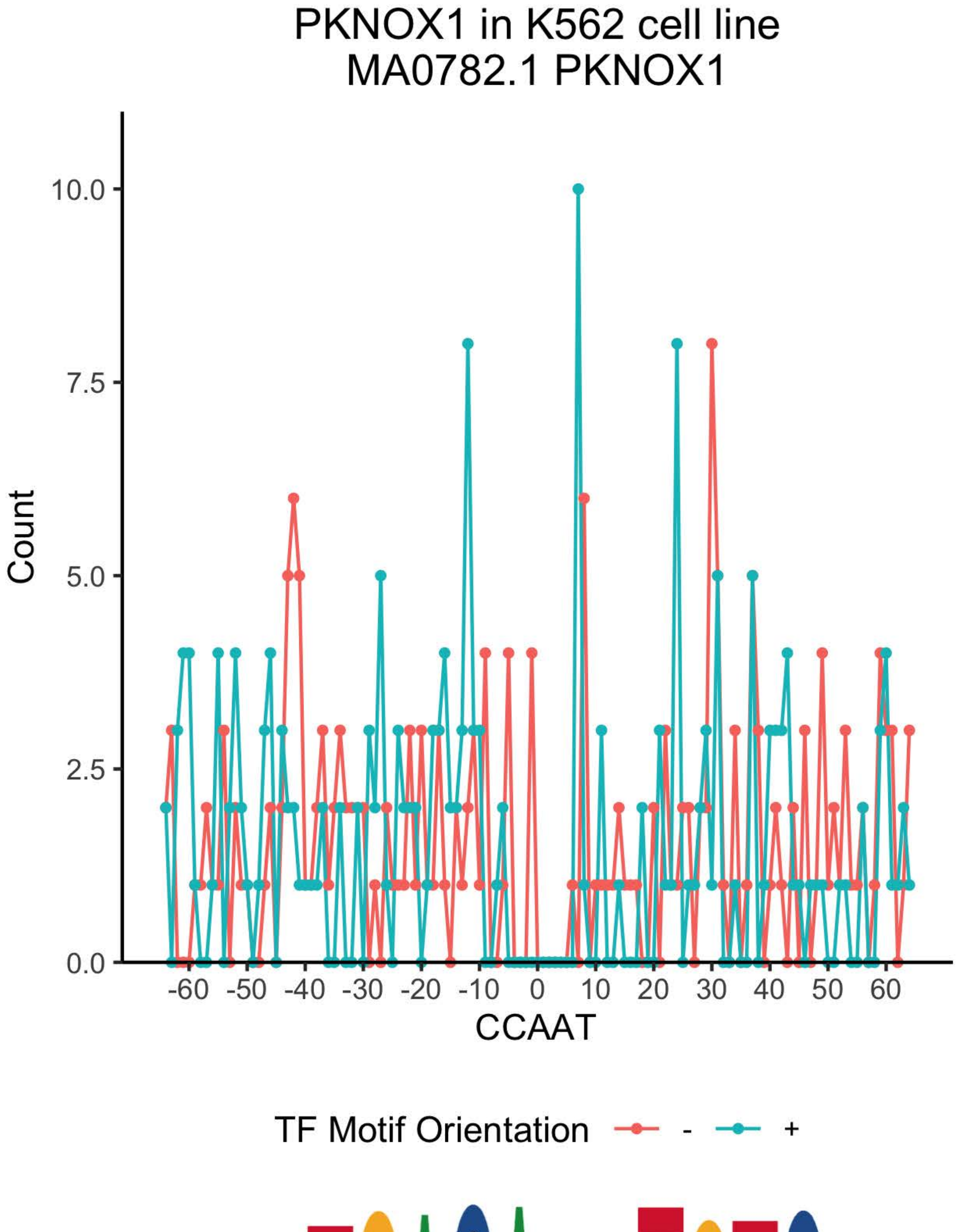

PKNOX1 in K562 cell line
MA0782.1 PKNOX1
Count
10.0
7.5
5.0
2.5
0.0
-60
-50
-40
-30
-20
-10
0
10
20
30
40
50
60
CCAAT
TF Motif Orientation
-
+

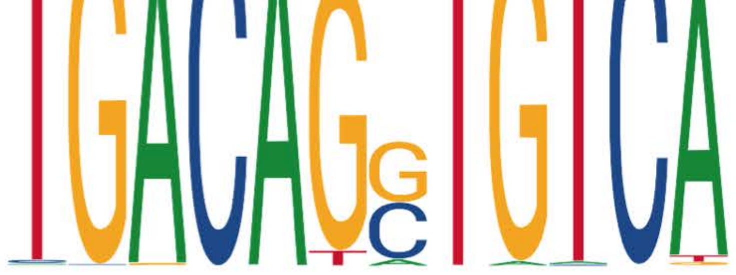

RFX5 in GM12878 cell line
MA0510.2 RFX5

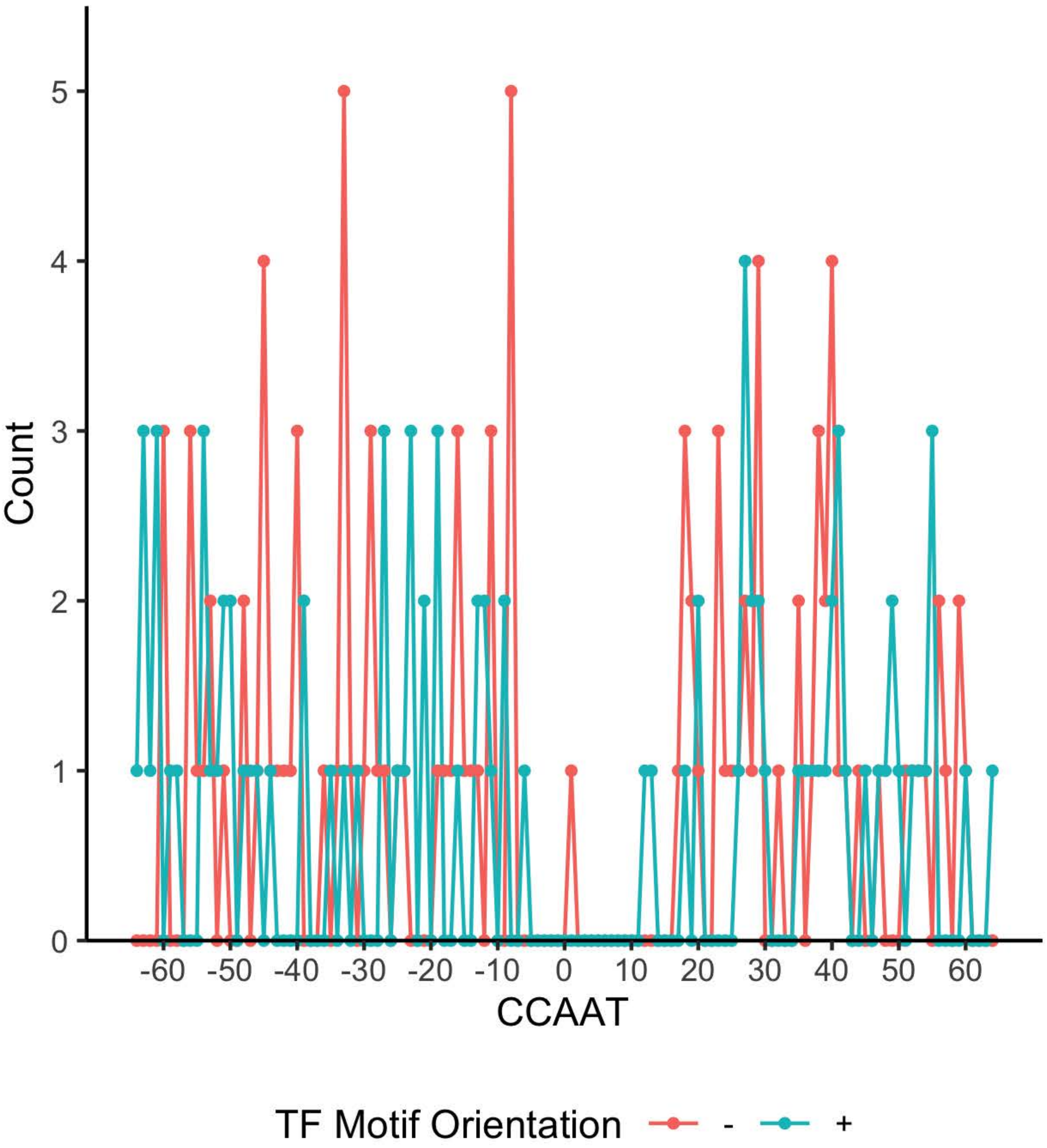

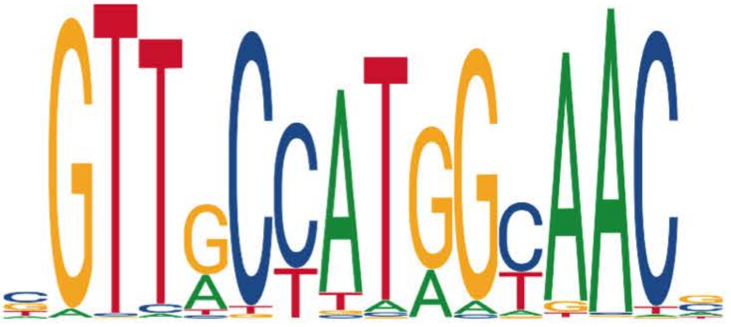

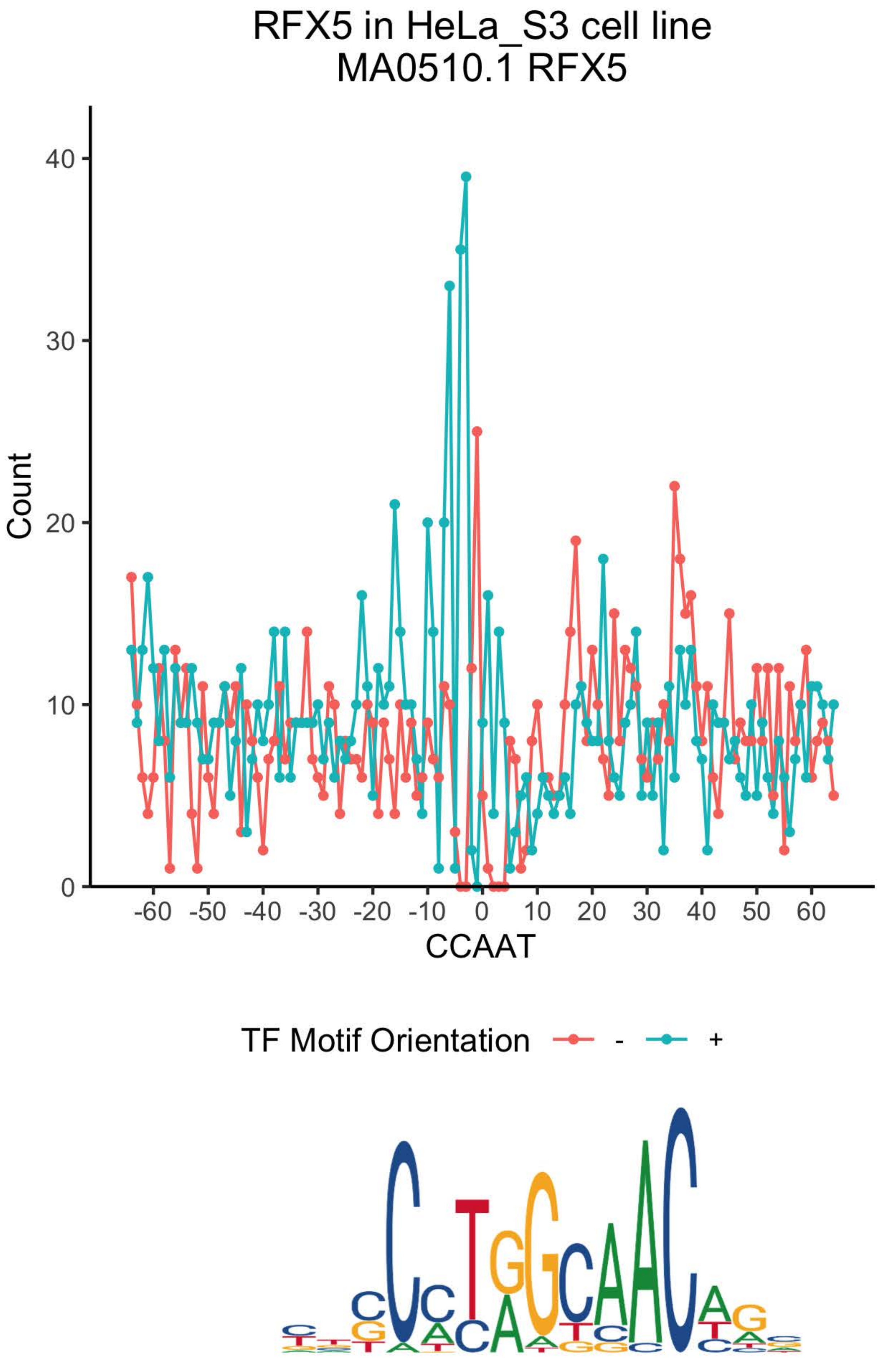
RFX5 in HeLa_S3 cell line
MA0510.1 RFX5
Count
40
30
20
10
0
-60 -50 -40 -30 -20 -10 0 10 20 30 40 50 60
CCAAT
TF Motif Orientation
-
+

RFX5 in HeLa_S3 cell line
MA0510.2 RFX5

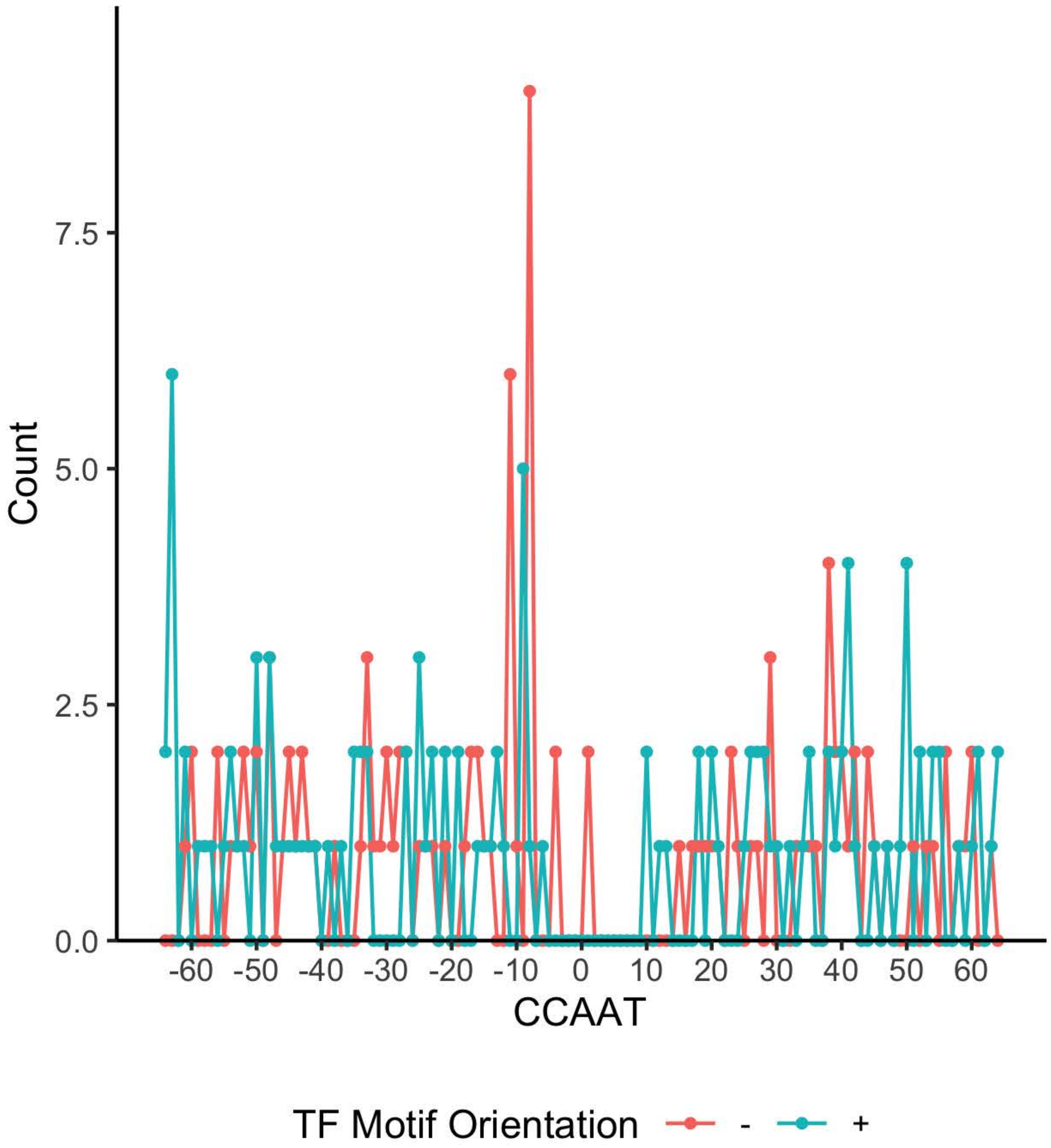

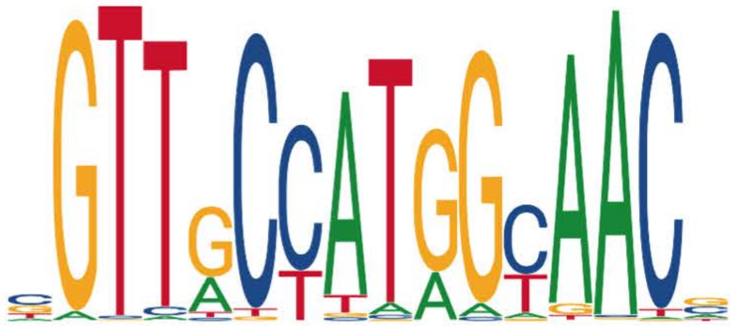

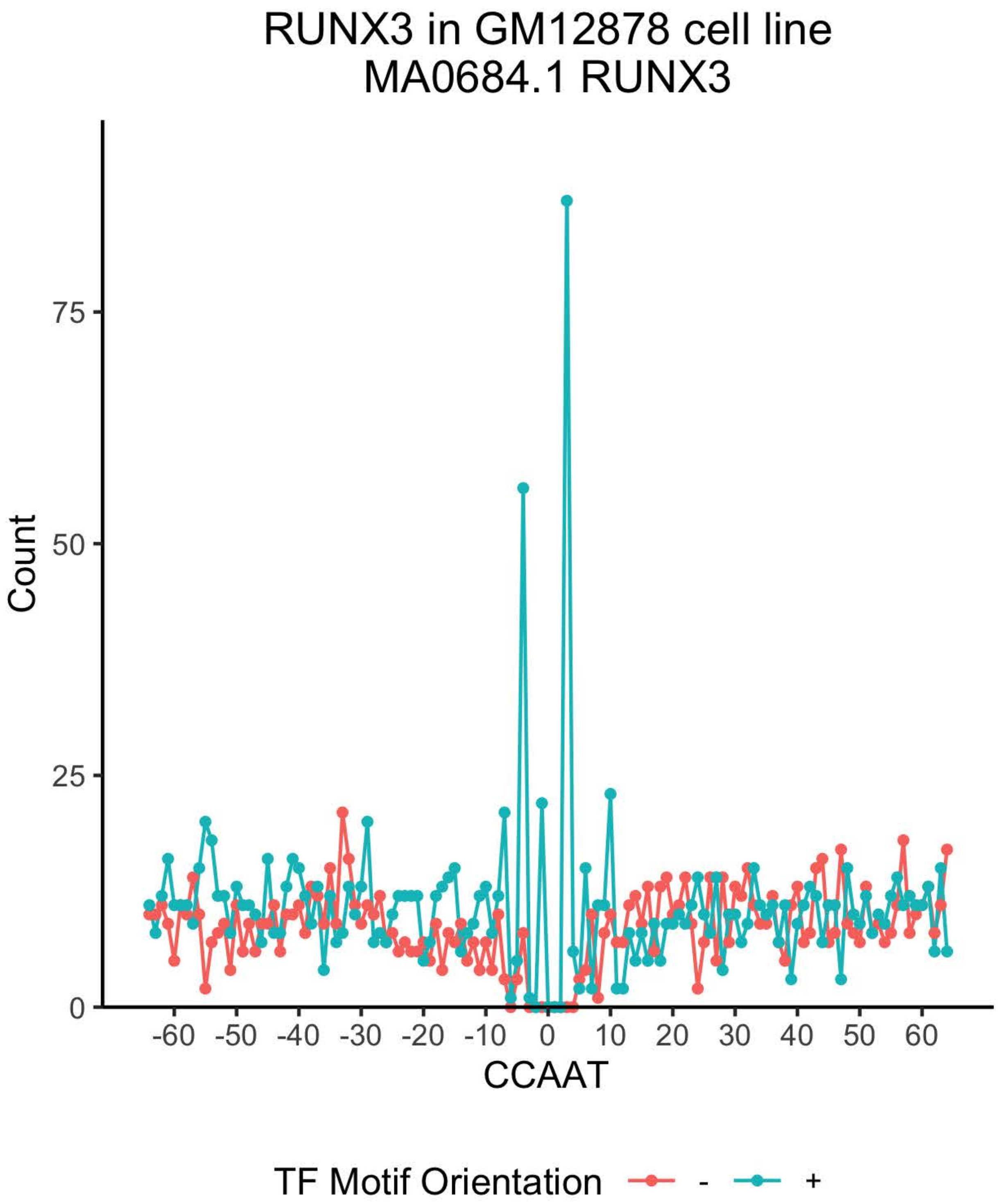

RUNX3 in GM12878 cell line
MA0684.1 RUNX3
Count
75
50
25
0
-60 -50 -40 -30 -20 -10 0 10 20 30 40 50 60
CCAAT
TF Motif Orientation
-
+

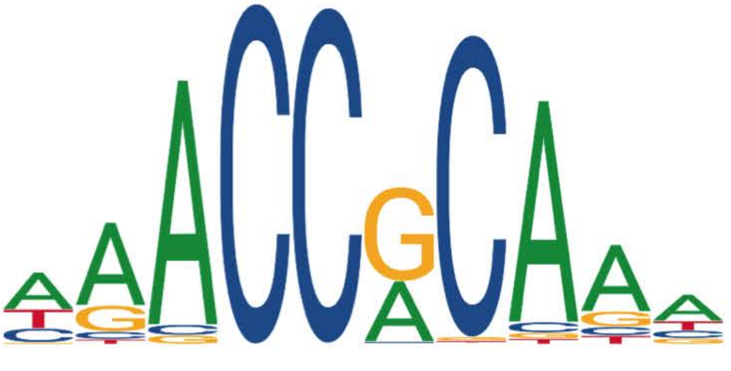

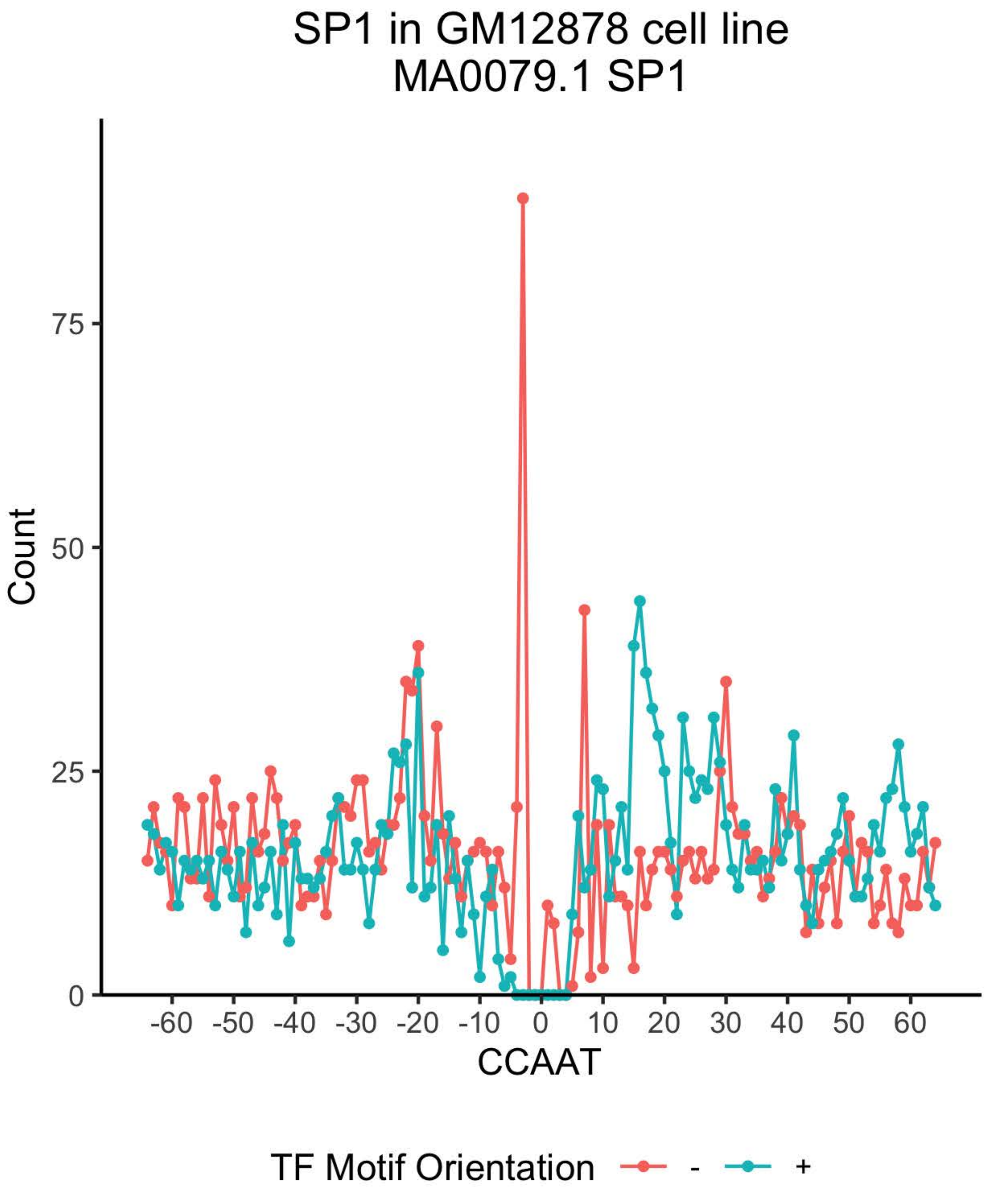

SP1 in GM12878 cell line
MA0079.1 SP1
Count
75
50
25
0
-60 -50 -40 -30 -20 -10 0 10 20 30 40 50 60
CCAAT
TF Motif Orientation
-
+

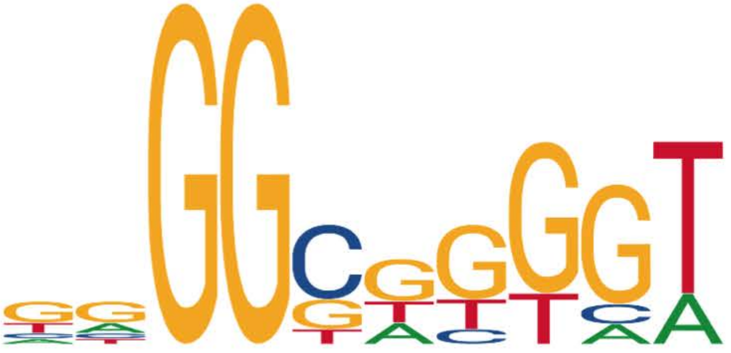

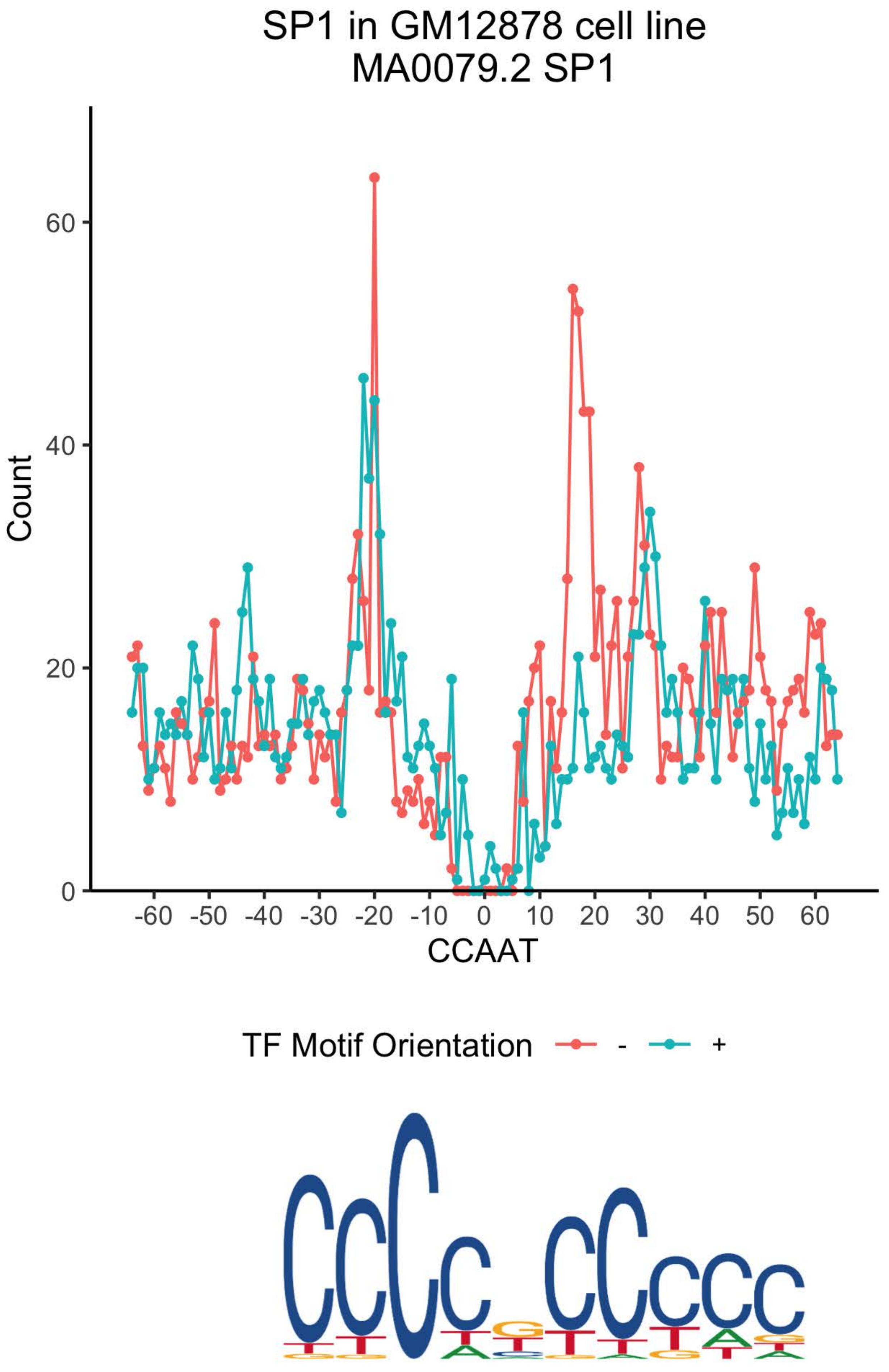
SP1 in GM12878 cell line
MA0079.2 SP1
Count
0
20
40
60
-60 -50 -40 -30 -20 -10 0 10 20 30 40 50 60
CCAAT
TF Motif Orientation
-
+

SP1 in GM12878 cell line
MA0079.3 SP1

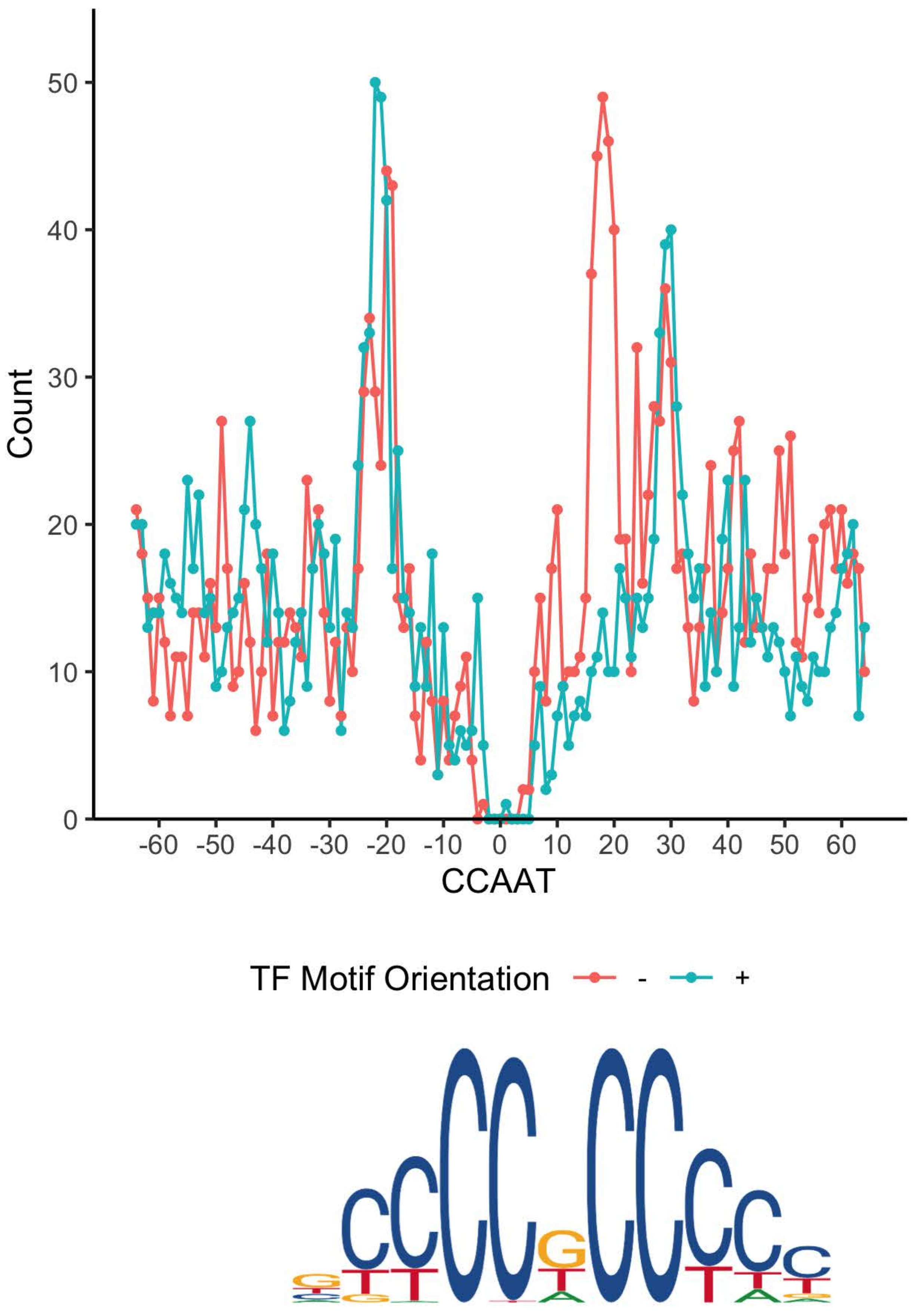

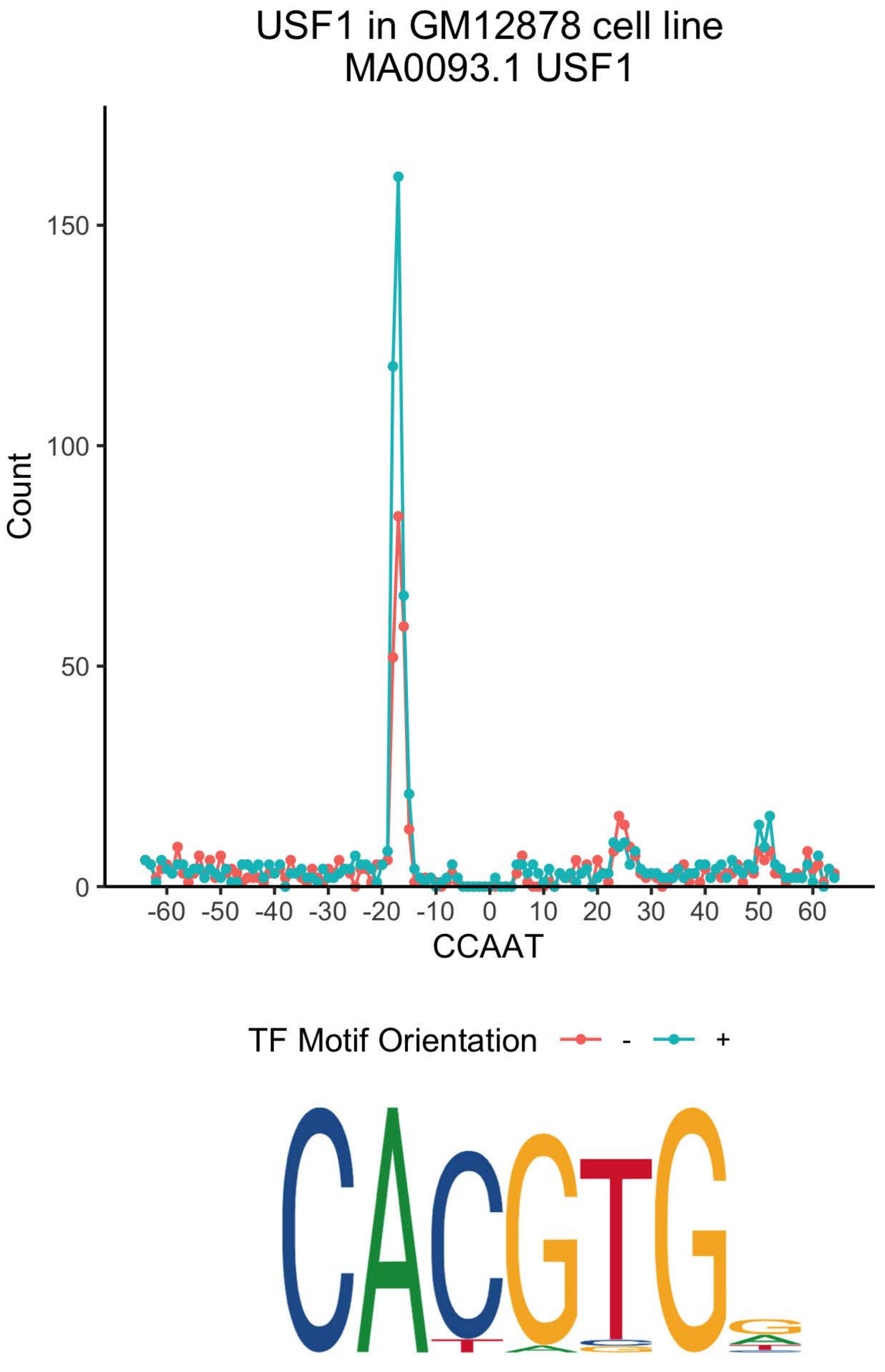
USF1 in GM12878 cell line
MA0093.1 USF1
Count
150
100
50
0
-60 -50 -40 -30 -20 -10 0 10 20 30 40 50 60
CCAAT
TF Motif Orientation
-
+
CACGTG

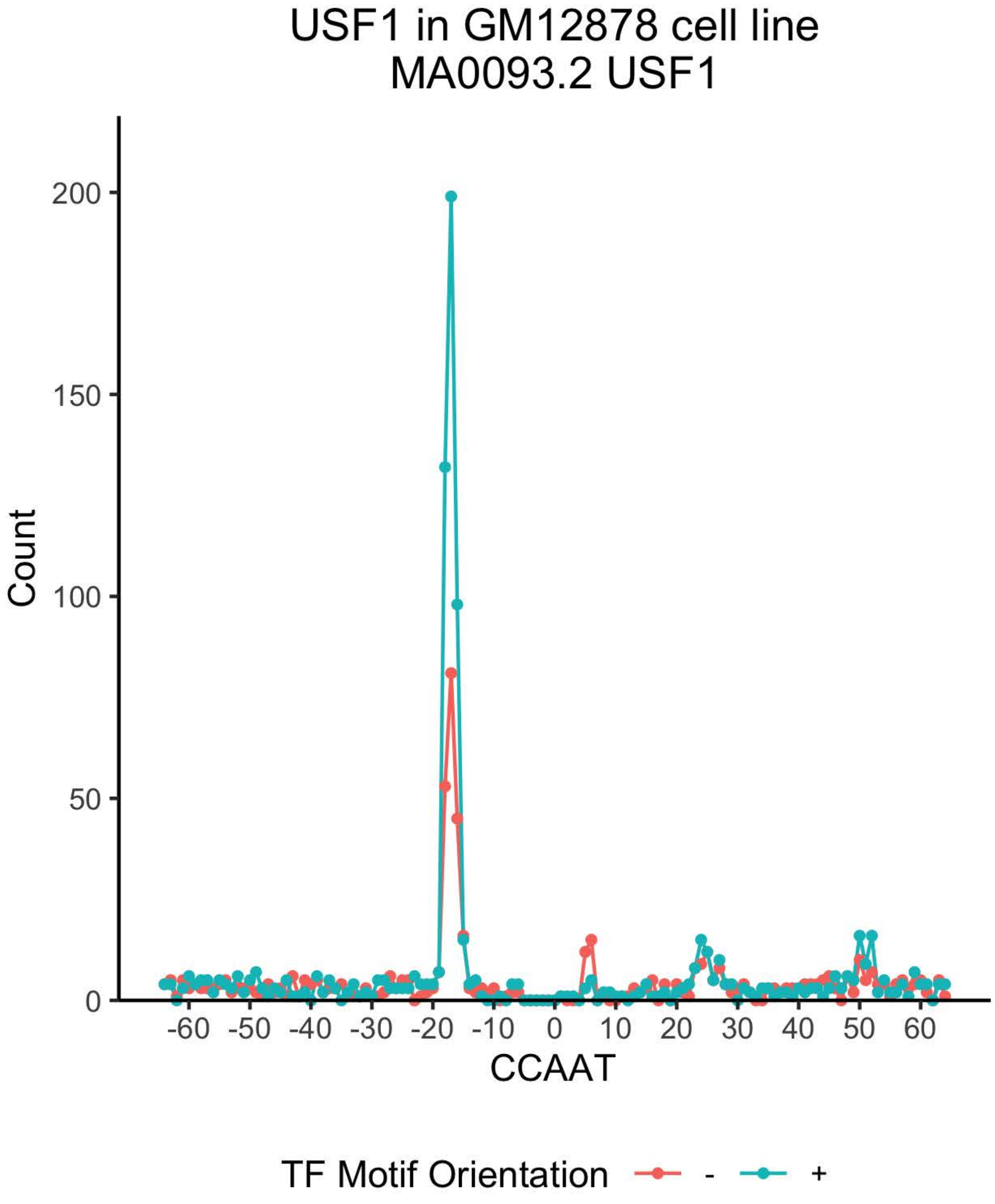
USF1 in GM12878 cell line
MA0093.2 USF1
Count
200
150
100
50
0
-60 -50 -40 -30 -20 -10 0 10 20 30 40 50 60
CCAAT
TF Motif Orientation
-
+

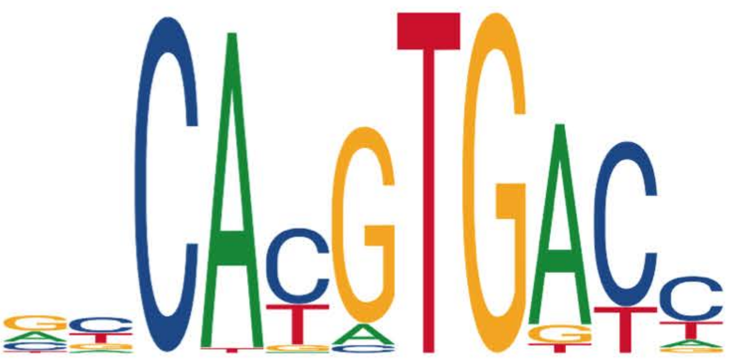

USF1 in GM12878 cell line
MA0093.3 USF1

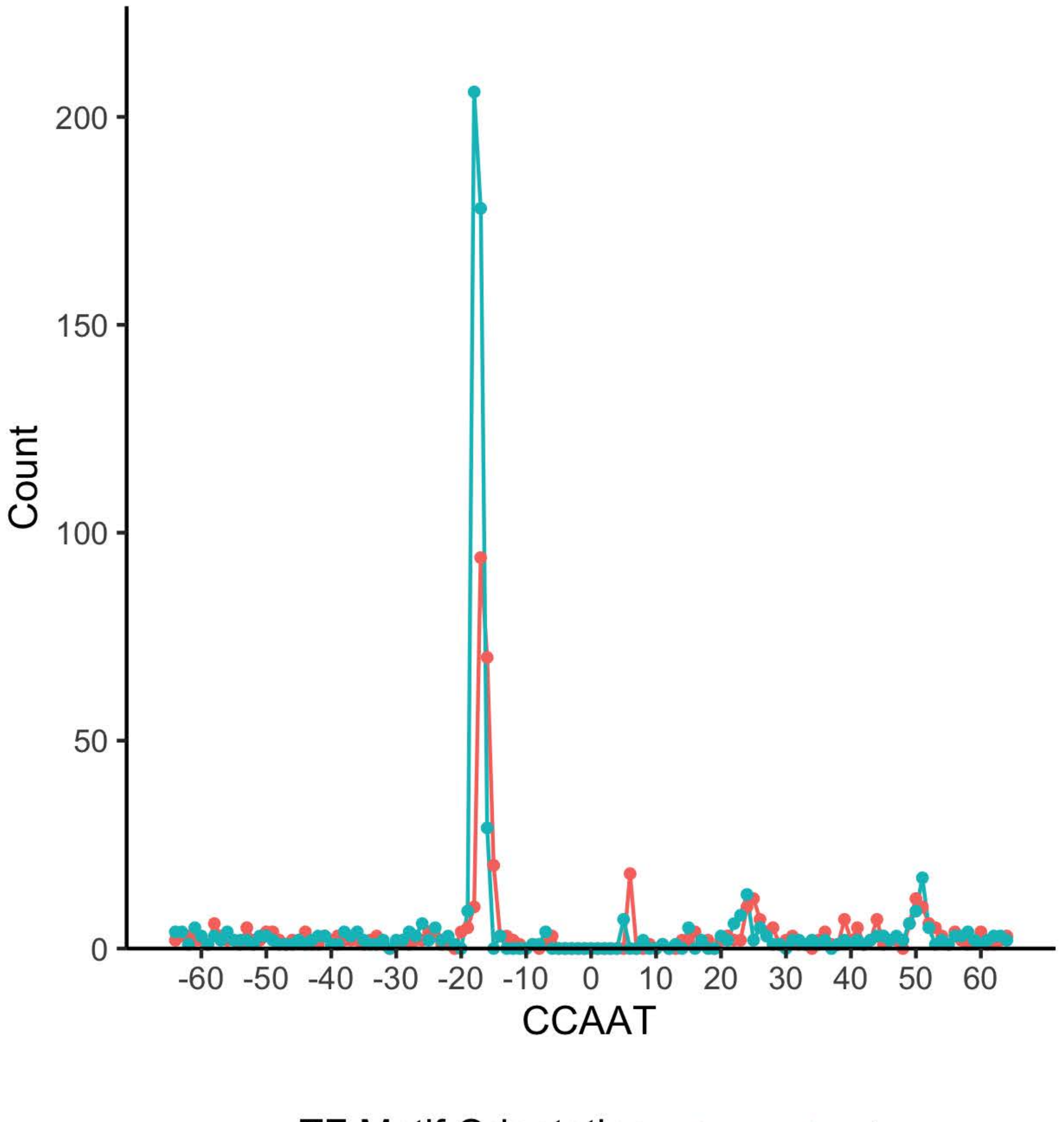

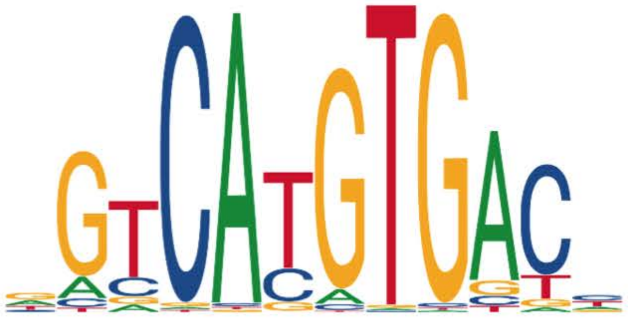

USF1 in K562 cell line
MA0093.1 USF1

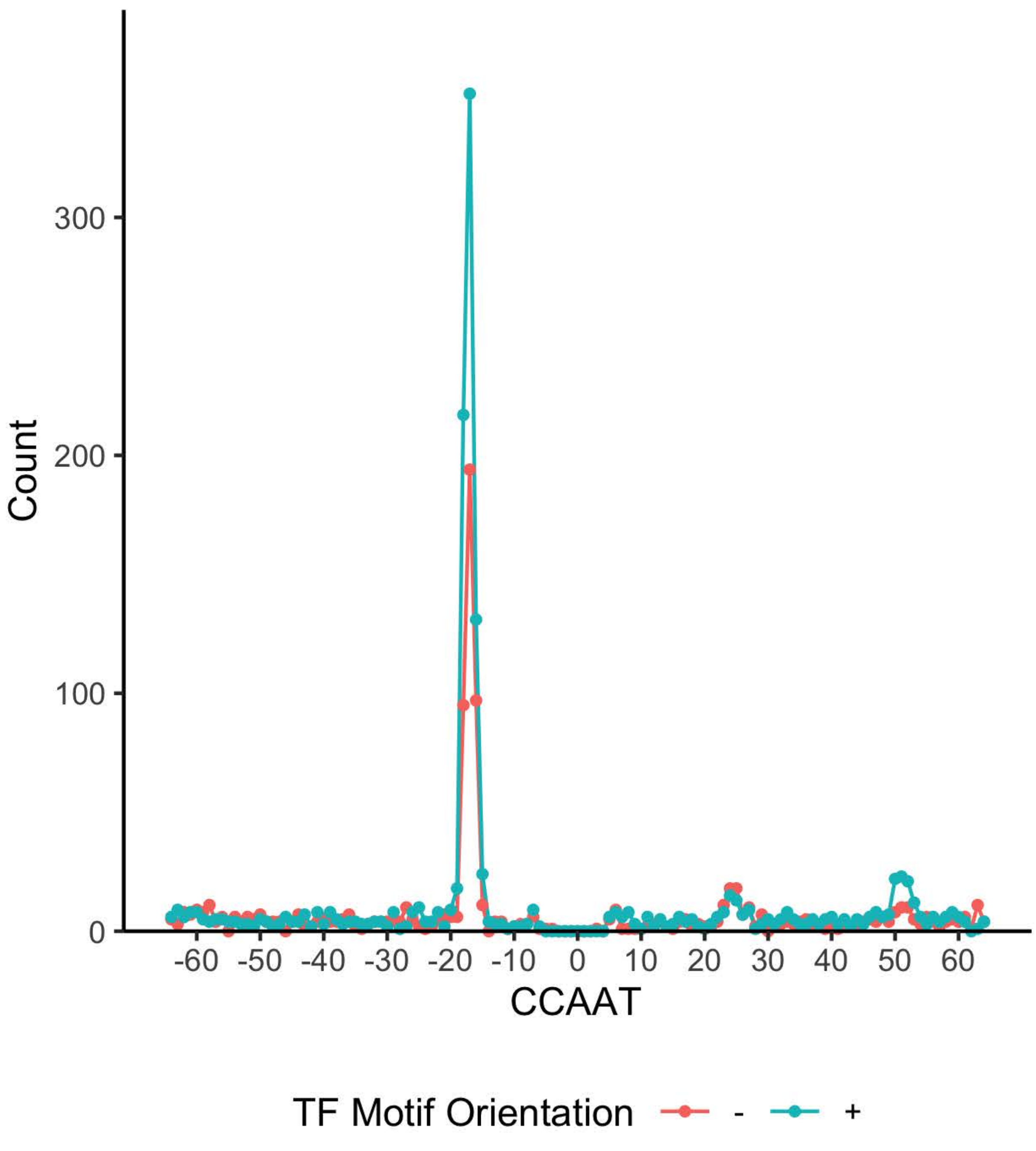

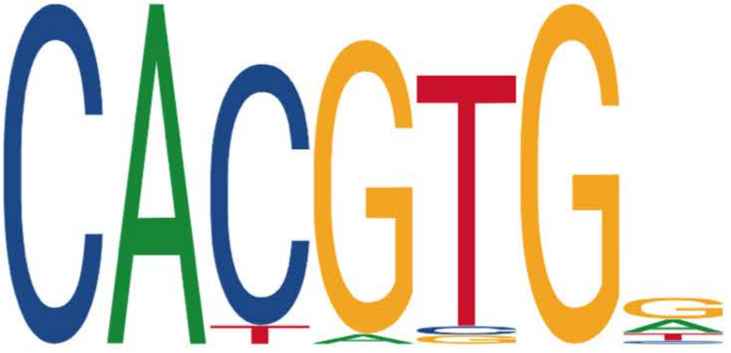

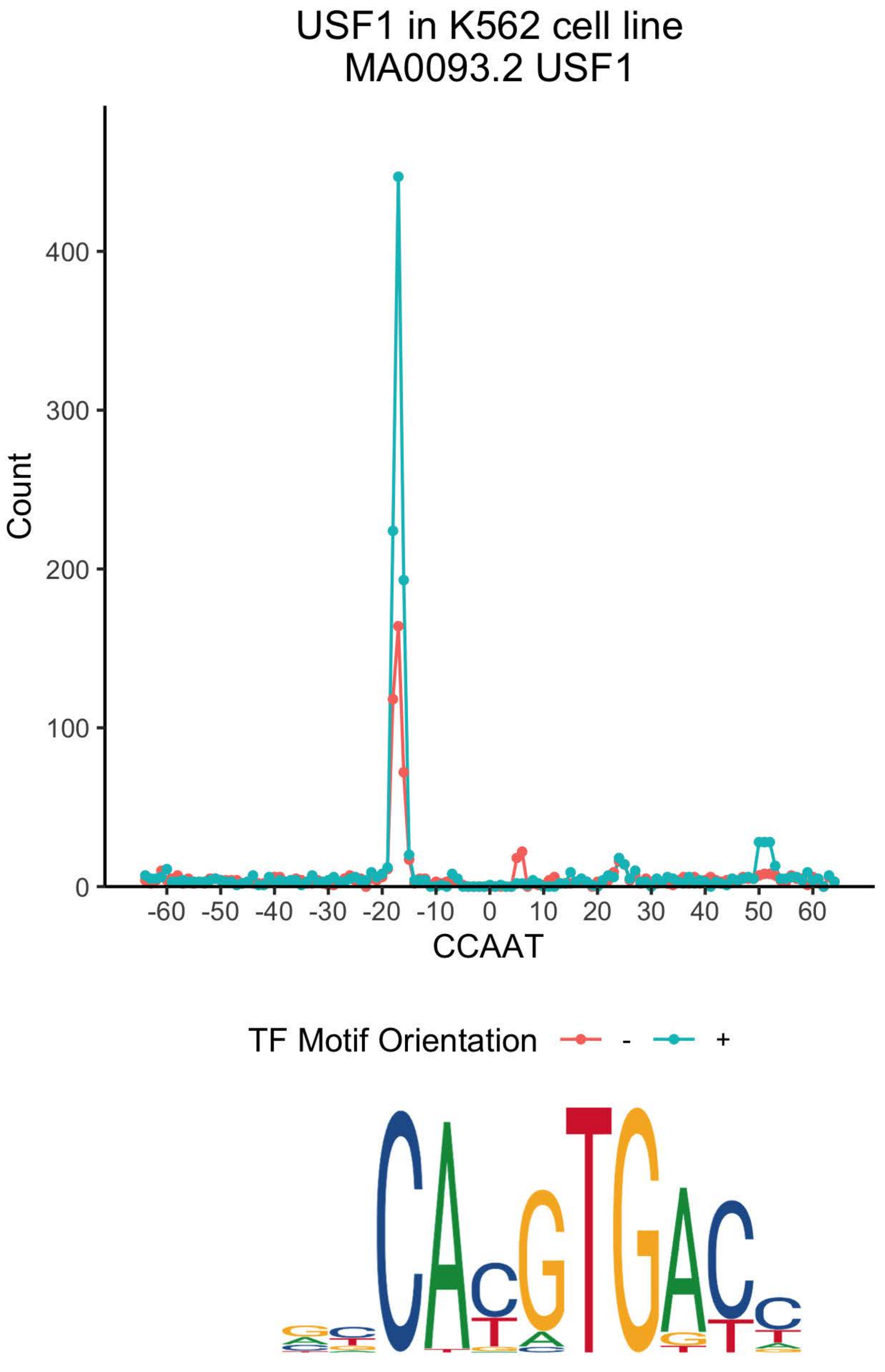

USF1 in K562 cell line
MA0093.2 USF1
Count
400
300
200
100
0
-60 -50 -40 -30 -20 -10 0 10 20 30 40 50 60
CCAAT
TF Motif Orientation
-
+

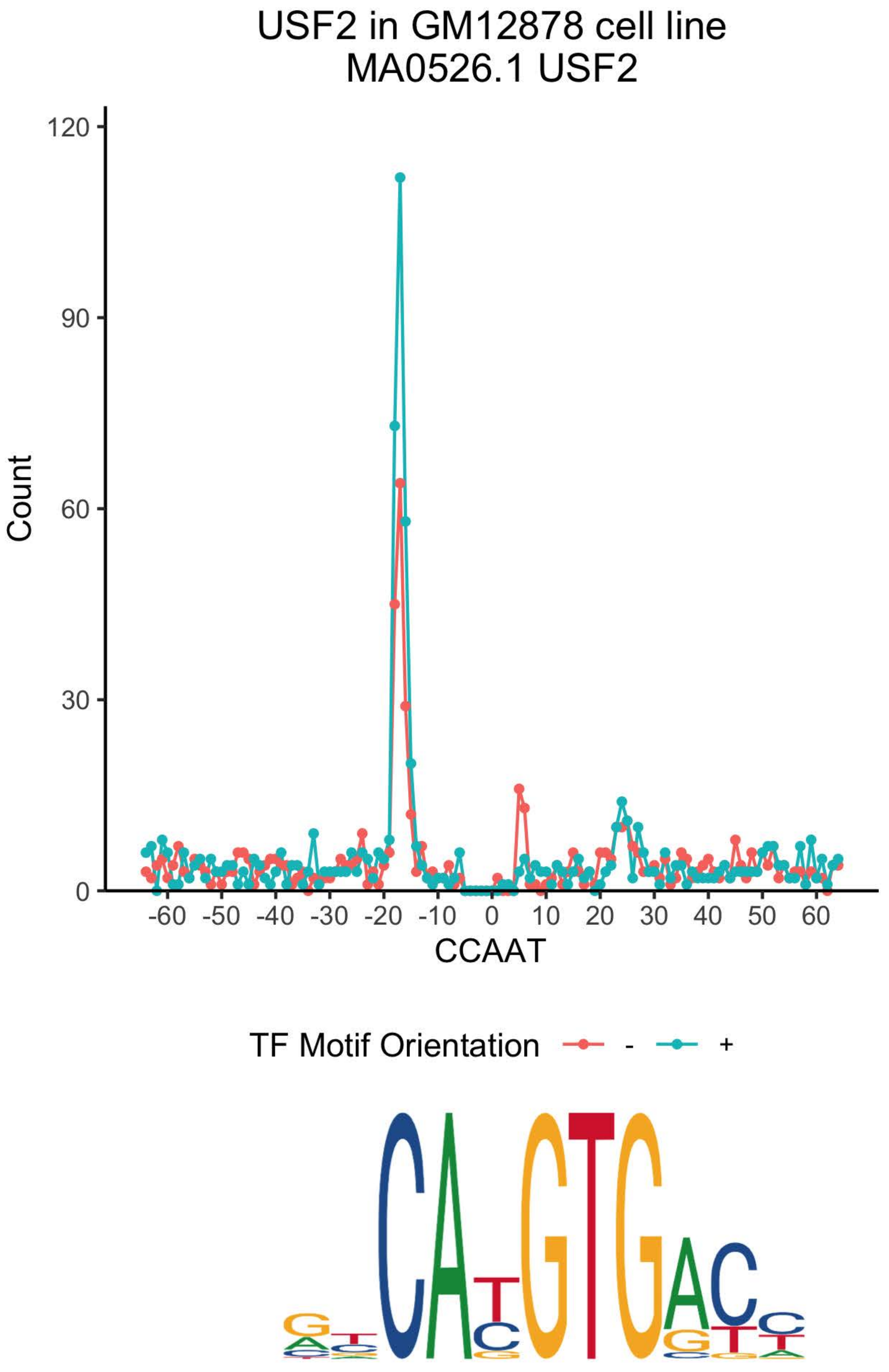

USF2 in GM12878 cell line
MA0526.1 USF2
Count
120
90
60
30
0
-60 -50 -40 -30 -20 -10 0 10 20 30 40 50 60
CCAAT
TF Motif Orientation - +

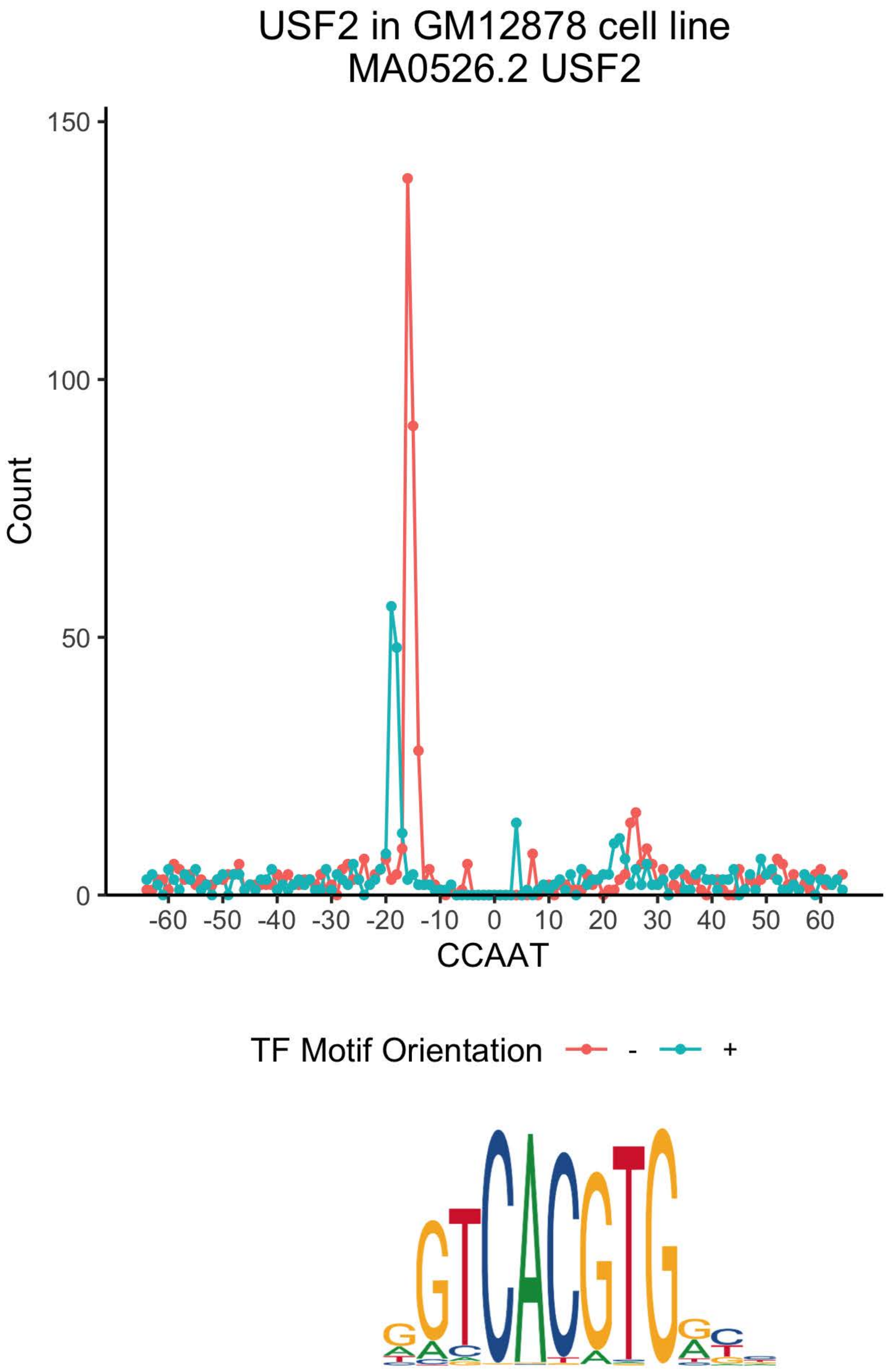
USF2 in GM12878 cell line
MA0526.2 USF2
Count
150
100
50
0
-60 -50 -40 -30 -20 -10 0 10 20 30 40 50 60
CCAAT
TF Motif Orientation
-
+

USF2 in GM12878 cell line
MA0526.3 USF2

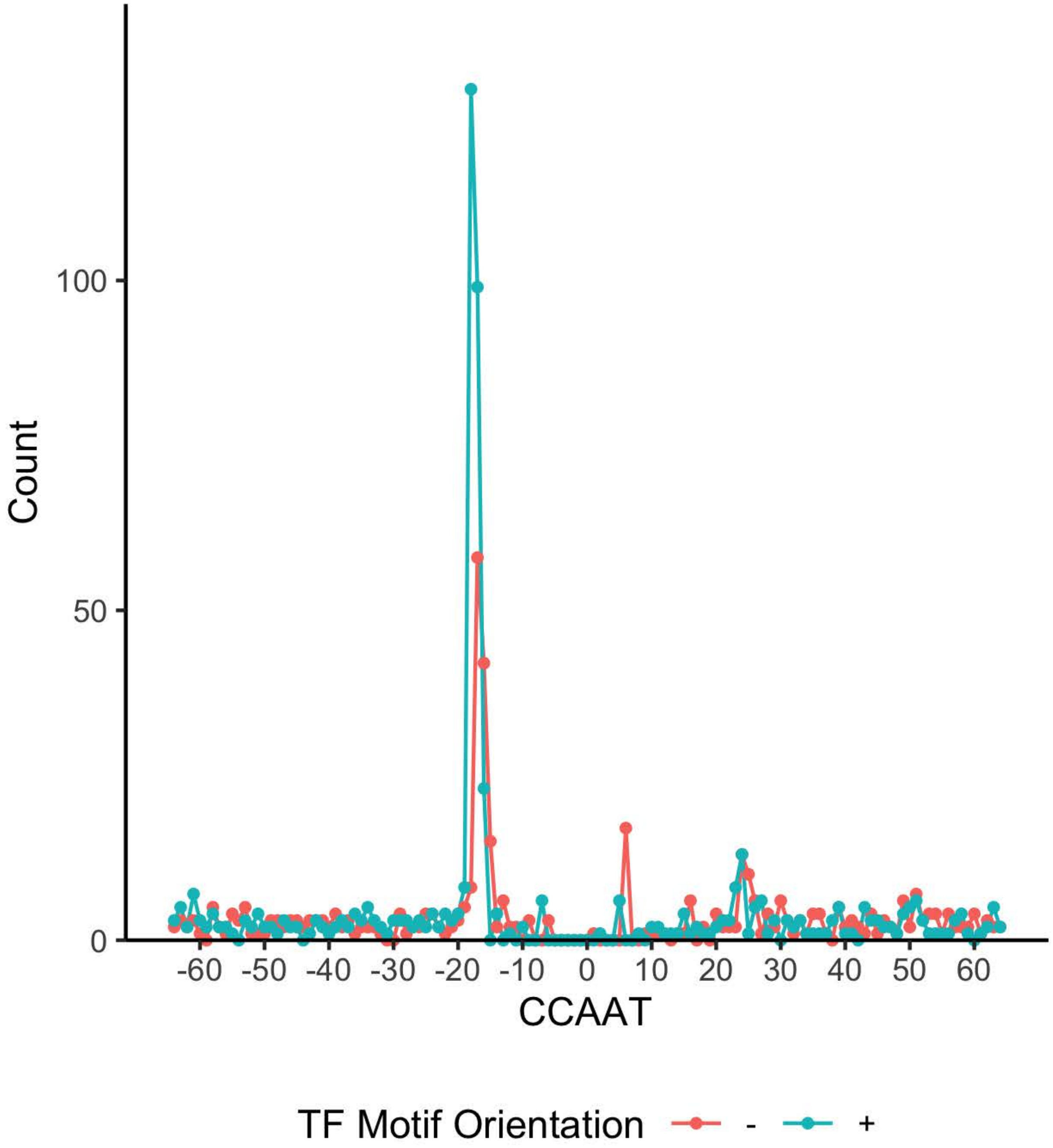

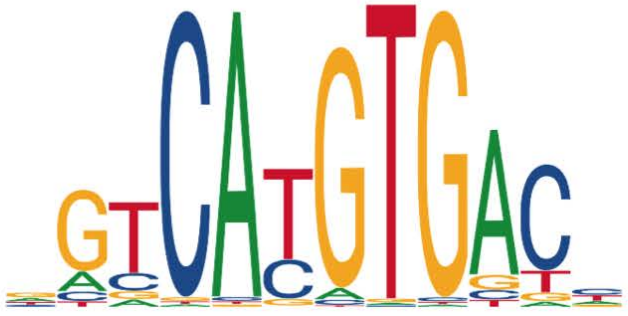

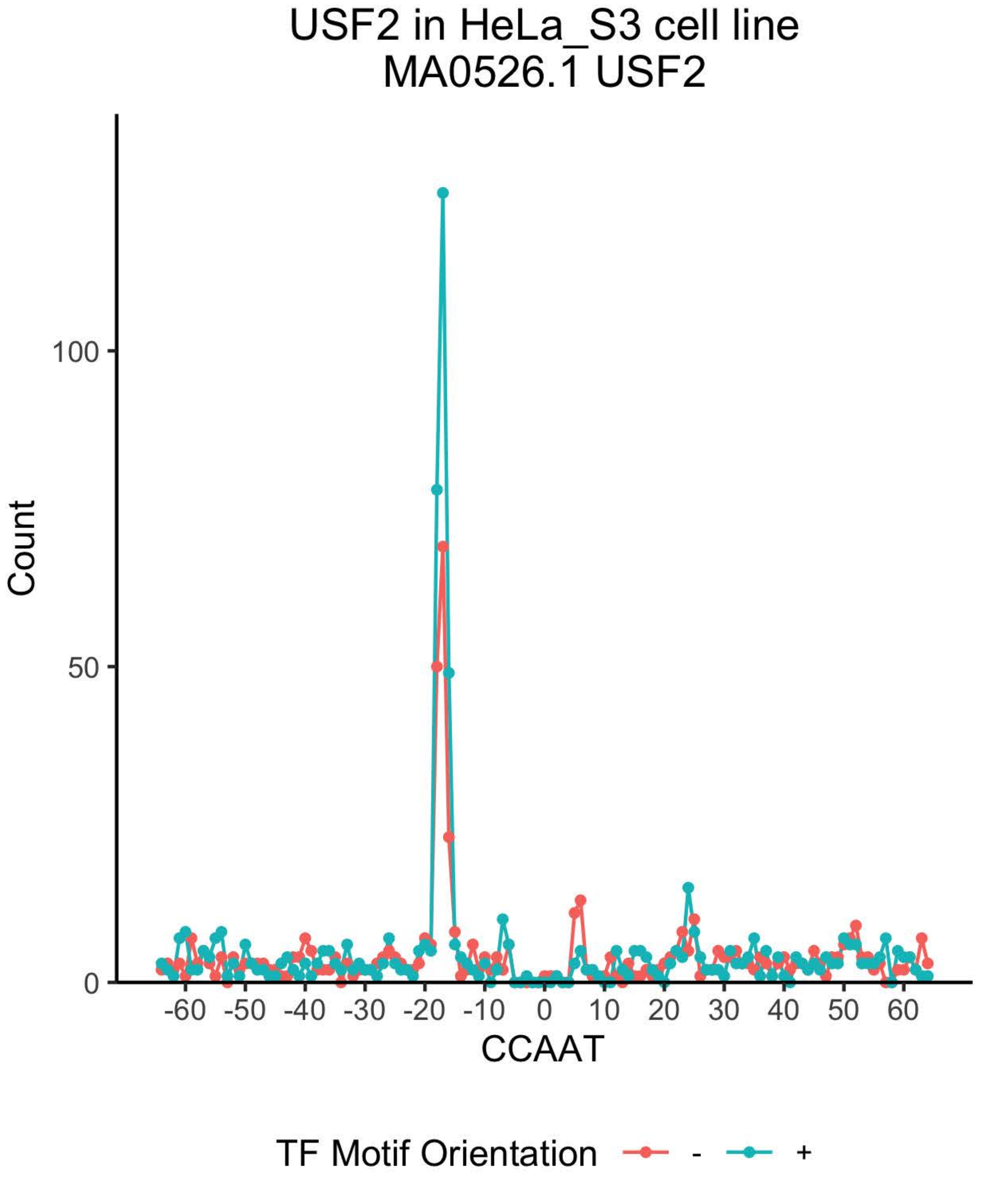

USF2 in HeLa_S3 cell line
MA0526.1 USF2
Count
100
50
0
-60
-50
-40
-30
-20
-10
0
10
20
30
40
50
60
CCAAT
TF Motif Orientation
-
+

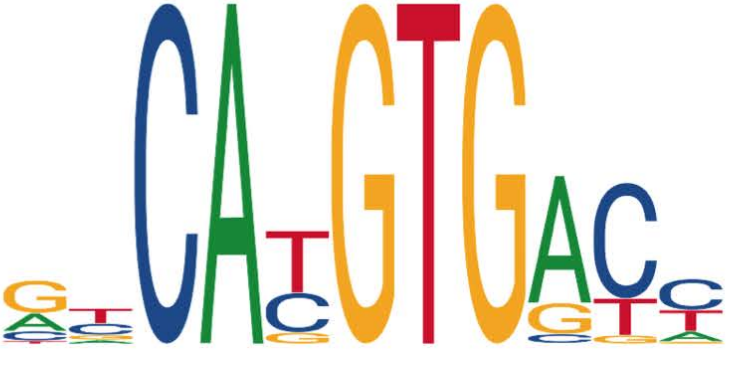

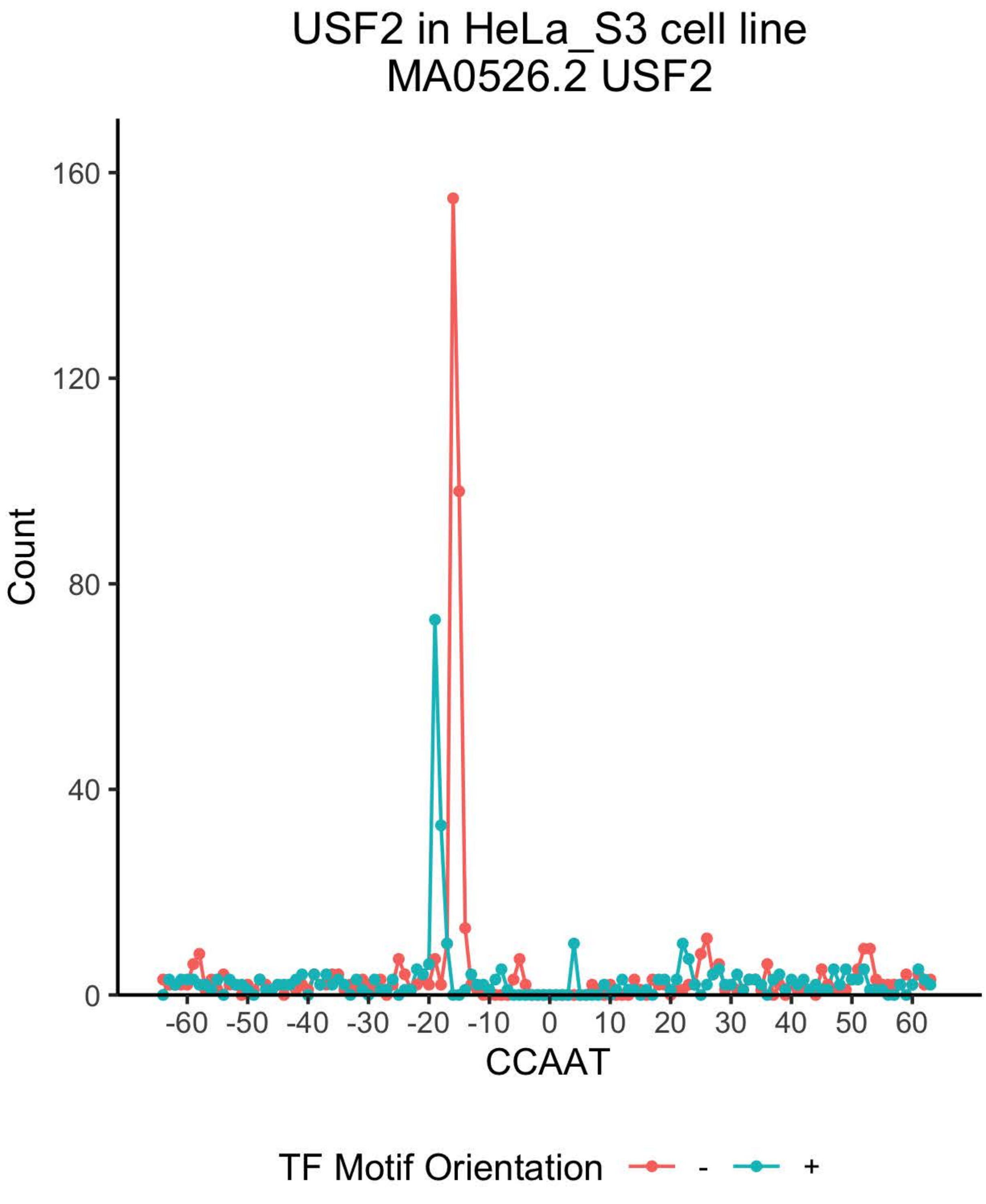
USF2 in HeLa_S3 cell line
MA0526.2 USF2
Count
160
120
80
40
0
-60 -50 -40 -30 -20 -10 0 10 20 30 40 50 60
CCAAT
TF Motif Orientation
-
+

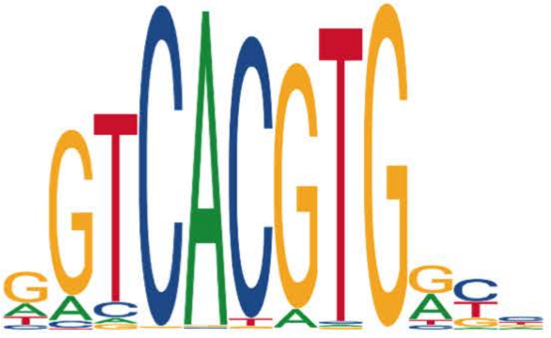

USF2 in HeLa_S3 cell line
MA0526.3 USF2

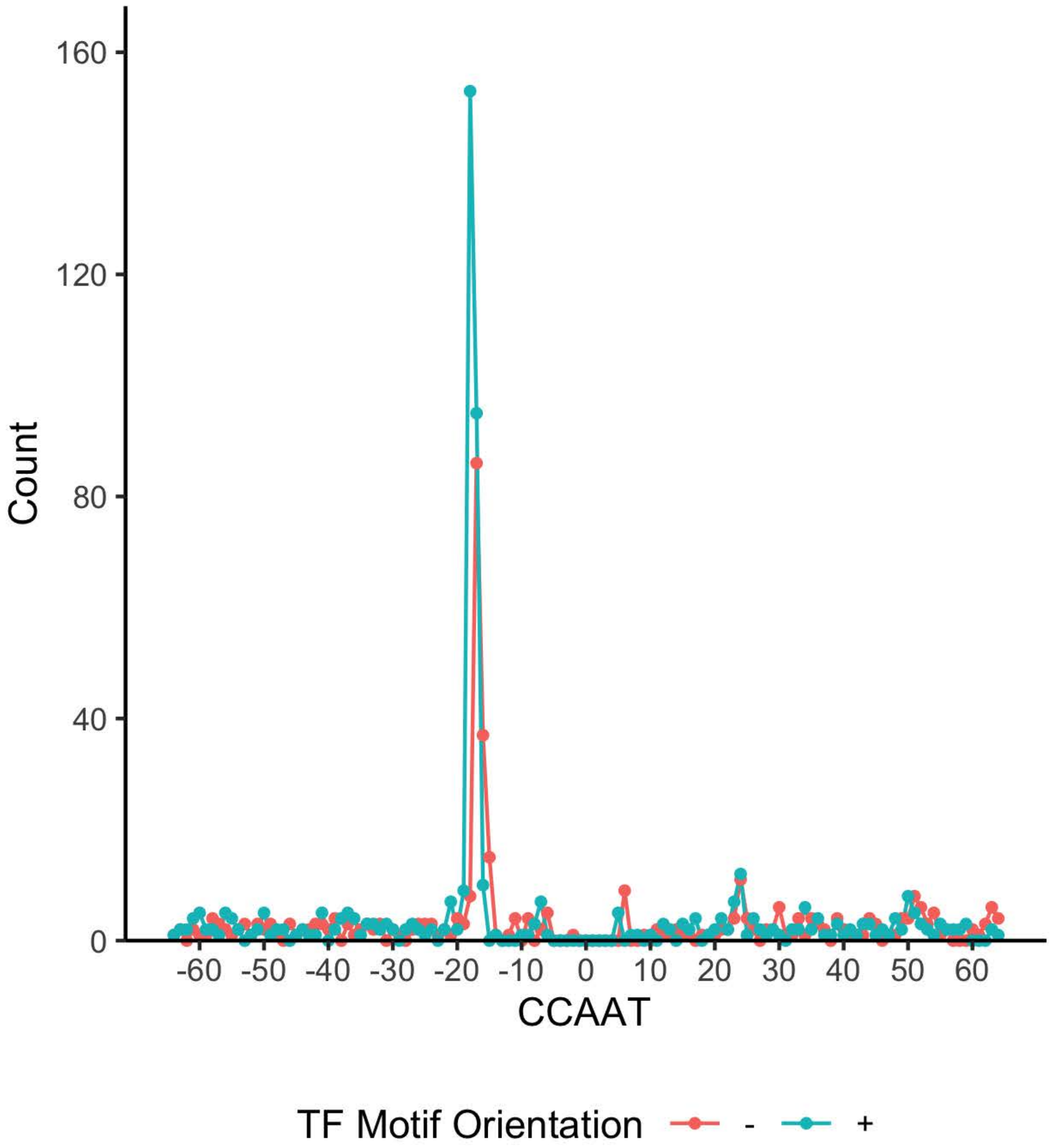

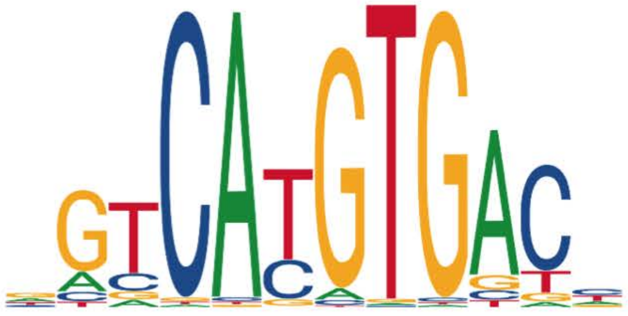

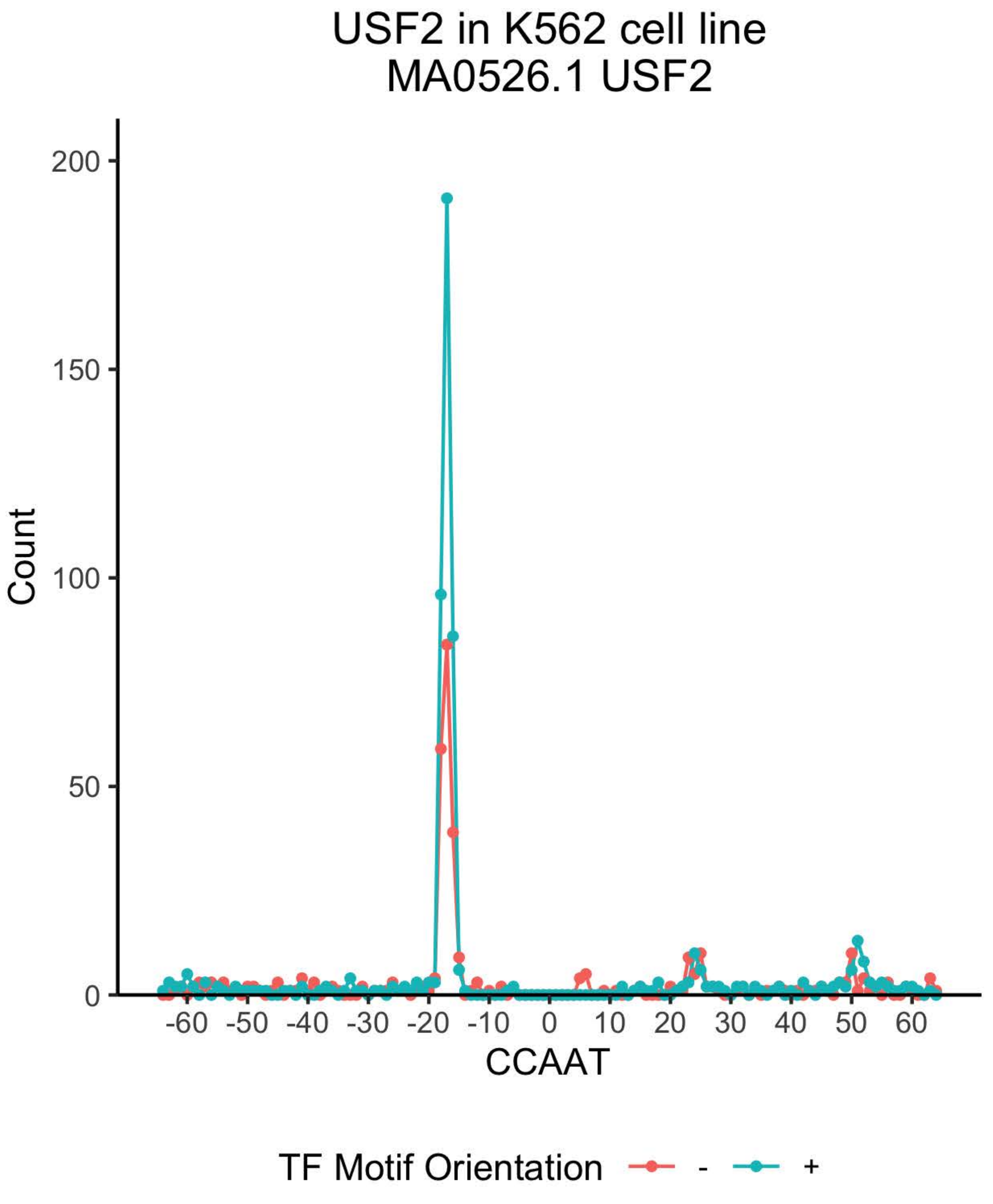

USF2 in K562 cell line
MA0526.1 USF2
Count
200
150
100
50
0
-60 -50 -40 -30 -20 -10 0 10 20 30 40 50 60
CCAAT
TF Motif Orientation
-
+

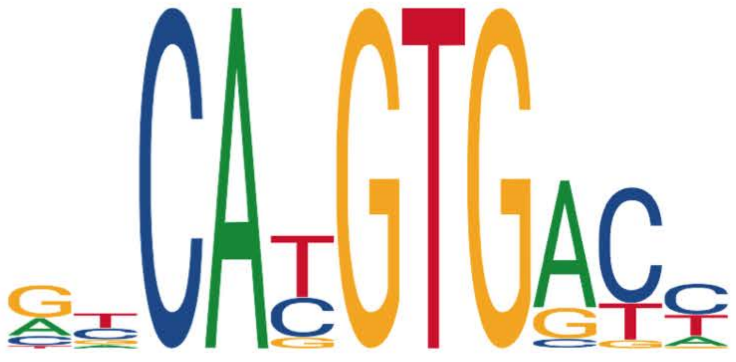

USF2 in K562 cell line
MA0526.2 USF2

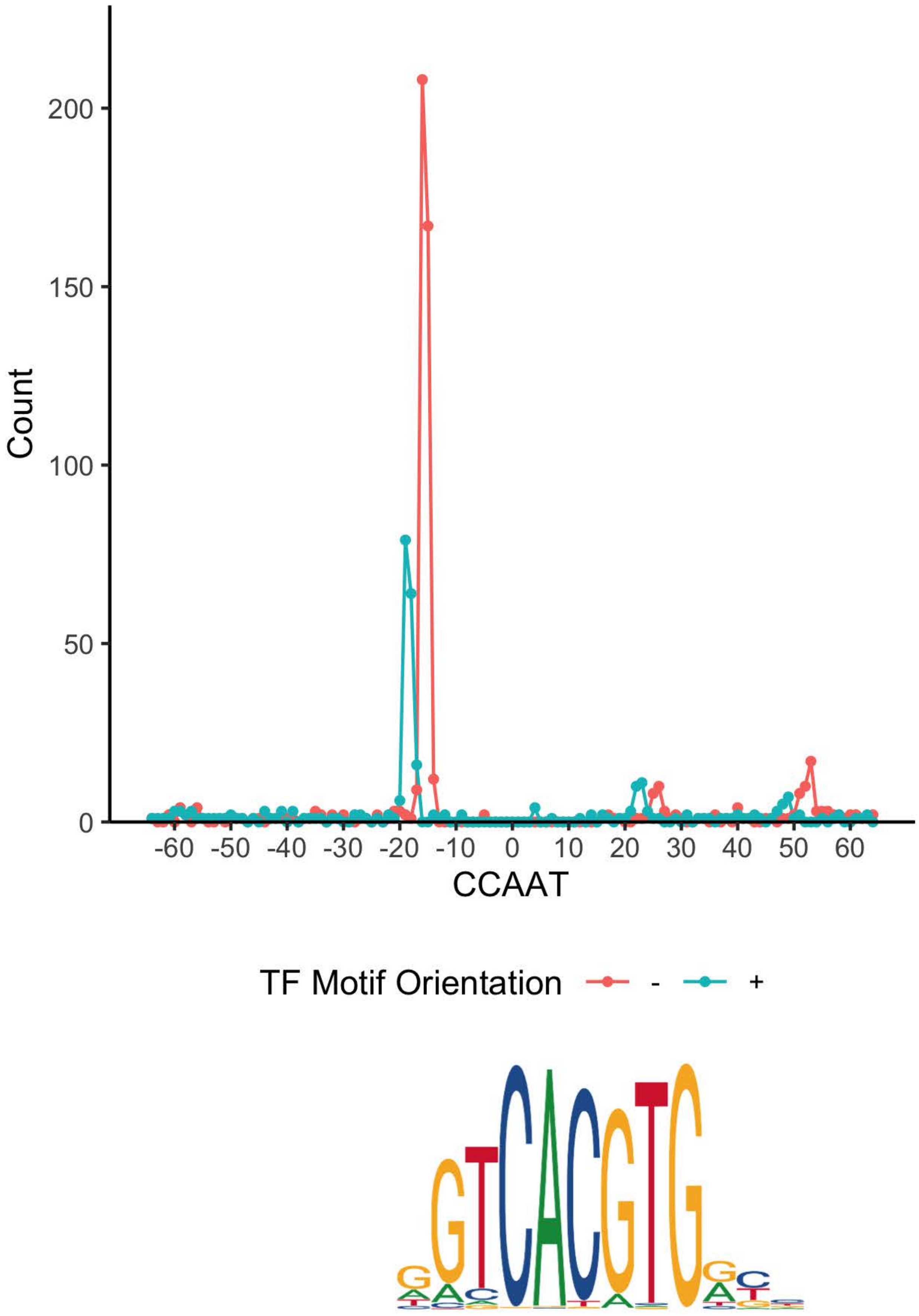

Supplement: S1 Fig — (PDF) [file pcbi.1008488.s010.pdf]
