# Supplementary figures and images for: On the NF-Y regulome as in ENCODE (2019)
Source: PLoS Comput Biol. 2020 Dec 28;16(12):e1008488. doi: 10.1371/journal.pcbi.1008488 (PMC7793273; doi:10.1371/journal.pcbi.1008488)

B

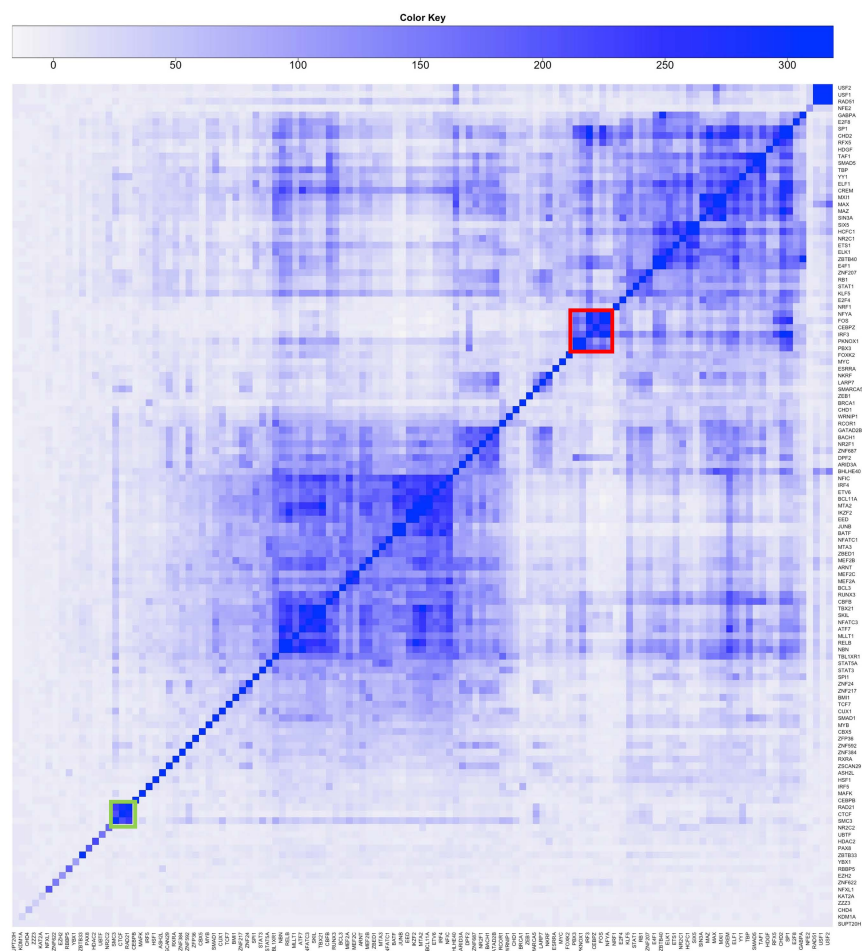

Supplement: S3 Fig — A. Genome-wide representation. B. Analysis restricted to NF-YB-bound regions. (PDF) [file pcbi.1008488.s012.pdf]

A

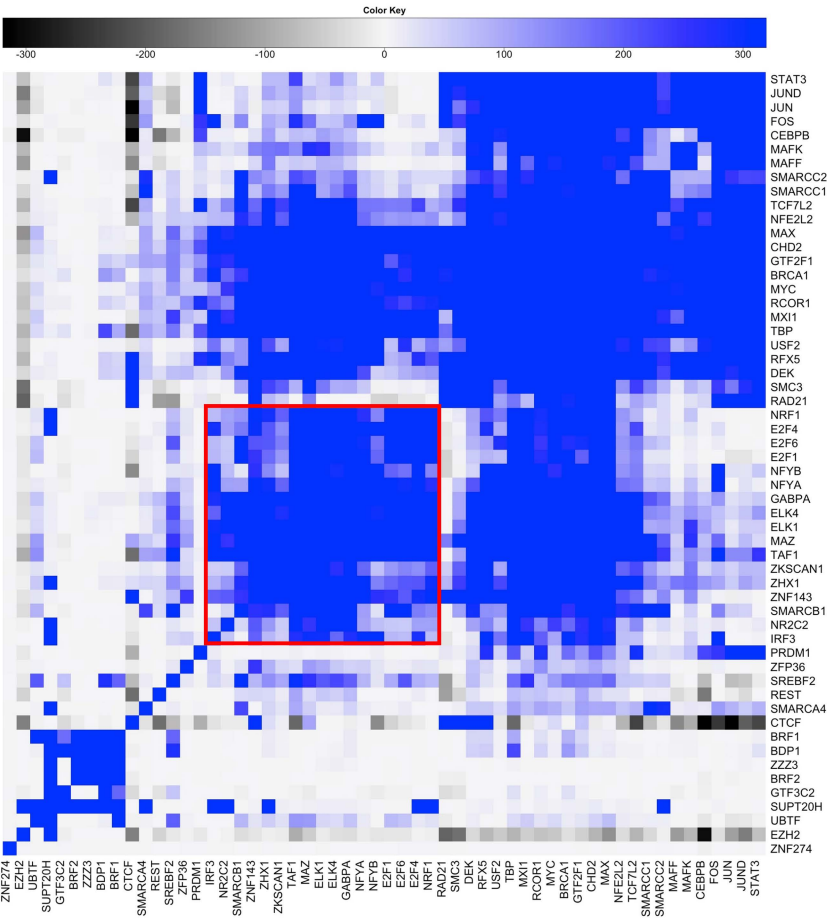

B

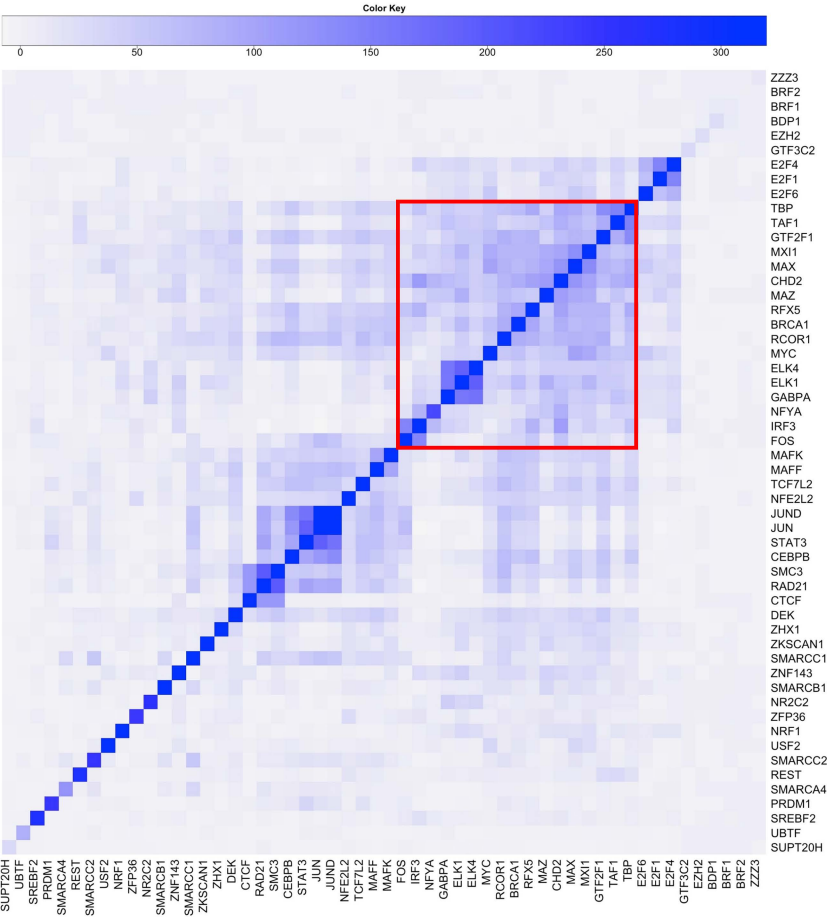

Figure S4

Supplement: S4 Fig — A. Genome-wide representation. B. Analysis restricted to NF-YB-bound regions. (PDF) [file pcbi.1008488.s013.pdf]

Figure S5

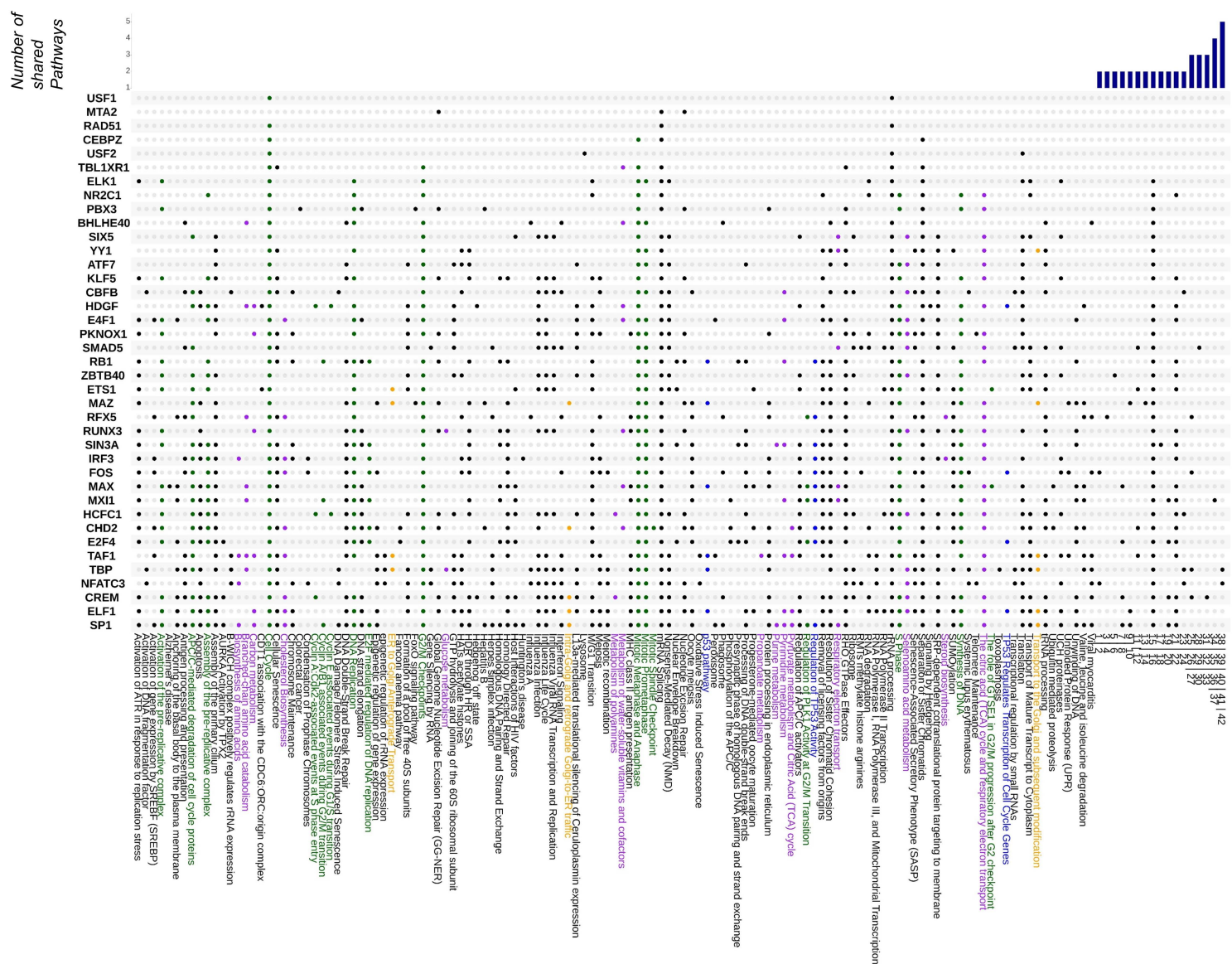

B

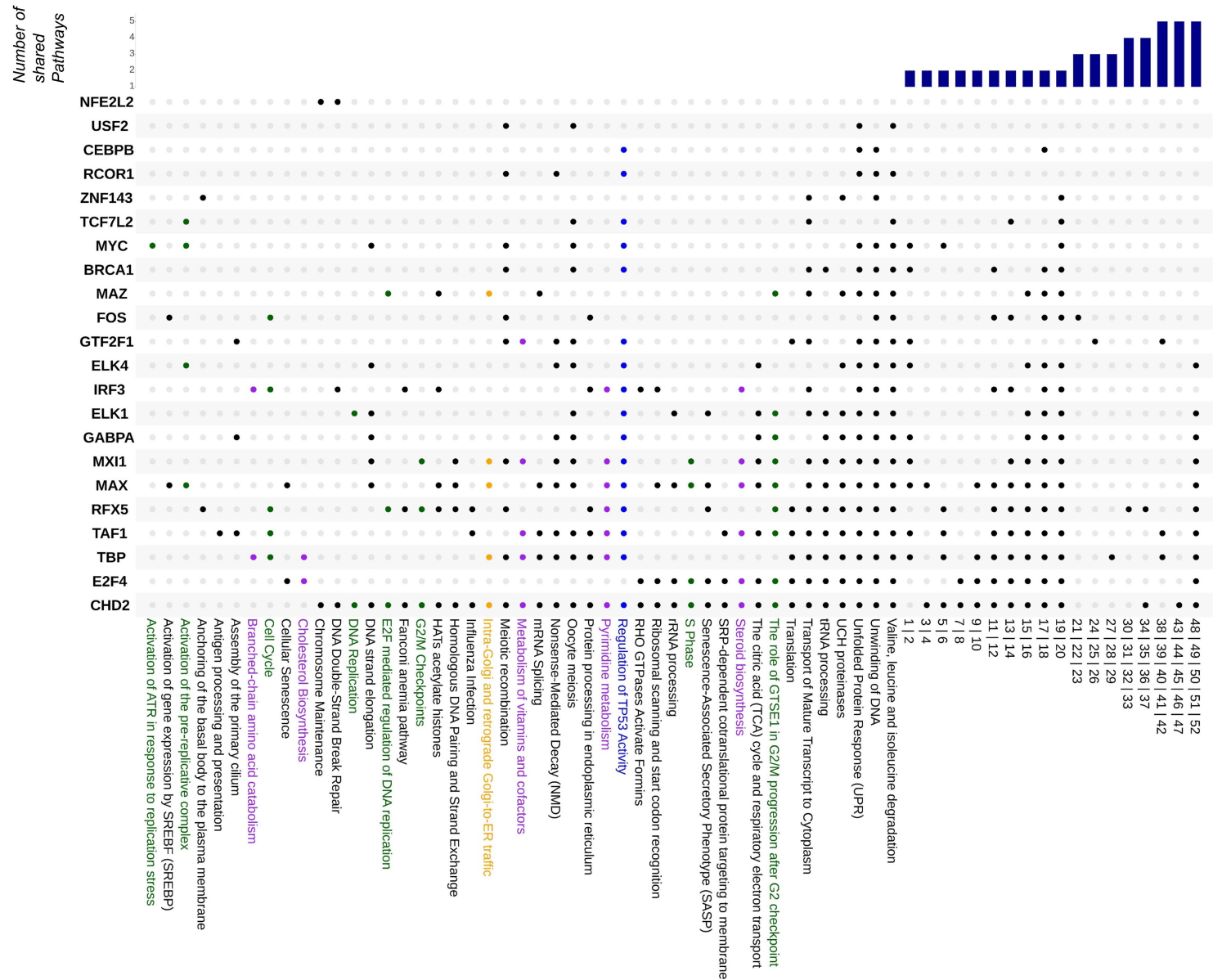

Figure S5

Supplement: S5 Fig — List of factors with significant overlap and/or CCAAT enrichment in A. GM12878 and B. HeLa-S3 cell lines, in ascending order based on the number of associated pathways. Blue bars represent the number of shared pathways among different factors, starting from one. Highlighted dots stand for positive intersection between the factor and the individual pathway. In green, cell-cycle related pathways; in purple, metabolism pathways; in blue, p53-related pathways; in yellow, endomembrane-system related pathways. (PDF) [file pcbi.1008488.s014.pdf]

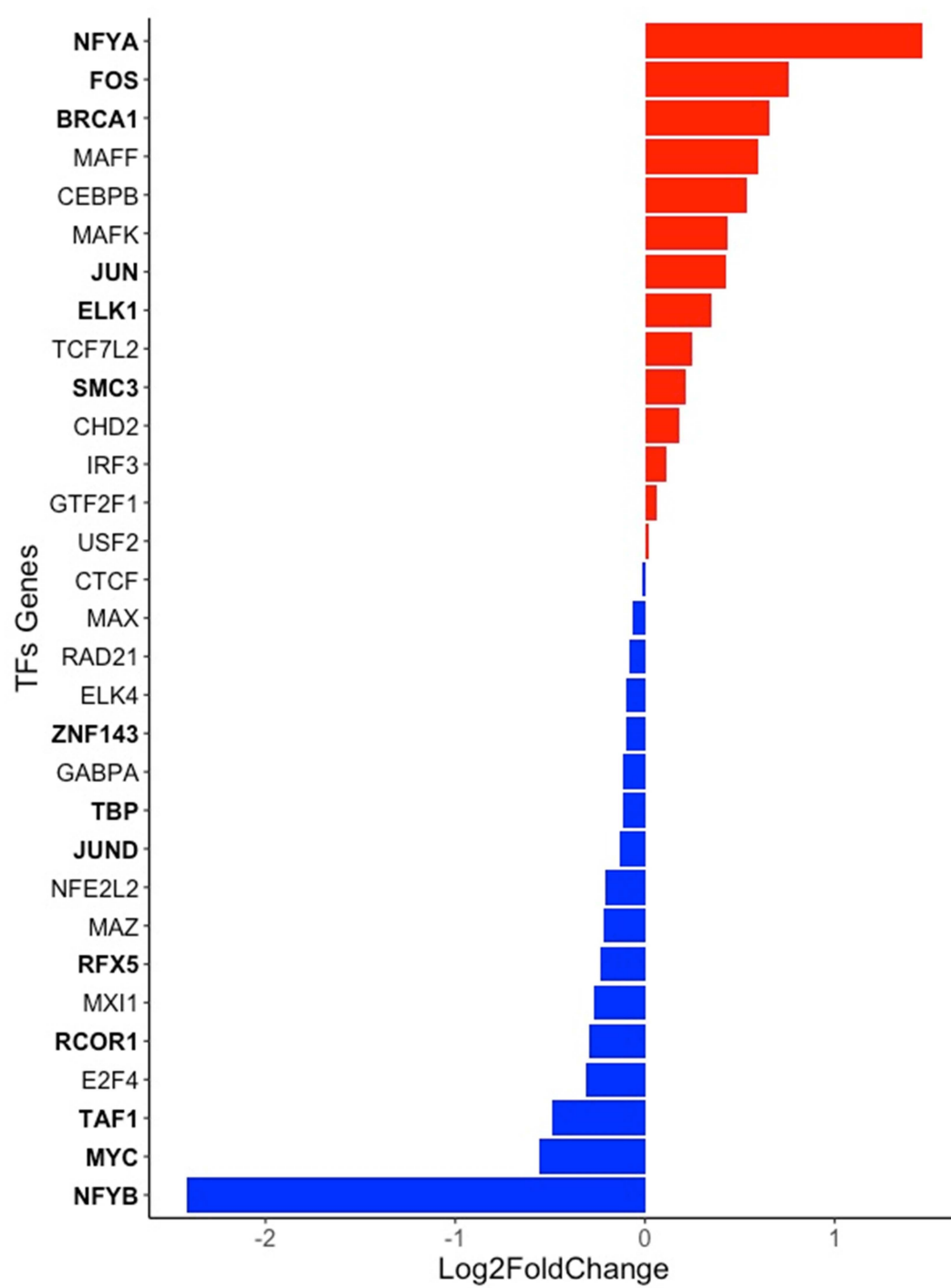

Figure S7

Supplement: S7 Fig — (PDF) [file pcbi.1008488.s016.pdf]

A

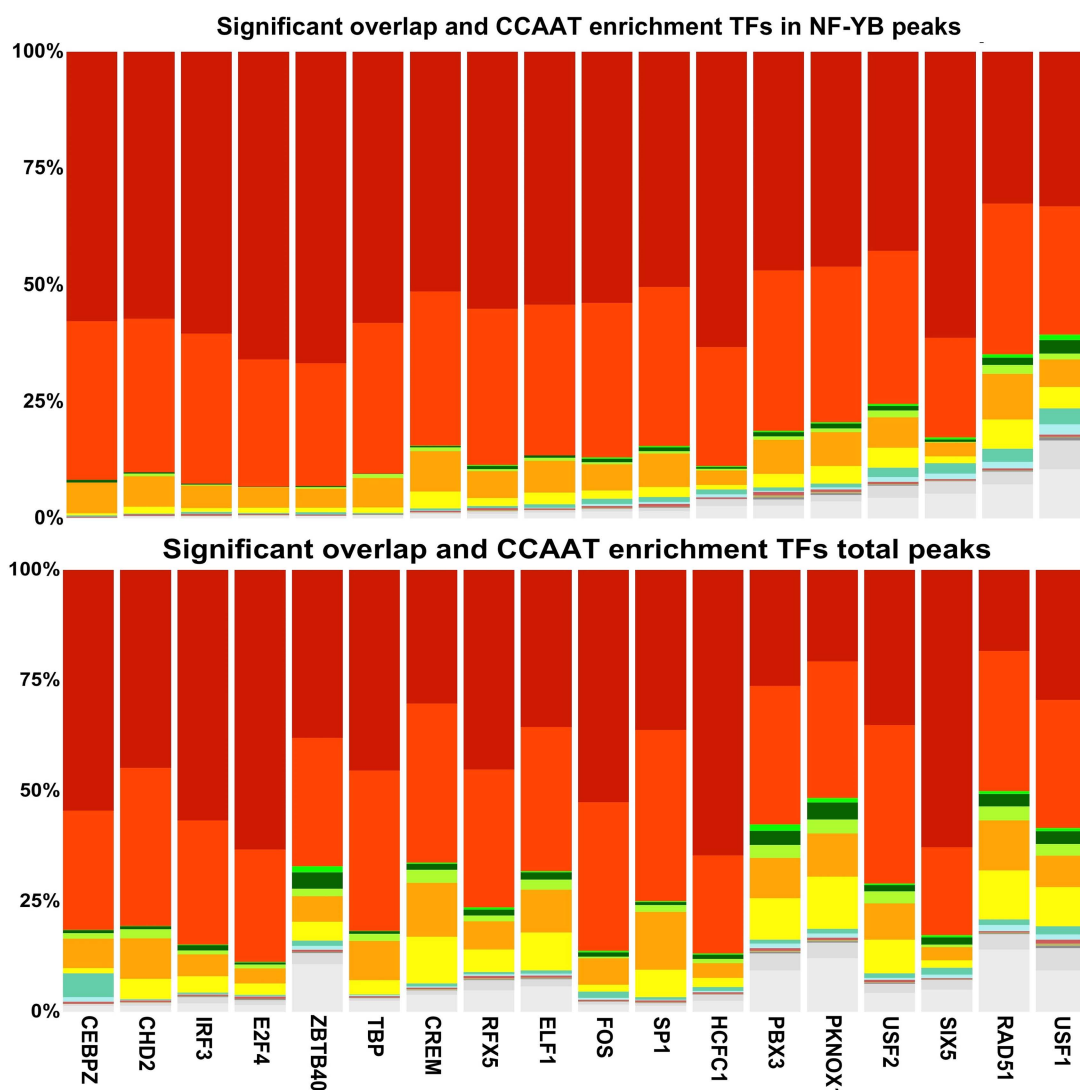

B

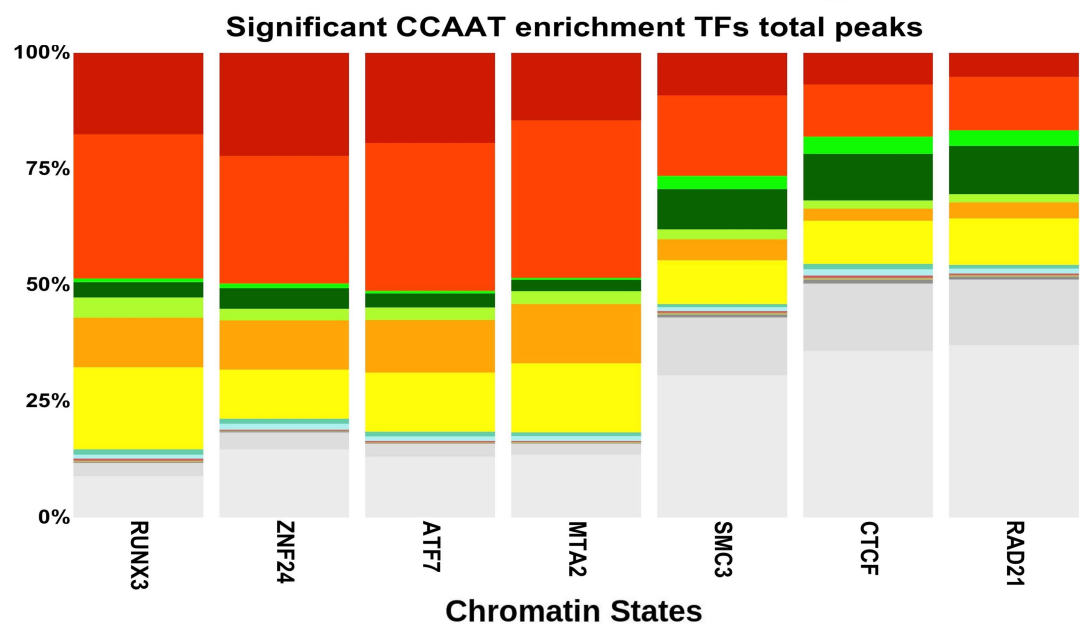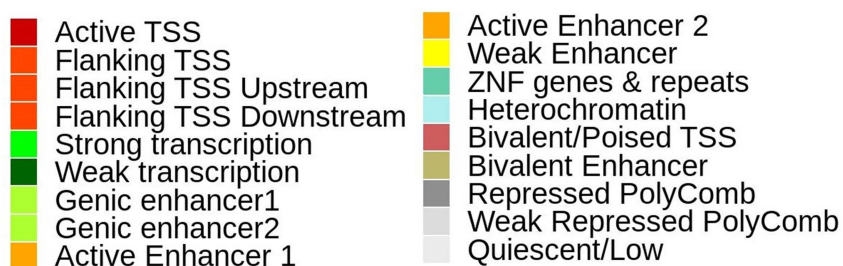

Figure S8

Supplement: S8 Fig — Relative distribution of chromatin states across factors ChIP-seq peak regions. A-B plots include factors with significant overlap with NF-YB and significant CCAAT enrichment. A. distribution of regions co-bound with NF-YB; B. distribution of all regions of factor. C. Distribution of regions of factors with significant CCAAT enrichment but not overlap. (PDF) [file pcbi.1008488.s017.pdf]

A

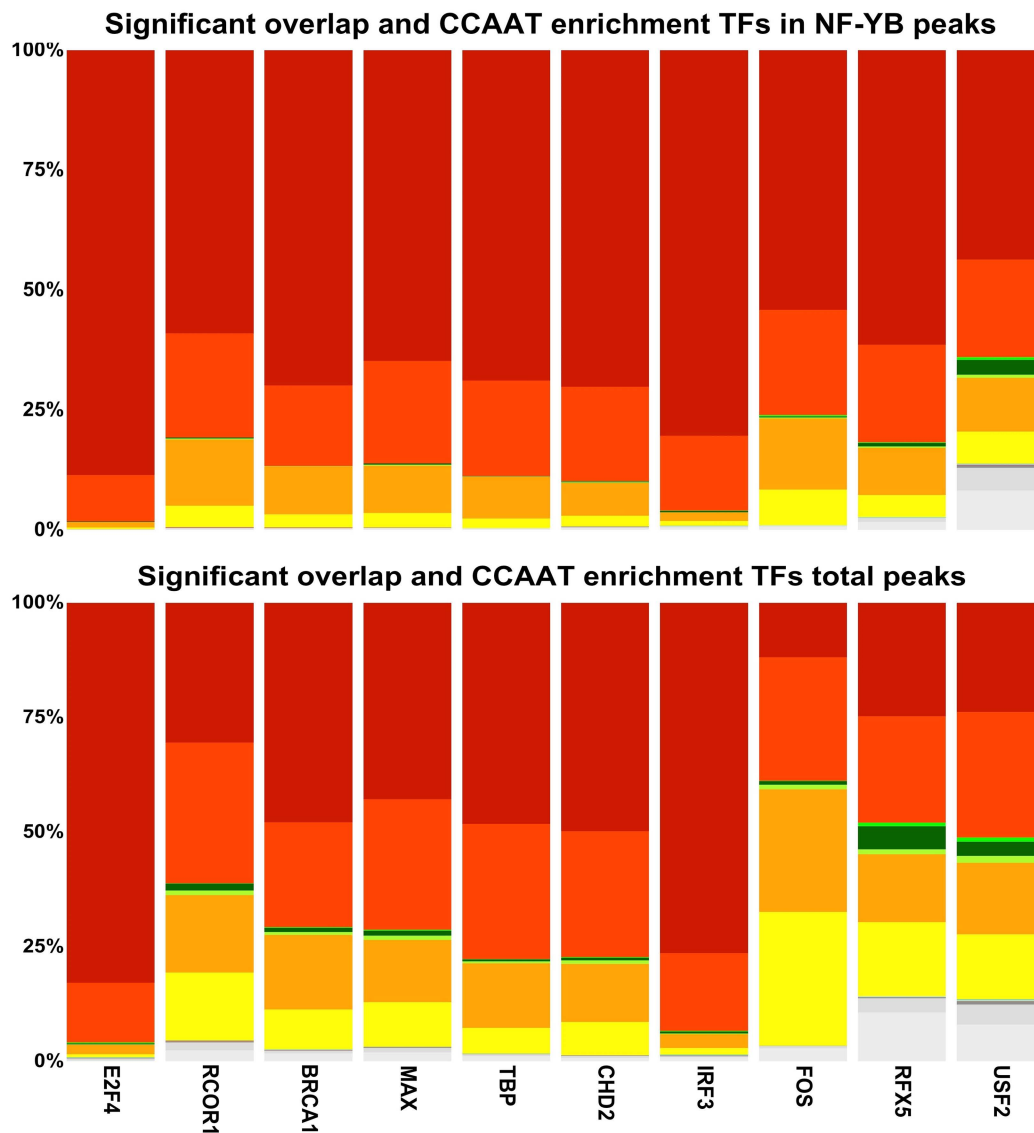

B

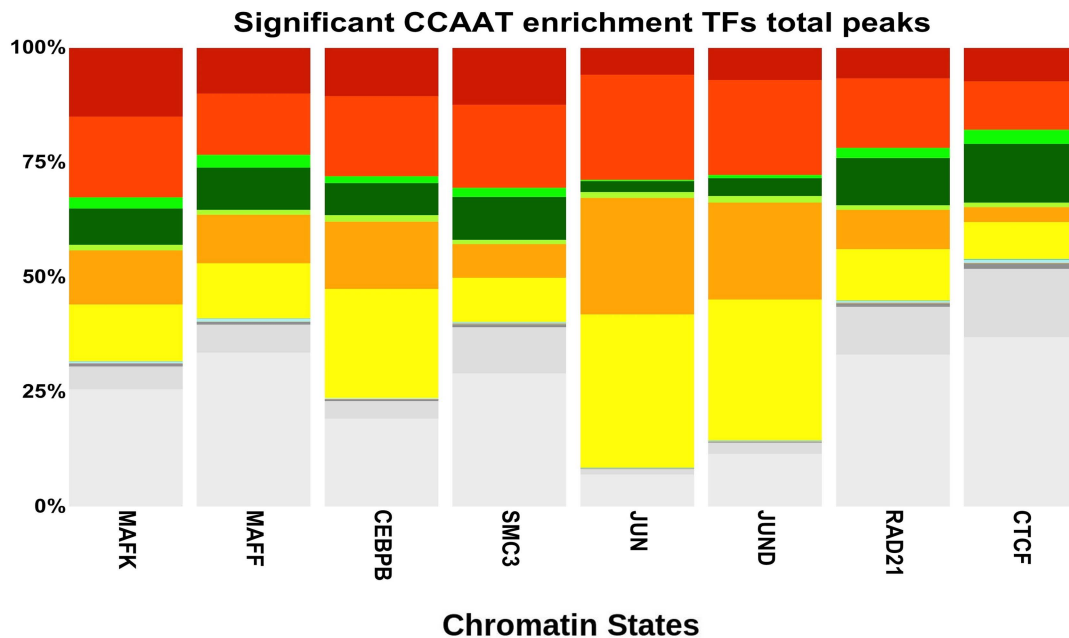

Figure S9

Supplement: S9 Fig — Relative distribution of chromatin states across factors ChIP-seq peak regions. A-B plots include factors with significant overlap with NF-YB and significant CCAAT enrichment. A. distribution of regions co-bound with NF-YB; B. distribution of all regions of factor. C. Distribution of regions of factors with significant CCAAT enrichment but not significant overlap. (PDF) [file pcbi.1008488.s018.pdf]
