# Supplementary material for: On the NF-Y regulome as in ENCODE (2019)
Source: PLoS Comput Biol. 2020 Dec 28;16(12):e1008488. doi: 10.1371/journal.pcbi.1008488 (PMC7793273; doi:10.1371/journal.pcbi.1008488)
Supplement: S6 Fig — Output of Pscan on promoters of up- (left) and down- (right) regulated genes after NF-YB inactivation, showing most enriched TFBS. (PDF) [file pcbi.1008488.s015.pdf]

1622
Up-regulated genes

MA0105.4	NFKB1	3.25364e-06
MA0649.1	HEY2	6.21711e-06
MA0006.1	Ahr::Arnt	6.62988e-06
MA0105.1	NFKB1	7.24461e-06
MA0080.1	SPI1	1.03151e-05
MA0646.1	GCM1	1.05201e-05
MA0139.1	CTCF	3.6282e-05
MA0048.2	NHLH1	3.97153e-05
MA0138.1	REST	4.90291e-05
MA0112.2	ESR1	7.97626e-05
MA0014.2	PAX5	0.000142086
MA0154.1	EBF1	0.0001726
MA0117.1	Mafb	0.000253525
MA0028.1	ELK1	0.000282936
MA0028.2	ELK1	0.000398396
MA0076.1	ELK4	0.000413097
MA0761.1	ETV1	0.000413318
MA0258.2	ESR2	0.000736602
MA0686.1	SPDEF	0.000861443
MA0062.1	GABPA	0.000922408
MA0159.1	RARA::RXRA	0.00114424
MA0060.1	NFYA	0.00117684
MA0759.1	ELK3	0.00122924
MA0764.1	ETV4	0.00134509
MA0595.1	SREBF1	0.00165507
MA0475.1	FLI1	0.00167421
MA0138.2	REST	0.00172614
MA0641.1	ELF4	0.00222286
MA0464.2	BHLHE40	0.00245357
MA0500.1	Myog	0.00369061
MA0473.2	ELF1	0.0039104
MA0734.1	GLI2	0.0040257
MA0499.1	Myod1	0.00421015
MA0154.2	EBF1	0.00564819
[illegible]	[illegible]	[illegible]

1602
Down-regulated Genes

Matrix ID	Matrix Name	P-value
MA0060.1	NFYA	6.40826e-78
MA0632.1	Tcfl5	9.92427e-59
MA0502.1	NFYB	1.06542e-56
MA0516.1	SP2	5.83111e-56
MA0506.1	NRF1	1.772e-54
MA0060.2	NFYA	2.16175e-54
MA0470.1	E2F4	5.55302e-54
MA0131.2	HINFP	5.76893e-54
MA0685.1	SP4	2.41372e-51
MA0062.2	Gabpa	3.47727e-49
MA0740.1	KLF14	3.72779e-48
MA0131.1	HINFP	7.32211e-47
MA0024.2	E2F1	2.22144e-45
MA0527.1	ZBTB33	1.72823e-44
MA0732.1	EGR3	5.09126e-44
MA1099.1	Hes1	5.90183e-44
MA0472.2	EGR2	9.21157e-44
MA0079.3	SP1	1.11079e-43
MA0039.2	Klf4	1.14848e-41
MA0028.2	ELK1	1.2781e-41
MA0076.1	ELK4	3.31914e-41
MA0765.1	ETV5	8.45433e-41
MA0469.1	E2F3	1.03426e-40
MA0615.1	Gmeb1	1.08186e-40
MA0003.1	TFAP2A	1.9077e-38
MA0741.1	KLF16	7.84687e-38
MA0162.1	Egr1	1.44079e-37
MA0746.1	SP3	2.07441e-37
MA0759.1	ELK3	6.62075e-37
MA0024.3	E2F1	8.13305e-37
MA0162.2	EGR1	8.64816e-37
MA0076.2	ELK4	3.5554e-36
MA0761.1	ETV1	4.67622e-36

Figure S6
